# Altered gene expression changes in *Arabidopsis *leaf tissues and protoplasts in response to *Plum pox virus *infection

**DOI:** 10.1186/1471-2164-9-325

**Published:** 2008-07-09

**Authors:** Mohan Babu, Jonathan S Griffiths, Tyng-Shyan Huang, Aiming Wang

**Affiliations:** 1 Southern Crop Protection and Food Research Centre, Agriculture and Agri-Food Canada, 1391 Sandford St., London, Ontario, N5V 4T3, Canada; 2 Department of Biology, The University of Western Ontario, Biological & Geological Building, 1151 Richmond St., London, Ontario, N6A 5B7, Canada; 3 Department of Molecular Genetics, The University of Toronto, M5S 1A8, Canada; 4 Department of Botany, The University of British Columbia, Vancouver, V6T 1Z4, Canada

## Abstract

**Background:**

Virus infection induces the activation and suppression of global gene expression in the host. Profiling gene expression changes in the host may provide insights into the molecular mechanisms that underlie host physiological and phenotypic responses to virus infection. In this study, the *Arabidopsis *Affymetrix ATH1 array was used to assess global gene expression changes in *Arabidopsis thaliana *plants infected with *Plum pox virus *(PPV). To identify early genes in response to PPV infection, an *Arabidopsis *synchronized single-cell transformation system was developed. *Arabidopsis *protoplasts were transfected with a PPV infectious clone and global gene expression changes in the transfected protoplasts were profiled.

**Results:**

Microarray analysis of PPV-infected *Arabidopsis *leaf tissues identified 2013 and 1457 genes that were significantly (*Q *≤ 0.05) up- (≥ 2.5 fold) and downregulated (≤ -2.5 fold), respectively. Genes associated with soluble sugar, starch and amino acid, intracellular membrane/membrane-bound organelles, chloroplast, and protein fate were upregulated, while genes related to development/storage proteins, protein synthesis and translation, and cell wall-associated components were downregulated. These gene expression changes were associated with PPV infection and symptom development. Further transcriptional profiling of protoplasts transfected with a PPV infectious clone revealed the upregulation of defence and cellular signalling genes as early as 6 hours post transfection. A cross sequence comparison analysis of genes differentially regulated by PPV-infected *Arabidopsis *leaves against uniEST sequences derived from PPV-infected leaves of *Prunus persica*, a natural host of PPV, identified orthologs related to defence, metabolism and protein synthesis. The cross comparison of genes differentially regulated by PPV infection and by the infections of other positive sense RNA viruses revealed a common set of 416 genes. These identified genes, particularly the early responsive genes, may be critical in virus infection.

**Conclusion:**

Gene expression changes in PPV-infected *Arabidopsis *are the molecular basis of stress and defence-like responses, PPV pathogenesis and symptom development. The differentially regulated genes, particularly the early responsive genes, and a common set of genes regulated by infections of PPV and other positive sense RNA viruses identified in this study are candidates suitable for further functional characterization to shed lights on molecular virus-host interactions.

## Background

Systemic virus infection in plants relies on complex molecular interactions between the invading virus and the host plant [1-4]. Through such interactions, the virus recruits diverse host gene products (host factors) and metabolites for the translation and replication of its genome, which disrupts the normal biological processes of the host [4-6]. On the other hand, the expression of resistance genes in the infected host is also triggered by specific molecular interactions to combat the infection. Thus, virus infection induces the activation and suppression of global gene expressions in the host [7-16]. These gene expression changes are the molecular basis of general stress and defence-like responses, viral pathogenesis and host symptom development.

*Plum pox virus *(PPV) is a member of potyviruses, and is considered the most devastating viral pathogen of many stone-fruit species such as *Prunus persica*, *P. persica var. nucipersica*, and *P. domestica *[6]. Despite numerous extensive screenings for genetic resistance against the virus, no natural resistant germplams are currently available for conventional breeding programs. Very little is known about the effects of PPV on host cells at the molecular level. A better understanding of molecular PPV-plant interactions would help identify key host factors required for PPV infection and assist in the development of novel recessive resistance to PPV [6].

To date, a major problem in dissecting molecular virus-host interactions is the progressive and asynchronous nature of the infection in leaves or in whole plants [1,16-18]. In systemically infected plants, some cells may be infected earlier, while others may be infected later. Some parts may have higher virus titres while others may have fewer viruses or even virus-free. In the whole plant, it may take from days to weeks for the invading virus to spread from the infection site and develop systemic symptoms in the intact plant. Thus, the infected tissues contain cells at various infection stages or with viral concentrations in a wide range. As a result, temporal and spatial expression changes of host genes are very difficult to investigate. In the absence of a synchronous virus infection system and a global gene expression profiling technology, Maule and colleagues elegantly assessed the expression of selected host genes in individual cells across an advancing infection front using a cell biological approach (immunocytochemistry and *in situ *hybridization) (see review by Maule et al.) [17]. To date, microarray technology has been adapted to profile genome-wide gene expression changes in plants in response to infections of a number of positive sense RNA viruses [7-10,12-16]. More recently, Yang *et al*. [16] creatively dissected infection foci away from the non-infected tissue into four distinct zones using an infectious clone of *Turnip mosaic virus *(TuMV) tagged with green fluorescence protein (GFP) (TuMV-GFP) and profiled global gene expression changes in each of these zones with microarray, making it possible to spatially and temporally correlate virus infection and accumulation with host gene expression. Collectively these works largely advance our understanding of virus-host interactions.

Considering the availability of whole genome microarrays, and increasing use of genetic and biochemical approaches in archetypal plants like *Arabidopsis thaliana *to explore the molecular interactions between viruses and their host plants [19], we used an *Arabidopsis *ATH1 Affymetrix array and analyzed global gene expression changes in *Arabidopsis *leaves infected with PPV at 17 days post inoculation (dpi). Further, we generated *Arabidopsis *synchronized single-cell protoplasts, transfected with a PPV infectious clone and assayed global gene expression changes in the protoplasts at 3, 6 and 12 hr post transfection (hpt). The aim of this study was to investigate general gene expression changes in infected leaf tissues and their association with PPV infection and symptom development, and to identify putative host gene factors differentially regulated upon PPV infection, particularly those at early time points involved in defence and viral pathogenesis.

## Results and discussion

### Symptom development in PPV-infected *Arabidopsis *plants

**Figure 1:**
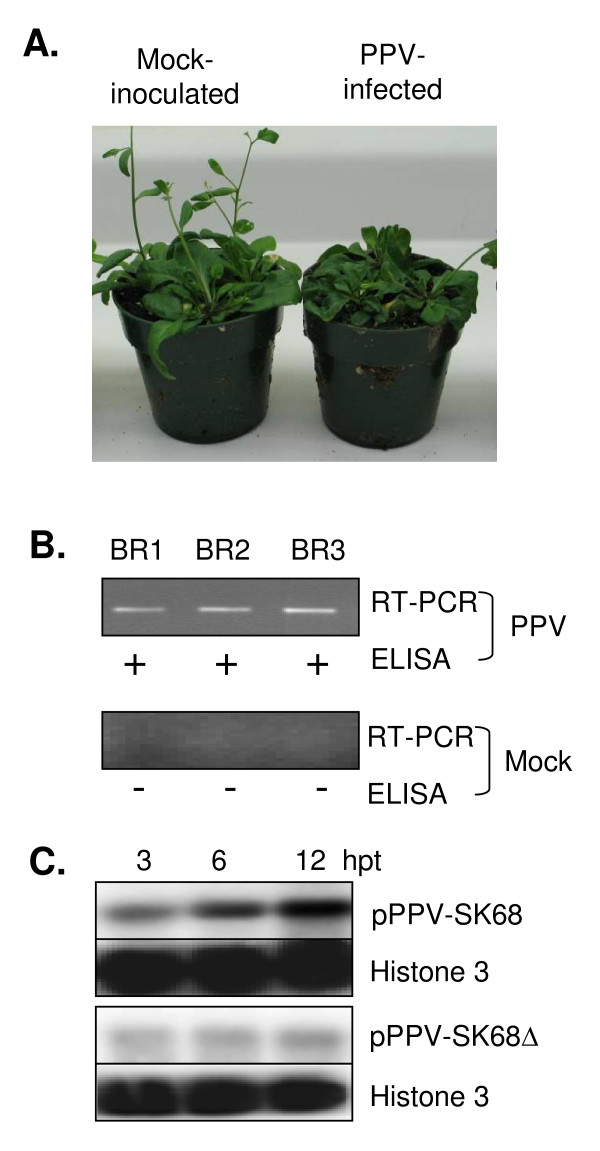
**Systemic infection and detection of PPV in *Arabidopsis thaliana *plants**. (A) Symptoms on mock-inoculated (left) and PPV-infected (right) plants 17 days post inoculation (dpi). (B) RT-PCR and ELISA analysis of PPV-infected and mock-inoculated leaf tissues at 17 dpi. BR1, BR2, BR3, represents three independent biological replicates of PPV-infected and mock-inoculated leaf tissues, respectively. RT-PCR amplification of a 467 bp fragment corresponding to a segment of the PPV genome (nt 9140 to 9607), as tested using 25 cycles of amplification. +, ELISA positive; -; ELISA negative. (C) sqRT-PCR amplification of a cDNA fragment of the PPV genome isolated from *Arabidopsis *protoplasts transfected with pPPV-SK68 and pPPV-SK68Δ at 3, 6 and 12 hours post transfection (hpt), respectively. Histone 3 gene was used as a loading control. RT-PCR amplification for both panel B and C were carried out using the same RNA samples that were used in microarray hybridizations.

Susceptibility of *Arabidopsis *accession Col-0 to the infection of a PPV-D strain isolated in Canada was monitored by recording the development of symptoms and the virus titre. No typical viral symptoms such as mosaic, chlorosis, ringspot, vein yellowing, or leaf curling were visible in plants inoculated with PPV at 17 dpi and up to 42 dpi. This is consistent with the recent finding that *Arabidopsis *plants infected by two PPV-D isolates displayed mild or no symptoms [20]. At 17 dpi, the most obvious phenotypes observed in this study were mild growth retardation, slightly delayed flowering and bolting (Figure 1A). These phenotypes were not documented for *Arabidopsis *accession Col-0 infected with two PPV-D isolates but were very similar to accession Bay-O inoculated with one of the two PPV-D isolates [20]. Considering the diverse phenotypes of different accession-isolate combinations, the observed phenotypic variation between the two studies is likely due to the PPV isolates used. Using reverse transcription-polymerase chain reaction (RT-PCR) and enzyme-linked immunosorbent assay (ELISA), PPV was detected in newly developed rosette leaves above the inoculated leaves (Figure 1B) at 17 dpi. Since PPV-induced symptoms appeared and the virus was detected by RT-PCR and ELISA at 17 dpi in the infected leaf tissues, it was expected that changes in host gene expression would be altered at this time point. In addition, we also inoculated *Nicotiana benthamiana *plants with PPV. Infected leaves showed chlorosis and severe mosaic symptoms as early as 7 dpi (data not shown). At 17 dpi, the virus titre in *N. benthamiana *was at least 100 times or higher than that in *Arabidopsis *(data not shown), suggesting that *Arabidopsis *may have stronger basal or inducible resistance to PPV than *N. benthamiana*.

### Over- or under-represented functional categories of differentially expressed *Arabidopsis *genes in PPV-infected leaf tissues

At 17 dpi, newly expanded PPV-infected rosette leaves from the inoculated plants and the corresponding leaves from the mock-inoculated plants were sampled for microarray hybridizations. Consistency of the microarray data was assessed by comparing the mean normalized signal intensities obtained from the mock-inoculated control samples across three independent biological replicates. A high correlation (0.96 – 0.98) among the mock inoculated control samples was observed, indicating low biological variability between the replicates.

Gene expression data were analyzed by one-way ANOVA to identify differentially expressed genes in PPV-infected rosette leaves from the inoculated plants relative to leaves from the mock-inoculated plants. Using stringent selection criteria with the Benjamini and Hochberg false discovery rate (FDR) of 5% [21], corresponding to *Q *≤ 0.05 [22], ~31.4% of the non-redundant (nr) genes (7,151 out of the 22,810 *Arabidopsis *genes printed on the chip) were identified as significantly differentially expressed (either induced or repressed) in response to PPV infection. These 7,151 genes were further filtered using a 2.5-fold increase or decrease in signal intensity. Using this fold change cut-off, we identified 2,013 and 1,457 *Arabidopsis *genes that were significantly induced and repressed by PPV in systemically infected tissues, respectively.

**Figure 2:**
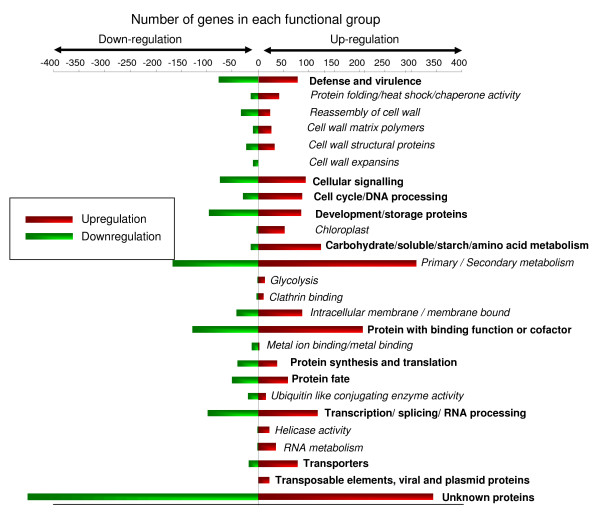
**Functional distribution of *Arabidopsis *genes significantly induced and repressed in PPV-infected leaves**. The genes were grouped following the methods of the MIPS *Arabidopsis *classification scheme. Genes whose function has not been determined were grouped under "unknown function". Number of genes identified in each functional group is indicated on the x-axis. Genes that belong to major functional categories are highlighted in bold and the subcategories within a major functional category are highlighted in italics.

Each identified gene was then assigned to a functional class according to the *Arabidopsis *MIPS (Munich Information Centre for Protein Sequences) functional classification scheme (Figure 2). With the information gathered from TAIR (The *Arabidopsis *Information Resource) database (please see Availability & requirements for more information), we were able to assign a putative function for 54.3% of the total 22,810 genes on the array, while the remaining 45.7% were undefined as they were annotated as "unknowns or orphans" (Table 1). The expression levels of *Arabidopsis *genes significantly induced or repressed by PPV were assigned to 12 different major functional groups. In some major functional groups, genes were further separated into subcategories to simplify the data for biological interpretation [see Additional file 1]. The number of genes in each functional category was subjected to Fisher's exact test [23,24] to determine if genes involved in certain biological processes were over- or under-represented (*P *≤ 0.001). The functional groups significantly over-represented in the set of upregulated genes were carbohydrate/soluble sugar/starch/amino acid metabolism (*P *< 7E-12), primary and secondary metabolism (*P *< 2.2E-07), intracellular membrane/membrane-bound organelle (*P *< 4.5E-06), clathrin binding (*P *< 1.2E-05), glycolysis (*P *< 2.0E-05), protein folding/heat shock/chaperone activity (*P *< 5.2E-06), cell wall-associated transcripts (*P *< 1.6E-05), RNA metabolism (*P *< 1.9E-05), chloroplast (*P *< 1.8E-06), protein fate (*P *< 3.5E-07), ubiquitin-like conjugating enzyme activity (*P *< 4.1E-05) and development/storage proteins (*P *< 4.8E-08). Among the downregulated genes, the over-represented functional groups were metal ion binding/metal binding (*P *< 1.6E-07), development/storage proteins (*P *< 3.3E-06), cell wall-associated transcripts (*P *< 0.00013) and protein synthesis and translation (*P *< 1.4E-43). Genes involved in primary/secondary metabolism (*P *< 1.4E-06) and intracellular membrane/membrane-bound organelle (*P *< 1.3E-05) functional groups were significantly under-represented.

**Table 1:** Assignment of upregulated and downregulated genes in each functional group identified in PPV-infected *Arabidopsis *leaves and protoplasts.

S.No	Functional category	Number of genes on the array ^a^	PPV-infected leaves Upregulated/Downregulated [Number (%)/Number (%)] ^b^	pPPV-SK68-transfected protoplasts Upregulated/Downregulated [Number (%)/Number (%)] ^b^
A	Defence and virulence	1488	204 (13.7)/165 (11.1)	32 (2.15)/33 (2.22)
B	Cellular communication/Signal transduction mechanism/transmembrane signal transduction	1657	94 (5.67)/74 (4.47)	16 (0.97)/18 (1.09)
C	Cell Cycle/DNA processing/chromatin regulation and cytoskeleton reorganization	771	87 (11.3)/30 (3.89)	10 (1.30)/9 (1.17)
D	Development/storage proteins	657	138 (21)/100 (15.2)	20 (3.04)/20 (3.04)
E	Metabolism	4238	548 (12.9)/226 (5.3)	35 (0.83)/41 (0.97)
F	Proteins with binding function or cofactor requirement	921	210 (22.8)/139 (15.1)	20 (2.17)/22 (2.39)
G	Protein synthesis and translation	314	37 (11.8)/39 (12.4)	1 (0.32)/1 (0.32)
H	Protein fate	647	77 (11.9)/69 (10.6)	9 (1.39)/12 (1.85)
I	Transcription/splicing/RNA processing/modification	1013	175 (17.3)/102 (10.1)	32 (3.16)/29 (2.86)
J	Transporters	563	78 (13.9)/19 (3.4)	7 (1.24)/7 (1.24)
K	Transposable elements, viral and plasmid proteins	124	22 (17.7)/44 (35.5)	8 (6.45)/21 (16.94)
L	Unknown proteins	10417	343 (3.3)/450 (4.3)	73 (0.70)/91 (0.87)

^a ^Number of genes in a given category represented on the chip.

^b ^Percentage was calculated based on the number of differentially regulated genes divided by the total number of genes in the functional category on the chip.

The analysis showed that that the number of upregulated genes was more than that of downregulated genes in PPV-infected *Arabidopsis *leaves, probably reflecting more diverse genes induced by PPV infection. This notion is supported by a disproportionate allocation in the number of altered genes in the infected leaf tissues belonging to major functional groups of metabolism, transcription/splicing/RNA processing proteins, defence and development/storage proteins.

### Gene expression changes in metabolism, chloroplast and protein fate in PPV-infected *Arabidopsis *leaf tissues likely involved in symptom development

Virus infections often disturb the general metabolism in the host. This includes the accumulation of soluble sugars (e.g., sucrose synthase) and starch (e.g., starch synthase), an increase in the rate of respiration, a decrease in the rate of photosynthesis and elevated levels of amino acid synthesis (e.g., amino acid permease and aminotransferase) [25-30]. In this study, we found that in PPV-infected leaves, 23% (124/548) of genes associated with metabolism of starch, soluble sugars and amino acids and 2.7% (15/548) of genes related to glycolysis showed elevated expression levels [see Additional file 1]. Appearance of disease symptoms such as chlorosis on the *Cucumber mosaic virus *(CMV)-infected leaf tissues in cucumber was correlated with increased glycolysis, phosphoenol pyruvate phosphate, respiration and starch accumulation, decreased photosynthesis and reduction in total protein synthesis [27]. The increased expression of these genes may result from the physical blockage of the transport path, the inactivation of sugar transport proteins or the induction of cell wall invertase [31-33]. Indeed, several cell wall invertase genes were also induced in PPV-infected leaves in this study. These data indicate that a series of intercrossing pathways involved in sugar metabolism was differentially regulated by PPV infection. It is possible that altered gene expression pathways involved in sugar metabolism might contribute to the mild symptom development observed in PPV-infected *Arabidopsis *leaves.

In addition to soluble sugars, starch and amino acids, PPV also induced 16% (87/548) of membrane-associated proteins, including proteins associated with integral membrane, plasma membrane, endoplasmic reticulum (ER), vacuole, mitochondria and peroxisomes [see Additional File 1]. Recently, membrane proteins have also been found to be upregulated in *N. benthamiana *plants infected with *Tomato ringspot virus *(ToRSV) and PPV [13]. For example, genes encoding proteins such as plasma membrane polypeptide, putative integral membrane, COP-coated vesicle membrane, clathrin protein and mitochondrial malate dehydrogenase were induced in PPV-infected *Arabidopsis *leaves that have similar functional identity to those upregulated in *N. benthamiana *plants infected with ToRSV and PPV. It is believed that many positive-strand RNA viruses replicate their genome in association with cellular membranes. Viral proteins that are required for the formation of viral replication complexes (VRCs) are often shown to be located in ER [34-36]. It would be interesting to investigate whether these upregulated membrane proteins are co-localized to VRCs. Characterization of such host membrane factors will provide useful information regarding molecular mechanisms governing membrane specificity for viral RNA replication.

Of the upregulated genes coding for nuclear or cytosolic proteins in PPV-infected *Arabidopsis *leaves, two OGTs (O-linked N-acetyl glucosamine transferase), named SEC (SECRET AGENT) and SPY (SPINDLY, a gibberellin response protein), which can potentially posttranslationally modify the viral capsid protein (CP), are very interesting. Study by Chen et al. [37] has shown that the N-terminus of the CP of PPV is modified with O-linked N-acetyl glucosamine in *N. clevelandii*, *N. benthamiana *and *Arabidopsis*, indicating that the modifications are not host specific. In *Arabidopsis*, this modification is catalyzed by SEC but not by SPY. Such modification is not essential but plays a role in the infection process [37]. SEC and SPY have partial functional redundancy in plants [37,38]. It is not clear whether the upregulation of SPY in PPV-infected *Arabidopsis *is a compensation for the loss of SEC that is used for the modification of the PPV CP.

We also observed as many as 52 chloroplast genes that were upregulated in PPV-infected leaf tissues [see Additional file 1]. These represent approximately 60% of the chloroplast genes in the *Arabidopsis *genome [39]. Several studies suggest that differential regulation of chloroplast gene expression is correlated to viral symptoms such as mosaic and chlorosis [13,16]. Recently, Dardick [13] has reported that ToRSV or PPV infection in *N. benthamiana *leaves induces severe mosaic and chlorosis symptoms. However, the infection of this host with *Prunus necrotic ringspot virus *(PNRSV) hardly induces any symptoms. Using potato cDNA chips, it has been shown that infections of ToRSV and PPV result in the suppression of 34% (83/243) of chloroplast-related genes, while no common genes are among the induced genes in ToRSV- and PPV-infected *N. benthamiana*. In contrast to other two viruses, PNRSV infection downregulates 67% (10/15) and upregulates 33% (5/15) of the chloroplast-related genes [13]. Analogous to PNRSV-infected *N. benthamiana*, PPV-infected *Arabidopsis *leaves did not show typical viral symptoms such as mosaic and chlorosis. The induction of such a large number of chloroplast-related genes in *Arabidopsis *may help maintain the normal function and morphology of the infected leaf and counteract possible detrimental effects resulting from PPV infection.

In virus infected plants, protein activities are often altered through posttranslational modifications such as ubiquitination [4]. In the current study, about 9% (60 genes) of the upregulated protein fate genes encoding proteins associated with the 26S proteasome were very significant (*Q *≤ 0.05) and over-represented (*P *< 3.5E-07). Of the seven upregulated 26S proteasome genes, two genes code for AAA-ATPase subunits including RPT3 that reside in the mature 26S proteasome [16] and five genes including *Rpn1*, *3 *and *6 *are involved in ubiquitination. Fundamentally, the 26S proteasome is known to control many biochemical processes by programmed degradation of regulatory protein targets [40]. Upregulation of these genes may interfere with ubiquitin-dependent reactions in plants [41]. It has been reported that, the 26S proteasome degrades the virus-encoded movement protein that facilitates the viral cell-to-cell spread [42]. Therefore, the upregulation of the 26S proteasome genes may be a resistance response to PPV infection.

On the other hand, in *N. benthamiana *plants infected with ToRSV, about 72% (33/46) of the proteasome genes have been shown to be upregulated [13]. A few archetype proteasome proteins such as subtilisin-like protease, aspartyl protease, ubiquitin conjugating enzyme, F-box protein and 26S proteasome regulatory subunits induced by PPV in *Arabidopsis *leaves are upregulated by ToRSV in *N. benthamiana *plants [13]. Upregulation of such protein fate and ubiquitin genes has also been observed in other studies during viral infection [10,16,43,44]. Study by Marathe *et al*. [10] has demonstrated that the induction of F-box proteins may mediate the degradation components of the CMV-Y resistance pathway. Though the above mentioned protease genes induced by PPV infection are known to function in the protein degradation, plant defence responses and hypersensitive reactions [45-47], their exact roles in PPV infection are to be elucidated.

### Association of the repression of development/storage proteins, protein synthesis and cell wall-related genes in *Arabidopsis *leaf tissues with PPV infection and symptom development

Many virus-induced symptoms have been linked to alterations in plant hormone synthesis [4,48,49]. Of the 96 development/storage genes identified in this study, 10 auxin, 5 ethylene and 4 gibberellin-related genes were repressed in PPV-infected leaves [see Additional file 2]. Recent studies [48,49] on TMV infection in *Arabidopsis *have shown that during the process of infection the TMV replicase protein interacts with a subset of Aux/IAA proteins, resulting in the activation of auxin responsive transcription factors (ARF). Such an alteration in the transcription levels of auxin-responsive genes and the disruption of host normal physiological process have shown to be associated with disease symptoms. In this study, PPV infection suppressed the expression of two such genes encoding auxin IAA proteins (At5g57420 and At1g52830). With respect to the downregulation of ethylene responsive genes, though there is no direct evidence suggesting interactions between virus and ethylene signalling components, specific viral proteins have been suggested to alter the ethylene signalling pathway [4]. For instance, the P6 protein of *Cauliflower mosaic virus *(CaMV) is associated with the disruption of the ethylene response pathway and this may play a key role in the induction of symptoms such as mild vein chlorosis to severe chlorosis and stunting [50]. In the case of downregulation of gibberellic acid (GA), a direct interaction between the *Rice dwarf virus *(RDV) P2 protein and the rice ent-kaurene oxidase, a key enzyme in the synthesis of GA in plants was found to be responsible for the significant reduction of GA1 in RDV-infected plants, and in the appearance of dwarf phenotype [51]. Collectively these examples demonstrate that the suppression of hormone-related genes by viruses induces symptoms through altering the host hormone and developmental signalling pathway. It would be interesting to know whether any of the observed downregulated hormone-related genes is associated with the reduced growth and delayed development of PPV-infected *Arabidopsis*.

Another significantly over-represented subset of genes suppressed in PPV-infected tissues includes genes coding for the protein synthesis and translation machinery [see Additional file 2]. This functional category contains 33 ribosomal subunits: 21 large, 11 small and 1 plastid specific ribosomal proteins. In the *Arabidopsis *genome, there are ~227 ribosomal genes [13,52]. About 15% of these ribosomal genes were printed on the array. Based on the analysis, we observed that approximately 81% (27/33) of the small and large ribosomal subunit genes were significantly repressed in PPV-infected leaves.

However, an opposite trend was observed in PPV-infected *N. benthamiana *plants [13], where 80% (105/131) of potato ribosomal genes were upregulated. Comparison of these potato upregulated ribosomal genes in *Arabidopsis *through BLAST (E-value < -10) sequence similarity searches identified 55 nuclear ribosomal subunits [13]. Comparative analysis of 27 *Arabidopsis *nuclear ribosomal subunits that were downregulated by PPV in our study with those in Dardick's study (using Table S5 from Dardick's study for comparison) showed 8 (7 large and 1 small) *Arabidopsis *ribosomal subunits with an opposing trend. Since plant viruses lack protein synthesis ability, they rely on the host cell translation machinery to produce viral proteins. Thus, PPV might benefit from the upregulation of ribosomal genes in *N. benthamiana *which showed more susceptibility to PPV and displayed much more severe symptoms than *Arabidopsis*. However, at present there is no evidence to discern whether the observed downregulation of ribosomal genes is due to the stronger basal resistance of *Arabidopsis *to PPV infection or due to the natural intricacy of the different host responses to PPV that are diverse depending on the tissue type, time post infection, temperature, host and virus strain, or simply due to an artifact of hybridizations, given that two different platforms were used in the studies.

Cell wall-related genes are major determinants of cell morphogenesis in plants, encoding several hundreds of different structural proteins and cell wall-related enzymes [53,54]. In this study, 75 cell wall-related genes encoding protein for structure (23 genes), reassembly (34 genes), expansins (9 genes) and matrix polymers (9 genes) were significantly downregulated in PPV-infected leaves [see Additional file 2]. This is consistent with results observed in *Arabidopsis *plants infected by TuMV [16] and in rice plants infected with RDV [15]. In these studies, repression of cell wall related genes is correlated with symptom development. Of these downregulated genes, extensin is a cell wall-localized hydroxyproline-rich glycoprotein that usually forms a cross-linked network with pectin to create a highly impassable barrier against pathogens [55]. Downregulation of extensin during PPV infection suggests a decreased extensin deposition [56]. Conceivably, PPV exploits the plasmodesmata to move from initially infected cells to the neighbouring healthy cells. It is not likely that PPV cell-to-cell movement is related to the deposition of extensin. Nonetheless, the molecular mechanisms and the biological connotation underlying the downregulation of cell wall-related genes in the infected leaf remain to be understood.

In addition to the above described genes, many genes in the category of metal ion binding or metal binding were also downregulated. It is not clear if the downregulation of these genes can lead to physiological changes that are associated with PPV infection.

### Identification of genes differentially regulated by PPV in infected *Arabidopsis *protoplasts

Resistance induced through signal transduction pathways in *Arabidopsis *may take place immediately after PPV infection. The *Arabidopsis *leaves infected with PPV at 17 dpi contained a mixture of uninfected, infected, and post-infected cells. Thus, the spatial and temporal information on gene expression was lost. Sampling at earlier time points such as 1 or 2 dpi can reduce the percentage of post-infected cells but will largely increase the percentage of non-infected cells. So, protoplasts have been suggested to overcome this barrier [17]. There are several advantages in using *Arabidopsis *protoplasts to study plant-virus interactions. First, in contrary to unpredictable infections on the leaf, synchronized protoplast cells allows for a more precise determination of gene expression. Second, a much higher proportion of the cells are infected than that in infected leaf tissues [57]. For example, we have shown that in *Arabidopsis *protoplasts transfected with plasmid pPPV-SK68, a PPV infectious clone, the transfection efficiency is approximately 35% [58]. Hence, the protoplast system makes it possible to study molecular events as early as just a few hours post infection. Like other experimental systems, the protoplast system also has some limitations. Protoplasts lack the cell wall so the physiological integrity of the plant organ is disrupted. During isolation, transfection and culture of protoplasts, gene expression changes induced by harsh treatments such as osmotic stress, enzymatic digestion and centrifugation may mask those induced by virus infection [17]. Physiological milieu that a virus encounters in protoplast cells might be quite different from that of an intact leaf cell. Sample bias may result from the minute amounts of RNA isolated from the protoplasts and from the subsequent amplification step. However, improved technologies and experimental designs may be used to minimize the negative impact of the system [59-62]. Recently, we reported a protocol describing the isolation and transfection of *Arabidopsis *protoplasts for studying PPV-host interactions [58]. In order to identify early time points for profiling global gene expression changes in infected protoplast cells, a time course experiment was performed. *Arabidopsis *protoplasts were transfected with a PPV infectious cDNA clone, pPPV-SK68 derived from an M strain and with a non-infectious deletion mutant, pPPV-SK68Δ as control, respectively [58]. In the mutant clone, the deletion abolishes the translation of viral proteins CI, 6K2 and NIa/VPg required for PPV replication. The quantitative results showed that the viral RNA in the protoplasts was detectable at 3 hpt, accumulated at 6 hpt and peaked at 12 hpt (Figure 1C), followed by a decline at 24 hpt [58]. The time course progression of PPV infection in *Arabidopsis *protoplasts is similar to that of *Cymbidium mosaic virus *(CyMV) in orchid protoplasts [63]. Thus, three time points, 3, 6 and 12 hpt were selected for this study.

**Figure 3:**
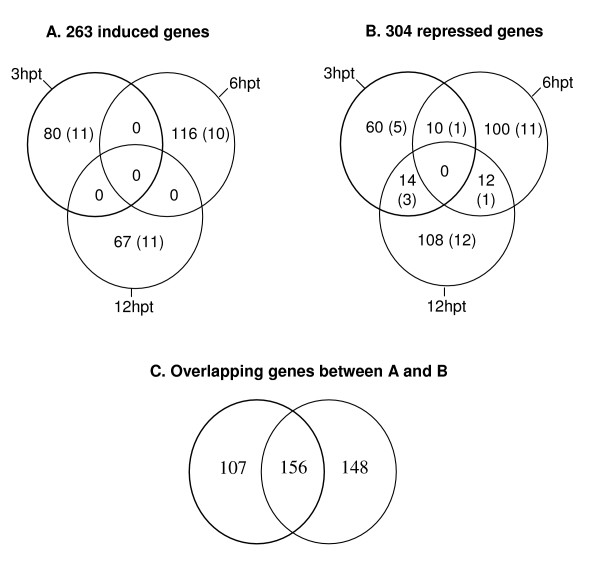
**Venn diagrams depicting the distribution of induced (≥ 2.5 fold) and repressed (≤ -2.5 fold) genes in PPV-infected protoplasts at three different time points**. A statistical cut-off with a FDR at 5% that corresponds to *Q *≤ 0.05, after Benjamini and Hochberg's correction was used to determine genes significantly differentially regulated in protoplasts transfected with a PPV infectious cDNA clone, pPPV-SK68 at 3, 6 and 12 hours post transfection (hpt). The number of genes in the non-overlapping sector represents unique significant genes at each time point, while the overlapping sectors represent genes that are in common at time points indicated. The number in parentheses in the Venn diagram circles corresponds to the genes induced or repressed in the PPV infected leaves.

Gene expression in PPV-infected *Arabidopsis *protoplasts was analyzed by one-way ANOVA to identify genes differentially expressed at each time point. The degree of biological variability and reproducibility of the microarray data was assessed by comparing the mean values of the normalized signal intensity from the protoplasts transfected with a non-infectious mutant, pPPV-SK68Δ with all three independent biological replicates at each time point. The analysis resulted with a high correlation coefficient of 0.90 – 0.95, indicating low technical and biological variability between the samples used in the hybridizations. Using FDR at 5%, we identified ~8.4% (1,907 out of 22,810) of non-redundant genes that were significantly (*Q *≤ 0.05) differentially regulated in protoplasts transfected with the PPV infectious clone, pPPV-SK68 at three different time points relative to the control transfected with the non-infectious deletion mutant, pPPV-SK68Δ. These genes were further filtered using a 2.5-fold increase or decrease in signal intensity, resulting in 263 nr genes that were induced (≥ 2.5-fold), and 304 genes that were repressed (≤ -2.5-fold), as illustrated in the Venn diagrams (Figure 3A and 3B). A comparison analysis between 263 induced and 304 suppressed genes identified 107 genes induced at one time point and were not repressed at other time points, 148 genes repressed at one time point and were not induced at other time points, and 156 genes induced and repressed at different time points (Figure 3C) [see Additional file 3]. The analysis also revealed that the number of repressed genes was greater than that of the induced genes at all three time points analyzed. The number of genes that were commonly repressed among the time points was 14 between 3 and 12 hpt, 10 between 3 and 6 hpt, and 12 between 6 and 12 hpt. We further examined each of these 263 induced and 304 repressed genes to see if they were also consistently induced or repressed at all three different time points of infection. Fifteen genes were found to be constantly repressed and 4 were consistently induced in the PPV transfected protoplasts, suggesting that the proportion of genes repressed at all three time points were greater than that of the genes induced.

**Figure 4:**
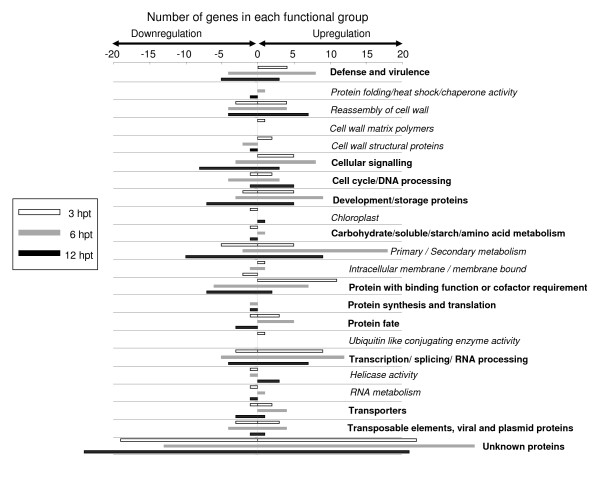
**Functional distribution of *Arabidopsis *genes significantly induced and repressed in PPV-infected protoplasts**. The genes were grouped following the method of the MIPS *Arabidopsis *classification scheme. Genes whose function has not been determined were grouped under "unknown function". Number of genes identified in each functional group is indicated on the x-axis. Genes that belong to major functional categories are highlighted in bold and the subcategories within a major functional category are highlighted in italics.

The genes induced or repressed in PPV-infected protoplasts were annotated, assigned to a predicted function and classified into 12 different major functional categories using the *Arabidopsis *MIPS functional classification scheme (Figure 4). Fisher's exact test [23,24] was used to determine whether the representation of the number of genes in each functional category was significant (*P *≤ 0.001) or by random chance. Of the upregulated genes, transcripts involved in primary and secondary metabolism (*P *< 6.2E-06), cellular signalling (*P *< 4.4E-11), defence response (*P *< 2.7E-06), transcription factors (*P *< 1.1E-11) and transporters (*P *< 1E-12) were over-represented, while in the case of downregulation, genes involved in transcription factors (*P *< 5E-09), primary and secondary metabolism (*P *< 6.4E-05), kinases or cellular signalling (*P *< 1.3E-05), and defence responses (*P *< 5.6E-05) were over-represented. A complete list of induced or repressed genes in PPV-infected protoplasts in different functional groups is provided [see Additional file 3].

As evident [see Additional file 4; Additional file 5; Additional file 6], comparison of the differentially expressed genes between PPV-infected *Arabidopsis *protoplasts (263 up- and 304 downregulated genes) and PPV-infected leaf tissues (2,013 induced genes and 1,457 suppressed genes) resulted in only 32 and 33 common genes that were significantly induced (≥ 2.5-fold) and repressed (≤ -2.5-fold), respectively. A few vital genes such as genes encoding heavy metal protein, disease resistance protein, FtsH protease, cytochrome P450 and β-1, 3-glucanase were significantly differentially regulated by PPV in the infected leaves and transfected protoplasts. The smaller percentage of significantly differentially regulated genes observed in the PPV-infected protoplasts than in the PPV-infected leaf suggests a remarkable gene expression differences between the two systems. However, as discussed above, the differences in the alteration of gene expression changes in the protoplasts induced by PPV infection could have been masked by harsh treatments during protoplast isolation, transfection and culture or might result from the two different PPV strains used. Though the PPV-D and -M strains used in this study share more than 95% sequence identity [64], these two strains may induce different symptoms on the same host [20,65].

**Figure 5:**
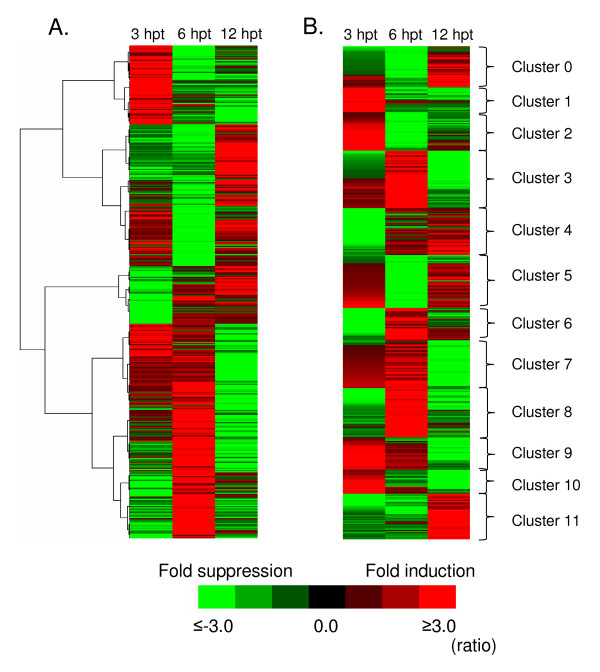
**Clustering analysis of differentially regulatedgenes in PPV-infected *Arabidopsis *protoplasts**. (A) Hierarchical clustering and changes in gene expression of 411 significantly (*Q *≤ 0.05) differentially regulated genes in PPV-infected *Arabidopsis *protoplasts at 3, 6 and 12 hours post transfection (hpt). Gene tree map generated from 411 significantly differentially regulated *Arabidopsis *genes are derived from 263 upregulated and 304 downregulated genes. (B) Using k-mean clustering, the gene expression profiles were grouped into twelve major cluster groups. The expression pattern of a gene in a cluster group is indicated in parentheses. Expression levels are color coded with red indicating upregulation by PPV infection; green indicating downregulation by PPV infection; and black indicating no change in expression. The intensity of color represents the degree of gene expression levels. List of genes induced or repressed at each time point in different cluster groups along with their expression levels and putative functions is provided in [Additional file 8].

To visualize gene expression profiling at all three time points, a hierarchical clustering analysis was carried out on the 263 induced and 304 repressed genes (Figure 5A). A gene tree heat map was built using Pearson correlation distance metric with an average linkage algorithm. To understand further the temporal relationship of the host gene expression in PPV-infected protoplasts at each time point, twelve distinct groups of expression pattern clusters were generated on the significant differential gene expression datasets using K-means clustering with Pearson correlation distance metric (Figure 5B) [see Additional file 7]. A complete list of genes induced or repressed at each time point in different cluster groups along with their expression levels and putative functions is provided [see Additional file 8]. The clustering analysis also revealed greater changes in gene expression (236 genes) at 6 hpt than at 3 hpt (163 genes) and at 12 hpt (201 genes) in PPV-infected *Arabidopsis *protoplasts. These observations suggest that early gene expressions in response to PPV infection are active and dynamic.

### Early upregulated genes related to disease resistance or defence responses in PPV-infected *Arabidopsis *protoplasts

PPV infection in protoplasts induced the expression of 263 genes. In contrast to about 11% of the suppressed genes that were common at different time points, there were no induced genes shared at different time points, suggesting induction of host gene expression was much more transient than suppression. Genes related to defence, cellular signalling, primary and secondary metabolism, transcription and transporters were upregulated and over-represented. Within these five categories, ~52% (48/93) of genes were predominantly upregulated (≥ 2.5-fold) at 6 hpt [see Additional file 3]. Examples of the upregulated genes in the above functional categories include MADS box protein, zinc finger family protein, WRKY family transcription factor, avirulence (Avr)-induced protein, hydrophobic protein, disease resistance TIR-NBS-LRR (toll-interleukin-1-receptor/nucleotide-binding site/leucine-rich repeat), protein kinases and phosphatases.

Another interesting gene upregulated by PPV encodes a disease resistance protein, TIR-NBS-LRR. This gene was upregulated with 6.39-fold in PPV-infected protoplasts at 6 hpt and 2.12-fold at 12 hpt, and 10.84-fold in PPV-infected leaves [see Additional file 4]. In the *Arabidopsis *(Col-0 accession) genome, there are 149 NBS-LRR-like genes [66,67]. The resistance gene *NBS-LRR *mostly confers resistance to viral diseases but not necessarily associates with cell death or tissue necrosis [66]. Genes *Sw5*, *N *and *Rx *are homologous to the *NBS-LRR *gene. *Rx *resistance to *Potato virus X *(PVX; Potexvirus) is the most classical example, which shows an extreme resistance that inhibits virus replication without hypersensitive cell death [68]. The *Sw5 *gene confers resistance to several Tospoviruses [69], and *N *and *Rx *are effective against many natural variants of TMV and PVX [70,71]. Since the specificity determinants subsist in LRR domains [66], it would be interesting to find out if the NBS-LRR gene detected in the PPV-infected leaf plays a role in resistance responses to PPV infection.

The comparison of induced genes at different time points in PPV-infected protoplasts revealed that genes upregulated at 3 hpt might regulate their target genes at 12 hpt. For instance, two genes coding for cnd41 (At5g24820), a plastid specific DNA binding factor, and calmodulin (At3g15050, At3g16490), a signalling binding protein were induced at 3 hpt and their target genes encoding metabolic enzymes such as serine carboxypeptidase (At1g11080) were upregulated at 12 hpt [57,58]. Similarly, transcription factors such as zinc finger proteins (At5g54020; At3g45480; At4g12140) was upregulated at 3 hpt and the genes encoding enzymes such as GDSL-motif lipase (At3g43550) and glycosyl hydrolases (At3g26140, At4g39010) were upregulated at 12 hpt [72]. These results suggest that virus infection may induce expression of specific upstream regulator genes first which then activate the expression of downstream target genes.

### Inhibition of host mRNA accumulation in PPV-infected protoplasts

Based on the expression analysis (Figure 3), the number of repressed genes in the transfected protoplasts was greater than that of the genes induced at all time points, suggesting that at early infection stages, suppression is more active than induction of host gene expression. Moreover, about 11% (33 out of 304) of the suppressed genes overlapped at different time points, indicating a relatively stable suppression of genes. Consistent with this, in pea cotyledons infected with *Pea seed borne mosaic virus *(PSbMV), suppression of diverse host genes including seed storage proteins (e.g., vicilin and convicilin), and starch synthase occurs only in a zone of six to eight cells immediately behind the infection front, which coincided with the onset of viral replication [73]. In the present study, a gene coding for the seed storage protein cupin family (At2g18540) that shares sequence homology to the pea vicilin and convicilin was significantly repressed by PPV infection at 12 hpt in *Arabidopsis *protoplasts. Suppression of such host gene expression or "host shutoff" has been suggested to be due to stress responses, induced defence states or a mechanism employed by viruses to outcompete against host mRNAs for host resources for their translation and replication [16,17,43,74]. Study by Rottier [75] has demonstrated that protein synthesis in protoplasts is blocked in cowpea protoplasts transfected with *Cowpea mosaic virus*. Despite this inhibition, viral mRNAs are preferentially translated. Since both viruses and hosts share the same translational apparatus, viruses must have a mechanism to avoid this inhibition. It is not clear whether the disappearance of host mRNAs is the consequence of mRNA destabilization triggered by stress response, or host transcription inhibition by the virus, or targeted degradation through a virus-induced ribonuclease activity.

### Identification of a common set of genes in general stress and defence-related groups differentially regulated by infections of PPV and other positive sense RNA viruses

We performed a cross sequence comparison analysis by blasting individual sequence of all the genes significantly differentially regulated by PPV in infected *Arabidopsis *leaves against 2,156 uniEST sequences derived from PPV-infected *P. persica *leaves [64]. High-scoring pair (HSP; ≥ 100 score) and E-value (E-value ≤ 10–20) were used to identify orthologs. A total of 170 induced [see Additional File 9] and 18 repressed *Arabidopsis *genes showed high sequence similarity to *P. persica *uniESTs. Of these 170 induced genes, approximately ~39% (67/170) and ~16% (28/170) of the genes were involved in metabolism and defence, respectively. The defence-related genes include those coding for pathogenesis related (PR)-proteins, heat shock and germin-like proteins (Ger2) [see Additional file 9]. An increased expression of PR proteins requires salicylic acid (SA) signifying that compatible host-virus interactions induce defence-like responses through a SA dependent pathway [76]. More recently, it has been shown that PPV infection in the apoplastic space of *P. persica *leaves induces a thaumatin-like protein (PR-5) [77]. Induction of such protein is thought to be mediated by an increase in the level of peroxidase accumulation [77]. A similar trend was also observed in PPV infected *Arabidopsis *leaves where genes encoding PR-5 and peroxidase were significantly induced and their counterparts were also found in the uniEST sequences derived from PPV-infected *P. persica*.

To determine whether the diverse RNA viruses have the ability to elicit common gene expression changes, we cross-compared the genes that were significantly differentially regulated by PPV infection in this study [Additional file 1] [Additional file 2] to those *Arabidopsis *genes that were differentially regulated by infections of other positive sense RNA viruses including CMV [9,10]; TMV [7]; TuMV, and *Oil seed rape mosaic virus *(ORMV) [16], and to five diverse RNA viruses, i.e., TuMV, ORMV, *Turnip vein clearing virus *(TVCV), *Potato virus X *(PVX), and CMV [8]. Collectively, 416 common genes [see Additional file 10] were identified from this analysis. These genes were further classified into 6 different categories. Group A contains 202 genes induced by infections of PPV and other positive sense RNA viruses. About ~40% (80/202) are metabolic genes and ~8% (17/202) are defence-related genes. Group B includes 41 *Arabidopsis *genes that were induced during PPV infection but repressed by infections of other positive sense RNA viruses. About ~27% (11/41) are involved in metabolism. Group C comprises of 119 *Arabidopsis *genes repressed during PPV infection but induced by infections of other positive sense RNA viruses. For example, a defence-related gene encoding glycosyl transferase (At1g70090), which was repressed in PPV-infected leaves at 17 dpi was observed to be induced more than 1.5-fold in TMV-infected leaves [7] at 14 dpi. Group D consists of 7 *Arabidopsis *genes repressed by infections of PPV and other positive sense RNA viruses. Group E comprises 25 genes that were induced during PPV infection but either induced or suppressed by infections of other positive sense RNA viruses. Group F contains 22 *Arabidopsis *genes repressed during PPV infection but induced during infections of more than one positive sense RNA virus. Previous studies have revealed that in virus-infected *Arabidopsis *and tomato leaves as many as one-third of the genes commonly induced by viruses are known or predicted to be involved in plant defence- and stress-related genes [5,15]. In the current study, about 52% (217/416) of the functional proteins differentially regulated in response to infections of PPV and other positive sense RNA viruses are involved in defence, cellular signalling and metabolism. Twenty-nine cellular signalling genes involved in defence mechanisms including protein kinase (At3g08760, At4g32250), protein phosphatase 2C (At5g02760, At3g05640), pyruvate kinase (At2g36580), serine/threonine protein kinase (At3g17410) and calmodulin-binding family protein (At5g62390) [3,78,79] were induced by infections of PPV and other positive sense RNA viruses. Substantial induction of these defence, cellular signalling and metabolism genes by PPV and other positive sense RNA viruses in infected leaf tissues suggests that there is a shared pathway modulating their expression.

### Validation of microarray data by semi-quantitative RT-PCR (sqRT-PCR) and Northern hybridizations

**Figure 6:**
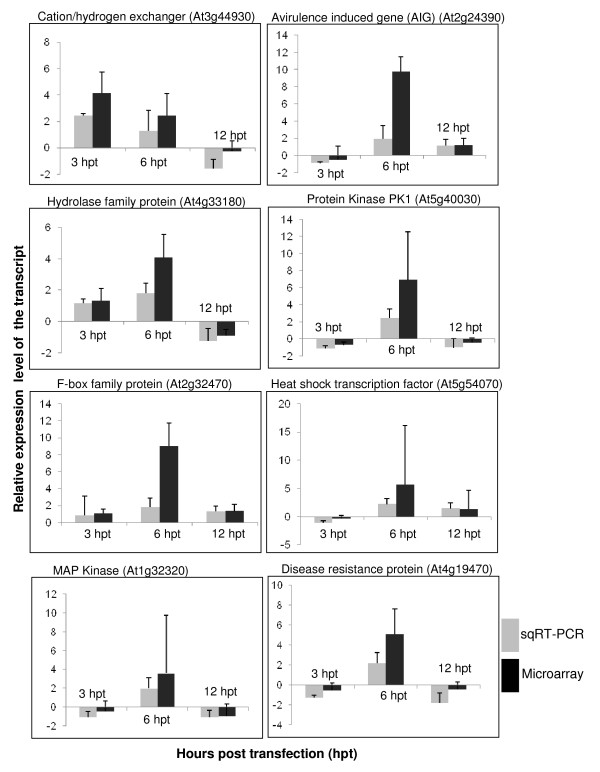
**Confirmation of relative expression levels of the transcripts selected from the microarray analysis with semi quantitative RT-PCR (sqRT-PCR)**. Expression changes of 8 selected genes were determined by sqRT-PCR and microarray. The signal intensity of each transcript was normalized using At3g18780 (Actin 2). The *Arabidopsis *Genome Initiative (AGI) locus identifier of each gene is provided. The y-axis indicates the normalized expression level of the transcript. The x-axis represents hours post transfection. Expression ratios are the average of three independent hybridizations ± standard deviation (SD). hpt, hours post transfection; dpi, days post inoculation. The error bars represent the standard deviation for the signals from each of the three independent hybridizations.

**Figure 7:**
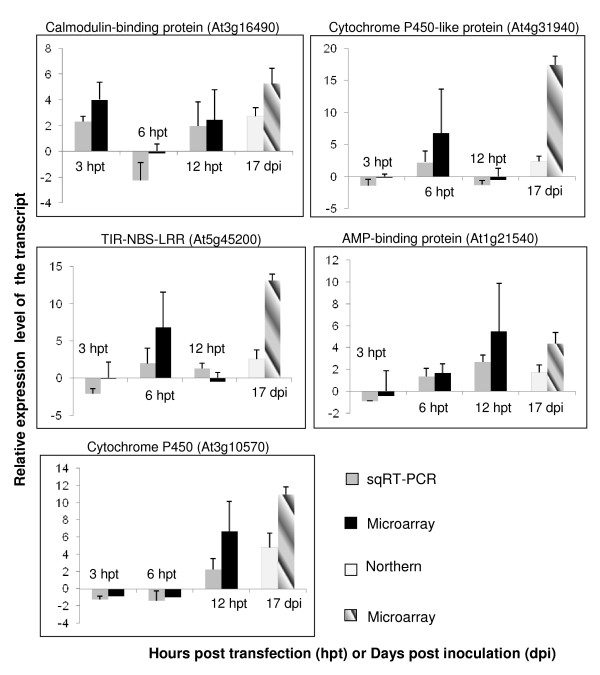
**Confirmation of relative expression levels of the transcripts selected from the microarray analysis with semi quantitative RT-PCR (sqRT-PCR) or Northern blot hybridizations**. Expression changes of 5 selected genes were determined by sqRT-PCR, microarray and Northern blot. The signal intensity of each transcript was normalized using At3g18780 (Actin 2). The *Arabidopsis *Genome Initiative (AGI) locus identifier of each gene is provided. The y-axis indicates the normalized expression level of the transcript. The x-axis represents hours post transfection or days post inoculation. Expression ratios are the average of three independent hybridizations ± standard deviation (SD). hpt, hours post transfection; dpi, days post inoculation. The error bars represent the standard deviation for the signals from each of the three independent hybridizations.

Since most changes in gene expression occurred at 6 hpt, 9 transcripts significantly upregulated and 1 transcript significantly downregulated at 6 hpt were selected to validate the microarray data using sqRT-PCR. We also selected 3 other transcripts that were significantly upregulated at 3 or 12 hpt for validating the microarray data through sqRT-PCR. We further confirmed the microarray data of 5 *Arabidopsis *genes that were differentially regulated in both PPV-infected protoplasts and leaves using sqRT-PCR or Northern hybridizations. Overall, the expression patterns of all the 13 genes analyzed by sqRT-PCR were consistent with those by microarray hybridizations (Figures 6 and 7) [see Additional file 11]. However, the relative levels of gene expression obtained from sqRT-PCR or Northern hybridizations were lower than those from microarray hybridizations. This inconsistency could have been attributed to several factors: the differences in the efficiencies of reverse transcriptase, the low copy of an mRNA transcript and the different priming methods or the fundamental differences in data normalization procedures between microarray and Northern hybridizations or sqRT-PCR. For example, in this study, the former was achieved through the global normalization, while the later used the expression of the housekeeping gene *Actin *to normalize the sqRT-PCR or Northern blots. So, application of normalization criteria might have an effect in the correlation of fold changes between these two methods [80,81]. Though the housekeeping gene *Actin *was considered to be constitutively expressed in tissues, due to the nature of the processing of hybridizing samples to Northern blots, one could reasonably assume a possible inconsistency resulting from these two methods. Certainly, other sensitive techniques such as quantitative RT-PCR (qRT-PCR) and nuclear run-on assays can be used further to validate the results generated from these expression studies.

## Conclusion

Microarray analyses conducted in this study allowed us to gain insights into global gene expression changes in *Arabidopsis *associated with PPV infection, symptom development and defence responses. At 17 dpi, PPV displayed mild growth retardation, slightly delayed flowering and bolting. PPV infection was associated with the downregulation of genes related to development/storage proteins, protein synthesis and translation and cell wall-related genes and the upregulation of genes involved in soluble sugars, starch, amino acid, intracellular membrane/membrane-bound organelles, chloroplast and protein fate. Transcriptional profiling of PPV-infected protoplasts revealed that at early infection stages, downregulation of host gene expression was more prominent than upregulation. Concomitant with this downregulation, several defence and cellular signalling genes were induced during PPV infection. These genes may be involved in signal transduction pathways to induce resistance to combat the virus. Further targeted functional studies of these early defence-related genes will help unravel their biological function during the course of PPV infection.

## Methods

### Plant growth and virus inoculation

*Arabidopsis thaliana *accession Col-0 was grown in a growth chamber at 80% RH with a day/night regime of 16 h light at 22°C followed by 8 h dark at 18°C. Prior to the preparation of viral inoculum, leaf tissues collected from *Prunus glandulosa *infected with a Canadian PPV-D isolate were tested positive by ELISA and by RT-PCR. The inoculum was then prepared by homogenizing approximately 1 g of *P. glandulosa *leaf tissues infected with PPV-D in 10 ml inoculation buffer (PO_4 _buffer, pH 7.4 containing 2% PVP-40 (w/v) and 0.2% diethyl dithiocarbonic acid). Viral inoculum was mechanically rub-inoculated onto *Arabidopsis *leaves at 5–6 leaf stage. Control plants were mock inoculated with extracts from healthy *P. glandulosa *leaf tissues ground in inoculation buffer. All the mock- and PPV-infected *Arabidopsis *plants used in the microarray experiment were randomized and grown in different locations within a greenhouse under the same growth conditions. Fifty fully expanded rosette leaves above the inoculated leaves were harvested from 25 infected or mock inoculated control plants at 17 dpi. The leaves were immediately frozen with liquid nitrogen and stored at -80°C prior to RNA extraction. Six independent hybridizations were performed using total RNA isolated from three independent biological replicates of the virus-infected or mock-inoculated control samples.

### PPV infectious cDNA clone and its deletion mutant

The PPV infectious cDNA clone pPPV-SK68 derived from an M strain was a kind gift from Drs. Laszlo Palkovics and Ervin Balazs, Agricultural Biotechnology Center, Hungary. A control plasmid, pPPV-SK68Δ, was generated by deleting 1489 nt Sma I cDNA fragment from nt 4272 to 5760 (inclusive). This deletion resulted in the frame-shift of the portion of PPV genome after nt 5760 [58]. The deletion region abolishes the translation of the viral protein domains CI-6k2-NIa/VPg-NIa/Pro-NIB-CP which is required for viral replication. Thus, clone pPPV-SK68Δ is non-infectious.

### Protoplast isolation and transfection

Protoplasts were isolated using fully expanded leaves of 3-week-old *Arabidopsis *accession Col-0 and transfected with the PPV infectious cDNA clone or the mutated PPV cDNA clone following the protocol we have developed recently [54]. Transfected protoplasts were incubated at room temperature for 5 min, and then transferred to 25°C and incubated in the dark for 3, 6 and 12 h, respectively. At selected time points, transfected protoplast cells were harvested, immediately frozen in liquid nitrogen and stored at -80°C prior to RNA extractions. Gene expression differences were averaged out by pooling six RNA samples isolated independently from protoplasts transfected with pPPV-SK68 and pPPV-SK68Δ at each time point after transfection. For time course comparison, three independent biological replicates were maintained. Thus, for three different time points, 18 gene chips in total were used to hybridize with RNA isolated from protoplasts transfected with pPPV-SK68 and pPPV-SK68Δ.

### RNA extraction, purification and virus detection

Total RNA from virus-infected and mock-inoculated leaves of *Arabidopsis *plants and from protoplasts transfected with pPPV-SK68 and pPPV-SK68Δ were isolated using Trizol reagent (Invitrogen) following the manufacturer's suggestion. Total RNA was purified using RNeasy Mini columns (Qiagen) after DNase I treatment (Invitrogen). Integrity of total RNA was assessed using Bioanalyzer 2100 (Agilent Technologies, Boblingen, Germany). Primers OL-PPVDF3 (5'TCAATGGAATGTGGGTGATGA 3') and OL-PPVDR6 (5' GATTAGACTCTCACCCAGGTA 3'); and PPVMF18 (5' GGAAATTGAGAGATACCT 3') and PPVMR16 (5' TTTGTCTAAAAGTGGGTTT 3') were used to detect PPV in the virus-infected or mock-inoculated control samples and in pPPV-SK68- and pPPV-SK68Δ-transfected protoplasts, respectively.

### Target preparation and hybridization

Standard RNA processing and hybridization protocols were followed as recommended by the Affymetrix manual (Santa Clara, CA, USA). A double-amplification approach (Affymetrix) was used to generate cRNA for hybridization. Total RNA isolated from virus-infected or mock-inoculated leaves were subjected to double-amplification. Labelling, hybridization and detection of hybridization signals were performed at the McGill University and Genome Quebec Innovation Centre (please see Availability & requirements for more information).

### Data analysis

Hybridization signals obtained from the scanned array images were quantified using MAS5.0 expression analysis algorithm, implemented in the Affymetrix GCOS (GeneChip^® ^Operating) software version 1.2. Raw MAS5.0 data files obtained from scanned array images were imported into GeneSpring microarray analysis software version 7.3.1 (Silicon Genetics, Redwood City, CA, USA). Systemic variations across the chips were normalized using per gene and per chip normalization methods available in the GeneSpring software. Mean normalized value of the signal intensity for each gene in three biological replicate experiments was adopted as the expression value of the gene. The significant changes in gene expression during PPV infection in infected leaves and in protoplasts were determined using *P *values (*P *≤ 0.05) derived from one-way ANOVA and the significant changes in the values of the identified genes were further adjusted using multiple testing correction of the Benjamini and Hochberg false discovery rate procedure [21] which applied a stringent cut off of 0.05 that corresponds to *Q *≤ 0.05 [22]. This multiple testing correction procedure is used to correct for the occurrence of false positives and to maximize the likelihood of finding significant gene sets. In order to identify genes that show significant changes of transcript levels during virus infection, we further searched for genes that were significantly up- (≥ 2.5-fold) or downregulated (≤ -2.5-fold) relative to the control. For clustering analysis, gene expression profiles from the protoplasts transfected with the PPV infectious clone at each time point were clustered based on their similarity in expression using hierarchical average linkage algorithm and Pearson correlation distance metric implemented in the Cluster 3.0. The clustering results were visualized by a gene tree heat map using the TreeView program [82]. K-means clustering was employed to cluster the gene expression data from the PPV transfected protoplasts into several distinct expression profiles using Pearson correlation distance metric. The microarray data from this work was submitted to the NCBI database with Gene Expression Omnibus (GEO) accession number [GSE11217].

### Functional categorization of genes

Each gene was assigned with a putative function by blasting the AGI probe identifier sequences against the *Arabidopsis *database deposited at TAIR (the *Arabidopsis *Information Resources) using the BLASTX program. Functional classification was performed by assigning the function of each gene based on protein sequence similarity using MIPS (Munich Information Centre for Protein Sequences; please see Availability & requirements for more information) *Arabidopsis *functional classification schema. Genes without a category name or with unclear classification were labeled as "unknowns". Over-or under-representation of genes involved in each functional group was evaluated using Fisher's exact test function [23,24].

### Determination of *P. persica *orthologs to *Arabidopsis *genes induced by PPV

The PPV-infected *P. persica *leaf (PPL) EST library [64], consisting of 2156 uniEST sequences extracted from NCBI dbESTs [GenBank: DN552797, DN556565] were formatted so that they were searchable using the tBLASTx algorithm. Sequences of 2013 PPV induced and 1457 PPV repressed *Arabidopsis *transcripts in infected leaf tissues respectively were aligned to this collection to search for the presence of *P. persica *orthologs using the tBLASTx algorithm with default settings [83]. The BLAST output for each transcript was then parsed for high-scoring pair (HSP) and the associated E-value. Any hits with HSP 100 and E-value 10^-20 ^were taken as an indicative of significant similarity [84].

### Cross comparison of genes differentially regulated by PPV infection against the *Arabidopsis *genes differentially regulated by infections of other positive sense RNA viruses

The *Arabidopsis *genes differentially regulated in PPV-infected leaf tissues were cross compared by searching against the *Arabidopsis *gene set that were differentially expressed in response to the infection by other positive sense RNA viruses [7-10,16].

### Northern hybridizations and semi quantitative RT-PCR (sqRT-PCR)

Northern hybridizations and sqRT-PCR were carried out to verify the microarray data. Probes for sqRT-PCR and Northern hybridizations were generated by PCR amplification of *Arabidopsis *cDNA with gene specific primers (Table 2). *Arabidopsis *Actin 2 (ACT2; At3g18780) was used as an internal control with the following primer sets; 5' GCCATCCAAGC TGTTCTCTC 3' and 5' GAACCACCGATCCAGACACT 3' to normalize small differences in template amounts. For Northern hybridization, 10 μg of total RNA was denatured with glyoxal, electrophoretically separated on 1.5% agarose gels, blotted onto a nylon membrane and UV cross-linked (1200 μJ/cm^2^). For sqRT-PCR, amplicons of RT-PCR were electrophoretically separated on a 1% agarose gel, transferred to a nylon membrane by capillary blotting and cross-linked as above. Hybridizations for Northern blots and sqRT-PCR were performed with at least three independent biological replicates. The probes were radiolabeled with [α-^32^P] dCTP by Ready-To-GO DNA labeling kit following the manufacturer's instructions (Amersham Biosciences). A Phosphor Imager (Imaging Screen K, Personal Molecular Imager^® ^FX, Bio-Rad) and Quality One Quantitation software v. 4.2 (Bio-Rad) were used for visualization and quantification of radioactive signals.

**Table 2:** Primers used in semi quantitative RT-PCR^a ^and in Northern hybridzations^b^

**Probe set IDs **^c^	**AGI**^d^**locus**	**Description**	**Forward Primer (5'---3')**	**Reverse Primer (5'---3')**
**A. Genes induced only in PPV-infected *Arabidopsis *protoplasts**				
256487_at	At1g31540	Disease resistance protein, TIR-NBS-LRR class	GCCAGTAGAGCTCGAGGTAAAATG	AGGCTCTTCACTGTGTTCAATCTC
246338_s_at	At3g44930	Cation/hydrogen exchanger CHX10, putative	TAACCCGAGGATCCGTGTAAC	ATCTCTAGCCGCCAGCAAATC
265682_at	At2g24390	Avirulence induced gene (AIG) protein, related	CAATGCACAGTGTCTTCGTCTA	TTCGGTAAATTCAACTCTTCAGC
254582_at	At4g19470	Disease resistance protein, related	CAGAAACGAAGACGGTGATTAC	TCTTCCTTTATTCTTTTCCTGTTC
267056_at	At2g32470	F-box family protein, related	TGGGGTTTCACAGTCAATAATAGT	TTCGCATACCAATCCCACAAC
248188_at	At5g54070	Heat shock transcription factor	TGAGTCATTGAAGGAGGAACAGAG	GAGCCACCGTCAACAAGTAGG
253366_at	At4g33180	Hydrolase family protein	TCGCAGACGCTTTCCATAGACT	GAAGAAGACAAGATCGGGAGAATA
260699_at	At1g32320	MAP kinase, putative	GACATAAAGCCTTCAAATCTCC	ATCTCCCAACAAAATCTCTAAACT
249440_at	At5g40030	Protein kinase PK1- like	CTTCAAGCCTCGGTTCCTCAA	GATCGGTTTCGGTATTTCTGGTG
**B. Genes induced in PPV-infected *Arabidopsis *protoplasts and leaf tissues**				
260921_at	At1g21540	AMP-binding protein, putative	TTAGGGACCCGAGAACC	GCAGAGACTTAGCCATTTGT
258962_at	At3g10570	Cytochrome P450, putative	TCGTGAGGTTGAGGAAAAAGATG	GATAAGCGCTCCACTCAAACTC
253502_at	At4g31940	Cytochrome P450-like protein	TGCACGGTCGCTGGTTACT	AGGCGTGGCTTTAGGAATG
248989_at	At5g45200	Disease resistance protein, TIR-NBS-LRR	ATTCTTGGTGGTTGGACTG	CACTTCTCTTTGGACTTTC
257229_at	At3g16490	Calmodulin-binding family protein	AATGAATGTGGCTGTATCC	CGTTTGCGGTTGTGGTTGT

^a^Genes shown in panels "A" and "B" were validated using semi quantitative RT-PCR.

^b^Genes shown in panel "B" were validated using Northern hybridizations.

^c^Probe set ID represents Affymeterix probe set number.

^d^AGI represents *Arabidopsis *Genome Initiative (AGI) locus identifier that corresponds to each gene represented on the array.

## Availability & requirements

The *Arabidopsis *Information Resource: 

McGill University and Genome Quebec Innovation Centre: 

Munich Information Centre for Protein Sequences: 

## Authors' contributions

MB carried out most of the experimental work including design of experiments, preparation and transfection of protoplasts, RNA isolation, analysis and interpretation of microarray data, and verification experiments. JSG participated in preparation and transfection of protoplasts, and isolation of RNA. T–SH prepared and inoculated *Arabidopsis *plants. AW conceived the work and interpreted the data. MB and AW wrote the paper. All authors have read and approved the manuscript.

## Supplementary Material

Additional file 1 Supplemental Table 1. Expression levels of genes induced in PPV-infected *Arabidopsis *leaf tissues 17 days post inoculation. Click here for file

Additional file 2 Supplemental Table 2. Expression levels of genes repressed in PPV-infected *Arabidopsis *leaf tissues 17 days post inoculation. Click here for file

Additional file 3 Supplemental Table 3. Expression levels of differentially regulated genes in PPV-infected *Arabidopsis *protoplasts. Click here for file

Additional file 4 Supplemental Table 4. Expression levels of common genes induced in PPV-infected *Arabidopsis *protoplasts and in infected leaf tissues. Click here for file

Additional file 5 Supplemental Table 5. Expression levels of common genes repressed in PPV-infected *Arabidopsis *protoplasts and in infected leaf tissues. Click here for file

Additional file 6 Supplemental Figure 1. Hierarchical clustering of common genes that were significantly differentially regulated by PPV in infected leaf tissues and in transfected protoplast cells. Expression levels are color-coded with red indicating upregulation by pPPV-SK68; green indicating downregulation by pPPV-SK68 and black indicating no change in expression. The intensity of color represents the degree of gene expression levels. The putative function of each gene is shown on the right side of the cluster. Click here for file

Additional file 7 Supplemental Figure 2. K-means profiling of gene expression using 411 *Arabidopsis *genes differentially regulated by PPV in transfected protoplasts at three different time points. The expression profiles were grouped into twelve distinct cluster groups. The AGI locus identifier of each gene differentially regulated by PPV in the transfected protoplasts at different time points in each cluster group is shown on the right side of the cluster. Values on the y-axis indicate the relative expression level of the gene, while the x-axis represents hours post transfection. Number of genes belonging to each cluster is shown in the cluster inset. Click here for file

Additional file 8 Supplemental Table 6. Gene expression profiles of *Arabidopsis *genes differentially regulated by PPV infectious clone in transfected protoplasts belonging to twelve distinct cluster groups. Click here for file

Additional file 9 Supplemental Table 7. Identification of *Prunus persica *orthologs to *Arabidopsis *genes induced by PPV infection in the leaf tissues at 17 days post inoculation. Click here for file

Additional file 10 Supplemental Table 8. Cross comparison of genes significantly differentially regulated by PPV (≥ 2.5- or ≤ -2.5-fold) in this study with the *Arabidopsis *genes regulated by other positive sense RNA viruses. Click here for file

Additional file 11 Supplemental Figure 3. Confirmation of microarray data by sqRT-PCR and Northern hybridizations. Panel A shows confirmation of microarray data using sqRT-PCR for genes induced in PPV-infected *Arabidopsis *protoplasts. Panel B shows confirmation of microarray data using sqRT-PCR (left panel) and Northern hybridizations (right panel) for *Arabidopsis *genes differentially regulated in PPV-infected protoplasts and in PPV-infected leaves. Probes for sqRT-PCR and Northern hybridizations were generated by PCR amplification of *Arabidopsis *cDNA using gene specific primers shown in Table 3. sqRT-PCR of the constitutively expressed Actin 2 gene (At3g18780) was used as a loading control. pPPV-SK68, a PPV infectious cDNA clone used to transfect protoplasts; pPPV-SK68Δ, a mutant non-infectious clone of pPPV-SK68 was used as a control; hpt, hours post transfection. Click here for file
