# Supplementary material for: Altered gene expression changes in Arabidopsis leaf tissues and protoplasts in response to Plum pox virus infection
Source: BMC Genomics. 2008 Jul 9;9:325. doi: 10.1186/1471-2164-9-325 (PMC2478689; doi:10.1186/1471-2164-9-325)
Supplement: Additional file 1 — Supplemental Table 1. Expression levels of genes induced in PPV-infected Arabidopsis leaf tissues 17 days post inoculation. [file 1471-2164-9-325-S1.pdf]

Supplemental Table 1. Expression levels of genes induced in PPV-infected *Arabidopsis* leaf tissues 17 days post inoculation

Probe set IDs[a]	AGI[b] locus	Annotation	*q*-value[c]	*p*-value[d]	Fold change[e]
A. Defence and virulence[f]					
260352_at	At1g69295	β-1,3-Glucanase-Related	0.0309	0.0386	5.65
259517_at	At1g20630	Catalase 1	0.0004	0.0050	2.93
253174_at	At4g35090	Catalase 2	0.0030	0.0126	3.56
263692_at	At1g26850	Dehydration-Responsive Family Protein	0.0083	0.0199	4.64
263774_at	At2g40280	Dehydration-Responsive Family Protein	0.0029	0.0123	2.54
255637_at	At4g00750	Dehydration-Responsive Family Protein	0.0075	0.0189	2.58
254972_at	At4g10440	Dehydration-Responsive Family Protein	0.0000	0.0010	3.74
246288_at	At1g31850	Dehydration-Responsive Protein, Putative	0.0001	0.0025	6.15
259771_at	At1g29470	Dehydration-Responsive Protein-Related	0.0038	0.0141	3.44
255638_at	At4g00740	Dehydration-Responsive Protein-Related	0.0062	0.0174	3.46
247316_at	At5g64030	Dehydration-Responsive Protein-Related	0.0077	0.0193	5.04
247095_at	At5g66400	Dehydrin (Rab18)	0.0274	0.0363	5.14
263753_at	At2g21490	Dehydrin Family Protein	0.0004	0.0048	6.45
249832_at	At5g23400	Disease Resistance Family Protein / Lrr Family Protein	0.0031	0.0129	3.18
245839_at	At1g58390	Disease Resistance Protein (CC-NBS-LRR Class), Putative	0.0014	0.0087	15.53
245838_at	At1g58410	Disease Resistance Protein (CC-NBS-LRR Class), Putative	0.0206	0.0315	3.41
264885_s_at	At1g61180	Disease Resistance Protein (CC-NBS-LRR Class), Putative	0.0051	0.0160	2.87
250829_at	At5g04720	Disease Resistance Protein (CC-NBS-LRR Class), Putative	0.0295	0.0378	3.09
250771_at	At5g05400	Disease Resistance Protein (CC-NBS-LRR Class), Putative	0.0150	0.0269	8.71
249724_at	At5g35450	Disease Resistance Protein (CC-NBS-LRR Class), Putative	0.0457	0.0478	2.51
247413_at	At5g63020	Disease Resistance Protein (CC-NBS-LRR Class), Putative	0.0177	0.0291	4.46
255090_at	At4g09360	Disease Resistance Protein (Nbs-Lrr Class), Putative	0.0187	0.0300	2.68
264910_at	At1g61100	Disease Resistance Protein (Tir Class), Putative	0.0089	0.0206	2.71
260734_at	At1g17600	Disease Resistance Protein (TIR-NBS-LRRClass), Putative	0.0198	0.0308	7.37
254553_at	At4g19530	Disease Resistance Protein (TIR-NBS-LRRClass), Putative	0.0084	0.0200	2.72
250069_at	At5g17970	Disease Resistance Protein (TIR-NBS-LRRClass), Putative	0.0432	0.0463	3.43
250039_at	At5g18370	Disease Resistance Protein (TIR-NBS-LRRClass), Putative	0.0003	0.0043	6.73
249639_at	At5g36930	Disease Resistance Protein (TIR-NBS-LRRClass), Putative	0.0118	0.0239	5.82
248989_at	At5g45200	Disease Resistance Protein (TIR-NBS-LRRClass), Putative	0.0437	0.0466	13.09
248873_at	At5g46450	Disease Resistance Protein (TIR-NBS-LRRClass), Putative	0.0004	0.0050	3.14
252485_at	At3g46530	Disease Resistance Protein, Rpp13-Like (CC-NBS Class), Putative	0.0021	0.0106	2.79
248979_at	At5g45080	Disease Resistance Protein-Related	0.0008	0.0071	3.24
261464_at	At1g07730	Disease Resistance-Responsive Family Protein	0.0389	0.0437	6.48
266978_at	At2g39430	Disease Resistance-Responsive Protein-Related / Dirigent Protein-Related	0.0301	0.0381	3.93
259736_at	At1g64390	Endo-1,4-Beta-Glucanase, Putative / Cellulase, Putative	0.0002	0.0037	2.62
262674_at	At1g75910	Extracellular Lipase 4 (Exl4)	0.0260	0.0354	2.99
252209_at	At3g50400	Gdsl-Motif Lipase/Hydrolase Family Protein	0.0041	0.0145	4.84
249474_s_at	At5g39190	Germin-Like Protein (Ger2)	0.0076	0.0191	5.38
249075_at	At5g44000	Glutathione S-Transferase C-Terminal Domain-Containing Protein	0.0036	0.0138	4.57
259813_at	At1g49860	Glutathione S-Transferase, Putative	0.0258	0.0353	3.70
260796_at	At1g78360	Glutathione S-Transferase, Putative	0.0206	0.0315	16.19
264476_at	At1g77130	Glycogenin Glucosyltransferase (Glycogenin)-Related	0.0125	0.0246	2.58
246402_at	At1g57570	Jacalin Lectin Family Protein	0.0082	0.0199	7.39
259327_at	At3g16460	Jacalin Lectin Family Protein	0.0001	0.0028	2.79
259381_s_at	At3g16390	Jacalin Lectin Family Protein	0.0125	0.0246	4.75
259382_s_at	At3g16430	Jacalin Lectin Family Protein	0.0220	0.0324	2.99
265051_at	At1g52100	Jacalin Lectin Family Protein	0.0025	0.0115	5.03
266989_at	At2g39330	Jacalin Lectin Family Protein	0.0007	0.0067	2.64
254715_at	At4g13550	Lipase Class 3 Family Protein	0.0010	0.0077	2.64
260833_at	At1g06800	Lipase Class 3 Family Protein	0.0301	0.0382	3.96
260393_at	At1g73920	Lipase Family Protein	0.0036	0.0138	3.20
265111_at	At1g62510	Lipid Transfer Protein (Ltp) Family Protein	0.0469	0.0483	2.76
265400_at	At2g10940	Lipid Transfer Protein (Ltp) Family Protein	0.0111	0.0231	2.57
265656_at	At2g13820	Lipid Transfer Protein (Ltp) Family Protein	0.0016	0.0094	5.97
262349_at	At2g48130	Lipid Transfer Protein (Ltp) Family Protein	0.0258	0.0353	4.59
262317_at	At2g48140	Lipid Transfer Protein (Ltp) Family Protein	0.0056	0.0167	10.83
256825_at	At3g22120	Lipid Transfer Protein (Ltp) Family Protein	0.0090	0.0208	4.28
256937_at	At3g22620	Lipid Transfer Protein (Ltp) Family Protein	0.0070	0.0183	3.32
254820_s_at	At4g12510	Lipid Transfer Protein (Ltp) Family Protein	0.0353	0.0415	7.78
248844_s_at	At5g46900	Lipid Transfer Protein (Ltp) Family Protein	0.0054	0.0164	7.04
247718_at	At5g59310	Lipid Transfer Protein 4 (Ltp4)	0.0110	0.0230	4.00
266566_at	At2g24040	Low Temperature And Salt Responsive Protein, Putative	0.0165	0.0282	5.56
266383_at	At2g14580	Pathogenesis-Related Protein, Putative	0.0321	0.0394	4.05
262727_at	At1g75800	Pathogenesis-Related Thaumatin Family Protein	0.0296	0.0379	2.57
254206_at	At4g24180	Pathogenesis-Related Thaumatin Family Protein	0.0001	0.0026	16.94
252949_at	At4g38670	Pathogenesis-Related Thaumatin Family Protein	0.0037	0.0140	2.52
265091_s_at	At1g03495	Pseudogene, Transferase Family	0.0020	0.0103	13.68
259519_at	At1g12270	Stress-Inducible Protein, Putative	0.0029	0.0125	4.67
266165_at	At2g28190	Superoxide Dismutase (Cu-Zn), Chloroplast (Sodcp)	0.0003	0.0044	7.22

259511_at	At1g12520	Superoxide Dismutase Copper Chaperone, Putative	0.0249	0.0347	2.93
257125_at	At3g20050	T-Complex Protein 1 Alpha Subunit / Tcp-1-Alpha / Chaperonin (Cct1)	0.0166	0.0282	2.50
255077_at	At4g09150	T-Complex Protein 11	0.0015	0.0091	2.94
252954_at	At4g38660	Thaumatin, Putative	0.0041	0.0146	5.99
256125_at	At1g18250	Thaumatin, Putative	0.0441	0.0469	4.32
264768_at	At1g61410	Tola Protein, Proline-Related	0.0267	0.0359	3.63
245516_at	At4g15820	Wound-Responsive Protein-Related	0.0001	0.0025	5.53
A1. Protein folding/heat shock/chaperone activity					
256905_at	At3g23990	Chaperonin (Cpn60) (Hsp60)	0.0383	0.0433	2.68
258816_at	At3g03960	Chaperonin, Putative	0.0185	0.0298	2.81
256983_at	At3g13470	Chaperonin, Putative	0.0363	0.0421	3.75
257608_at	At3g13860	Chaperonin, Putative	0.0192	0.0303	2.70
249977_at	At5g18820	Chaperonin, Putative	0.0155	0.0274	10.98
247331_at	At5g63530	Copper Chaperone (Cch)-Related	0.0073	0.0188	4.82
263374_at	At2g20560	DNA J Heat Shock Family Protein	0.0344	0.0409	3.75
258220_at	At3g17830	DNA J Heat Shock Family Protein	0.0284	0.0371	7.15
258986_at	At3g08910	DNA J Heat Shock Family Protein	0.0000	0.0014	2.68
252670_at	At3g44110	DNA J Heat Shock Family Protein	0.0097	0.0215	4.66
260251_at	At1g74250	DNA J Heat Shock N-Terminal Domain-Containing Protein	0.0031	0.0128	4.89
265613_at	At2g25560	DNA J Heat Shock N-Terminal Domain-Containing Protein	0.0011	0.0079	4.17
266858_at	At2g26890	DNA J Heat Shock N-Terminal Domain-Containing Protein	0.0297	0.0379	2.66
265850_at	At2g35720	DNA J Heat Shock N-Terminal Domain-Containing Protein	0.0082	0.0199	4.23
245176_at	At2g47440	DNA J Heat Shock N-Terminal Domain-Containing Protein	0.0182	0.0295	3.26
259087_at	At3g04980	DNA J Heat Shock N-Terminal Domain-Containing Protein	0.0016	0.0095	7.52
257654_at	At3g13310	DNA J Heat Shock N-Terminal Domain-Containing Protein	0.0107	0.0227	2.53
255801_at	At4g10130	DNA J Heat Shock N-Terminal Domain-Containing Protein	0.0007	0.0065	7.68
250004_at	At5g18750	DNA J Heat Shock N-Terminal Domain-Containing Protein	0.0005	0.0056	12.38
259284_at	At3g11450	Dnaj Heat Shock N-Terminal Domain-Containing Protein	0.0021	0.0105	4.36
250995_at	At5g02500	Heat Shock Cognate 70 Kda Protein 1 (Hsc70-1) (Hsp70-1)	0.0120	0.0241	4.56
258979_at	At3g09440	Heat Shock Cognate 70 Kda Protein 3 (Hsc70-3) (Hsp70-3)	0.0023	0.0111	3.18
245379_at	At4g17750	Heat Shock Factor Protein 1 (Hsf1)	0.0034	0.0135	3.90
246450_at	At5g16820	Heat Shock Factor Protein 3 (Hsf3)	0.0328	0.0398	2.60
246554_at	At5g15450	Heat Shock Protein 100, Putative	0.0001	0.0026	5.13
250502_at	At5g09590	Heat Shock Protein 70 / Hsp70 (Hsc70-5)	0.0037	0.0139	6.67
248582_at	At5g49910	Heat Shock Protein 70 / Hsp70 (Hsc70-7)	0.0046	0.0152	2.63
253013_at	At4g37910	Heat Shock Protein 70, Mitochondrial, Putative / Hsp70, Mitochondrial, Putative	0.0015	0.0092	3.64
245293_at	At4g16660	Heat Shock Protein 70, Putative / Hsp70, Putative	0.0075	0.0189	3.08
254148_at	At4g24280	Heat Shock Protein 70, Putative / Hsp70, Putative	0.0026	0.0117	4.68
262054_s_at	At1g79920	Heat Shock Protein 70, Putative / Hsp70, Putative	0.0356	0.0416	3.31
256245_at	At3g12580	Heat Shock Protein 70, Putative / Hsp70, Putative	0.0001	0.0031	5.24
248332_at	At5g52640	Heat Shock Protein 81-1 (Hsp81-1) / Heat Shock Protein 83 (Hsp83)	0.0016	0.0094	6.36
248045_at	At5g56030	Heat Shock Protein 81-2 (Hsp81-2)	0.0000	0.0016	4.63
248043_s_at	At5g56000	Heat Shock Protein 81-4 (Hsp81-4)	0.0001	0.0028	4.75
262814_at	At1g11660	Heat Shock Protein, Putative	0.0000	0.0005	3.83
263483_at	At2g04030	Heat Shock Protein, Putative	0.0185	0.0298	2.68
259248_at	At3g07770	Heat Shock Protein-Related	0.0001	0.0027	2.75
266772_s_at	At2g03020	Heat Shock Protein-Related	0.0339	0.0405	5.85
245956_s_at	At5g28540	Luminal Binding Protein 1 (Bip-1) (Bp1)	0.0009	0.0073	4.60
252644_at	At3g44600	Peptidyl-Prolyl Cis-Trans Isomerase Cyclophilin-Type Family Protein	0.0012	0.0083	3.04
265087_at	At1g03760	Prefoldin Subunit Family Protein	0.0052	0.0161	2.81
248094_at	At5g55220	Trigger Factor Type Chaperone Family Protein	0.0101	0.0220	2.53
A2. Cell wall associated transcripts					
A2.1. Reassmebly of cell wall associated genes					
264656_at	At1g09010	Glycoside Hydrolase Family 2 Protein	0.0001	0.0023	2.94
262299_at	At1g27520	Glycoside Hydrolase Family 47 Protein	0.0293	0.0377	2.54
245094_at	At2g40840	Glycoside Hydrolase Family 77 Protein	0.0037	0.0139	2.77
246829_at	At5g26570	Glycoside Hydrolase Starch-Binding Domain-Containing Protein	0.0024	0.0112	4.15
262118_at	At1g02850	Glycosyl Hydrolase Family 1 Protein	0.0393	0.0439	3.28
258512_at	At3g06510	Glycosyl Hydrolase Family 1 Protein	0.0052	0.0161	4.16
258151_at	At3g18080	Glycosyl Hydrolase Family 1 Protein	0.0247	0.0345	4.13
249636_at	At5g36890	Glycosyl Hydrolase Family 1 Protein	0.0439	0.0467	5.06
259640_at	At1g52400	Glycosyl Hydrolase Family 1 Protein / Beta-Glucosidase, Putative (Bg1)	0.0241	0.0341	2.80
249235_at	At5g42100	Glycosyl Hydrolase Family 17 Protein	0.0008	0.0071	3.32
247845_at	At5g58090	Glycosyl Hydrolase Family 17 Protein	0.0428	0.0460	2.60
262181_at	At1g78060	Glycosyl Hydrolase Family 3 Protein	0.0284	0.0371	2.63
252445_at	At3g47000	Glycosyl Hydrolase Family 3 Protein	0.0127	0.0248	3.89
251228_at	At3g62710	Glycosyl Hydrolase Family 3 Protein	0.0048	0.0155	5.39
247266_at	At5g64570	Glycosyl Hydrolase Family 3 Protein	0.0051	0.0160	5.26
250604_at	At5g07830	Glycosyl Hydrolase Family 79 N-Terminal Domain-Containing Protein	0.0353	0.0414	2.77
261665_at	At1g18310	Glycosyl Hydrolase Family 81 Protein	0.0392	0.0438	3.52
267595_at	At2g32990	Glycosyl Hydrolase Family 9 Protein	0.0424	0.0457	2.61

255054_s_at	At4g09740	Glycosyl Hydrolase Family 9 Protein	0.0166	0.0282	4.46
260584_at	At2g43660	Glycosyl Hydrolase Family Protein 17	0.0343	0.0408	5.64
247563_at	At5g61130	Glycosyl Hydrolase Family Protein 17	0.0078	0.0193	3.75
252740_at	At3g43270	Pectinesterase Family Protein	0.0059	0.0170	2.78
250490_at	At5g09760	Pectinesterase Family Protein	0.0010	0.0078	4.10
248263_at	At5g53370	Pectinesterase Family Protein	0.0259	0.0354	3.96
A2.2. Matrix polymers					
245725_at	At1g73370	Glucosyltransferase, Putative	0.0152	0.0272	3.03
255521_at	At4g02280	Glucosyltransferase, Putative	0.0450	0.0474	3.15
257797_at	At3g15940	Glycosyl Transferase Family 1 Protein	0.0059	0.0170	3.06
259041_at	At3g07330	Glycosyl Transferase Family 2 Protein	0.0478	0.0488	3.21
263019_at	At1g23870	Glycosyl Transferase Family 20 Protein / Trehalose-Phosphatase Family Protein	0.0076	0.0192	3.98
264246_at	At1g60140	Glycosyl Transferase Family 20 Protein / Trehalose-Phosphatase Family Protein	0.0037	0.0139	2.82
266072_at	At2g18700	Glycosyl Transferase Family 20 Protein / Trehalose-Phosphatase Family Protein	0.0015	0.0090	3.55
245348_at	At4g17770	Glycosyl Transferase Family 20 Protein / Trehalose-Phosphatase Family Protein	0.0011	0.0081	3.54
265729_at	At2g31960	Glycosyl Transferase Family 48 Protein	0.0076	0.0191	3.68
263891_at	At2g36850	Glycosyl Transferase Family 48 Protein	0.0017	0.0098	4.08
250272_at	At5g13000	Glycosyl Transferase Family 48 Protein	0.0009	0.0071	2.69
253037_at	At4g38270	Glycosyl Transferase Family 8 Protein	0.0067	0.0180	3.61
254552_at	At4g19900	Glycosyl Transferase-Related	0.0151	0.0271	6.65
262744_at	At1g28680	Transferase Family Protein	0.0289	0.0374	2.57
267440_at	At2g19070	Transferase Family Protein	0.0335	0.0402	3.72
258070_at	At3g26040	Transferase Family Protein	0.0018	0.0098	9.15
256924_at	At3g29590	Transferase Family Protein	0.0037	0.0140	5.50
248639_at	At5g48930	Transferase Family Protein	0.0005	0.0056	3.82
263176_at	At1g05530	Udp-Glucoronosyl/Udp-Glucosyl Transferase Family Protein	0.0140	0.0260	8.39
266532_at	At2g16890	Udp-Glucoronosyl/Udp-Glucosyl Transferase Family Protein	0.0138	0.0258	8.49
263477_at	At2g31790	Udp-Glucoronosyl/Udp-Glucosyl Transferase Family Protein	0.0117	0.0237	3.04
257205_at	At3g16520	Udp-Glucoronosyl/Udp-Glucosyl Transferase Family Protein	0.0000	0.0006	2.72
257949_at	At3g21750	Udp-Glucoronosyl/Udp-Glucosyl Transferase Family Protein	0.0001	0.0027	2.94
252476_at	At3g46650	Udp-Glucoronosyl/Udp-Glucosyl Transferase Family Protein	0.0312	0.0388	5.92
252482_at	At3g46670	Udp-Glucoronosyl/Udp-Glucosyl Transferase Family Protein	0.0005	0.0054	3.47
250750_at	At5g05870	Udp-Glucoronosyl/Udp-Glucosyl Transferase Family Protein	0.0181	0.0295	2.93
246826_at	At5g26310	Udp-Glucoronosyl/Udp-Glucosyl Transferase Family Protein	0.0387	0.0436	3.46
A2.3.Structural proteins					
266552_at	At2g46330	Arabinogalactan-Protein (Agp16)	0.0091	0.0208	3.78
266588_at	At2g14890	Arabinogalactan-Protein (Agp9)	0.0337	0.0404	5.94
262849_at	At1g14710	Hydroxyproline-Rich Glycoprotein Family Protein	0.0305	0.0384	5.97
260984_at	At1g53645	Hydroxyproline-Rich Glycoprotein Family Protein	0.0019	0.0101	4.66
245655_at	At1g56530	Hydroxyproline-Rich Glycoprotein Family Protein	0.0235	0.0336	8.00
262187_at	At1g77970	Hydroxyproline-Rich Glycoprotein Family Protein	0.0259	0.0354	2.52
261347_at	At1g79730	Hydroxyproline-Rich Glycoprotein Family Protein	0.0029	0.0125	3.81
255838_at	At2g33490	Hydroxyproline-Rich Glycoprotein Family Protein	0.0151	0.0270	14.89
266170_at	At2g39050	Hydroxyproline-Rich Glycoprotein Family Protein	0.0325	0.0397	2.53
266460_at	At2g47930	Hydroxyproline-Rich Glycoprotein Family Protein	0.0145	0.0264	3.57
258206_at	At3g14010	Hydroxyproline-Rich Glycoprotein Family Protein	0.0004	0.0052	3.10
258455_at	At3g22440	Hydroxyproline-Rich Glycoprotein Family Protein	0.0108	0.0228	4.38
258282_at	At3g26910	Hydroxyproline-Rich Glycoprotein Family Protein	0.0043	0.0148	4.44
253800_at	At4g28160	Hydroxyproline-Rich Glycoprotein Family Protein	0.0117	0.0238	7.31
250500_at	At5g09530	Hydroxyproline-Rich Glycoprotein Family Protein	0.0201	0.0310	90.14
247149_at	At5g65660	Hydroxyproline-Rich Glycoprotein Family Protein	0.0031	0.0128	2.87
264226_at	At1g67510	Leucine-Rich Repeat Family Protein	0.0211	0.0317	4.76
267310_at	At2g34680	Leucine-Rich Repeat Family Protein	0.0019	0.0102	2.92
258409_at	At3g17640	Leucine-Rich Repeat Family Protein	0.0190	0.0301	10.30
248945_at	At5g45510	Leucine-Rich Repeat Family Protein	0.0025	0.0115	2.55
245202_at	At1g67720	Leucine-Rich Repeat Family Protein	0.0140	0.0259	3.53
265467_at	At2g37050	Leucine-Rich Repeat Family Protein	0.0018	0.0099	4.24
256981_at	At3g13380	Leucine-Rich Repeat Family Protein	0.0273	0.0363	5.09
250642_at	At5g07180	Leucine-Rich Repeat Family Protein	0.0412	0.0451	6.85
263590_at	At2g01820	Leucine-Rich Repeat Family Protein	0.0087	0.0204	4.62
267376_at	At2g26330	Leucine-Rich Repeat Family Protein	0.0003	0.0042	4.76
256516_at	At1g66150	Leucine-Rich Repeat Family Protein	0.0281	0.0369	3.94
253769_at	At4g28560	Leucine-Rich Repeat Family Protein (Fragment)	0.0016	0.0093	7.08
262393_at	At1g49490	Leucine-Rich Repeat Family Protein / Extensin Family Protein	0.0422	0.0456	2.77
258342_at	At3g22800	Leucine-Rich Repeat Family Protein / Extensin Family Protein	0.0277	0.0366	2.89
248363_at	At5g52480	Leucine-Rich Repeat Protein, N7-Related	0.0485	0.0493	4.49
247445_at	At5g62640	Proline-Rich Family Protein	0.0195	0.0305	2.91
252161_at	At3g50580	Proline-Rich Family Protein	0.0018	0.0099	3.96
252971_at	At4g38770	Proline-Rich Family Protein (Prp4)	0.0435	0.0465	3.60
B. Cellular communication/signal transduction mechanism/transmembrane signal transduction					
262506_at	At1g21640	Atp-Nad Kinase Family Protein	0.0333	0.0401	3.27

246284_at	At4g36780	Brassinosteroid Signalling Positive Regulator-Related	0.0027	0.0119	16.42
250393_at	At5g10900	Calcineurin	0.0066	0.0179	10.38
247137_at	At5g66210	Calcium-Dependent Protein Kinase Family Protein / Cdpk Family Protein	0.0016	0.0093	2.63
267531_at	At2g41860	Calcium-Dependent Protein Kinase, Putative / Cdpk, Putative	0.0060	0.0171	5.29
251636_at	At3g57530	Calcium-Dependent Protein Kinase, Putative / Cdpk, Putative	0.0255	0.0352	3.32
261433_s_at	At1g07670	Calcium-Transporting Atpase 4, Endoplasmic Reticulum-Type (Eca4)	0.0208	0.0316	4.64
253995_at	At4g26100	Casein Kinase, Putative	0.0068	0.0181	3.70
257938_at	At3g19820	Cell Elongation Protein / Dwarf1 / Diminuto (Dim)	0.0240	0.0340	5.46
250632_at	At5g07450	Cyclin Family Protein	0.0004	0.0050	7.08
247536_at	At5g61650	Cyclin Family Protein	0.0037	0.0139	3.79
260504_at	At1g47220	Cyclin, Putative	0.0042	0.0147	6.55
267618_at	At2g26760	Cyclin, Putative	0.0195	0.0305	3.87
263017_at	At2g17620	Cyclin, Putative (Cyc2A)	0.0154	0.0273	3.44
250369_at	At5g11300	Cyclin, Putative (Cyc3B)	0.0013	0.0086	8.53
262833_at	At1g14750	Cyclin, Putative (Sds)	0.0013	0.0085	18.39
250451_at	At5g10270	Cyclin-Dependent Kinase, Putative / Cdk, Putative	0.0382	0.0432	2.92
257073_at	At3g19650	Cyclin-Related	0.0113	0.0233	4.58
245478_at	At4g16130	Ghmp Kinase Family Protein	0.0153	0.0272	3.78
246286_at	At1g31910	Ghmp Kinase Family Protein	0.0191	0.0302	2.65
261043_at	At1g01220	Ghmp Kinase-Related	0.0071	0.0185	2.50
258341_at	At3g22790	Kinase Interacting Family Protein	0.0007	0.0066	8.59
267518_at	At2g30500	Kinase Interacting Family Protein	0.0078	0.0193	3.54
252975_s_at	At4g38430	Kinase protein like gene family	0.0019	0.0101	2.82
247626_at	At5g60300	Lectin Protein Kinase Family Protein	0.0019	0.0100	2.51
266281_at	At2g29250	Lectin Protein Kinase, Putative	0.0241	0.0341	7.27
255503_at	At4g02420	Lectin Protein Kinase, Putative	0.0487	0.0494	3.25
255121_at	At4g08480	Mitogen-Activated Protein Kinase, Putative	0.0491	0.0495	2.64
249964_at	At5g19010	Mitogen-Activated Protein Kinase, Putative / Mapk, Putative (Mpk16)	0.0227	0.0331	5.49
255806_at	At4g10260	Pfkb-Type Carbohydrate Kinase Family Protein	0.0363	0.0421	3.87
252459_s_at	At3g47220	Phosphoinositide-Specific Phospholipase C Family Protein	0.0019	0.0101	2.91
267536_at	At2g42010	Phospholipase D Beta 1 / Pld Beta 1 (Pldbeta1)	0.0206	0.0315	3.92
265522_at	At2g06210	Phosphoprotein-Related	0.0262	0.0356	9.07
256982_at	At3g13460	Physically interacts with CIPK1	0.0314	0.0389	2.96
245358_at	At4g15900	Pp1/Pp2A Phosphatases Pleiotropic Regulator 1 (Prl1)	0.0237	0.0338	2.70
251315_at	At3g61410	Protein kinase	0.0032	0.0130	6.77
251994_at	At3g52890	Protein Kinase (Kipk)	0.0216	0.0321	3.01
264602_at	At1g04700	Protein Kinase Family Protein	0.0155	0.0274	5.17
245845_at	At1g26150	Protein Kinase Family Protein	0.0266	0.0358	2.95
260036_at	At1g68830	Protein Kinase Family Protein	0.0040	0.0145	3.50
260362_at	At1g70530	Protein Kinase Family Protein	0.0036	0.0138	2.55
261883_at	At1g80870	Protein Kinase Family Protein	0.0423	0.0456	2.51
264621_at	At2g17700	Protein Kinase Family Protein	0.0461	0.0479	2.77
265923_at	At2g18470	Protein Kinase Family Protein	0.0172	0.0286	2.55
265308_at	At2g20300	Protein Kinase Family Protein	0.0176	0.0291	17.00
267599_at	At2g32850	Protein Kinase Family Protein	0.0016	0.0094	5.08
267422_at	At2g35050	Protein Kinase Family Protein	0.0010	0.0078	5.32
256783_at	At3g13670	Protein Kinase Family Protein	0.0040	0.0145	3.90
255559_at	At4g02010	Protein Kinase Family Protein	0.0415	0.0452	4.96
254251_at	At4g23300	Protein Kinase Family Protein	0.0187	0.0299	2.82
251122_at	At5g01020	Protein Kinase Family Protein	0.0320	0.0393	2.64
251068_at	At5g01920	Protein Kinase Family Protein	0.0023	0.0110	3.99
250341_at	At5g11850	Protein Kinase Family Protein	0.0013	0.0087	4.57
249950_at	At5g18910	Protein Kinase Family Protein	0.0450	0.0474	3.32
247918_at	At5g57610	Protein Kinase Family Protein	0.0224	0.0328	2.68
247661_at	At5g60080	Protein Kinase Family Protein	0.0002	0.0037	3.99
247853_at	At5g58140	Protein Kinase Family Protein / Non Phototropic Hypocotyl 1-Like Protein (Npl1)	0.0034	0.0133	4.59
262106_at	At1g02970	Protein Kinase, Putative	0.0124	0.0245	11.20
264625_at	At1g09020	Protein Kinase, Putative	0.0257	0.0353	4.13
259887_at	At1g76360	Protein Kinase, Putative	0.0490	0.0495	3.11
265581_at	At2g20040	Protein Kinase, Putative	0.0011	0.0080	2.63
259083_at	At3g04810	Protein Kinase, Putative	0.0467	0.0483	14.19
257696_at	At3g12690	Protein Kinase, Putative	0.0007	0.0063	4.68
258186_s_at	At3g17850	Protein Kinase, Putative	0.0326	0.0397	4.52
254560_at	At4g19110	Protein Kinase, Putative	0.0270	0.0361	3.01
254269_at	At4g23050	Protein Kinase, Putative	0.0060	0.0172	3.01
253976_at	At4g26610	Protein Kinase, Putative	0.0357	0.0416	2.87
248720_at	At5g47750	Protein Kinase, Putative	0.0257	0.0353	3.68
247334_at	At5g63610	Protein Kinase, Putative	0.0004	0.0050	4.98
248300_at	At5g53000	Protein Phosphatase 2A-Associated 46 Kda Protein	0.0174	0.0288	2.84
266758_at	At2g46920	Protein Phosphatase 2C Family Protein / Pp2C Family Protein	0.0001	0.0025	3.74
257887_at	At3g17090	Protein Phosphatase 2C Family Protein / Pp2C Family Protein	0.0161	0.0279	2.88
261077_at	At1g07430	Protein Phosphatase 2C, Putative / Pp2C, Putative	0.0000	0.0006	3.44
255910_at	At1g18030	Protein Phosphatase 2C, Putative / Pp2C, Putative	0.0019	0.0101	2.87
258901_at	At3g05640	Protein Phosphatase 2C, Putative / Pp2C, Putative	0.0024	0.0112	2.55

248428_at	At5g51760	Protein Phosphatase 2C, Putative / Pp2C, Putative	0.0391	0.0438	4.80
264730_at	At1g62090	Pseudogene, Protein Kinase Family	0.0017	0.0096	9.35
245528_at	At4g15530	Pyruvate Phosphate Dikinase Family Protein	0.0269	0.0361	3.73
251521_at	At3g59420	Receptor Protein Kinase, Putative (Acr4)	0.0011	0.0081	14.41
263159_at	At1g54130	Rela/Spot Protein, Putative (Rsh3)	0.0162	0.0280	2.96
253612_at	At4g30310	Ribitol Kinase, Putative	0.0103	0.0222	3.02
265772_at	At2g48010	Serine/Threonine Protein Kinase (Rfk3)	0.0097	0.0215	4.31
267564_at	At2g30740	Serine/Threonine Protein Kinase, Putative	0.0236	0.0338	2.85
266453_at	At2g43230	Serine/Threonine Protein Kinase, Putative	0.0107	0.0226	9.76
258463_at	At3g17410	Serine/Threonine Protein Kinase, Putative	0.0499	0.0499	3.36
246917_at	At5g25280	Serine-Rich Protein-Related	0.0407	0.0448	2.60
264686_at	At1g09840	Shaggy-Related Protein Kinase Kappa / Ask-Kappa (Ask10)	0.0238	0.0339	2.74
255635_at	At4g00720	Shaggy-Related Protein Kinase Theta / Ask-Theta (Ask8)	0.0228	0.0331	6.47
261873_at	At1g11350	S-Locus Lectin Protein Kinase Family Protein	0.0014	0.0089	2.51
261819_at	At1g11410	S-Locus Protein Kinase, Putative	0.0044	0.0149	2.95
264764_at	At1g61440	S-Locus Protein Kinase, Putative	0.0147	0.0267	4.19
258925_at	At3g10420	Sporulation Protein-Related	0.0228	0.0331	3.27
264776_at	At1g22860	Tgf Beta Receptor Associated Protein-Related	0.0002	0.0036	3.92
256790_at	At3g16857	Two-Component Responsive Regulator Family Protein	0.0230	0.0332	3.52
C. Cell Cycle/ DNA processing/chromatin regulation and cytoskeleton reorganization					
267175_s_at	At2g37620	Actin 1 (Act1)	0.0289	0.0373	2.97
257749_at	At3g18780	Actin 2 (Act2)	0.0035	0.0135	2.55
260765_at	At1g49240	Actin 8 (Act8)	0.0140	0.0260	2.74
267030_at	At2g38440	Actin microfilament	0.0001	0.0030	5.24
245232_at	At4g25590	Actin-Depolymerizing Factor, Putative	0.0099	0.0218	3.39
259513_at	At1g12430	Armadillo/Beta-Catenin Repeat Family Protein / Kinesin Motor Family Protein	0.0186	0.0299	2.50
262802_at	At1g20930	Cell Division Control Protein, Putative	0.0062	0.0174	11.94
258649_at	At3g09840	Cell Division Cycle Protein 48 (Cdc48A) (Cdc48)	0.0019	0.0100	5.78
251975_at	At3g53230	Cell Division Cycle Protein 48, Putative / Cdc48, Putative	0.0090	0.0207	4.57
250899_at	At5g03340	Cell Division Cycle Protein 48, Putative / Cdc48, Putative	0.0011	0.0078	3.85
262473_at	At1g50250	Cell Division Protein Ftsh Homolog 1, Chloroplast (Ftsh1) (Ftsh)	0.0068	0.0181	2.86
267603_at	At2g32900	Centromere/Kinetochore Protein, Putative (Zw10)	0.0188	0.0300	5.33
253425_at	At4g32190	Centromeric Protein-Related	0.0064	0.0176	5.51
264486_at	At1g77180	Chromatin Protein Family	0.0000	0.0004	2.75
264384_at	At2g25170	Chromatin Remodeling Factor Chd3 (Pickle)	0.0242	0.0341	3.47
248597_at	At5g49160	Dna (Cytosine-5-)-Methyltransferase (Athim)	0.0006	0.0060	5.98
255562_at	At4g02070	Dna Mismatch Repair Protein Msh6-1 (Msh6-1) (Agaa.3)	0.0053	0.0163	4.41
264227_at	At1g67500	Dna Polymerase Family B Protein	0.0268	0.0359	4.84
264969_at	At1g67320	Dna Primase, Large Subunit Family	0.0265	0.0357	3.55
265664_at	At2g24420	Dna Repair Atpase-Related	0.0099	0.0218	2.82
257027_at	At3g19210	Dna Repair Protein Rad54, Putative	0.0227	0.0331	3.64
264099_at	At1g79050	Dna Repair Protein Reca	0.0016	0.0095	2.69
249307_s_at	At5g41370	Dna Repair Protein, Putative	0.0068	0.0181	3.22
248555_at	At5g50340	Dna Repair Protein-Related	0.0111	0.0231	2.72
265678_at	At2g31970	Dna Repair-Recombination Protein (Rad50)	0.0365	0.0422	2.93
248099_at	At5g55300	Dna Topoisomerase I	0.0303	0.0382	2.80
248109_at	At5g55310	Dna Topoisomerase I, Putative	0.0069	0.0182	3.45
247301_at	At5g63920	Dna Topoisomerase Iii Alpha, Putative	0.0081	0.0198	2.69
256237_at	At3g12610	Dna-Damage-Repair/Toleration Protein, Putative (Drt100)	0.0314	0.0389	2.98
258904_at	At3g06400	Dna-Dependent Atpase, Putative	0.0008	0.0069	4.83
249997_at	At5g18620	Dna-Dependent Atpase, Putative	0.0242	0.0342	8.19
258148_s_at	At3g18090	Dna-Directed Rna Polymerase Family Protein	0.0461	0.0479	2.55
253133_at	At4g35800	Dna-Directed Rna Polymerase Ii Largest Subunit (Rpb205) (Rpii) (Rpb1)	0.0163	0.0280	3.18
259672_at	At1g68990	Dna-Directed Rna Polymerase, Mitochondrial (Rpomt)	0.0031	0.0128	4.86
259908_at	At1g60850	Dna-Directed Rna Polymerase, Putative	0.0162	0.0280	6.49
257044_at	At3g19720	Dynamin Family Protein	0.0010	0.0075	3.52
264406_at	At1g10290	Dynamin-Like Protein 6 (Adl6)	0.0039	0.0142	2.69
262837_at	At1g14830	Dynamin-Like Protein C (Dl1C)	0.0465	0.0481	2.91
262077_at	At1g59610	Dynamin-Like Protein, Putative (Adl3)	0.0008	0.0070	4.87
266541_at	At2g35110	Hem Protein-Related	0.0215	0.0321	3.67
263909_at	At2g36490	Hhh-Gpd Base Excision Dna Repair Family Protein (Ros1)	0.0002	0.0035	11.54
257854_at	At3g12980	Histone Acetyltransferase 5 (Hac5)	0.0009	0.0073	3.10
247559_at	At5g61070	Histone Deacetylase Family Protein (Hda18)	0.0283	0.0370	7.52
246881_at	At5g26040	Histone Deacetylase Family Protein (Hda2)	0.0033	0.0133	4.06
261720_at	At1g08460	Histone Deacetylase Family Protein (Hda8)	0.0152	0.0272	2.73
261303_at	At1g48620	Histone H1/H5 Family Protein	0.0041	0.0145	3.39
258141_at	At3g18035	Histone H1/H5 Family Protein	0.0018	0.0099	2.86
254717_at	At4g13570	Histone H2A, Putative	0.0000	0.0006	4.88
260622_at	At1g07980	Histone-Like Transcription Factor (Cbf/Nf-Y) Family Protein	0.0030	0.0126	3.77
246095_at	At5g19310	Homeotic Gene Regulator, Putative	0.0084	0.0201	13.89
252916_at	At4g38950	Kinesin Motor Family Protein	0.0010	0.0077	12.37
261557_at	At1g63640	Kinesin Motor Protein-Related	0.0001	0.0021	22.54

263762_at	At2g21380	Kinesin Motor Protein-Related	0.0000	0.0017	3.04
252548_at	At3g45850	Kinesin Motor Protein-Related	0.0002	0.0034	4.26
252215_at	At3g50240	Kinesin Motor Protein-Related	0.0022	0.0107	10.21
250685_at	At5g06670	Kinesin Motor Protein-Related	0.0224	0.0328	10.86
250429_at	At5g10470	Kinesin Motor Protein-Related	0.0005	0.0057	3.89
247115_at	At5g65930	Kinesin-Like Calmodulin-Binding Protein (Zwichel)	0.0052	0.0161	3.16
248773_at	At5g47820	Kinesin-Like Protein (Fra1)	0.0012	0.0083	2.56
253903_at	At4g27180	Kinesin-Like Protein B (Katb)	0.0035	0.0135	4.20
264972_at	At1g67370	Meiotic Asynaptic Mutant 1 (Asy1)	0.0293	0.0377	9.08
255093_s_at	At4g08580	Microfibrillar-Associated Protein-Related	0.0217	0.0322	9.74
248095_at	At5g55230	Microtubule Associated Protein (Map65/Ase1) Family Protein	0.0241	0.0341	2.60
262847_at	At1g14840	Microtubule association protein	0.0227	0.0331	10.60
265864_at	At2g01750	Microtubule association protein	0.0002	0.0039	15.07
256623_at	At3g19960	Myosin (Atm)	0.0095	0.0213	3.00
259572_at	At1g20400	Myosin Heavy Chain-Related	0.0364	0.0421	4.61
263189_at	At1g36100	Myosin Heavy Chain-Related	0.0273	0.0363	4.52
265464_at	At2g37080	Myosin Heavy Chain-Related	0.0027	0.0119	4.85
266589_at	At2g46250	Myosin Heavy Chain-Related	0.0292	0.0376	3.19
251992_at	At3g53350	Myosin Heavy Chain-Related	0.0000	0.0017	20.53
247738_at	At5g59210	Myosin Heavy Chain-Related	0.0147	0.0266	2.57
264599_at	At1g04600	Myosin, Putative	0.0385	0.0434	6.36
260711_at	At1g17580	Myosin, Putative	0.0004	0.0050	2.55
246081_s_at	At5g20470	Myosin, Putative	0.0406	0.0447	2.71
264572_at	At1g05320	Myosin-Related	0.0002	0.0033	2.53
256204_at	At1g50840	Poli-Like Dna Polymerase, Putative	0.0029	0.0125	2.79
264077_at	At2g28560	Protein of the RAD51B family	0.0214	0.0320	4.57
246500_at	At5g16270	Rad21/Rec8-Like Family Protein	0.0445	0.0471	5.93
245054_at	At2g26460	Red Family Protein	0.0246	0.0344	2.97
248693_at	At5g48330	Regulator Of Chromosome Condensation (Rcc1) Family Protein	0.0280	0.0368	3.05
263532_s_at	At5g37350	Rio1 Family Protein	0.0017	0.0098	3.09
245917_at	At5g28740	Transcription-Coupled Dna Repair Protein-Related	0.0000	0.0011	2.60
261129_at	At1g04820	Tubulin Alpha-2/Alpha-4 Chain (Tua4)	0.0003	0.0040	3.05
245915_s_at	At5g19770	Tubulin Alpha-3/Alpha-5 Chain (Tua3)	0.0024	0.0112	4.67
247442_s_at	At5g62690	Tubulin Beta-2/Beta-3 Chain (Tub2)	0.0069	0.0181	5.37
250317_at	At5g12250	Tubulin Beta-6 Chain (Tub6)	0.0243	0.0342	2.59
D. Development/storage proteins					
265095_at	At1g03880	12S Seed Storage Protein (Crb)	0.0206	0.0314	5.46
245492_at	At4g16340	Adapter Protein Spike1 (Spk1)	0.0274	0.0364	2.56
258392_at	At3g15400	Anther Development Protein, Putative	0.0052	0.0161	3.69
264113_at	At2g31260	Autophagy 9 (Apg9)	0.0330	0.0399	2.51
254780_s_at	At4g12770	Auxilin-Related	0.0135	0.0256	2.67
247468_at	At5g62010	Auxin response transcription factor	0.0133	0.0254	2.86
259128_at	At3g02260	Auxin Transport Protein (Big)	0.0047	0.0153	3.90
266300_at	At2g01420	Auxin Transport Protein, Putative	0.0029	0.0125	2.84
262914_at	At1g59750	Auxin-Responsive Transcription Factor (Arf1)	0.0090	0.0208	3.17
267452_at	At2g33860	Auxin-Responsive Transcription Factor (Arf3) / Ettin Protein (Ett)	0.0223	0.0327	6.97
256311_at	At1g30330	Auxin-Responsive Transcription Factor (Arf6)	0.0338	0.0405	4.52
245971_at	At5g20730	Auxin-Responsive Transcription Factor (Arf7)	0.0019	0.0102	5.85
254194_at	At4g23980	Auxin-Responsive Transcription Factor (Arf9)	0.0061	0.0173	5.27
266611_at	At2g14960	Auxin-Responsive Gh3 Family Protein	0.0001	0.0029	6.63
259018_at	At3g07390	Auxin-Responsive Protein / Auxin-Induced Protein (Air12)	0.0469	0.0483	3.53
264021_at	At2g21200	Auxin-Responsive Protein, Putative	0.0068	0.0181	6.51
254746_at	At4g12980	Auxin-Responsive Protein, Putative	0.0087	0.0204	8.24
252431_at	At3g47700	Chromosome Structural Maintenance Protein-Related	0.0286	0.0372	3.43
261415_at	At1g07750	Cupin Family Protein	0.0013	0.0085	3.50
249193_at	At5g42480	DnaJ Plastid Division Protein (Arc6)	0.0001	0.0019	3.89
245668_at	At1g28330	Dormancy-Associated Protein, Putative (Drm1)	0.0103	0.0222	20.35
266368_at	At2g41380	Embryo-Abundant Protein-Related	0.0003	0.0043	4.02
265896_at	At2g25660	Embryonic development	0.0009	0.0073	2.59
246476_at	At5g16730	Embryonic development	0.0046	0.0152	5.57
257922_at	At3g23150	Ethylene Receptor, Putative (Etr2)	0.0028	0.0120	6.23
250928_at	At5g03280	Ethylene-Insensitive 2 (Ein2)	0.0000	0.0006	3.40
245256_at	At4g15090	Far-Red Impaired Response Protein (Far1) C462	0.0023	0.0111	38.77
259259_at	At3g11540	Gibberellin Signal Transduction Protein (Spindly)	0.0006	0.0063	2.59
246550_at	At5g14920	Gibberellin-Regulated Family Protein	0.0312	0.0388	3.04
255049_at	At4g09610	Gibberellin-Regulated Protein 2 (Gasa2) / Gibberellin-Responsive Protein 2	0.0434	0.0464	4.80
255128_at	At4g08310	Glutamic acid-rich protein	0.0043	0.0148	5.07
251678_at	At3g56990	Glycine-Rich Protein	0.0398	0.0442	3.68
253754_at	At4g29020	Glycine-Rich Protein	0.0090	0.0207	3.40
253347_at	At4g33610	Glycine-Rich Protein	0.0218	0.0323	6.39
250355_at	At5g11700	Glycine-Rich Protein	0.0061	0.0173	4.57
258240_at	At3g27660	Glycine-Rich Protein / Oleosin	0.0455	0.0477	5.25

249353_at	At5g40420	Glycine-Rich Protein / Oleosin	0.0060	0.0172	7.62
251390_at	At3g60860	Guanine Nucleotide Exchange Family Protein	0.0002	0.0032	3.05
261621_at	At1g01960	Guanine Nucleotide Exchange Family Protein	0.0123	0.0245	2.84
266905_at	At2g34560	Katanin, Putative	0.0021	0.0105	2.96
252019_at	At3g53040	Late Embryogenesis Abundant Protein, Putative / Lea Protein, Putative	0.0052	0.0161	3.35
262128_at	At1g52690	Late Embryogenesis Abundant Protein, Putative / Lea Protein, Putative	0.0245	0.0343	8.97
262976_at	At1g75520	Lateral Root Primordium (Lrp) Protein-Related	0.0065	0.0178	4.94
264145_at	At1g79310	Latex-Abundant Protein, Putative (Amc4) / Caspase Family Protein	0.0005	0.0056	6.88
247565_at	At5g61150	Leo1-Like Family Protein	0.0069	0.0181	2.72
263865_at	At2g36910	Multidrug Resistance P-Glycoprotein (Pgp1)	0.0001	0.0022	4.32
254034_at	At4g25960	Multidrug Resistance P-Glycoprotein, Putative	0.0202	0.0311	2.73
257137_at	At3g28860	Multidrug Resistance P-Glycoprotein, Putative	0.0085	0.0201	4.10
259579_at	At1g28010	Multidrug Resistance P-Glycoprotein, Putative	0.0300	0.0381	3.11
247084_at	At5g66300	No Apical Meristem (Nam) Family Protein	0.0004	0.0050	6.44
248240_at	At5g53950	No Apical Meristem (Nam) Family Protein	0.0000	0.0017	7.92
249944_at	At5g22290	No Apical Meristem (Nam) Family Protein	0.0009	0.0073	3.04
262514_at	At1g34190	No Apical Meristem (Nam) Family Protein	0.0061	0.0173	2.84
260288_at	At1g80530	Nodulin Family Protein	0.0116	0.0236	3.76
248804_at	At5g47470	Nodulin Mtn21 Family Protein	0.0364	0.0421	4.06
249397_at	At5g40230	Nodulin-Related	0.0128	0.0250	8.95
267044_at	At2g34350	Nodulin-Related	0.0443	0.0470	8.18
247157_at	At5g65770	Nuclear Matrix Constituent Protein-Related	0.0304	0.0384	8.83
262773_at	At1g13220	Nuclear Matrix Constituent Protein-Related	0.0115	0.0235	4.72
257000_at	At3g14120	Nuclear pore complex protein NUP 107	0.0151	0.0270	2.72
250769_at	At5g05680	Nuclear Pore Complex Protein-Related	0.0004	0.0048	2.88
265724_at	At2g32100	Ovate Protein-Related	0.0149	0.0269	3.02
261395_at	At1g79700	Ovule Development Protein, Putative	0.0489	0.0495	3.34
262316_at	At2g48120	Pale Cress Protein (Pac)	0.0452	0.0475	5.29
252985_at	At4g38350	Patched Family Protein	0.0026	0.0117	2.75
262614_at	At1g13980	Pattern Formation Protein (Emb30) (Gnom)	0.0007	0.0068	3.29
245688_at	At1g28290	Pollen Ole E 1 Allergen And Extensin Family Protein	0.0208	0.0316	4.06
265567_at	At2g05580	Pseudogene, Glycine-Rich Protein	0.0059	0.0170	5.18
257350_x_at	At2g19040	Rapid Alkalinization Factor (Ralf) Family Protein	0.0358	0.0417	3.89
258432_at	At3g16570	Rapid Alkalinization Factor (Ralf) Family Protein	0.0057	0.0169	4.81
257606_at	At3g13870	Root Hair Defective 3 (Rhd3)	0.0005	0.0058	4.96
254166_at	At4g24190	Shepherd Protein (Shd) / Clavata Formation Protein, Putative	0.0175	0.0290	5.51
246628_at	At1g48900	Signal Recognition Particle 54 Kda Protein 3 / Srp54 (Srp-54C)	0.0004	0.0053	2.89
250884_at	At5g03940	Signal Recognition Particle 54 Kda Protein, Chloroplast / 54 Chloroplast Protein / Srp54 (Ffc)	0.0026	0.0117	2.57
247505_at	At5g61970	Signal Recognition Particle-Related / Srp-Related	0.0059	0.0170	2.54
245712_at	At5g04360	Starch Debranching Enzyme, Putative	0.0447	0.0472	3.31
262784_at	At1g10760	Starch Excess Protein (Sex1)	0.0002	0.0039	3.72
260751_at	At1g49040	Stomatal Cytokinesis Defective / Scd1 Protein (Scd1)	0.0062	0.0173	2.52
255432_at	At4g03330	Syntaxin, Putative (Syp123)	0.0042	0.0146	8.78
254465_at	At4g20420	Tapetum-Specific Protein-Related	0.0354	0.0415	3.80
266673_at	At2g29630	Thiamine Biosynthesis Family Protein / Thic Family Protein	0.0055	0.0166	2.78
266463_at	At2g47840	Tic20 Protein-Related	0.0001	0.0028	4.35
249739_at	At5g24520	Transparent Testa Glabra 1 Protein (Ttg1)	0.0443	0.0470	4.61
257221_at	At3g27920	Trichome Differentiation Protein / Glabrous1 Protein (Gl1)	0.0433	0.0464	2.88
256655_at	At3g18890	UV-B and Ozone similiarity regulated protein 1	0.0041	0.0146	4.40
255510_at	At4g02020	Zeste-Like Protein 1 (Eza1)	0.0105	0.0224	3.09
D1. Chloroplast					
250733_at	At5g06290	2-Cys Peroxiredoxin, Chloroplast, Putative	0.0334	0.0402	4.12
263706_s_at	At1g31180	3-Isopropylmalate Dehydrogenase, Chloroplast, Putative	0.0021	0.0105	2.53
260285_at	At1g80560	3-Isopropylmalate Dehydrogenase, Chloroplast, Putative	0.0002	0.0035	3.24
253394_at	At4g32770	Chlorophyll metabolic process	0.0004	0.0052	3.50
263652_at	At1g04330	Chloroplast	0.0238	0.0339	2.73
264728_at	At1g22850	Chloroplast	0.0317	0.0391	3.38
263014_at	At1g23400	Chloroplast	0.0100	0.0219	4.30
262738_at	At1g28530	Chloroplast	0.0103	0.0222	17.05
259818_at	At1g49890	Chloroplast	0.0043	0.0148	2.74
261853_at	At1g50660	Chloroplast	0.0188	0.0300	3.86
260167_at	At1g71970	Chloroplast	0.0386	0.0435	3.46
265596_at	At2g20020	Chloroplast	0.0000	0.0005	5.68
266551_at	At2g35260	Chloroplast	0.0014	0.0089	2.86
266100_at	At2g37980	Chloroplast	0.0088	0.0205	3.98
266128_at	At2g45000	Chloroplast	0.0410	0.0449	4.23
260580_at	At2g47390	Chloroplast	0.0063	0.0175	5.95
258800_at	At3g04550	Chloroplast	0.0054	0.0163	2.64
258802_at	At3g04650	Chloroplast	0.0161	0.0279	5.82
256784_at	At3g13674	Chloroplast	0.0022	0.0108	4.27
258397_at	At3g15357	Chloroplast	0.0223	0.0328	11.29
257717_at	At3g18390	Chloroplast	0.0025	0.0116	3.77
258256_at	At3g26890	Chloroplast	0.0020	0.0104	3.66

251828_at	At3g55070	Chloroplast	0.0054	0.0163	2.50
251741_at	At3g56040	Chloroplast	0.0028	0.0120	2.63
254631_at	At4g18610	Chloroplast	0.0042	0.0146	15.43
254580_at	At4g19390	Chloroplast	0.0471	0.0484	3.15
254522_at	At4g19980	Chloroplast	0.0027	0.0120	3.73
253978_at	At4g26660	Chloroplast	0.0113	0.0233	6.18
253686_at	At4g29750	Chloroplast	0.0061	0.0173	4.03
252979_at	At4g38225	Chloroplast	0.0449	0.0474	2.59
250290_at	At5g13310	Chloroplast	0.0102	0.0221	3.14
250295_at	At5g13390	Chloroplast	0.0430	0.0462	3.10
246849_at	At5g26850	Chloroplast	0.0083	0.0200	2.60
248762_at	At5g47455	Chloroplast	0.0229	0.0332	7.96
247889_at	At5g57930	Chloroplast	0.0051	0.0160	3.53
247770_at	At5g58930	Chloroplast	0.0033	0.0133	2.50
259508_at	At1g43920	Chloroplast	0.0000	0.0008	6.80
246811_at	At5g27170	Chloroplast	0.0280	0.0368	3.16
255474_at	At4g02470	Chloroplast Atpase activity	0.0010	0.0074	3.18
257218_at	At3g15000	Chloroplast differentiation and Palisade development	0.0309	0.0387	3.32
260815_at	At1g06950	Chloroplast Inner Envelope Protein-Related	0.0021	0.0106	3.69
264672_at	At1g09750	Chloroplast Nucleoid Dna-Binding Protein-Related	0.0011	0.0079	4.53
252492_at	At3g46740	Chloroplast Outer Envelope Protein, Putative	0.0080	0.0197	3.61
255482_at	At4g02510	Chloroplast Outer Membrane Protein, Putative	0.0009	0.0073	5.85
265412_at	At2g16640	Chloroplast Outer Membrane Protein, Putative	0.0006	0.0060	26.90
245123_at	At2g47450	Chloroplast Signal Recognition Particle Component (Cao)	0.0091	0.0209	3.32
261488_at	At1g14345	Chloroplast thylakoid membrane	0.0062	0.0174	3.02
249808_at	At5g23890	Chloroplast thylakoid membrane	0.0136	0.0257	3.50
266957_at	At2g34640	Plastid chromosome	0.0000	0.0012	8.11
252441_at	At3g46780	Plastid chromosome	0.0011	0.0080	5.80
266638_at	At2g35490	Plastid-Lipid Associated Protein Pap, Putative	0.0047	0.0154	2.92
250058_at	At5g17870	Plastid-Specific Ribosomal Protein-Related	0.0208	0.0316	3.03
E. Metabolism					
E1. Carbohydrate/soluble sugar/startch/aminoacid metabolism					
250906_at	At5g03650	1,4-Alpha-Glucan Branching Enzyme / Starch Branching Enzyme Class Ii (Sbe2-2)	0.0012	0.0083	2.57
264346_at	At1g12010	1-Aminocyclopropane-1-Carboxylate Oxidase, Putative / Acc Oxidase, Putative	0.0191	0.0302	3.58
260637_at	At1g62380	1-Aminocyclopropane-1-Carboxylate Oxidase, Putative / Acc Oxidase, Putative	0.0008	0.0070	2.62
249866_at	At5g23010	2-Isopropylmalate Synthase 3 (Ims3)	0.0003	0.0046	2.55
261668_at	At1g18500	2-Isopropylmalate Synthase, Putative	0.0114	0.0235	3.39
252325_at	At3g48560	Acetolactate Synthase / Acetohydroxy-Acid Synthase (Als)	0.0011	0.0078	3.19
250111_at	At5g16290	Acetolactate Synthase Small Subunit, Putative	0.0014	0.0088	4.34
260286_at	At1g80600	Acetylornithine Aminotransferase, Mitochondrial, Putative	0.0005	0.0055	3.02
264911_at	At1g60690	Aldo/Keto Reductase Family Protein	0.0011	0.0078	3.03
267168_at	At2g37770	Aldo/Keto Reductase Family Protein	0.0221	0.0325	3.18
250186_at	At5g14500	Aldose 1-Epimerase Family Protein	0.0133	0.0254	3.02
251642_at	At3g57520	Alkaline Alpha Galactosidase, Putative	0.0144	0.0263	4.53
260169_at	At1g71990	Alpha-(1,4)-Fucosyltransferase	0.0077	0.0193	6.61
260412_at	At1g69830	Alpha-Amylase, Putative / 1,4-Alpha-D-Glucan Glucanohydrolase, Putative	0.0335	0.0402	2.62
250336_at	At5g11720	Alpha-Glucosidase 1 (Aglu1)	0.0107	0.0226	4.95
247298_at	At5g63840	Alpha-Glucosidase, Putative	0.0015	0.0092	3.26
262230_at	At1g68560	Alpha-Xylosidase (Xyl1)	0.0202	0.0311	5.52
252967_at	At4g38880	Amidophosphoribosyltransferase, Putative	0.0401	0.0443	2.54
246389_at	At1g77380	Amino Acid Carrier, Putative / Amino Acid Permease, Putative	0.0015	0.0091	8.24
265790_at	At2g01170	Amino Acid Permease Family Protein	0.0001	0.0029	2.65
251133_at	At5g01240	Amino Acid Permease, Putative	0.0495	0.0498	2.80
264394_at	At1g11860	Aminomethyltransferase, Putative	0.0041	0.0146	2.62
253360_at	At4g33090	Aminopeptidase M	0.0078	0.0194	3.71
259296_at	At3g05350	Aminopeptidase P, Cytosolic, Putative	0.0020	0.0105	2.51
247916_at	At5g57590	Aminotransferase Class Iii Family Protein	0.0140	0.0260	6.08
263714_at	At2g20610	Aminotransferase, Putative	0.0306	0.0385	4.41
265965_at	At2g37500	Arginine Biosynthesis Protein Argj Family	0.0000	0.0017	2.67
253203_at	At4g34710	Arginine Decarboxylase 2 (Spe2)	0.0155	0.0274	2.97
250403_at	At5g10920	Argininosuccinate Lyase, Putative / Arginosuccinase, Putative	0.0032	0.0129	2.98
254134_at	At4g24830	Arginosuccinate Synthase Family	0.0004	0.0048	3.35
247218_at	At5g65010	Asparagine Synthetase 2 (Asn2)	0.0000	0.0019	3.66
245951_at	At5g19550	Aspartate Aminotransferase, Cytoplasmic Isozyme 1 / Transaminase A (Asp2)	0.0251	0.0348	2.99
258977_s_at	At3g02020	Aspartate Kinase, Lysine-Sensitive, Putative	0.0262	0.0356	2.99
245346_at	At4g17090	Beta-Amylase (Ct-Bmy) / 1,4-Alpha-D-Glucan Maltohydrolase	0.0143	0.0263	3.88
256861_at	At3g23920	Beta-Amylase, Putative / 1,4-Alpha-D-Glucan Maltohydrolase, Putative	0.0159	0.0277	3.40
262038_at	At1g35580	Beta-Fructofuranosidase, Putative / Invertase, Putative	0.0347	0.0411	2.67
258732_at	At3g05820	Beta-Fructofuranosidase, Putative / Invertase, Putative	0.0161	0.0279	8.86
265118_at	At1g62660	Beta-Fructosidase (Bfruct3) / Beta-Fructofuranosidase / Invertase, Vacuolar	0.0137	0.0257	4.47
260969_at	At1g12240	Beta-Fructosidase (Bfruct4) / Beta-Fructofuranosidase / Invertase, Vacuolar	0.0457	0.0478	3.10
264078_at	At2g28470	Beta-Galactosidase, Putative / Lactase, Putative	0.0145	0.0264	2.60
267556_at	At2g32810	Beta-Galactosidase, Putative / Lactase, Putative	0.0184	0.0297	4.79

256772_at	At3g13750	Beta-Galactosidase, Putative / Lactase, Putative	0.0002	0.0037	2.73
253090_at	At4g36360	Beta-Galactosidase, Putative / Lactase, Putative	0.0299	0.0381	5.31
267377_at	At2g26250	Beta-Ketoacyl-Coa Synthase Family (Fiddlehead) (Fdh)	0.0025	0.0117	4.36
267606_at	At2g26640	Beta-Ketoacyl-Coa Synthase, Putative	0.0041	0.0145	2.82
263696_at	At1g31230	Bifunctional Aspartate Kinase/Homoserine Dehydrogenase / Ak-Hsdh	0.0004	0.0048	4.61
261636_at	At1g50110	Branched-Chain Amino Acid Aminotransferase 6	0.0048	0.0155	6.15
256002_at	At1g29900	Carbamoyl-Phosphate Synthase Family Protein	0.0013	0.0086	4.75
261505_at	At1g71696	Carboxypeptidase D, Putative	0.0002	0.0034	4.42
252886_at	At4g39350	Cellulose Synthase, Catalytic Subunit (Ath-A)	0.0095	0.0213	3.03
250827_at	At5g05170	Cellulose Synthase, Catalytic Subunit (Ath-B)	0.0177	0.0291	3.25
254618_at	At4g18780	Cellulose Synthase, Catalytic Subunit (Irx1)	0.0001	0.0023	12.31
253428_at	At4g32410	Cellulose Synthase, Catalytic Subunit, Putative	0.0189	0.0300	4.05
261957_at	At1g64660	Cys/Met Metabolism Pyridoxal-Phosphate-Dependent Enzyme Family Protein	0.0124	0.0245	8.49
259172_at	At3g03630	Cysteine Synthase, Putative / O-Acetylserine (Thiol)-Lyase, Putative	0.0102	0.0220	8.73
262460_s_at	At1g50390	Fructokinase-Related	0.0041	0.0145	6.26
247278_at	At5g64380	Fructose-1,6-Bisphosphatase Family Protein	0.0091	0.0208	2.77
251885_at	At3g54050	Fructose-1,6-Bisphosphatase, Putative	0.0137	0.0257	2.53
256036_at	At1g07110	Fructose-6-Phosphate 2-Kinase / Fructose-2,6-Bisphosphatase (F2Kp)	0.0173	0.0287	2.78
245060_at	At2g39770	Gdp-Mannose Pyrophosphorylase (Gmp1)	0.0056	0.0167	5.34
256746_at	At3g29320	Glucan Phosphorylase, Putative	0.0492	0.0496	2.85
256098_at	At1g13700	Glucosamine/Galactosamine-6-Phosphate Isomerase Family Protein	0.0003	0.0048	33.29
248687_at	At5g48300	Glucose-1-Phosphate Adenylyltransferase Small Subunit 1 (Aps1)	0.0000	0.0017	3.07
264859_at	At1g24280	Glucose-6-Phosphate 1-Dehydrogenase, Putative / G6Pd, Putative	0.0050	0.0158	2.52
254141_at	At4g24620	Glucose-6-Phosphate Isomerase, Putative	0.0004	0.0051	2.83
246445_at	At5g17630	Glucose-6-Phosphate/Phosphate Translocator, Putative	0.0136	0.0257	3.55
251746_at	At3g56060	Glucose-Methanol-Choline (Gmc) Oxidoreductase Family Protein	0.0146	0.0265	2.64
245701_at	At5g04140	Glutamate Synthase (Glu1) / Ferredoxin-Dependent Glutamate Synthase (Fd-Gogat 1)	0.0007	0.0068	2.90
248267_at	At5g53460	Glutamate Synthase (Nadh), Chloroplast, Putative	0.0000	0.0003	5.32
266365_at	At2g41220	Glutamate Synthase, Chloroplast (Glu2)	0.0006	0.0061	4.57
258160_at	At3g17820	Glutamine Synthetase (Gs1)	0.0077	0.0193	4.01
251860_at	At3g54660	Gluthatione Reductase, Chloroplast	0.0362	0.0420	2.76
258588_s_at	At3g04120	Glyceraldehyde-3-Phosphate Dehydrogenase, Cytosolic (Gapc)	0.0167	0.0282	3.52
260014_at	At1g68010	Glycerate Dehydrogenase / Nadh-Dependent Hydroxypyruvate Reductase	0.0002	0.0037	2.53
260274_at	At1g80460	Glycerol Kinase, Putative	0.0101	0.0219	2.95
266892_at	At2g26080	Glycine Dehydrogenase (Decarboxylating), Putative	0.0153	0.0273	6.53
253387_at	At4g33010	Glycine Dehydrogenase (Decarboxylating), Putative	0.0020	0.0104	4.58
254740_s_at	At4g13890	Glycine Hydroxymethyltransferase, Putative	0.0295	0.0378	3.30
258359_s_at	At3g14415	Glycolate Oxidase, Putative	0.0186	0.0299	2.79
255814_at	At1g19900	Glyoxal Oxidase-Related	0.0001	0.0027	3.98
262972_at	At1g75620	Glyoxal Oxidase-Related	0.0352	0.0414	2.54
251175_at	At3g63250	Homocysteine S-Methyltransferase 2 (Hmt-2)	0.0078	0.0193	3.56
264360_at	At1g03310	Isoamylase, Putative / Starch Debranching Enzyme, Putative	0.0053	0.0163	2.99
250197_at	At5g14590	Isocitrate Dehydrogenase, Putative / Nadp+ Isocitrate Dehydrogenase, Putative	0.0013	0.0084	3.50
250770_at	At5g05390	Laccase, Putative / Diphenol Oxidase, Putative	0.0316	0.0390	8.23
264372_at	At1g11840	Lactoylglutathione Lyase, Putative / Glyoxalase I, Putative	0.0014	0.0089	2.62
252376_at	At3g47930	L-Galactono-1,4-Lactone Dehydrogenase, Putative	0.0005	0.0055	2.78
245324_at	At4g17260	L-Lactate Dehydrogenase, Putative	0.0138	0.0257	3.40
263663_at	At1g04410	Malate Dehydrogenase, Cytosolic, Putative	0.0153	0.0273	2.53
261355_at	At1g79750	Malate Oxidoreductase, Putative	0.0063	0.0174	2.97
248576_at	At5g49810	Methionine S-Methyltransferase	0.0380	0.0432	2.78
258416_at	At3g17310	Methyltransferase Family Protein	0.0056	0.0167	22.19
253585_at	At4g30720	Monooxygenase	0.0005	0.0056	5.18
258218_at	At3g18000	Phosphoethanolamine N-Methyltransferase 1 / Peamt 1 (Nmt1)	0.0186	0.0299	2.73
259842_at	At1g73600	Phosphoethanolamine N-Methyltransferase 3, Putative (Nmt3)	0.0000	0.0004	2.88
249411_at	At5g40390	Raffinose Synthase Family Protein	0.0081	0.0198	2.55
249827_at	At5g23330	Riboflavin Biosynthesis Protein-Related	0.0004	0.0051	9.31
263882_at	At2g21790	Ribonucleoside-Diphosphate Reductase Small Chain, Putative	0.0009	0.0072	2.70
267365_at	At2g44530	Ribose-Phosphate Pyrophosphokinase, Putative	0.0022	0.0107	2.70
245061_at	At2g39730	Ribulose Bisphosphate Carboxylase/Oxygenase Activase / Rubisco Activase	0.0160	0.0278	4.11
249599_at	At5g37990	S-Adenosyl-L-Methionine:Carboxyl Methyltransferase Family Protein	0.0126	0.0247	7.71
256375_at	At1g66720	S-Adenosyl-L-Methionine:Carboxyl Methyltransferase Family Protein	0.0194	0.0304	6.49
260913_at	At1g02500	S-Adenosylmethionine Synthetase 1 (Sam1)	0.0410	0.0449	7.35
255552_at	At4g01850	S-Adenosylmethionine Synthetase 2 (Sam2)	0.0025	0.0115	2.82
249854_at	At5g22960	Serine Carboxypeptidase S10 Family Protein	0.0132	0.0253	14.41
252468_at	At3g46970	Starch Phosphorylase, Putative	0.0331	0.0399	3.10
249785_at	At5g24300	Starch Synthase, Putative	0.0072	0.0187	5.67
254659_at	At4g18240	Starch Synthase-Related Protein	0.0457	0.0478	12.97
245998_at	At5g20830	Sucrose Synthase / Sucrose-Udp Glucosyltransferase (Sus1)	0.0037	0.0139	4.16
260517_at	At1g51420	Sucrose-Phosphatase, Putative	0.0055	0.0165	11.14
255016_at	At4g10120	Sucrose-Phosphate Synthase, Putative	0.0009	0.0072	4.10
246076_at	At5g20280	Sucrose-Phosphate Synthase, Putative	0.0025	0.0115	4.01
253700_at	At4g29840	Threonine Synthase, Chloroplast	0.0149	0.0269	4.95
262380_at	At1g72810	Threonine Synthase, Putative	0.0018	0.0100	3.83
260059_at	At1g78090	Trehalose-6-Phosphate Phosphatase (Tppb)	0.0049	0.0157	3.94

249515_at	At5g38530	Tryptophan Synthase-Related	0.0004	0.0050	5.85
256745_at	At3g29360	UDP-Glucose 6-Dehydrogenase, Putative	0.0485	0.0493	2.74
246511_at	At5g15490	UDP-Glucose 6-Dehydrogenase, Putative	0.0279	0.0367	5.87
258556_at	At3g07020	UDP-Glucose:Sterol Glucosyltransferase (Ugt80A2)	0.0064	0.0177	2.93
253386_at	At4g33030	UDP-Sulfoquinovose Synthase / Sulfite:Udp-Glucose Sulfotransferase	0.0087	0.0203	2.80
250074_at	At5g17310	UTP--Glucose-1-Phosphate Uridylyltransferase, Putative	0.0054	0.0164	3.02
258849_at	At3g03250	UTP--Glucose-1-Phosphate Uridylyltransferase, Putative	0.0329	0.0398	3.41
263773_at	At2g21370	Xylulose Kinase, Putative	0.0410	0.0449	4.77
E2. Primary /secondary metabolism					
247637_at	At5g60600	1-Hydroxy-2-Methyl-2-(E)-Butenyl 4-Diphosphate Synthase, Putative	0.0108	0.0227	3.28
264668_at	At1g09780	2,3-Biphosphoglycerate-Independent Phosphoglycerate Mutase, Putative	0.0000	0.0008	2.87
258679_at	At3g08590	2,3-Biphosphoglycerate-Independent Phosphoglycerate Mutase, Putative	0.0018	0.0098	3.72
253333_at	At4g33510	2-Dehydro-3-Deoxyphosphoheptonate Aldolase 2	0.0023	0.0110	3.19
262638_at	At1g06650	2-Oxoglutarate-Dependent Dioxygenase, Putative	0.0087	0.0204	2.63
255471_at	At4g03050	2-Oxoglutarate-Dependent Dioxygenase, Putative (Aop3)	0.0286	0.0372	7.06
257052_at	At3g15290	3-Hydroxybutyryl-Coa Dehydrogenase, Putative	0.0154	0.0273	2.88
248903_at	At5g46290	3-Oxoacyl-(Acyl-Carrier-Protein) Synthase I	0.0044	0.0150	3.47
258964_at	At3g10540	3-Phosphoinositide-Dependent Protein Kinase, Putative	0.0171	0.0286	8.24
247216_at	At5g64860	4-Alpha-Glucanotransferase, Putative / Disproportionating Enzyme, Putative	0.0437	0.0466	2.69
256186_at	At1g51680	4-Coumarate--Coa Ligase 1 / 4-Coumaroyl-Coa Synthase 1 (4Cl1)	0.0052	0.0162	3.66
266574_at	At2g23890	5' Nucleotidase Family Protein	0.0116	0.0237	5.22
249200_at	At5g42540	5'-3' Exoribonuclease (Xrn2)	0.0008	0.0069	3.98
255284_at	At4g04610	5'-Adenylylsulfate Reductase (Apr1) / Paps Reductase Homolog (Prh19)	0.0018	0.0098	3.33
259343_s_at	At3g03780	5-Methyltetrahydropteroyltriglutamate--Homocysteine Methyltransferase, Putative	0.0439	0.0467	6.92
256328_at	At3g02360	6-Phosphogluconate Dehydrogenase Family Protein	0.0058	0.0170	2.52
262130_at	At1g02890	Aaa-Type Atpase Family Protein	0.0283	0.0370	3.37
262347_at	At1g64110	Aaa-Type Atpase Family Protein	0.0005	0.0054	3.27
265340_at	At2g18330	Aaa-Type Atpase Family Protein	0.0053	0.0163	2.86
257046_at	At3g19740	Aaa-Type Atpase Family Protein	0.0182	0.0295	3.53
256593_at	At3g28510	Aaa-Type Atpase Family Protein	0.0276	0.0365	7.80
257564_at	At3g28610	Aaa-Type Atpase Family Protein	0.0035	0.0136	8.50
252156_at	At3g50940	Aaa-Type Atpase Family Protein	0.0240	0.0340	4.99
254136_at	At4g24860	Aaa-Type Atpase Family Protein	0.0286	0.0372	6.01
246461_at	At5g16930	Aaa-Type Atpase Family Protein	0.0015	0.0091	13.77
266099_at	At2g38040	Acetyl Co-Enzyme A Carboxylase Carboxyltransferase Alpha Subunit Family	0.0008	0.0071	5.46
263192_at	At1g36160	Acetyl-Coa Carboxylase 1 (Acc1)	0.0361	0.0419	8.90
253135_at	At4g35830	Aconitate Hydratase, Cytoplasmic / Citrate Hydro-Lyase / Aconitase (Aco)	0.0064	0.0176	2.72
263348_at	At2g05710	Aconitate Hydratase, Cytoplasmic, Putative	0.0285	0.0371	2.82
253954_at	At4g26970	Aconitate Hydratase, Cytoplasmic, Putative	0.0177	0.0291	4.57
260570_at	At2g43710	Acyl-(Acyl-Carrier-Protein) Desaturase / Stearoyl-Acp Desaturase (Ssi2)	0.0046	0.0153	3.29
259647_at	At1g55320	Acyl-Activating Enzyme 18 (Aae18)	0.0276	0.0365	6.44
259159_at	At3g05420	Acyl-Coa Binding Family Protein	0.0061	0.0173	2.91
253840_at	At4g27780	Acyl-Coa Binding Protein 2 (Acbp2)	0.0234	0.0336	4.24
258524_at	At3g06810	Acyl-Coa Dehydrogenase-Related	0.0033	0.0132	3.89
245249_at	At4g16760	Acyl-Coa Oxidase (Acx1)	0.0394	0.0440	3.72
260789_s_at	At1g06290	Acyl-Coa Oxidase (Acx3)	0.0129	0.0251	2.81
265843_at	At2g35690	Acyl-Coa Oxidase, Putative	0.0009	0.0074	2.62
249972_at	At5g19040	Adenylate Isopentenyltransferase 5 / Cytokinin Synthase (Ipt5)	0.0332	0.0400	3.78
255741_at	At1g25410	Adenylate Isopentenyltransferase 6 / Adenylate Dimethylallyltransferase	0.0170	0.0285	3.49
260250_at	At1g74260	Air Synthase-Related Family Protein	0.0000	0.0019	8.05
258983_at	At3g08860	Alanine--Glyoxylate Aminotransferase, Putative	0.0143	0.0263	3.64
264953_at	At1g77120	Alcohol Dehydrogenase (Adh)	0.0027	0.0119	5.94
254572_at	At4g19380	Alcohol Oxidase-Related	0.0031	0.0128	7.89
265188_at	At1g23800	Aldehyde Dehydrogenase, Mitochondrial (Aldh3)	0.0445	0.0471	11.08
267095_at	At2g38280	Amp Deaminase, Putative / Myoadenylate Deaminase, Putative	0.0135	0.0256	3.45
259545_at	At1g20560	Amp-Dependent Synthetase And Ligase Family Protein	0.0013	0.0087	2.65
264589_at	At2g17650	Amp-Dependent Synthetase And Ligase Family Protein	0.0000	0.0007	24.06
257880_at	At3g16910	Amp-Dependent Synthetase And Ligase Family Protein	0.0067	0.0180	2.85
259334_at	At3g03790	Ankyrin Repeat Family Protein	0.0008	0.0071	5.92
266671_at	At2g29690	Anthranilate Synthase, Alpha Subunit, Component I-2 (Asa2)	0.0352	0.0414	3.72
252391_at	At3g47860	Apolipoprotein D-Related	0.0025	0.0116	3.05
254891_at	At4g11740	Ara4-Interacting Protein, Putative (Say1)	0.0016	0.0094	2.82
258053_at	At3g16230	ASC-1 complex subunit P50	0.0241	0.0341	2.54
245867_at	At1g58080	Atp Phosphoribosyl Transferase 1 (Atp-Prt1)	0.0102	0.0220	3.28
258515_at	At3g06650	Atp-Citrate Synthase, Putative / Atp-Citrate (Pro-S-)-Lyase, Putative	0.0422	0.0456	5.26
247494_at	At5g61790	Calnexin 1 (Cnx1)	0.0021	0.0105	3.87
256216_at	At1g56340	Calreticulin 1 (Crt1)	0.0023	0.0111	3.28
264260_at	At1g09210	Calreticulin 2 (Crt2)	0.0000	0.0005	2.71
262751_at	At1g16310	Cation Efflux Family Protein	0.0089	0.0206	9.72
262940_at	At1g79520	Cation Efflux Family Protein	0.0392	0.0438	3.74
264146_at	At1g02205	Cer1 Protein	0.0043	0.0148	7.04
264175_at	At1g02050	Chalcone And Stilbene Synthase Family Protein	0.0123	0.0244	4.13

250207_at	At5g13930	Chalcone Synthase / Naringenin-Chalcone Synthase	0.0004	0.0048	9.13
246601_at	At1g31710	Copper Amine Oxidase, Putative	0.0096	0.0214	3.87
265882_at	At2g42490	Copper Amine Oxidase, Putative	0.0495	0.0498	3.41
255402_at	At4g03205	Coproporphyrinogen Iii Oxidase, Putative / Coproporphyrinogenase, Putative	0.0297	0.0379	2.62
255068_at	At4g08920	Cryptochrome 1 Apoprotein (Cry1) / Flavin-Type Blue-Light Photoreceptor (Hy4)	0.0132	0.0253	4.93
257702_at	At3g12670	Ctp Synthase, Putative / Utp--Ammonia Ligase, Putative	0.0004	0.0050	3.05
253273_at	At4g34180	Cyclase Family Protein	0.0075	0.0189	2.63
266045_s_at	At2g07727	Cytochrome B (Mtcyb) (Cob) (Cytb)	0.0141	0.0261	3.28
262591_at	At1g15220	Cytochrome C Biogenesis Protein Family	0.0415	0.0452	6.59
265227_s_at	At2g07695	Cytochrome C Oxidase Subunit Ii, Putative	0.0082	0.0199	2.87
262780_at	At1g13090	Cytochrome P450 71B28, Putative (Cyp71B28)	0.0003	0.0046	2.59
252827_at	At4g39950	Cytochrome P450 79B2, Putative (Cyp79B2)	0.0338	0.0405	2.69
253088_at	At4g36220	Cytochrome P450 84A1 (Cyp84A1) / Ferulate-5-Hydroxylase (Fah1)	0.0084	0.0201	5.48
247765_at	At5g58860	Cytochrome P450 86A1 (Cyp86) (Cyp86A1)	0.0063	0.0175	7.63
266996_at	At2g34490	Cytochrome P450 Family Protein	0.0282	0.0370	3.55
257129_at	At3g20100	Cytochrome P450 Family Protein	0.0031	0.0128	6.96
256875_at	At3g26330	Cytochrome P450 Family Protein	0.0100	0.0219	5.01
253886_at	At4g27710	Cytochrome P450 Family Protein	0.0015	0.0091	4.37
246978_at	At5g24910	Cytochrome P450 Family Protein	0.0446	0.0472	4.47
249684_s_at	At5g36110	Cytochrome P450 Family Protein	0.0072	0.0186	6.94
262717_s_at	At1g16410	Cytochrome P450, Putative	0.0043	0.0149	5.59
260599_at	At1g55940	Cytochrome P450, Putative	0.0000	0.0015	11.73
258962_at	At3g10570	Cytochrome P450, Putative	0.0126	0.0247	10.97
256788_at	At3g13730	Cytochrome P450, Putative	0.0113	0.0234	3.95
258113_at	At3g14650	Cytochrome P450, Putative	0.0279	0.0367	4.03
253502_at	At4g31940	Cytochrome P450, Putative	0.0018	0.0099	17.43
253073_at	At4g37410	Cytochrome P450, Putative	0.0009	0.0071	3.67
245710_at	At5g04330	Cytochrome P450, Putative / Ferulate-5-Hydroxylase, Putative	0.0198	0.0308	2.84
265996_at	At2g24200	Cytosol Aminopeptidase	0.0031	0.0128	3.35
259403_at	At1g17745	D-3-Phosphoglycerate Dehydrogenase / 3-Pgdh	0.0212	0.0319	2.73
253274_at	At4g34200	D-3-Phosphoglycerate Dehydrogenase, Putative / 3-Pgdh, Putative	0.0202	0.0311	4.25
256862_at	At3g23940	Dehydratase Family	0.0186	0.0299	3.02
251775_s_at	At3g55610	Delta 1-Pyrroline-5-Carboxylate Synthetase B / P5Cs B (P5Cs2)	0.0170	0.0285	10.02
247436_at	At5g62530	Delta-1-Pyrroline-5-Carboxylate Dehydrogenase (P5Cdh)	0.0188	0.0300	2.59
247323_at	At5g64170	Dentin Sialophosphoprotein-Related	0.0017	0.0096	3.63
256341_at	At1g72040	Deoxynucleoside Kinase Family	0.0166	0.0282	3.84
249840_at	At5g23450	Diacylglycerol Kinase Family Protein	0.0269	0.0361	2.57
259070_at	At3g11670	Digalactosyldiacylglycerol Synthase 1 (Dgd1) / Mgdg:Mgdg Galactosyltransferase	0.0085	0.0201	3.11
251392_at	At3g60880	Dihydrodipicolinate Synthase 1 (Dhdps1) (Dhdps) (Dhps1)	0.0000	0.0017	2.53
257895_at	At3g16950	Dihydrolipoamide Dehydrogenase 1, Plastidic / Lipoamide Dehydrogenase 1 (Ptlpd1)	0.0279	0.0367	4.43
258439_at	At3g17240	Dihydrolipoamide Dehydrogenase 2, Mitochondrial	0.0004	0.0051	4.18
256682_at	At3g52200	Dihydrolipoamide S-Acetyltransferase, Putative	0.0049	0.0156	3.08
257866_at	At3g17770	Dihydroxyacetone Kinase Family Protein	0.0009	0.0072	4.52
251474_at	At3g59630	Diphthamide Synthesis Dph2 Family Protein	0.0051	0.0160	3.20
264683_at	At1g65580	Endonuclease/Exonuclease/Phosphatase Family Protein	0.0262	0.0356	2.51
251581_at	At3g58560	Endonuclease/Exonuclease/Phosphatase Family Protein	0.0451	0.0475	2.69
251582_at	At3g58580	Endonuclease/Exonuclease/Phosphatase Family Protein	0.0146	0.0266	2.56
250359_at	At5g11350	Endonuclease/Exonuclease/Phosphatase Family Protein	0.0069	0.0182	16.70
259322_at	At3g05270	Endosome-associated protein (EEA1)	0.0082	0.0199	3.71
263924_at	At2g36530	Enolase	0.0007	0.0068	3.41
262619_at	At1g06550	Enoyl-Coa Hydratase/Isomerase Family Protein	0.0012	0.0084	4.36
253469_at	At4g32180	Eukaryotic Pantothenate Kinase Family Protein	0.0148	0.0268	4.53
256858_at	At3g15140	Exonuclease Family Protein	0.0143	0.0262	4.07
264357_at	At1g03360	Exonuclease Family Protein	0.0045	0.0151	2.83
247470_at	At5g62220	Exostosin Family Protein	0.0128	0.0249	2.72
249885_at	At5g22940	Exostosin Family Protein	0.0299	0.0381	2.50
265475_at	At2g15620	Ferredoxin--Nitrite Reductase, Putative	0.0168	0.0283	4.04
265122_at	At1g62540	Flavin-Containing Monooxygenase Family Protein / Fmo Family Protein	0.0330	0.0399	3.38
265121_at	At1g62560	Flavin-Containing Monooxygenase Family Protein / Fmo Family Protein	0.0000	0.0006	2.54
265119_at	At1g62570	Flavin-Containing Monooxygenase Family Protein / Fmo Family Protein	0.0485	0.0493	3.57
253794_at	At4g28720	Flavin-Containing Monooxygenase Family Protein / Fmo Family Protein	0.0110	0.0230	3.67
259126_at	At3g02280	Flavodoxin Family Protein	0.0008	0.0069	3.66
247333_at	At5g63600	Flavonol Synthase, Putative	0.0164	0.0280	10.39
253042_at	At4g37550	Formamidase, Putative / Formamide Amidohydrolase, Putative	0.0001	0.0028	4.41
253048_at	At4g37560	Formamidase, Putative / Formamide Amidohydrolase, Putative	0.0086	0.0203	3.51
262129_at	At1g52500	Formamidopyrimidine-Dna Glycolase Family Protein / Mutm, Putative (Mmh-1)	0.0259	0.0354	3.35
246595_at	At5g14780	Formate Dehydrogenase (Fdh)	0.0112	0.0232	3.64
245417_at	At4g17360	Formyltetrahydrofolate Deformylase, Putative	0.0000	0.0002	6.62
250227_at	At5g13830	Ftsj-Like Methyltransferase Family Protein	0.0423	0.0456	3.35
255956_at	At1g22015	Galactosyltransferase Family Protein	0.0106	0.0225	3.98
266537_at	At2g16860	Gcip-Interacting Family Protein	0.0000	0.0014	3.83
266189_at	At2g39020	Gcn5-Related N-Acetyltransferase (Gnat) Family Protein	0.0462	0.0480	2.82
266142_at	At2g39030	Gcn5-Related N-Acetyltransferase (Gnat) Family Protein	0.0033	0.0132	3.91
252996_s_at	At4g38460	Geranylgeranyl Pyrophosphate Synthase, Putative	0.0330	0.0399	4.22

254065_at	At4g25420	Gibberellin 20-Oxidase	0.0139	0.0258	6.12
261768_at	At1g15550	Gibberellin 3-Beta-Dioxygenase / Gibberellin 3 Beta-Hydroxylase (Ga4)	0.0062	0.0174	2.92
264211_at	At1g22770	Gigantea Protein (Gi)	0.0358	0.0418	3.90
264855_at	At2g17265	Homoserine Kinase (Hsk)	0.0240	0.0340	3.52
249585_at	At5g37830	Hydantoinase/Oxoprolinase Family Protein	0.0197	0.0307	3.36
254076_at	At4g25340	Immunophilin-Related / Fkbp-Type Peptidyl-Prolyl Cis-Trans Isomerase-Related	0.0420	0.0455	3.38
258531_at	At3g06720	Importin Alpha-1 Subunit, Putative (Impa1)	0.0102	0.0221	2.90
245844_at	At1g26170	Importin Beta-2 Subunit Family Protein	0.0219	0.0323	3.56
251495_at	At3g59020	Importin Beta-2 Subunit Family Protein	0.0035	0.0135	4.14
266523_at	At2g16950	Importin Beta-2 Subunit Family Protein	0.0215	0.0321	2.81
261208_at	At1g12930	Importin-Related	0.0335	0.0402	2.65
265339_at	At2g18230	Inorganic Pyrophosphatase (Soluble) (Ppa)	0.0084	0.0201	3.28
262754_at	At1g16350	Inosine-5'-Monophosphate Dehydrogenase, Putative	0.0329	0.0399	2.92
261522_at	At1g71710	Inositol Polyphosphate 5-Phosphatase, Putative	0.0002	0.0037	2.71
252863_at	At4g39800	Inositol-3-Phosphate Synthase Isozyme 1	0.0069	0.0183	3.30
253392_at	At4g32650	Inward Rectifying Potassium Channel, Putative (Kat3) (Akt4) (Kc1)	0.0407	0.0448	2.55
263083_at	At2g27190	Iron(Iii)-Zinc(Ii) Purple Acid Phosphatase (Pap12)	0.0313	0.0389	2.65
254547_at	At4g19860	Lecithin:Cholesterol Acyltransferase Family Protein / Lact Family Protein	0.0061	0.0173	4.04
260190_at	At1g67560	Lipoxygenase Family Protein	0.0001	0.0030	3.86
259761_at	At1g77590	Long-Chain-Fatty-Acid--Coa Ligase Family Protein	0.0007	0.0064	2.67
264100_at	At1g78970	Lupeol Synthase (Lup1) / 2,3-Oxidosqualene-Triterpenoid Cyclase	0.0019	0.0101	2.78
250243_at	At5g13630	Magnesium-Chelatase Subunit Chlh, Chloroplast, Putative	0.0009	0.0073	5.94
249218_at	At5g42390	Metalloendopeptidase	0.0000	0.0002	3.08
263707_at	At1g09300	Metallopeptidase M24 Family Protein	0.0455	0.0477	3.65
259378_at	At3g16310	Mitotic Phosphoprotein N' End (Mppn) Family Protein	0.0026	0.0118	2.82
253733_at	At4g29170	Mnd1 Family Protein	0.0410	0.0449	7.43
260325_at	At1g63940	Monodehydroascorbate Reductase, Putative	0.0168	0.0283	5.24
264752_at	At1g23010	Multi-Copper Oxidase Type I Family Protein	0.0001	0.0026	2.59
264954_at	At1g77060	Mutase Family Protein	0.0277	0.0366	3.06
261354_at	At1g79690	Mutt/Nudix Family Protein	0.0110	0.0230	3.13
263153_s_at	At1g54010	Myrosinase-Associated Protein, Putative	0.0035	0.0135	5.50
260985_at	At1g53500	Nad-Dependent Epimerase/Dehydratase Family Protein	0.0051	0.0160	3.37
263134_at	At1g78570	Nad-Dependent Epimerase/Dehydratase Family Protein	0.0044	0.0150	3.41
256575_at	At3g14790	Nad-Dependent Epimerase/Dehydratase Family Protein	0.0002	0.0033	3.17
252123_at	At3g51240	Naringenin 3-Dioxygenase / Flavanone 3-Hydroxylase (F3H)	0.0011	0.0078	4.59
265398_at	At2g11000	Natc N(Alpha)-Terminal Acetyltransferase, Mak10 Subunit Family Protein	0.0014	0.0090	2.91
259681_at	At1g77760	Nitrate Reductase 1 (Nr1)	0.0185	0.0299	10.65
261979_at	At1g37130	Nitrate Reductase 2 (Nr2)	0.0143	0.0263	3.09
264169_at	At1g02020	Nitroreductase Family Protein	0.0063	0.0175	3.81
267513_at	At2g45620	Nucleotidyltransferase Family Protein	0.0119	0.0240	2.99
258575_at	At3g04240	O-Linked N-Acetyl Glucosamine Transferase, Putative	0.0346	0.0410	2.86
253547_at	At4g30950	Omega-6 Fatty Acid Desaturase, Chloroplast (Fad6) (Fadc)	0.0099	0.0218	3.78
260164_at	At1g79870	Oxidoreductase Family Protein	0.0071	0.0185	4.01
262572_at	At1g15140	Oxidoreductase Protein	0.0093	0.0211	2.78
262527_at	At1g17010	Oxidoreductase, 2Og-Fe(Ii) Oxygenase Family Protein	0.0188	0.0300	3.39
262482_at	At1g17020	Oxidoreductase, 2Og-Fe(Ii) Oxygenase Family Protein	0.0155	0.0274	12.32
257135_at	At3g12900	Oxidoreductase, 2Og-Fe(Ii) Oxygenase Family Protein	0.0325	0.0397	5.29
256892_at	At3g19000	Oxidoreductase, 2Og-Fe(Ii) Oxygenase Family Protein	0.0001	0.0027	3.44
252530_at	At3g46500	Oxidoreductase, 2Og-Fe(Ii) Oxygenase Family Protein	0.0065	0.0177	3.53
245204_at	At5g12270	Oxidoreductase, 2Og-Fe(Ii) Oxygenase Family Protein	0.0014	0.0087	2.94
264283_at	At1g61850	Patatin Family Protein	0.0010	0.0074	13.41
246252_s_at	At4g37070	Patatin, Putative	0.0086	0.0203	3.99
251174_at	At3g63200	Patatin-Related	0.0027	0.0119	2.89
264611_at	At1g04680	Pectate Lyase Family Protein	0.0091	0.0209	3.84
247377_at	At5g63180	Pectate Lyase Family Protein	0.0031	0.0128	3.31
264177_at	At1g02150	Pentatricopeptide (Ppr) Repeat-Containing Protein	0.0009	0.0073	2.52
256107_at	At1g16830	Pentatricopeptide (Ppr) Repeat-Containing Protein	0.0005	0.0055	4.09
261133_at	At1g19720	Pentatricopeptide (Ppr) Repeat-Containing Protein	0.0022	0.0107	3.23
263220_at	At1g30610	Pentatricopeptide (Ppr) Repeat-Containing Protein	0.0001	0.0025	4.35
259622_at	At1g43010	Pentatricopeptide (Ppr) Repeat-Containing Protein	0.0031	0.0129	6.64
260595_at	At1g55890	Pentatricopeptide (Ppr) Repeat-Containing Protein	0.0082	0.0198	4.44
246414_at	At1g77340	Pentatricopeptide (Ppr) Repeat-Containing Protein	0.0125	0.0246	10.48
262941_at	At1g79490	Pentatricopeptide (Ppr) Repeat-Containing Protein	0.0013	0.0087	3.36
266114_at	At2g02150	Pentatricopeptide (Ppr) Repeat-Containing Protein	0.0034	0.0135	6.20
265474_at	At2g15690	Pentatricopeptide (Ppr) Repeat-Containing Protein	0.0099	0.0218	3.78
265484_at	At2g15820	Pentatricopeptide (Ppr) Repeat-Containing Protein	0.0059	0.0170	3.78
266951_at	At2g18940	Pentatricopeptide (Ppr) Repeat-Containing Protein	0.0008	0.0070	32.46
266164_at	At2g28050	Pentatricopeptide (Ppr) Repeat-Containing Protein	0.0011	0.0080	3.21
267086_at	At2g32630	Pentatricopeptide (Ppr) Repeat-Containing Protein	0.0305	0.0384	3.93
267445_at	At2g33680	Pentatricopeptide (Ppr) Repeat-Containing Protein	0.0059	0.0170	10.62
256325_at	At3g02330	Pentatricopeptide (Ppr) Repeat-Containing Protein	0.0073	0.0187	4.74
258355_at	At3g14330	Pentatricopeptide (Ppr) Repeat-Containing Protein	0.0001	0.0030	3.31
252216_at	At3g50420	Pentatricopeptide (Ppr) Repeat-Containing Protein	0.0266	0.0358	4.93

251496_at	At3g59040	Pentatricopeptide (Ppr) Repeat-Containing Protein	0.0126	0.0247	2.50
255573_at	At4g01400	Pentatricopeptide (Ppr) Repeat-Containing Protein	0.0003	0.0042	3.75
254725_at	At4g13650	Pentatricopeptide (Ppr) Repeat-Containing Protein	0.0006	0.0058	7.84
245498_at	At4g16470	Pentatricopeptide (Ppr) Repeat-Containing Protein	0.0177	0.0291	2.96
253979_at	At4g26680	Pentatricopeptide (Ppr) Repeat-Containing Protein	0.0000	0.0017	3.34
253116_at	At4g35850	Pentatricopeptide (Ppr) Repeat-Containing Protein	0.0002	0.0033	2.51
250856_at	At5g04810	Pentatricopeptide (Ppr) Repeat-Containing Protein	0.0012	0.0084	3.25
250303_at	At5g12100	Pentatricopeptide (Ppr) Repeat-Containing Protein	0.0155	0.0274	3.23
250257_at	At5g13770	Pentatricopeptide (Ppr) Repeat-Containing Protein	0.0269	0.0360	2.80
246173_s_at	At5g28370	Pentatricopeptide (Ppr) Repeat-Containing Protein	0.0091	0.0208	3.27
246174_s_at	At5g28380	Pentatricopeptide (Ppr) Repeat-Containing Protein	0.0485	0.0493	8.71
249247_at	At5g42310	Pentatricopeptide (Ppr) Repeat-Containing Protein	0.0008	0.0069	4.72
249088_at	At5g44230	Pentatricopeptide (Ppr) Repeat-Containing Protein	0.0066	0.0179	7.58
247099_at	At5g66500	Pentatricopeptide (Ppr) Repeat-Containing Protein	0.0418	0.0454	4.28
260295_at	At1g63770	Peptidase M1 Family Protein	0.0032	0.0129	2.77
250441_at	At5g10540	Peptidase M3 Family Protein / Thimet Oligopeptidase Family Protein	0.0256	0.0352	2.87
247152_at	At5g65620	Peptidase M3 Family Protein / Thimet Oligopeptidase Family Protein	0.0361	0.0419	2.57
259763_at	At1g77630	Peptidoglycan-Binding Lysm Domain-Containing Protein	0.0021	0.0105	2.97
257822_at	At3g25230	Peptidyl-Prolyl Cis-Trans Isomerase / Fk506-Binding Protein (Rof1)	0.0000	0.0007	2.51
248657_at	At5g48570	Peptidyl-Prolyl Cis-Trans Isomerase, Putative / Fk506-Binding Protein, Putative	0.0001	0.0031	3.88
251932_at	At3g54010	Peptidyl-Prolyl Cis-Trans Isomerase, Putative / Fk506-Binding Protein, Putative	0.0075	0.0189	2.70
250926_at	At5g03555	Permease, Cytosine/Purines, Uracil, Thiamine, Allantoin Family Protein	0.0258	0.0353	3.73
261518_at	At1g71695	Peroxidase 12 (Per12) (P12) (Prxr6)	0.0053	0.0163	3.68
254386_at	At4g21960	Peroxidase 42 (Per42) (P42) (Prxr1)	0.0315	0.0390	3.90
267053_s_at	At2g38390	Peroxidase, Putative	0.0017	0.0095	7.00
252138_at	At3g50990	Peroxidase, Putative	0.0192	0.0303	2.99
245821_at	At1g26270	Phosphatidylinositol 3- And 4-Kinase Family Protein	0.0065	0.0178	2.91
262387_s_at	At1g49340	Phosphatidylinositol 3- And 4-Kinase Family Protein	0.0010	0.0076	3.49
260466_at	At1g10900	Phosphatidylinositol-4-Phosphate 5-Kinase Family Protein	0.0331	0.0399	3.96
258361_at	At3g14270	Phosphatidylinositol-4-Phosphate 5-Kinase Family Protein	0.0233	0.0335	2.60
253371_at	At4g33240	Phosphatidylinositol-4-Phosphate 5-Kinase Family Protein	0.0035	0.0135	8.32
257217_at	At3g14940	Phosphoenolpyruvate Carboxylase, Putative / Pep Carboxylase, Putative	0.0216	0.0321	2.76
260590_at	At1g53310	Phosphoenolpyruvate Carboxylase, Putative / Pep Carboxylase, Putative (Ppc1)	0.0304	0.0384	6.11
263491_at	At2g42600	Phosphoenolpyruvate Carboxylase, Putative / Pep Carboxylase, Putative (Ppc2)	0.0048	0.0155	5.26
256041_at	At1g07230	Phosphoesterase Family Protein	0.0096	0.0215	3.17
264903_at	At1g23190	Phosphoglucomutase, Cytoplasmic, Putative / Glucose Phosphomutase, Putative	0.0007	0.0068	3.38
249899_at	At5g22620	Phosphoglycerate/Bisphosphoglycerate Mutase Family Protein	0.0058	0.0169	2.97
261060_at	At1g17340	Phosphoinositide Phosphatase Family Protein	0.0168	0.0283	3.15
255720_at	At1g32060	Phosphoribulokinase (Prk) / Phosphopentokinase	0.0111	0.0231	4.77
255067_at	At4g08960	Phosphotyrosyl Phosphatase Activator (Ptpa) Family Protein	0.0162	0.0280	2.70
245150_at	At2g47590	Photolyase/Blue Light Photoreceptor (Phr2)	0.0172	0.0286	3.57
262557_at	At1g31330	Photosystem I Reaction Center Subunit Iii Family Protein	0.0365	0.0421	2.54
257082_at	At3g20580	Phytochelatin Synthetase-Related	0.0012	0.0084	7.09
264508_at	At1g09570	Phytochrome A (Phya)	0.0135	0.0256	3.81
249666_at	At5g35840	Phytochrome C (Phyc)	0.0081	0.0197	3.18
245487_at	At4g16250	Phytochrome D (Phyd)	0.0062	0.0174	4.56
264510_at	At1g09530	Phytochrome Interacting Factor 3 (Pif3)	0.0053	0.0163	3.26
261407_at	At1g18810	Phytochrome Kinase Substrate-Related	0.0195	0.0305	2.75
245792_at	At1g32100	Pinoresinol-Lariciresinol Reductase, Putative	0.0044	0.0149	3.14
251434_at	At3g59850	Polygalacturonase, Putative / Pectinase, Putative	0.0022	0.0108	14.06
250570_at	At5g08170	Porphyromonas-Type Peptidyl-Arginine Deiminase Family Protein	0.0001	0.0023	3.30
251732_at	At3g56110	Prenylated Rab Acceptor (Pra1) Family Protein	0.0269	0.0360	3.42
256785_at	At3g13720	Prenylated Rab Acceptor (Pra1) Family Protein	0.0145	0.0264	8.05
259663_at	At1g55190	Prenylated Rab Acceptor (Pra1) Family Protein	0.0095	0.0213	2.76
264808_at	At1g08770	Prenylated Rab Acceptor (Pra1) Family Protein	0.0018	0.0098	2.96
261758_at	At1g08250	Prephenate Dehydratase Family Protein	0.0430	0.0462	3.22
255540_at	At4g01800	Preprotein Translocase Seca Subunit, Putative	0.0077	0.0193	3.07
253704_at	At4g29500	Prolidase-Related	0.0031	0.0128	2.68
256288_at	At3g12270	Protein Arginine N-Methyltransferase Family Protein	0.0461	0.0479	8.95
266687_at	At2g19670	Protein Arginine N-Methyltransferase, Putative	0.0010	0.0074	4.92
259757_at	At1g77510	Protein Disulfide Isomerase, Putative	0.0000	0.0010	4.22
262504_at	At1g21750	Protein Disulfide Isomerase, Putative	0.0004	0.0052	4.63
253871_at	At4g27440	Protochlorophyllide Reductase B, Chloroplast / Pcr B	0.0018	0.0098	4.58
255537_at	At4g01690	Protoporphyrinogen Oxidase (Ppox)	0.0408	0.0448	3.91
264497_at	At1g30840	Purine Permease-Related	0.0009	0.0073	2.72
267552_at	At2g32770	Purple Acid Phosphatase (Pap13)	0.0163	0.0280	7.00
260594_at	At1g55880	Pyridoxal-5'-Phosphate-Dependent Enzyme, Beta Family Protein	0.0034	0.0134	7.74
253416_at	At4g33070	Pyruvate Decarboxylase, Putative	0.0078	0.0193	3.04
256160_at	At1g30120	Pyruvate Dehydrogenase E1 Component Beta Subunit, Chloroplast	0.0138	0.0258	3.92
248486_at	At5g51060	Respiratory Burst Oxidase Protein C (Rbohc) / Nadph Oxidase	0.0281	0.0368	2.95
256011_at	At1g19230	Respiratory Burst Oxidase Protein E (Rbohe) / Nadph Oxidase	0.0043	0.0148	6.58
255891_at	At1g17870	S2P-like putative metalloprotease	0.0166	0.0282	2.71
251589_at	At3g58040	Seven In Absentia (Sina) Family Protein	0.0320	0.0393	2.88
267581_at	At2g41980	Seven In Absentia (Sina) Family Protein	0.0270	0.0361	4.11

249947_at	At5g19200	Short-Chain Dehydrogenase/Reductase (Sdr) Family Protein	0.0263	0.0356	5.84
267516_at	At2g30520	Signal Transducer Of Phototropic Response (Rpt2)	0.0431	0.0462	2.78
252613_at	At3g45190	Sit4 Phosphatase-Associated Family Protein	0.0478	0.0488	3.94
260679_at	At1g07990	Sit4 Phosphatase-Associated Family Protein	0.0125	0.0246	3.24
259451_at	At1g13890	Snap25 Homologous Protein, Putative	0.0154	0.0273	2.98
249773_at	At5g24140	Squalene Monooxygenase 2 / Squalene Epoxidase 2 (Sqp2)	0.0274	0.0363	2.56
253039_at	At4g37760	Squalene Monooxygenase, Putative / Squalene Epoxidase, Putative	0.0474	0.0486	3.27
266831_at	At2g22830	Squalene Monooxygenase, Putative / Squalene Epoxidase, Putative	0.0219	0.0323	2.74
250846_at	At5g04590	Sulfite Reductase / Ferredoxin (Sir)	0.0009	0.0071	3.19
260385_at	At1g74090	Sulfotransferase Family Protein	0.0000	0.0006	2.88
254512_at	At4g20230	Terpene Synthase/Cyclase Family Protein	0.0208	0.0316	4.99
257557_at	At3g14490	Terpene Synthase/Cyclase Family Protein	0.0182	0.0295	16.20
260935_at	At1g45110	Tetrapyrrole Methylase Family Protein	0.0386	0.0435	3.29
261053_at	At1g01320	Tetratricopeptide Repeat (Tpr)-Containing Protein	0.0000	0.0000	5.85
262577_at	At1g15290	Tetratricopeptide Repeat (Tpr)-Containing Protein	0.0167	0.0282	4.20
257932_at	At3g17040	Tetratricopeptide Repeat (Tpr)-Containing Protein	0.0082	0.0199	2.87
254275_at	At4g22670	Tetratricopeptide Repeat (Tpr)-Containing Protein	0.0027	0.0120	3.56
247832_at	At5g58550	Tetratricopeptide Repeat (Tpr)-Containing Protein	0.0275	0.0365	2.73
245118_at	At2g41680	Thioredoxin Reductase, Putative / Nadph-Dependent Thioredoxin Reductase, Putative	0.0426	0.0459	4.38
250234_at	At5g13420	Transaldolase, Putative	0.0012	0.0082	3.52
260967_at	At1g12230	Transaldolase, Putative	0.0164	0.0280	2.83
251396_at	At3g60750	Transketolase, Putative	0.0009	0.0071	9.76
253698_at	At4g29680	Type I Phosphodiesterase/Nucleotide Pyrophosphatase Family Protein	0.0055	0.0164	2.57
253197_at	At4g35250	Vestitone Reductase-Related	0.0297	0.0379	3.43
260521_at	At2g41740	Villin 2 (Vln2)	0.0287	0.0372	2.55
253719_at	At4g29490	Xaa-Pro Dipeptidase, Putative / Prolidase, Putative	0.0202	0.0311	3.59
266254_at	At2g27810	Xanthine/Uracil Permease Family Protein	0.0058	0.0170	2.80
E3. Glycolysis					
265735_at	At2g01140	Fructose-Bisphosphate Aldolase, Putative	0.0027	0.0120	4.38
263761_at	At2g21330	Fructose-Bisphosphate Aldolase, Putative	0.0366	0.0422	2.69
252022_at	At3g52930	Fructose-Bisphosphate Aldolase, Putative	0.0004	0.0052	5.32
253971_at	At4g26530	Fructose-Bisphosphate Aldolase, Putative	0.0243	0.0342	3.08
247983_at	At5g56630	Phosphofructokinase Family Protein	0.0005	0.0057	2.56
256228_at	At1g56190	Phosphoglycerate Kinase, Putative	0.0072	0.0186	2.56
262944_at	At1g79550	Phosphoglycerate Kinase, Putative	0.0174	0.0289	3.27
259969_at	At1g76550	Pyrophosphate--Fructose-6-Phosphate 1-Phosphotransferase Alpha Subunit, Putative	0.0008	0.0071	2.51
264386_at	At1g12000	Pyrophosphate--Fructose-6-Phosphate 1-Phosphotransferase Alpha Subunit, Putative	0.0015	0.0091	3.32
262806_at	At1g20950	Pyrophosphate--Fructose-6-Phosphate 1-Phosphotransferase Alpha Subunit, Putative	0.0306	0.0385	2.73
263922_s_at	At2g36580	Pyruvate Kinase, Putative	0.0328	0.0398	3.83
256836_at	At3g22960	Pyruvate Kinase, Putative	0.0323	0.0395	3.83
250526_at	At5g08570	Pyruvate Kinase, Putative	0.0015	0.0091	4.25
248283_at	At5g52920	Pyruvate Kinase, Putative	0.0162	0.0280	3.34
247989_at	At5g56350	Pyruvate Kinase, Putative	0.0077	0.0193	5.36
E4. Clathrin binding					
254261_at	At4g23460	Beta-Adaptin, Putative	0.0027	0.0120	6.30
254925_at	At4g11380	Beta-Adaptin, Putative	0.0215	0.0321	5.24
250816_at	At5g05010	Clathrin Adaptor Complexes Medium Subunit-Related	0.0025	0.0115	3.31
256437_s_at	At3g11130	Clathrin Heavy Chain, Putative	0.0318	0.0392	3.30
253528_s_at	At4g31480	Coatomer Beta Subunit, Putative / Beta-Coat Protein, Putative / Beta-Cop, Putative	0.0001	0.0030	3.07
263743_at	At2g21390	Coatomer Protein Complex, Subunit Alpha, Putative	0.0066	0.0180	3.92
264309_at	At1g62020	Coatomer Protein Complex, Subunit Alpha, Putative	0.0028	0.0120	3.69
262051_at	At1g79990	Coatomer Protein Complex, Subunit Beta 2 (Beta Prime), Putative	0.0475	0.0486	2.94
256146_at	At1g48760	Delta-Adaptin, Putative	0.0000	0.0012	9.81
246634_at	At1g31730	Epsilon-Adaptin, Putative	0.0317	0.0391	2.79
256485_at	At1g31440	Sh3 Domain-Containing Protein 1 (Sh3P1)	0.0199	0.0309	4.60
253229_at	At4g34660	Sh3 Domain-Containing Protein 2 (Sh3P2)	0.0092	0.0210	4.91
E5. Intracellular membrane/membrane bound organelle					
258625_at	At3g04370	33 Kda Secretory protein	0.0002	0.0032	3.27
265011_at	At1g24490	60 Kda Inner Membrane Family Protein	0.0077	0.0193	2.68
250766_at	At5g05550	6B-Interacting protein 1	0.0210	0.0317	2.82
266939_at	At2g18960	Atpase 1, Plasma Membrane-Type, Putative	0.0043	0.0149	3.54
253609_at	At4g30190	Atpase 1, Plasma Membrane-Type, Putative	0.0000	0.0018	2.64
249846_at	At5g23630	Atpase E1-E2 Type Family Protein	0.0005	0.0054	4.05
267488_at	At2g19110	Atpase E1-E2 Type Family Protein	0.0035	0.0135	3.84
266012_s_at	At2g07741	Atpase Subunit 6, Putative	0.0008	0.0071	3.41
252820_at	At3g42640	Atpase, Plasma Membrane-Type, Putative / Proton Pump, Putative	0.0019	0.0102	4.00
247439_at	At5g62670	Atpase, Plasma Membrane-Type, Putative / Proton Pump, Putative	0.0000	0.0000	2.99
265443_at	At2g20750	Beta-Expansin, Putative (Expb1)	0.0062	0.0174	6.08
265576_at	At2g20190	Clip-Associating Protein (Clasp) -Related	0.0069	0.0182	2.64
246263_at	At1g31780	Conserved Oligomeric Golgi Complex Component-Related	0.0167	0.0283	2.63

253876_at	At4g27430	Cop1-Interacting Protein 7 (Cip7)	0.0046	0.0152	3.50
261031_at	At1g17360	Cop1-Interacting Protein-Related	0.0004	0.0049	3.00
257385_at	At2g01800	Cop1-Interacting Protein-Related	0.0098	0.0217	10.46
249271_at	At5g41790	Cop1-Interactive Protein 1 / Cip1	0.0027	0.0120	9.09
251311_at	At3g61140	Cop9 Signalosome Complex Subunit 1 / Csn Complex Subunit 1 (Csn1)	0.0019	0.0101	2.51
257693_at	At3g12850	Cop9 Signalosome Complex-Related / Csn Complex-Related	0.0144	0.0263	3.62
258054_at	At3g16240	Delta Tonoplast Integral Protein (Delta-Tip)	0.0405	0.0446	2.52
263618_at	At2g04660	E3 Ubiquitin Ligase, Putative	0.0005	0.0058	5.14
246650_at	At5g35160	Endomembrane Protein 70, Putative	0.0312	0.0388	2.74
254827_at	At4g12650	Endomembrane Protein 70, Putative	0.0413	0.0451	3.79
260482_at	At1g10950	Endomembrane Protein 70, Putative	0.0236	0.0337	2.66
245211_at	At5g12370	Exocyst Complex Component Sec10-Related	0.0186	0.0299	3.08
259156_at	At3g10380	Exocyst Complex Component-Related	0.0424	0.0457	2.65
245979_at	At5g13150	Exocyst Subunit Exo70 Family Protein	0.0270	0.0361	7.85
261266_at	At1g26770	Expansin, Putative (Exp10)	0.0372	0.0425	2.68
261226_at	At1g20190	Expansin, Putative (Exp11)	0.0035	0.0135	3.61
266770_at	At2g03090	Expansin, Putative (Exp15)	0.0022	0.0107	3.20
249501_s_at	At5g39270	Expansin, Putative (Exp22)	0.0345	0.0410	5.28
267158_at	At2g37640	Expansin, Putative (Exp3)	0.0288	0.0373	4.33
258003_at	At3g29030	Expansin, Putative (Exp5)	0.0260	0.0354	2.64
258267_at	At3g15870	Fatty Acid Desaturase Family Protein	0.0004	0.0050	16.24
258555_at	At3g06860	Fatty Acid Multifunctional Protein (Mfp2)	0.0194	0.0304	4.65
258911_at	At3g06470	Gns1/Sur4 Membrane Family Protein	0.0273	0.0363	2.54
253842_at	At4g27860	Integral Membrane Family Protein	0.0498	0.0499	9.27
252002_at	At3g52760	Integral Membrane Yip1 Family Protein	0.0142	0.0262	2.72
266595_at	At2g46180	Intracellular Protein Transport Protein Uso1-Related	0.0176	0.0290	2.75
260820_at	At1g06840	Leucine-Rich Repeat Transmembrane Protein Kinase, Putative	0.0056	0.0167	3.37
263913_at	At2g36570	Leucine-Rich Repeat Transmembrane Protein Kinase, Putative	0.0088	0.0205	2.63
258684_at	At3g08680	Leucine-Rich Repeat Transmembrane Protein Kinase, Putative	0.0228	0.0332	2.92
257297_at	At3g28040	Leucine-Rich Repeat Transmembrane Protein Kinase, Putative	0.0012	0.0083	3.88
251718_at	At3g56100	Leucine-Rich Repeat Transmembrane Protein Kinase, Putative	0.0018	0.0098	9.86
251714_at	At3g56370	Leucine-Rich Repeat Transmembrane Protein Kinase, Putative	0.0047	0.0154	3.02
251075_at	At5g01890	Leucine-Rich Repeat Transmembrane Protein Kinase, Putative	0.0041	0.0146	3.06
250462_at	At5g10020	Leucine-Rich Repeat Transmembrane Protein Kinase, Putative	0.0005	0.0055	5.93
250102_at	At5g16590	Leucine-Rich Repeat Transmembrane Protein Kinase, Putative	0.0051	0.0160	3.71
248590_at	At5g49660	Leucine-Rich Repeat Transmembrane Protein Kinase, Putative	0.0110	0.0230	7.24
248237_at	At5g53890	Leucine-Rich Repeat Transmembrane Protein Kinase, Putative	0.0139	0.0259	2.64
247527_at	At5g61480	Leucine-Rich Repeat Transmembrane Protein Kinase, Putative	0.0300	0.0381	4.08
246986_at	At5g67280	Leucine-Rich Repeat Transmembrane Protein Kinase, Putative	0.0072	0.0186	2.94
256398_at	At3g06100	Major Intrinsic Family Protein / Mip Family Protein	0.0040	0.0145	3.32
263867_at	At2g36830	Major Intrinsic Family Protein / Mip Family Protein	0.0001	0.0019	5.91
257213_at	At3g15020	Malate Dehydrogenase (Nad), Mitochondrial, Putative	0.0014	0.0089	2.77
262569_at	At1g15180	Mate Efflux Family Protein	0.0007	0.0065	3.56
261880_at	At1g50500	Membrane Trafficking Vps53 Family Protein	0.0006	0.0060	2.65
263118_at	At1g03090	Methylcrotonyl-Coa Carboxylase Alpha Chain, Mitochondrial	0.0118	0.0239	2.97
253279_at	At4g34030	Methylcrotonyl-Coa Carboxylase Beta Chain, Mitochondrial	0.0002	0.0039	2.86
258862_at	At3g02090	Mitochondrial Processing Peptidase Beta Subunit, Putative	0.0250	0.0347	2.66
249224_at	At5g42130	Mitochondrial Substrate Carrier Family Protein	0.0097	0.0216	2.77
263943_at	At2g35800	Mitochondrial Substrate Carrier Family Protein	0.0049	0.0158	2.50
249900_at	At5g22640	Morn (Membrane Occupation And Recognition Nexus) Repeat-Containing Protein	0.0185	0.0299	3.21
249627_at	At5g37510	Nadh-Ubiquinone Dehydrogenase, Mitochondrial, Putative	0.0224	0.0329	3.84
250334_at	At5g11770	Nadh-Ubiquinone Oxidoreductase 20 Kda Subunit, Mitochondrial	0.0332	0.0401	3.05
256277_at	At3g12120	Omega-6 Fatty Acid Desaturase, Endoplasmic Reticulum (Fad2) / Delta-12 Desaturase	0.0327	0.0398	4.63
259532_at	At1g12470	Pep3/Vps18/Deep Orange Family Protein	0.0302	0.0382	2.58
249990_at	At5g18540	Periplasmic Cytochrome C-Related	0.0214	0.0319	4.21
251993_at	At3g52960	Peroxiredoxin Type 2, Putative	0.0068	0.0181	2.57
247422_at	At5g62810	Peroxisomal Protein (Pex14)	0.0407	0.0448	4.19
248010_at	At5g56290	Peroxisomal Targeting Signal Type 1 Receptor (Pex5)	0.0093	0.0211	2.58
259430_at	At1g01610	Phospholipid/Glycerol Acyltransferase Family Protein	0.0498	0.0499	4.83
255674_at	At4g00430	Plasma Membrane Intrinsic Protein, Putative	0.0275	0.0365	3.31
249009_at	At5g44610	Plasma Membrane Polypeptide-Related	0.0062	0.0174	6.87
259504_at	At1g15690	Pyrophosphate-Energized Vacuolar Membrane Proton Pump	0.0351	0.0413	4.16
264871_at	At1g24180	Pyruvate Dehydrogenase E1 Component Alpha Subunit, Mitochondrial, Putative	0.0221	0.0326	2.50
265822_at	At2g17980	Sec1 Family Protein	0.0009	0.0073	3.48
247632_at	At5g60460	Sec61Beta Family Protein	0.0005	0.0054	5.80
247060_at	At5g66760	Succinate Dehydrogenase (Ubiquinone) Flavoprotein Subunit, Mitochondrial	0.0001	0.0021	3.01
265329_at	At2g18450	Succinate Dehydrogenase (Ubiquinone) Flavoprotein Subunit, Mitochondrial, Putative	0.0244	0.0343	6.01
256387_at	At3g06170	Tms Membrane Family Protein / Tumour Differentially Expressed (Tde) Family Protein	0.0417	0.0454	3.48
257357_at	At2g41050	Transmembrane Family Protein	0.0136	0.0257	7.13
261783_at	At1g08190	Vacuolar Assembly Protein, Putative (Vps41)	0.0473	0.0485	6.59
264302_at	At1g78900	Vacuolar Atp Synthase Catalytic Subunit A / V-Atpase A Subunit	0.0026	0.0118	4.07
252998_at	At4g38510	Vacuolar Atp Synthase Subunit B, Putative / V-Atpase B Subunit	0.0354	0.0415	3.56
252027_at	At3g52850	Vacuolar Sorting Receptor, Putative	0.0002	0.0040	2.54
257971_at	At3g27530	Vesicle Tethering Family Protein	0.0103	0.0222	10.32

F. Proteins with binding function or cofactor requirement

251256_at	At3g62300	Agenet Domain-Containing Protein	0.0081	0.0197	10.44
245415_at	At4g17330	Agenet Domain-Containing Protein	0.0161	0.0279	6.61
260921_at	At1g21540	Amp-Binding Protein, Putative	0.0456	0.0478	4.35
245807_at	At1g46768	Ap2 Domain-Containing Protein Rap2.1 (Rap2.1)	0.0080	0.0197	4.15
262197_at	At1g53910	Ap2 Domain-Containing Protein Rap2.12 (Rap2.12)	0.0480	0.0489	4.91
258366_at	At3g14230	Ap2 Domain-Containing Protein Rap2.2 (Rap2.2)	0.0120	0.0241	4.90
263486_at	At2g22200	Ap2 Domain-Containing Transcription Factor	0.0067	0.0180	3.79
262135_at	At1g78080	Ap2 Domain-Containing Transcription Factor Rap2.4	0.0222	0.0326	3.23
252486_at	At3g46510	Armadillo/Beta-Catenin Repeat Family Protein	0.0385	0.0435	2.68
251407_at	At3g60350	Armadillo/Beta-Catenin Repeat Family Protein / F-Box Family Protein	0.0353	0.0414	4.90
266727_at	At2g03150	Atp/Gtp-Binding Protein Family	0.0168	0.0283	5.21
256308_s_at	At1g30410	Atp-Binding Cassette Transport Protein, Putative	0.0000	0.0010	16.99
253085_s_at	At4g36270	Atp-Binding Region, Atpase-Like Domain-Containing Protein	0.0409	0.0449	4.59
248487_at	At5g51070	Atp-Dependent Clp Protease Atp-Binding Subunit (Clpd), (Erd1)	0.0120	0.0240	2.91
248480_at	At5g50920	Atp-Dependent Clp Protease Atp-Binding Subunit / Clpc	0.0003	0.0043	4.21
255586_at	At4g01560	Brix Domain-Containing Protein	0.0213	0.0319	2.64
252402_s_at	At3g48050	Bromo-Adjacent Homology (Bah) Domain-Containing Protein	0.0491	0.0495	3.84
266580_at	At2g46260	Btb/Poz Domain-Containing Protein	0.0331	0.0399	2.53
251328_at	At3g61600	Btb/Poz Domain-Containing Protein	0.0001	0.0022	2.62
263205_at	At1g05500	C2 Domain-Containing Protein	0.0007	0.0064	3.45
261935_at	At1g22610	C2 Domain-Containing Protein	0.0013	0.0084	2.53
260719_at	At1g48090	C2 Domain-Containing Protein	0.0139	0.0258	3.23
260511_at	At1g51570	C2 Domain-Containing Protein	0.0033	0.0132	3.91
262209_at	At1g74720	C2 Domain-Containing Protein	0.0011	0.0081	3.25
259222_at	At3g03680	C2 Domain-Containing Protein	0.0022	0.0106	2.72
251364_at	At3g61300	C2 Domain-Containing Protein	0.0095	0.0213	6.12
248731_at	At5g48060	C2 Domain-Containing Protein	0.0122	0.0244	8.30
265388_s_at	At2g20990	C2 Domain-Containing Protein (Syta)	0.0022	0.0108	2.72
259731_at	At1g77460	C2 Domain-Containing Protein / Armadillo/Beta-Catenin Repeat Family Protein	0.0005	0.0054	2.87
264568_at	At1g05150	Calcium-Binding Ef Hand Family Protein	0.0010	0.0074	3.13
262492_at	At1g21630	Calcium-Binding Ef Hand Family Protein	0.0000	0.0019	2.81
261421_at	At1g18840	Calmodulin-Binding Family Protein	0.0366	0.0422	3.28
262210_at	At1g74690	Calmodulin-Binding Family Protein	0.0017	0.0097	7.12
260610_at	At2g43680	Calmodulin-Binding Family Protein	0.0000	0.0018	3.21
257229_at	At3g16490	Calmodulin-Binding Family Protein	0.0096	0.0215	5.28
250613_at	At5g07240	Calmodulin-Binding Family Protein	0.0025	0.0115	6.31
249709_at	At5g35670	Calmodulin-Binding Family Protein	0.0001	0.0019	7.63
247990_at	At5g56360	Calmodulin-Binding Protein	0.0013	0.0086	6.03
247270_at	At5g64220	Calmodulin-Binding Protein	0.0078	0.0194	3.31
256764_at	At3g29310	Calmodulin-Binding Protein-Related	0.0094	0.0212	3.02
259438_at	At1g01510	C-Terminal Binding Protein (Angustifolia)	0.0015	0.0091	4.88
258324_at	At3g22780	Cxc Domain Protein (Tso1)	0.0037	0.0140	2.62
258660_at	At3g09850	D111/G-Patch Domain-Containing Protein	0.0008	0.0071	2.99
265123_at	At1g55440	Dc1 Domain-Containing Protein	0.0041	0.0145	5.68
264534_at	At1g55700	Dc1 Domain-Containing Protein	0.0300	0.0381	6.41
267478_at	At2g02700	Dc1 Domain-Containing Protein	0.0002	0.0038	3.01
251501_at	At3g59120	Dc1 Domain-Containing Protein	0.0007	0.0065	3.91
251502_at	At3g59130	Dc1 Domain-Containing Protein	0.0165	0.0282	8.81
261036_at	At1g17470	Developmentally Regulated Gtp-Binding Protein (Drg1)	0.0487	0.0494	4.47
251664_at	At3g56940	Dicarboxylate Diiron Protein, Putative (Crd1)	0.0284	0.0371	2.53
247370_at	At5g63320	DNA-binding Bromodomain containing protein	0.0008	0.0071	3.79
267386_at	At2g44430	DNA-binding Bromodomain containing protein	0.0005	0.0056	13.46
259164_at	At3g01770	DNA-binding Bromodomain containing protein	0.0040	0.0145	2.58
250424_at	At5g10550	DNA-binding Bromodomain containing protein	0.0092	0.0209	2.66
247147_at	At5g65630	DNA-binding Bromodomain containing protein	0.0177	0.0291	3.04
261548_at	At1g63480	DNA-Binding Family Protein	0.0002	0.0035	10.57
258819_at	At3g04590	DNA-Binding Family Protein	0.0019	0.0101	2.79
262586_at	At1g15480	DNA-Binding Family Protein	0.0000	0.0000	11.43
260331_at	At1g80270	DNA-Binding Family Protein	0.0461	0.0479	4.28
249677_at	At5g35970	Dna-Binding Protein, Putative	0.0228	0.0331	2.98
247452_at	At5g62430	Dof-Type Zinc Finger Domain-Containing Protein	0.0460	0.0479	8.40
252429_at	At3g47500	Dof-Type Zinc Finger Domain-Containing Protein	0.0036	0.0136	5.63
256430_at	At3g11020	Dre-Binding Protein (Dreb2B)	0.0371	0.0425	2.78
251138_at	At5g01160	E-Cadherin Binding Protein-Related	0.0075	0.0190	2.57
256119_at	At1g18070	Ef-1-Alpha-Related Gtp-Binding Protein, Putative	0.0052	0.0162	3.26
262017_at	At1g35550	Elongation Factor Tu C-Terminal Domain-Containing Protein	0.0000	0.0004	3.15
250274_at	At5g13020	Emsy N Terminus Domain-Containing Protein / Ent Domain-Containing Protein	0.0066	0.0179	2.65
266328_at	At2g01600	Epsin N-Terminal Homology (Enth) Domain-Containing Protein	0.0118	0.0238	2.68
247941_at	At5g57200	Epsin N-Terminal Homology (Enth) Domain-Containing Protein	0.0274	0.0363	3.25
257053_at	At3g15210	Ethylene-Responsive Element-Binding Factor 4 (Erf4)	0.0433	0.0463	3.73
253257_at	At4g34390	Extra-Large Guanine Nucleotide Binding Protein, Putative / G-Protein, Putative	0.0193	0.0303	2.79

263228_at	At1g30700	Fad-Binding Domain-Containing Protein	0.0454	0.0477	4.36
264527_at	At1g30760	Fad-Binding Domain-Containing Protein	0.0016	0.0095	5.23
251178_at	At3g63440	Fad-Binding Domain-Containing Protein / Cytokinin Oxidase Family Protein	0.0105	0.0224	4.86
265814_at	At2g17930	Fat Domain-Containing Protein / Phosphatidylinositol 3- And 4-Kinase Family Protein	0.0056	0.0167	2.87
257963_at	At3g19840	Ff Domain-Containing Protein / Ww Domain-Containing Protein	0.0002	0.0037	2.68
245137_at	At2g45460	Forkhead-Associated Domain-Containing Protein / Fha Domain-Containing Protein	0.0075	0.0190	2.58
258822_s_at	At3g07260	Forkhead-Associated Domain-Containing Protein / Fha Domain-Containing Protein	0.0125	0.0246	2.65
248771_at	At5g47790	Forkhead-Associated Domain-Containing Protein / Fha Domain-Containing Protein	0.0412	0.0451	2.91
246261_at	At1g31810	Formin Homology 2 Domain-Containing Protein / Fh2 Domain-Containing Protein	0.0076	0.0191	3.03
260565_at	At2g43800	Formin Homology 2 Domain-Containing Protein / Fh2 Domain-Containing Protein	0.0093	0.0210	2.63
257912_at	At3g25500	Formin Homology 2 Domain-Containing Protein / Fh2 Domain-Containing Protein	0.0028	0.0123	2.52
248696_at	At5g48360	Formin Homology 2 Domain-Containing Protein / Fh2 Domain-Containing Protein	0.0032	0.0131	5.02
246995_at	At5g67470	Formin Homology 2 Domain-Containing Protein / Fh2 Domain-Containing Protein	0.0006	0.0058	2.76
245714_at	At5g04280	Glycine-Rich Rna-Binding Protein	0.0043	0.0149	2.79
252885_at	At4g39260	Glycine-Rich Rna-Binding Protein 8 (Grp8) (Ccr1)	0.0085	0.0201	3.50
264921_at	At1g60650	Glycine-Rich Rna-Binding Protein, Putative	0.0012	0.0083	3.19
247891_at	At5g57960	Gtp-Binding Family Protein	0.0344	0.0409	3.39
265154_at	At1g30960	Gtp-Binding Protein (Erg)	0.0009	0.0072	4.67
256207_at	At1g50920	Gtp-Binding Protein-Related	0.0010	0.0074	4.89
251158_at	At3g63150	Gtp-Binding Protein-Related	0.0048	0.0155	4.14
256144_at	At1g48630	Guanine Nucleotide-Binding Family Protein	0.0231	0.0333	2.69
248933_at	At5g46070	Guanylate-Binding Family Protein	0.0003	0.0045	4.27
256823_at	At3g22220	Hat Dimerisation Domain-Containing Protein	0.0053	0.0163	2.56
252947_at	At4g38600	Hect-Domain-Containing Protein / Ubiquitin-Transferase Family Protein	0.0044	0.0150	5.61
255444_at	At4g02560	Homeobox Protein Luminidependens (Ld)	0.0028	0.0123	3.20
263956_at	At2g35940	Homeodomain-Containing Protein	0.0108	0.0228	6.00
259927_at	At1g75100	J-domain of auxilin	0.0066	0.0179	2.70
261988_at	At1g33680	KH Domain-Containing Protein	0.0036	0.0137	5.01
266846_at	At2g25970	KH Domain-Containing Protein	0.0331	0.0400	4.78
258790_at	At3g04610	KH Domain-Containing Protein	0.0051	0.0160	2.83
255136_s_at	At4g08350	Kow Domain-Containing Transcription Factor Family Protein	0.0000	0.0003	2.54
267214_at	At2g43970	La Domain-Containing Protein	0.0011	0.0078	2.73
266544_at	At2g35300	Late Embryogenesis Abundant Group 1 Domain-Containing Protein	0.0289	0.0373	6.56
247103_at	At5g66610	Lim Domain-Containing Protein	0.0235	0.0337	4.06
262704_at	At1g16530	Lob Domain Protein 3 / Lateral Organ Boundaries Domain Protein 3 (Lbd3)	0.0113	0.0233	2.55
264626_at	At1g65620	Lob Domain Protein 6 / Lateral Organ Boundaries Domain Protein 6 (Lbd6)	0.0392	0.0438	3.33
259055_at	At3g03340	Luc7 N_Terminus Domain-Containing Protein	0.0012	0.0084	2.68
246436_at	At5g17440	Luc7 N_Terminus Domain-Containing Protein	0.0390	0.0438	11.06
254089_at	At4g24800	Ma3 Domain-Containing Protein	0.0005	0.0055	7.97
250066_at	At5g17930	Ma3 Domain-Containing Protein	0.0101	0.0220	10.51
258333_at	At3g16000	Matrix-Localized Mar Dna-Binding Protein-Related	0.0050	0.0158	5.14
263654_at	At1g04300	Meprin And Traf Homology Domain-Containing Protein	0.0287	0.0372	2.72
257673_at	At3g20370	Meprin And Traf Homology Domain-Containing Protein	0.0119	0.0239	2.80
249100_at	At5g43560	Meprin And Traf Homology Domain-Containing Protein	0.0082	0.0199	4.07
262600_at	At1g15340	Methyl-Cpg-Binding Domain-Containing Protein	0.0082	0.0199	4.97
258264_at	At3g15790	Methyl-Cpg-Binding Domain-Containing Protein	0.0015	0.0091	3.34
264471_at	At1g67120	Midasin-Related	0.0414	0.0452	5.15
261885_at	At1g80930	Mif4G Domain-Containing Protein / Ma3 Domain-Containing Protein	0.0080	0.0196	4.31
251431_at	At3g60240	Mif4G Domain-Containing Protein / Ma3 Domain-Containing Protein	0.0148	0.0268	5.42
263727_at	At2g13540	Mrna Cap-Binding Protein (Abh1)	0.0020	0.0105	2.91
251157_at	At3g63140	Mrna-Binding Protein, Putative	0.0289	0.0373	2.59
264048_at	At2g22400	Nol1/Nop2/Sun Family Protein	0.0080	0.0197	2.66
245387_at	At4g17520	Nuclear Rna-Binding Protein, Putative	0.0005	0.0055	3.50
256605_at	At3g32940	Nucleic acid binding	0.0054	0.0164	3.26
250516_at	At5g09620	Octicosapeptide/Phox/Bem1P (Pb1) Domain-Containing Protein	0.0007	0.0064	3.07
261040_at	At1g17370	Oligouridylate-Binding Protein, Putative	0.0112	0.0232	4.25
262548_at	At1g31280	Paz Domain-Containing Protein / Piwi Domain-Containing Protein	0.0002	0.0037	2.53
263586_at	At2g25350	Phox (Px) Domain-Containing Protein	0.0232	0.0334	10.56
247822_at	At5g58440	Phox (Px) Domain-Containing Protein	0.0042	0.0147	3.52
261975_at	At1g64640	Plastocyanin-Like Domain-Containing Protein	0.0035	0.0135	11.46
248236_at	At5g53870	Plastocyanin-Like Domain-Containing Protein	0.0167	0.0283	3.87
264111_at	At2g13690	Prli-Interacting Factor, Putative	0.0232	0.0334	2.50
262348_at	At2g48160	Pwwp Domain-Containing Protein	0.0114	0.0234	5.14
249281_at	At5g41940	Rabgap/Tbc Domain-Containing Protein	0.0040	0.0145	3.08
267562_at	At2g39670	Radical Sam Domain-Containing Protein	0.0081	0.0197	4.01
256343_at	At1g72090	Radical Sam Domain-Containing Protein / Tram Domain-Containing Protein	0.0003	0.0043	3.84
254844_at	At4g11790	Ran-Binding Protein 1 Domain-Containing Protein	0.0136	0.0257	4.62
264395_at	At1g12070	Rho Gdp-Dissociation Inhibitor Family Protein	0.0417	0.0453	2.70
254021_at	At4g25650	Rieske (2Fe-2S) Domain-Containing Protein	0.0270	0.0361	2.64
263533_at	At2g24820	Rieske (2Fe-2S) Domain-Containing Protein	0.0148	0.0268	3.41
262824_at	At1g11650	Rna-Binding Protein 45 (Rbp45), Putative	0.0461	0.0479	2.63
262433_s_at	At1g47500	Rna-Binding Protein 47 (Rbp47), Putative	0.0088	0.0205	3.21
265966_at	At2g37220	Rna-Binding Protein Cp29, Putative	0.0077	0.0193	3.16
264822_at	At1g03457	Rna-Binding Protein, Putative	0.0121	0.0242	3.11

264069_at	At2g28000	Rubisco Subunit Binding-Protein Alpha Subunit, Chloroplast	0.0215	0.0321	3.06
261206_at	At1g12800	S1 Rna-Binding Domain-Containing Protein	0.0034	0.0133	3.62
258573_at	At3g04260	Sap Domain-Containing Protein	0.0171	0.0286	2.67
264480_at	At1g77300	Set Domain-Containing Protein	0.0309	0.0387	2.53
267290_at	At2g23750	Set Domain-Containing Protein	0.0166	0.0282	2.92
250170_at	At5g14260	Set Domain-Containing Protein	0.0197	0.0307	2.94
254745_at	At4g13460	Set Domain-Containing Protein (Suvh9)	0.0026	0.0118	3.49
262798_at	At1g20980	Spl1-Related2 Protein (Spl1R2)	0.0002	0.0040	2.61
261872_s_at	At1g11520	Spliceosome Associated Protein-Related	0.0042	0.0147	3.28
259107_at	At3g05460	Sporozoite Surface Protein-Related	0.0192	0.0303	9.08
251450_at	At3g60030	Squamosa Promoter-Binding Protein-Like 12 (Spl12)	0.0390	0.0438	2.90
249793_at	At5g23680	Sterile Alpha Motif (Sam) Domain-Containing Protein	0.0002	0.0034	2.50
250218_at	At5g14170	Swib Complex Baf60B Domain-Containing Protein	0.0011	0.0078	2.99
252504_at	At3g46590	Telomere Repeat-Binding Protein, Putative	0.0064	0.0177	3.53
251909_at	At3g53790	Telomere-Binding Protein, Putative	0.0276	0.0365	6.20
258219_at	At3g17880	Tetratricoredoxin (Tdx)	0.0040	0.0144	2.96
254916_at	At4g11340	Toll-Interleukin-Resistance (Tir) Domain-Containing Protein	0.0215	0.0321	3.23
264649_at	At1g09060	Transcription Factor Jumonji (Jmjc) Domain-Containing Protein	0.0000	0.0017	2.93
260627_at	At1g62310	Transcription Factor Jumonji (Jmjc) Domain-Containing Protein	0.0168	0.0283	2.99
259252_at	At3g07610	Transcription Factor Jumonji (Jmjc) Domain-Containing Protein	0.0217	0.0322	16.24
254412_at	At4g21430	Transcription Factor Jumonji (Jmjc) Domain-Containing Protein	0.0349	0.0412	5.25
245935_at	At5g19840	Transcription Factor Jumonji (Jmjc) Domain-Containing Protein	0.0283	0.0370	2.98
266407_at	At2g38560	Transcription Factor S-Ii (Tfiis) Domain-Containing Protein	0.0083	0.0200	2.74
263261_at	At1g10580	Transducin Family Protein / Wd-40 Repeat Family Protein	0.0109	0.0229	3.01
262395_at	At1g49540	Transducin Family Protein / Wd-40 Repeat Family Protein	0.0004	0.0050	4.64
265259_at	At2g20330	Transducin Family Protein / Wd-40 Repeat Family Protein	0.0013	0.0085	4.70
255830_at	At2g33340	Transducin Family Protein / Wd-40 Repeat Family Protein	0.0017	0.0097	3.39
263824_at	At2g40360	Transducin Family Protein / Wd-40 Repeat Family Protein	0.0059	0.0171	3.95
260606_at	At2g43770	Transducin Family Protein / Wd-40 Repeat Family Protein	0.0245	0.0343	2.76
257684_s_at	At3g13290	Transducin Family Protein / Wd-40 Repeat Family Protein	0.0357	0.0416	7.57
256967_at	At3g21060	Transducin Family Protein / Wd-40 Repeat Family Protein	0.0026	0.0118	4.98
258166_at	At3g21540	Transducin Family Protein / Wd-40 Repeat Family Protein	0.0328	0.0398	5.84
252273_at	At3g49660	Transducin Family Protein / Wd-40 Repeat Family Protein	0.0040	0.0145	5.63
250222_at	At5g14050	Transducin Family Protein / Wd-40 Repeat Family Protein	0.0058	0.0169	4.08
249798_at	At5g23730	Transducin Family Protein / Wd-40 Repeat Family Protein	0.0009	0.0071	2.83
245861_at	At5g28300	Trihelix Dna-Binding Protein, Putative	0.0000	0.0015	2.74
247517_at	At5g61780	Tudor Domain-Containing Protein / Nuclease Family Protein	0.0000	0.0005	4.03
250626_at	At5g07350	Tudor Domain-Containing Protein / Nuclease Family Protein	0.0006	0.0060	2.89
246541_at	At5g15400	U-Box Domain-Containing Protein	0.0057	0.0169	2.98
259605_at	At1g27910	U-Box Domain-Containing Protein	0.0003	0.0046	15.60
261597_at	At1g49780	U-Box Domain-Containing Protein	0.0088	0.0205	2.86
246292_at	At3g56860	Ubp1 Interacting Protein 2A (Uba2A)	0.0188	0.0300	5.83
245557_at	At4g15410	Ubx Domain-Containing Protein	0.0020	0.0104	4.11
254936_at	At4g10790	Ubx Domain-Containing Protein	0.0002	0.0037	3.19
254452_at	At4g21100	Uv-Damaged Dna-Binding Protein, Putative	0.0098	0.0217	3.40
253418_at	At4g32760	Vhs Domain-Containing Protein / Gat Domain-Containing Protein	0.0297	0.0379	4.93
260276_at	At1g80450	Vq Motif-Containing Protein	0.0001	0.0019	21.94
259501_at	At1g15750	Wd-40 Repeat Family Protein	0.0332	0.0400	4.29
264548_at	At1g55680	Wd-40 Repeat Family Protein	0.0004	0.0048	2.55
258268_at	At3g15880	Wd-40 Repeat Family Protein	0.0459	0.0479	2.81
257553_at	At3g16830	Wd-40 Repeat Family Protein	0.0248	0.0346	3.32
254871_at	At4g11920	Wd-40 Repeat Family Protein	0.0040	0.0145	5.02
248198_at	At5g54200	Wd-40 Repeat Family Protein	0.0044	0.0149	8.64
256072_at	At1g18080	Wd-40 Repeat Family Protein	0.0320	0.0393	5.40
267501_at	At2g45540	Wd-40 Repeat Family Protein	0.0213	0.0319	3.18
267102_at	At2g41500	Wd-40 Repeat Family Protein	0.0148	0.0268	3.65
245796_at	At1g32230	Wwe Domain-Containing Protein / Ceo Protein, Putative (Ceo)	0.0100	0.0219	2.55
261837_s_at	At1g15910	Xh/Xs Domain-Containing Protein / Xs Zinc Finger Domain-Containing Protein	0.0379	0.0431	2.57
261891_at	At1g80790	Xh/Xs Domain-Containing Protein / Xs Zinc Finger Domain-Containing Protein	0.0010	0.0077	3.05
249843_at	At5g23570	Xs Domain-Containing Protein / Xs Zinc Finger Domain-Containing Protein-Related	0.0213	0.0319	3.25
247555_at	At5g61020	Yt521-B-Like Family Protein	0.0041	0.0145	2.65
264814_at	At2g17900	Zinc Finger (Mynd Type) Family Protein / Set Domain-Containing Protein	0.0093	0.0210	5.63
F1. Metal ion binding/metal binding					
249969_at	At5g19090	Heavy-Metal-Associated Domain-Containing Protein	0.0162	0.0280	2.82
252956_at	At4g38580	Heavy-Metal-Associated Domain-Containing Protein	0.0104	0.0223	2.92
253516_at	At4g31360	Selenium binding	0.0231	0.0334	13.66
G. Protein synthesis and translation					
260165_at	At1g79850	30S Ribosomal Protein S17, Chloroplast / Cs17 (Rps17)	0.0005	0.0057	2.53
263821_s_at	At2g09990	40S Ribosomal Protein S16 (Rps16A)	0.0192	0.0303	2.66
246747_at	At5g27700	40S Ribosomal Protein S21 (Rps21C)	0.0116	0.0237	4.58
251357_at	At3g61110	40S Ribosomal Protein S27 (Ars27A)	0.0101	0.0220	3.12
252693_s_at	At3g44010	40S Ribosomal Protein S29 (Rps29B)	0.0004	0.0048	2.62

253248_at	At4g34670	40S Ribosomal Protein S3A (Rps3Ab)	0.0272	0.0362	2.51
250440_at	At5g10360	40S Ribosomal Protein S6 (Rps6B)	0.0074	0.0188	3.29
260426_at	At1g72370	40S Ribosomal Protein Sa (Rpsaa)	0.0120	0.0241	2.66
267349_at	At2g40010	60S Acidic Ribosomal Protein P0 (Rpp0A)	0.0049	0.0156	7.47
259006_at	At3g09200	60S Acidic Ribosomal Protein P0 (Rpp0B)	0.0106	0.0226	2.63
261578_at	At1g01100	60S Acidic Ribosomal Protein P1 (Rpp1A)	0.0201	0.0311	3.41
255657_at	At4g00810	60S Acidic Ribosomal Protein P1 (Rpp1B)	0.0103	0.0222	3.34
248768_at	At5g47700	60S Acidic Ribosomal Protein P1 (Rpp1C)	0.0058	0.0169	4.86
247900_at	At5g57290	60S Acidic Ribosomal Protein P3 (Rpp3B)	0.0000	0.0004	2.80
252347_at	At3g48130	60S Ribosomal Protein L13 (Rpl13A), Pseudogene	0.0083	0.0200	3.33
245355_at	At4g17390	60S Ribosomal Protein L15 (Rpl15B)	0.0185	0.0298	2.85
262117_at	At1g02780	60S Ribosomal Protein L19 (Rpl19A)	0.0007	0.0068	4.60
264421_at	At1g43170	60S Ribosomal Protein L3 (Rpl3A)	0.0134	0.0255	2.67
258715_at	At3g09630	60S Ribosomal Protein L4/L1 (Rpl4A)	0.0088	0.0205	2.94
261911_at	At1g80750	60S Ribosomal Protein L7 (Rpl7A)	0.0364	0.0421	6.12
256648_at	At3g13580	60S Ribosomal Protein L7 (Rpl7D)	0.0276	0.0365	5.03
251185_at	At3g62870	60S Ribosomal Protein L7A (Rpl7Ab)	0.0012	0.0082	2.51
247644_s_at	At5g60390	Elongation Factor 1-Alpha / Ef-1-Alpha	0.0398	0.0442	4.92
253758_at	At4g29060	Elongation Factor Ts Family Protein	0.0001	0.0019	3.50
260786_s_at	At1g06220	Elongation Factor Tu Family Protein	0.0003	0.0047	4.98
262645_at	At1g62750	Elongation Factor Tu Family Protein	0.0000	0.0003	4.78
254873_at	At4g11420	Eukaryotic Translation Initiation Factor 3 Subunit 10 / Eif-3 Theta / Eif3A (Tif3A1)	0.0039	0.0144	7.34
251738_at	At3g56150	Eukaryotic Translation Initiation Factor 3 Subunit 8 / Eif3 P110 / Eif3C / P105 (Tif3C1)	0.0006	0.0058	3.68
246865_s_at	At5g25780	Eukaryotic Translation Initiation Factor 3 Subunit 9, Putative / Eif-3 Eta	0.0067	0.0180	3.94
257023_at	At3g19760	Eukaryotic Translation Initiation Factor 4A, Putative / Eif-4A, Putative	0.0153	0.0273	3.16
247839_at	At5g57870	Eukaryotic Translation Initiation Factor 4F, Putative / Eif-4F, Putative	0.0301	0.0381	4.28
261827_at	At1g11480	Eukaryotic Translation Initiation Factor-Related	0.0006	0.0061	5.49
266127_s_at	At2g45030	Mitochondrial Elongation Factor, Putative	0.0052	0.0162	5.70
251120_at	At3g63490	Ribosomal Protein L1 Family Protein	0.0010	0.0076	3.50
259505_at	At1g15810	Ribosomal Protein S15 Family Protein	0.0033	0.0132	3.12
259392_at	At1g06380	Ribosomal Protein-Related	0.0020	0.0103	2.60
262859_at	At1g64790	Translational Activator Family Protein	0.0033	0.0133	2.81
H. Protein fate					
247810_at	At5g58290	26S Proteasome Aaa-Atpase Subunit (Rpt3)	0.0308	0.0386	4.16
246147_s_at	At5g20000	26S Proteasome Aaa-Atpase Subunit, Putative	0.0021	0.0105	2.72
263716_at	At2g20580	26S Proteasome Regulatory Subunit S2 (Rpn1)	0.0131	0.0252	5.64
262686_at	At1g75990	26S Proteasome Regulatory Subunit S3, Putative (Rpn3)	0.0369	0.0424	3.15
261174_at	At1g04810	26S Proteasome Regulatory Subunit, Putative	0.0411	0.0450	7.16
267543_at	At2g32730	26S Proteasome Regulatory Subunit, Putative	0.0043	0.0148	3.24
260842_at	At1g29150	26S Proteasome Regulatory Subunit, Putative (Rpn6)	0.0039	0.0143	3.21
251287_at	At3g61820	Aspartyl Protease Family Protein	0.0410	0.0449	3.45
251025_at	At5g02190	Aspartyl Protease Family Protein	0.0000	0.0009	7.32
264641_at	At1g09130	Atp-Dependent Clp Protease Proteolytic Subunit, Putative	0.0459	0.0479	4.10
263685_at	At1g26830	Cullin, Putative	0.0203	0.0312	3.03
266509_at	At2g47940	Degp2 Protease (Degp2)	0.0000	0.0000	3.41
260616_at	At1g53280	Dj-1 Family Protein	0.0041	0.0145	3.50
255746_at	At1g32020	F-Box Family Protein	0.0059	0.0170	3.45
263670_at	At2g04840	F-Box Family Protein	0.0073	0.0187	17.37
265997_at	At2g24250	F-Box Family Protein	0.0096	0.0214	10.09
258411_at	At3g17280	F-Box Family Protein	0.0089	0.0206	12.06
258378_at	At3g17490	F-Box Family Protein	0.0366	0.0422	6.88
252284_at	At3g49020	F-Box Family Protein	0.0462	0.0480	4.06
251547_at	At3g58860	F-Box Family Protein	0.0066	0.0179	2.99
250883_at	At5g03970	F-Box Family Protein	0.0307	0.0385	2.67
253369_at	At4g33210	F-Box Family Protein (Fbl15)	0.0001	0.0027	3.15
253364_at	At4g33160	F-Box Family Protein (Fbx13)	0.0433	0.0464	5.09
264955_at	At1g76920	F-Box Family Protein (Fbx3)	0.0410	0.0449	2.50
264058_at	At2g03560	F-Box Family Protein (Fbx7)	0.0258	0.0353	3.90
266179_at	At2g02300	F-Box Family Protein / Skp1 Interacting Partner 3-Related	0.0054	0.0164	3.03
263721_at	At2g13630	F-Box Family Protein-Related	0.0476	0.0487	3.15
258142_at	At3g18120	F-Box Family Protein-Related	0.0281	0.0369	2.51
257195_at	At3g23680	F-Box Family Protein-Related	0.0202	0.0311	3.40
262626_at	At1g06430	Ftsh Protease, Putative	0.0132	0.0253	3.85
261064_at	At1g07510	Ftsh Protease, Putative	0.0040	0.0145	2.73
262937_at	At1g79560	Ftsh Protease, Putative	0.0018	0.0100	4.08
266842_at	At2g26140	Ftsh Protease, Putative	0.0187	0.0299	6.43
254181_at	At4g23940	Ftsh Protease, Putative	0.0176	0.0291	4.49
250162_at	At5g15250	Ftsh Protease, Putative	0.0397	0.0442	4.75
248303_at	At5g53170	Ftsh Protease, Putative	0.0058	0.0169	3.13
255813_at	At1g19930	Kelch Repeat-Containing F-Box Family Protein	0.0053	0.0163	2.53
258518_at	At3g06570	Kelch Repeat-Containing F-Box Family Protein	0.0186	0.0299	2.57

254548_at	At4g19870	Kelch Repeat-Containing F-Box Family Protein	0.0145	0.0264	8.54
252901_at	At4g39550	Kelch Repeat-Containing F-Box Family Protein	0.0324	0.0396	3.63
248818_at	At5g47040	Lon Protease Homolog 1, Mitochondrial (Lon)	0.0148	0.0267	4.52
264340_at	At1g70280	Nhl Repeat-Containing Protein	0.0027	0.0119	2.55
266459_at	At2g47970	Npl4 Family Protein	0.0000	0.0008	4.05
250041_at	At5g18410	P53 Inducible protein	0.0090	0.0208	3.92
245989_s_at	At5g20620	Polyubiquitin (Ubq4)	0.0030	0.0128	2.89
250377_at	At5g11560	Pqq Enzyme Repeat-Containing Protein	0.0033	0.0132	3.05
264807_at	At1g08700	Presenilin Family Protein	0.0046	0.0152	2.88
267015_at	At2g39340	Sac3/Ganp Family Protein	0.0027	0.0119	2.70
262334_at	At1g64010	Serpin, Putative / Serine Protease Inhibitor, Putative	0.0453	0.0476	2.90
261224_at	At1g20160	Subtilase Family Protein	0.0015	0.0092	3.58
245088_at	At2g39850	Subtilase Family Protein	0.0075	0.0190	5.96
258368_at	At3g14240	Subtilase Family Protein	0.0345	0.0409	3.39
254466_at	At4g20430	Subtilase Family Protein	0.0252	0.0349	4.60
254433_at	At4g20850	Subtilase Family Protein	0.0103	0.0222	3.87
254377_at	At4g21650	Subtilase Family Protein	0.0212	0.0318	4.56
248961_at	At5g45650	Subtilase Family Protein	0.0293	0.0376	4.86
247760_at	At5g59130	Subtilase Family Protein	0.0391	0.0438	2.80
263406_at	At2g04160	Subtilisin-Like Protease (Air3)	0.0020	0.0103	5.61
263302_at	At2g15190	Ulp1 Protease Family	0.0101	0.0220	21.63
255661_at	At4g00690	Ulp1 Protease Family Protein	0.0446	0.0472	3.17
H1. Ubiquitin like conjugating enzyme activity					
267301_at	At2g30110	Ubiquitin Activating Enzyme 1 (Uba1)	0.0028	0.0120	3.74
253542_at	At4g31670	Ubiquitin Carboxyl-Terminal Hydrolase Family Protein	0.0334	0.0401	3.94
266248_at	At2g27650	Ubiquitin Carboxyl-Terminal Hydrolase-Related	0.0108	0.0228	4.13
252392_s_at	At3g47890	Ubiquitin Carboxyl-Terminal Hydrolase-Related	0.0212	0.0319	10.55
263197_at	At1g53930	Ubiquitin Family Protein	0.0036	0.0137	10.60
263422_s_at	At2g17200	Ubiquitin Family Protein	0.0290	0.0374	2.60
249240_at	At5g42220	Ubiquitin Family Protein	0.0000	0.0014	6.03
266776_at	At2g29070	Ubiquitin Fusion Degradation Ufd1 Family Protein	0.0137	0.0257	7.06
260839_at	At1g43690	Ubiquitin Interaction Motif-Containing Protein	0.0445	0.0471	3.39
261153_at	At1g04850	Ubiquitin-Associated (Uba)/Ts-N Domain-Containing Protein	0.0012	0.0083	3.62
267598_at	At2g33010	Ubiquitin-Associated (Uba)/Ts-N Domain-Containing Protein	0.0452	0.0475	4.84
267456_at	At2g33770	Ubiquitin-Conjugating Enzyme Family Protein	0.0035	0.0135	3.10
264335_s_at	At1g70320	Ubiquitin-Protein Ligase 2 (Upl2)	0.0009	0.0072	3.37
250693_at	At5g06600	Ubiquitin-Specific Protease 12 (Ubp12)	0.0225	0.0329	3.11
257085_at	At3g20630	Ubiquitin-Specific Protease 14, Putative (Ubp14)	0.0342	0.0407	3.58
247838_at	At5g57990	Ubiquitin-Specific Protease 23, Putative (Ubp23)	0.0008	0.0071	2.95
252275_at	At3g49600	Ubiquitin-Specific Protease 26 (Ubp26)	0.0024	0.0111	2.55
I. Transcription/splicing/RNA processing/modification					
254073_at	At4g25500	Arginine/Serine-Rich Splicing Factor Rsp40 (Rsp40)	0.0017	0.0097	3.39
248369_at	At5g52040	Arginine/Serine-Rich Splicing Factor Rsp41 (Rsp41)	0.0001	0.0019	3.85
262246_at	At1g48410	Argonaute Protein (Ago1)	0.0273	0.0363	3.28
245748_at	At1g51140	Basic Helix-Loop-Helix (Bhlh) Family Protein	0.0067	0.0180	3.51
267628_at	At2g42280	Basic Helix-Loop-Helix (Bhlh) Family Protein	0.0137	0.0257	10.59
257990_at	At3g19860	Basic Helix-Loop-Helix (Bhlh) Family Protein	0.0101	0.0220	2.60
251299_at	At3g61950	Basic Helix-Loop-Helix (Bhlh) Family Protein	0.0000	0.0003	6.39
250569_at	At5g08130	Basic Helix-Loop-Helix (Bhlh) Family Protein	0.0042	0.0147	2.81
248247_at	At5g53210	Basic Helix-Loop-Helix (Bhlh) Family Protein	0.0157	0.0276	4.34
260230_at	At1g74500	BHLH Family Protein	0.0078	0.0193	12.16
247199_at	At5g65210	Bzip Family Transcription Factor (Tga1)	0.0192	0.0303	5.66
252969_at	At4g38900	Bzip Protein	0.0022	0.0107	4.42
245092_at	At2g40950	Bzip Transcription Factor Family Protein	0.0120	0.0241	2.88
258759_at	At3g10800	Bzip Transcription Factor Family Protein	0.0247	0.0345	3.05
251702_at	At3g56660	Bzip Transcription Factor Family Protein	0.0057	0.0169	5.37
250671_at	At5g06950	Bzip Transcription Factor Hbp-1B Homolog	0.0001	0.0023	3.25
263955_at	At2g36010	E2F Transcription Factor-3 (E2F3)	0.0030	0.0127	3.80
251042_at	At5g02310	Eceriferum3 Protein, Putative	0.0106	0.0226	2.54
261701_at	At1g32750	Hac13 Protein (Hac13)	0.0011	0.0081	3.37
254964_at	At4g11080	High Mobility Group (Hmg1/2) Family Protein	0.0137	0.0257	25.63
260166_at	At1g79840	Homeobox-Leucine Zipper Protein 10 (Hb-10) / Hd-Zip Transcription Factor 10	0.0169	0.0284	8.84
266327_at	At2g46680	Homeobox-Leucine Zipper Protein 7 (Hb-7) / Hd-Zip Transcription Factor 7	0.0104	0.0223	3.68
253402_at	At4g32880	Homeobox-Leucine Zipper Transcription Factor (Hb-8)	0.0144	0.0263	13.51
250244_at	At5g13680	Iki3 Family Protein	0.0466	0.0482	3.48
266859_at	At2g26880	Mads-Box Family Protein	0.0367	0.0423	5.69
245819_at	At1g26310	Mads-Box Protein, Putative	0.0444	0.0471	3.56
264093_at	At1g79220	Mitochondrial Transcription Termination Factor Family Protein / Mterf Family Protein	0.0316	0.0390	4.42

250554_at	At5g07900	Mitochondrial Transcription Termination Factor Family Protein / Mterf Family Protein	0.0456	0.0478	2.94
266899_at	At2g34620	Mitochondrial Transcription Termination Factor-Related / Mterf-Related	0.0137	0.0257	2.65
245565_at	At4g14605	Mitochondrial Transcription Termination Factor-Related / Mterf-Related	0.0483	0.0492	6.80
264709_at	At1g09770	Myb Family Transcription Factor	0.0022	0.0107	3.55
259365_at	At1g13300	Myb Family Transcription Factor	0.0012	0.0081	17.63
256198_at	At1g58220	Myb Family Transcription Factor	0.0437	0.0466	2.66
258626_at	At3g04450	Myb Family Transcription Factor	0.0293	0.0377	3.03
258961_at	At3g10580	Myb Family Transcription Factor	0.0117	0.0238	6.99
246790_at	At5g27610	Myb Family Transcription Factor	0.0059	0.0170	4.45
261491_at	At1g14350	Myb Family Transcription Factor (Myb124)	0.0043	0.0148	4.23
247549_at	At5g61420	Myb Family Transcription Factor (Myb28)	0.0461	0.0479	3.86
266719_at	At2g46830	Myb-Related Transcription Factor (Cca1)	0.0008	0.0070	2.77
264460_at	At1g10170	Nf-X1 Type Zinc Finger Family Protein	0.0009	0.0071	3.63
259117_at	At3g01320	Paired Amphipathic Helix Repeat-Containing Protein	0.0125	0.0246	2.60
264858_at	At1g24190	Paired Amphipathic Helix Repeat-Containing Protein	0.0002	0.0038	7.08
258892_at	At3g05670	Phd Finger Family Protein	0.0034	0.0134	2.70
255472_at	At4g02430	Pre-Mrna Splicing Factor, Putative / Sr1 Protein, Putative	0.0030	0.0127	3.96
265125_at	At1g55410	Pseudogene, Chp-Rich Zinc Finger Protein, Putative	0.0494	0.0497	6.82
264781_at	At1g08540	Rna Polymerase Sigma Subunit Sigb (Sigb) / Sigma Factor 2 (Sig2)	0.0147	0.0266	5.68
250255_at	At5g13730	Rna Polymerase Sigma Subunit Sigd (Sigd) / Sigma-Like Factor (Sig4)	0.0000	0.0017	3.63
246207_at	At4g36960	Rna Recognition Motif (Rrm)-Containing Protein	0.0186	0.0299	3.08
248869_at	At5g46840	Rna Recognition Motif (Rrm)-Containing Protein	0.0012	0.0084	3.11
250447_at	At5g10800	Rna Recognition Motif (Rrm)-Containing Protein	0.0346	0.0410	2.57
255598_at	At4g00830	Rna Recognition Motif (Rrm)-Containing Protein	0.0070	0.0184	2.78
258316_at	At3g22660	Rrna Processing Protein-Related	0.0082	0.0199	2.89
246447_at	At5g16780	Sart-1 Family Protein	0.0080	0.0196	4.73
245247_at	At4g17230	Scarecrow-Like Transcription Factor 13 (Scl13)	0.0434	0.0464	2.92
252173_at	At3g50650	Scarecrow-Like Transcription Factor 7 (Scl7)	0.0011	0.0079	3.36
246140_at	At5g19910	Soh1 Family Protein	0.0268	0.0360	4.17
257267_at	At3g15030	Tcp Family Transcription Factor, Putative	0.0026	0.0118	15.34
262096_at	At1g56010	Transcription Activator Nac1 (Nac1)	0.0092	0.0209	6.21
253065_at	At4g37740	Transcription factor GRL2	0.0388	0.0436	4.63
261079_s_at	At1g07470	Transcription Factor Iia Large Subunit, Putative / Tfiia Large Subunit, Putative	0.0099	0.0217	2.62
246255_at	At4g36650	Transcription Factor Iib (Tfiib) Family Protein	0.0082	0.0198	2.97
264792_at	At1g08620	Transcription Factor Jumonji (Jmj) Family Protein	0.0239	0.0339	2.96
256258_at	At3g12480	Transcription Factor, Putative	0.0058	0.0169	2.51
257524_at	At3g01330	Transcription Factor, Putative / E2F-Like Repressor E2L2 (E2L2)	0.0004	0.0049	3.77
256695_at	At3g32090	Transcription Factor-Related	0.0145	0.0264	3.66
260374_at	At1g73960	Transcription initiation factor	0.0045	0.0151	2.53
249133_at	At5g43130	Transcription Initiation Factor Iid (Tfiid) Component Taf4 Family Protein	0.0099	0.0218	3.55
261041_at	At1g17440	Transcription Initiation Factor Iid (Tfiid) Subunit A Family Protein	0.0445	0.0471	7.15
266605_at	At2g46020	Transcription Regulatory Protein Snf2, Putative	0.0003	0.0040	3.42
245288_at	At4g16420	Transcriptional Adaptor (Ada2B)	0.0429	0.0461	2.51
245237_at	At4g25520	Transcriptional Co-Regulator Family Protein	0.0008	0.0069	2.94
253540_at	At4g31620	Transcriptional Factor B3 Family Protein	0.0032	0.0131	9.27
253501_at	At4g32010	Transcriptional Factor B3 Family Protein	0.0045	0.0151	3.86
254992_at	At4g10710	Transcriptional Regulator-Related	0.0029	0.0123	4.17
261429_at	At1g18860	Wrky Family Transcription Factor	0.0187	0.0299	16.45
265718_at	At2g03340	Wrky Family Transcription Factor	0.0498	0.0499	2.60
258975_at	At3g01970	Wrky Family Transcription Factor	0.0386	0.0435	3.14
254347_at	At4g22070	Wrky Family Transcription Factor	0.0004	0.0048	15.17
253983_at	At4g26640	Wrky Family Transcription Factor	0.0007	0.0068	2.59
260266_at	At1g68520	Zinc Finger (B-Box Type) Family Protein	0.0019	0.0102	3.20
252236_at	At3g49930	Zinc Finger (C2H2 Type) Family Protein	0.0000	0.0015	10.50
255491_at	At4g02670	Zinc Finger (C2H2 Type) Family Protein	0.0408	0.0448	5.31
253526_at	At4g31420	Zinc Finger (C2H2 Type) Family Protein	0.0005	0.0057	2.57
245691_at	At5g04240	Zinc Finger (C2H2 Type) Family Protein	0.0134	0.0255	3.29
263908_at	At2g36480	Zinc Finger (C2H2-Type) Family Protein	0.0017	0.0095	6.85
252401_at	At3g48030	Zinc Finger (C3Hc4-Type Ring Finger) Family Protein	0.0053	0.0163	4.29
265086_at	At1g03770	Zinc Finger (C3Hc4-Type Ring Finger) Family Protein	0.0068	0.0181	4.43
260823_at	At1g06770	Zinc Finger (C3Hc4-Type Ring Finger) Family Protein	0.0081	0.0198	10.32
259662_at	At1g55255	Zinc Finger (C3Hc4-Type Ring Finger) Family Protein	0.0157	0.0275	3.62
245828_at	At1g57820	Zinc Finger (C3Hc4-Type Ring Finger) Family Protein	0.0071	0.0185	4.35
262302_at	At1g70910	Zinc Finger (C3Hc4-Type Ring Finger) Family Protein	0.0002	0.0037	5.60
264854_at	At2g17450	Zinc Finger (C3Hc4-Type Ring Finger) Family Protein	0.0008	0.0071	2.53
263462_at	At2g31780	Zinc Finger (C3Hc4-Type Ring Finger) Family Protein	0.0134	0.0255	12.68
245059_at	At2g39720	Zinc Finger (C3Hc4-Type Ring Finger) Family Protein	0.0037	0.0139	3.28
267539_at	At2g42030	Zinc Finger (C3Hc4-Type Ring Finger) Family Protein	0.0084	0.0201	3.41
258758_at	At3g10810	Zinc Finger (C3Hc4-Type Ring Finger) Family Protein	0.0469	0.0483	2.71
251708_at	At3g56580	Zinc Finger (C3Hc4-Type Ring Finger) Family Protein	0.0136	0.0256	3.24
251454_at	At3g60080	Zinc Finger (C3Hc4-Type Ring Finger) Family Protein	0.0152	0.0271	3.01
255075_at	At4g09110	Zinc Finger (C3Hc4-Type Ring Finger) Family Protein	0.0290	0.0374	10.83
245264_at	At4g17245	Zinc Finger (C3Hc4-Type Ring Finger) Family Protein	0.0155	0.0274	4.87

253267_at	At4g34100	Zinc Finger (C3Hc4-Type Ring Finger) Family Protein	0.0256	0.0352	3.70
246968_at	At5g24870	Zinc Finger (C3Hc4-Type Ring Finger) Family Protein	0.0104	0.0223	4.57
266695_at	At2g19810	Zinc Finger (Ccch-Type) Family Protein	0.0177	0.0291	3.38
266656_at	At2g25900	Zinc Finger (Ccch-Type) Family Protein	0.0004	0.0051	3.78
265274_at	At2g28450	Zinc Finger (Ccch-Type) Family Protein	0.0312	0.0388	3.21
250321_at	At5g12850	Zinc Finger (Ccch-Type) Family Protein	0.0066	0.0179	2.77
261082_at	At1g07360	Zinc Finger (Ccch-Type) Family Protein	0.0013	0.0087	3.14
250736_s_at	At5g06420	Zinc Finger (Ccch-Type/C3Hc4-Type Ring Finger) Family Protein	0.0357	0.0416	4.31
254142_at	At4g24630	Zinc Finger (Dhhc Type) Family Protein	0.0050	0.0158	17.09
261231_at	At1g20110	Zinc Finger (Fyve Type) Family Protein	0.0138	0.0257	3.22
266125_at	At2g45050	Zinc Finger (Gata Type) Family Protein	0.0033	0.0132	2.80
252129_at	At3g50890	Zinc Finger Homeobox Family Protein / Zf-Hd Homeobox Family Protein	0.0490	0.0495	3.27
246523_at	At5g15850	Zinc Finger Protein Constans-Like 1 (Col1)	0.0169	0.0284	4.93
258497_at	At3g02380	Zinc Finger Protein Constans-Like 2 (Col2)	0.0021	0.0105	3.73
260484_at	At1g68360	Zinc Finger Protein-Related	0.0006	0.0063	6.87
257062_at	At3g18290	Zinc Finger Protein-Related	0.0073	0.0187	3.11
264633_at	At1g65660	Zinc Knuckle (Cchc-Type) Family Protein	0.0002	0.0035	4.98
I1. Helicase activity					
257310_at	At3g26560	Atp-Dependent Rna Helicase, Putative	0.0000	0.0004	6.23
247465_at	At5g62190	Dead Box Rna Helicase (Prh75)	0.0045	0.0151	3.10
262851_at	At1g20920	Dead Box Rna Helicase, Putative	0.0074	0.0188	2.75
266932_s_at	At2g07750	Dead Box Rna Helicase, Putative	0.0133	0.0254	3.83
267454_at	At2g33730	Dead Box Rna Helicase, Putative	0.0016	0.0095	4.28
247564_at	At5g61140	Dead Box Rna Helicase, Putative	0.0158	0.0277	2.84
251534_at	At3g58510	Dead Box Rna Helicase, Putative (Rh11)	0.0007	0.0064	2.52
261584_at	At1g01040	Dead/Deah Box Helicase Carpel Factory / Caf	0.0456	0.0478	3.60
258554_at	At3g06980	Dead/Deah Box Helicase, Putative	0.0004	0.0053	2.62
252443_at	At3g46960	Dead/Deah Box Helicase, Putative	0.0013	0.0084	2.58
251362_at	At3g61240	Dead/Deah Box Helicase, Putative (Rh12)	0.0033	0.0133	2.80
266501_at	At2g06990	Dead-Box Rna Helicase, Putative	0.0334	0.0401	3.80
262636_at	At1g06670	Helicase	0.0015	0.0092	13.37
256140_at	At1g48650	Helicase Domain-Containing Protein	0.0263	0.0356	4.30
263936_at	At2g35920	Helicase Domain-Containing Protein	0.0316	0.0391	2.69
255600_s_at	At5g10370	Helicase Domain-Containing Protein / Ibr Domain-Containing Protein	0.0048	0.0155	35.87
256082_at	At1g20720	Helicase-Related	0.0370	0.0424	3.70
260695_at	At1g32490	RNA Helicase, Putative	0.0005	0.0055	3.99
254675_at	At4g18465	RNA Helicase, Putative	0.0000	0.0001	3.92
250273_at	At5g13010	RNA Helicase, Putative	0.0011	0.0078	3.07
251876_at	At3g54280	Snf2 Domain-Containing Protein / Helicase Domain-Containing Protein	0.0143	0.0262	2.73
262800_at	At1g20960	U5 Small Nuclear Ribonucleoprotein Helicase, Putative	0.0015	0.0091	7.21
I2. RNA metabolism					
262468_at	At1g50200	Aminoacyl-Trna Synthetase Family Protein	0.0013	0.0087	3.53
253935_at	At4g26870	Aspartyl-Trna Synthetase, Putative / Aspartate--Trna Ligase, Putative	0.0025	0.0116	3.38
261887_at	At1g80780	Ccr4-Not Transcription Complex Protein, Putative	0.0192	0.0303	2.86
245631_at	At1g25350	Glutamine-Trna Ligase, Putative	0.0032	0.0131	4.43
256000_at	At1g29880	Glycyl-Trna Synthetase / Glycine--Trna Ligase	0.0010	0.0077	3.75
258601_at	At3g02760	Histidyl-Trna Synthetase, Putative / Histidine--Trna Ligase, Putative	0.0031	0.0128	3.01
260021_at	At1g30010	Intron Maturase, Type Ii Family Protein	0.0172	0.0286	5.46
260256_at	At1g74350	Intron Maturase, Type Ii Family Protein	0.0282	0.0370	5.24
252941_at	At4g39280	Phenylalanyl-Trna Synthetase, Putative / Phenylalanine--Trna Ligase, Putative	0.0001	0.0030	2.68
261614_at	At1g49760	Polyadenylate-Binding Protein, Putative / Pabp, Putative	0.0151	0.0270	3.57
245085_at	At2g23350	Polyadenylate-Binding Protein, Putative / Pabp, Putative	0.0367	0.0423	3.63
259377_at	At3g16380	Polyadenylate-Binding Protein, Putative / Pabp, Putative	0.0002	0.0033	6.59
249821_at	At5g23690	Polynucleotide Adenylyltransferase Family Protein	0.0211	0.0318	8.81
259344_at	At3g03710	Polyribonucleotide Nucleotidyltransferase, Putative	0.0460	0.0479	5.94
258034_at	At3g21300	Rna Methyltransferase Family Protein	0.0063	0.0175	9.50
246780_at	At5g27470	Seryl-Trna Synthetase / Serine--Trna Ligase	0.0000	0.0011	3.64
264350_at	At1g11870	Seryl-Trna Synthetase, Putative / Serine--Trna Ligase, Putative	0.0000	0.0004	3.58
254475_at	At4g20440	Small Nuclear Ribonucleoprotein Associated Protein B, Putative	0.0247	0.0345	22.79
266005_at	At2g37340	Splicing Factor Rsz33 (Rsz33)	0.0050	0.0158	2.69
247271_at	At5g64270	Splicing Factor, Putative	0.0087	0.0204	4.44
251837_s_at	At3g55200	Splicing Factor, Putative	0.0044	0.0150	4.10
248452_at	At5g51300	Splicing Factor-Related	0.0000	0.0018	3.66
248106_at	At5g55100	Swap (Suppressor-Of-White-Apricot)/Surp Domain-Containing Protein	0.0026	0.0117	14.36
253565_at	At4g31200	Swap (Suppressor-Of-White-Apricot)/Surp Domain-Containing Protein	0.0107	0.0226	2.61
260779_at	At1g14650	Swap (Suppressor-Of-White-Apricot)/Surp Domain-Containing Protein	0.0474	0.0486	3.64
253295_at	At4g33760	tRNA Synthetase Class I (D, K And N) Family Protein	0.0158	0.0276	2.90
251264_at	At3g62120	tRNA Synthetase Class I (G, H, P And S) Family Protein	0.0001	0.0020	2.63
248339_at	At5g52520	tRNA Synthetase Class I (G, H, P And S) Family Protein	0.0006	0.0060	4.08
264705_at	At1g09620	tRNA Synthetase Class I (I, L, M And V) Family Protein	0.0185	0.0298	4.36
249630_s_at	At5g37150	tRNA-Splicing Endonuclease Positive Effector-Related	0.0001	0.0026	6.81

267489_s_at	At2g19120	tRNA-Splicing Endonuclease Positive Effector-Related	0.0061	0.0173	5.66
246257_at	At4g36690	U2 Snrnp Auxiliary Factor Large Subunit, Putative	0.0071	0.0185	4.05
264922_s_at	At1g60830	U2 Snrnp Auxiliary Factor Large Subunit, Putative	0.0012	0.0082	2.61
250528_at	At5g08600	U3 Ribonucleoprotein (Utp) Family Protein	0.0022	0.0107	4.20
255501_at	At4g02400	U3 Ribonucleoprotein (Utp) Family Protein	0.0060	0.0172	2.82
260780_at	At1g14610	Valyl-Trna Synthetase / Valine--Trna Ligase (Valrs)	0.0000	0.0017	4.20
J. Transporters					
262005_at	At1g64550	ABC Transporter Family Protein	0.0078	0.0193	4.88
266038_at	At2g07680	ABC Transporter Family Protein	0.0112	0.0232	3.19
258916_at	At3g10670	ABC Transporter Family Protein	0.0039	0.0142	3.29
258033_at	At3g21250	ABC Transporter Family Protein	0.0352	0.0414	2.66
252379_at	At3g47730	ABC Transporter Family Protein	0.0044	0.0150	2.68
251845_at	At3g54540	ABC Transporter Family Protein	0.0027	0.0120	4.97
251781_at	At3g55320	ABC Transporter Family Protein	0.0209	0.0317	2.84
253863_at	At4g27420	ABC Transporter Family Protein	0.0178	0.0292	4.16
247222_at	At5g64840	ABC Transporter Family Protein	0.0004	0.0051	4.34
261353_at	At1g79600	ABC1 Family Protein	0.0046	0.0152	4.29
257253_at	At3g24190	ABC1 Family Protein	0.0106	0.0225	3.34
246973_at	At5g24970	ABC1 Family Protein	0.0212	0.0318	4.01
248554_at	At5g50330	ABC1 Family Protein	0.0002	0.0033	10.30
247232_at	At5g64940	ABC1 Family Protein	0.0076	0.0191	2.55
253006_at	At4g38250	Amino Acid Transporter Family Protein	0.0013	0.0084	4.26
247120_at	At5g65990	Amino Acid Transporter Family Protein	0.0239	0.0339	6.19
254723_at	At4g13510	Ammonium Transporter 1, Member 1 (Amt1.1)	0.0347	0.0410	3.43
262883_at	At1g64780	Ammonium Transporter 1, Member 2 (Amt1.2)	0.0317	0.0391	2.85
267142_at	At2g38290	Ammonium Transporter 2 (Amt2)	0.0041	0.0145	2.71
259514_at	At1g12480	C4-Dicarboxylate Transporter/Malic Acid Transport Family Protein	0.0011	0.0080	10.98
249765_at	At5g24030	C4-Dicarboxylate Transporter/Malic Acid Transport Family Protein	0.0030	0.0127	3.47
251053_at	At5g01490	Cation Exchanger, Putative (Cax4)	0.0394	0.0440	3.95
267275_at	At2g30240	Cation/Hydrogen Exchanger, Putative (Chx13)	0.0032	0.0130	2.57
252087_at	At3g52080	Cation/Hydrogen Exchanger, Putative (Chx28)	0.0239	0.0339	8.51
262757_at	At1g16380	Cation/Proton Exchanger, Putative (Chx1)	0.0414	0.0452	3.69
256751_at	At3g27170	Chloride Channel Protein (Clc-B)	0.0024	0.0114	3.64
246510_at	At5g15410	Cyclic Nucleotide-Regulated Ion Channel / Cyclic Nucleotide-Gated Channel (Cngc2)	0.0394	0.0439	3.15
261142_at	At1g19780	Cyclic Nucleotide-Regulated Ion Channel, Putative (Cngc8)	0.0013	0.0086	8.63
256305_at	At1g30400	Glutathione S-Conjugate Abc Transporter (Mrp1)	0.0002	0.0035	4.59
267319_at	At2g34660	Glutathione S-Conjugate Abc Transporter (Mrp2)	0.0109	0.0229	3.74
266464_at	At2g47800	Glutathione-Conjugate Transporter (Mrp4)	0.0211	0.0318	8.79
251227_at	At3g62700	Glutathione-Conjugate Transporter, Putative	0.0196	0.0306	4.95
248338_at	At5g52440	Hcf106 Protein	0.0003	0.0040	2.64
255686_at	At4g00630	K+ Efflux Antiporter, Putative (Kea2)	0.0068	0.0181	3.49
265424_at	At2g20780	Mannitol Transporter, Putative	0.0007	0.0068	2.76
258693_at	At3g08650	Metal Transporter Family Protein	0.0068	0.0181	3.54
250206_at	At5g14040	Mitochondrial Phosphate Transporter	0.0053	0.0163	2.50
266752_at	At2g47000	Multidrug Resistant (Mdr) Abc Transporter, Putative	0.0154	0.0273	3.35
264456_at	At1g10390	Nucleoporin Family Protein	0.0273	0.0363	2.99
245116_at	At2g41620	Nucleoporin Interacting Component Family Protein	0.0001	0.0030	2.91
246112_at	At5g20200	Nucleoporin-Related	0.0044	0.0149	2.72
261074_at	At1g07290	Nucleotide-Sugar Transporter Family Protein	0.0103	0.0222	9.90
257789_at	At3g27020	Oligopeptide Transporter Opt Family Protein	0.0486	0.0494	3.96
254938_at	At4g10770	Oligopeptide Transporter Opt Family Protein	0.0316	0.0391	2.97
245296_at	At4g16370	Oligopeptide Transporter Opt Family Protein	0.0042	0.0146	3.53
247286_at	At5g64280	Oxoglutarate/Malate Translocator, Putative	0.0148	0.0267	4.47
247289_at	At5g64290	Oxoglutarate/Malate Translocator, Putative	0.0214	0.0319	4.90
250278_at	At5g12860	Oxoglutarate/Malate Translocator, Putative	0.0000	0.0011	5.12
253427_at	At4g32390	Phosphate Translocator-Related	0.0458	0.0479	2.67
257311_at	At3g26570	Phosphate Transporter Family Protein	0.0036	0.0138	3.78
267423_at	At2g35060	Potassium Transporter Family Protein	0.0206	0.0315	3.09
264338_at	At1g70300	Potassium Transporter, Putative	0.0008	0.0069	3.34
258829_at	At3g07100	Protein Transport Protein Sec24, Putative	0.0329	0.0399	4.39
248344_at	At5g52280	Protein Transport Protein-Related	0.0001	0.0027	3.25
261425_at	At1g18880	Proton-Dependent Oligopeptide Transport (Pot) Family Protein	0.0108	0.0228	2.99
260693_at	At1g32450	Proton-Dependent Oligopeptide Transport (Pot) Family Protein	0.0001	0.0031	4.86
262281_at	At1g68570	Proton-Dependent Oligopeptide Transport (Pot) Family Protein	0.0025	0.0115	7.16
252594_at	At3g45680	Proton-Dependent Oligopeptide Transport (Pot) Family Protein	0.0347	0.0411	2.77
252525_at	At3g46450	Sec14 Cytosolic Factor Family Protein / Phosphoglyceride Transfer Family Protein	0.0001	0.0031	3.09
259803_at	At1g72150	Sec14 Cytosolic Factor Family Protein / Phosphoglyceride Transfer Family Protein	0.0048	0.0155	3.43
259804_at	At1g72160	Sec14 Cytosolic Factor Family Protein / Phosphoglyceride Transfer Family Protein	0.0442	0.0469	3.70
252884_at	At4g39170	Sec14 Cytosolic Factor, Putative / Phosphoglyceride Transfer Protein, Putative	0.0025	0.0116	2.71
252662_at	At3g44340	Sec23/Sec24 Transport Family Protein	0.0325	0.0397	2.68
248084_at	At5g55470	Sodium Proton Exchanger / Na+/H+ Exchanger 4 (Nhx4)	0.0000	0.0004	7.42
248756_at	At5g47560	Sodium/Dicarboxylate Cotransporter, Putative	0.0069	0.0182	3.27

264204_at	At1g22710	Sucrose Transporter / Sucrose-Proton Symporter (Suc2)	0.0286	0.0372	4.06
260170_at	At1g71890	Sucrose Transporter / Sucrose-Proton Symporter (Suc5)	0.0296	0.0379	3.33
245499_at	At4g16480	Sugar Transporter Family Protein	0.0015	0.0091	2.56
246310_at	At3g51895	Sulfate Transporter (St1)	0.0003	0.0045	2.74
251504_at	At3g59030	Transparent Testa 12 Protein (Tt12) / Multidrug Transporter-Like Protein	0.0426	0.0459	4.55
251187_at	At3g62770	Transport Protein-Related	0.0063	0.0175	2.62
263104_at	At2g05120	Transporter	0.0033	0.0132	17.23
262797_at	At1g20840	Transporter-Related	0.0003	0.0042	5.16
253188_at	At4g35300	Transporter-Related	0.0037	0.0140	3.87
246122_at	At5g20380	Transporter-Related	0.0430	0.0462	3.24
264419_s_at	At1g43310	Triose Phosphate/Phosphate Translocator-Related	0.0477	0.0487	4.32
266336_at	At2g32270	Zinc Transporter (Zip3)	0.0423	0.0456	3.85
251545_at	At3g58810	Zinc Transporter, Putative	0.0070	0.0184	6.70
K. Transposable elements, viral and plasmid proteins					
265757_x_at	At2g13160	Cacta-Like Transposase Family (Ptta/En/Spm)	0.0031	0.0128	5.39
255322_at	At4g04270	Cacta-Like Transposase Family (Ptta/En/Spm)	0.0288	0.0373	6.72
255034_at	At4g09540	Copia-Like Retrotransposon Family	0.0002	0.0033	6.43
249114_at	At5g43800	Copia-Like Retrotransposon Family	0.0293	0.0377	6.59
263186_at	At1g36110	Copia-Like Retrotransposon Family	0.0121	0.0242	3.18
260866_at	At1g43886	Copia-Like Retrotransposon Family	0.0103	0.0222	3.53
266432_s_at	At2g07080	Copia-Like Retrotransposon Family	0.0001	0.0031	2.76
254985_x_at	At4g10580	Gypsy-Like Retrotransposon Family	0.0098	0.0217	5.96
255320_at	At4g04230	Gypsy-Like Retrotransposon Family	0.0018	0.0098	24.41
260016_at	At1g41795	Gypsy-Like Retrotransposon Family (Athila)	0.0060	0.0172	8.31
252847_at	At3g42170	Hat-Like Transposase Family (Hobo/Ac/Tam3)	0.0098	0.0217	3.22
261977_at	At1g37057	Hat-Like Transposase Family (Hobo/Ac/Tam3)	0.0426	0.0459	4.34
265744_at	At2g06660	Mariner-Like Transposase Family	0.0077	0.0193	2.98
257344_s_at	At1g45070	Mutator-Like Transposase Family	0.0500	0.0500	3.37
258550_at	At3g06940	Mutator-Like Transposase Family	0.0094	0.0211	2.94
258733_at	At3g05850	Mutator-Like Transposase Family	0.0001	0.0029	14.71
264411_at	At1g43240	Mutator-Like Transposase Family	0.0479	0.0489	3.30
265294_at	At2g14030	Mutator-Like Transposase Family	0.0472	0.0485	3.22
266382_at	At2g14595	Mutator-Like Transposase Family	0.0158	0.0276	3.84
259456_s_at	At1g43995	Non-LTR Retroelement reverse transcriptase	0.0121	0.0242	6.74
265497_at	At2g15720	Non-Ltr Retrotransposon Family (Line)	0.0000	0.0011	6.94
246193_at	At5g20880	Retrotransposon Family	0.0020	0.0104	2.55
L. Unknown proteins					
261049_at	At1g01430	Expressed Protein	0.0070	0.0183	4.52
261558_at	At1g01770	Expressed Protein	0.0058	0.0169	3.11
259415_at	At1g02330	Expressed Protein	0.0063	0.0175	4.06
264824_at	At1g03420	Expressed Protein	0.0002	0.0034	3.85
264326_at	At1g04230	Expressed Protein	0.0008	0.0071	2.52
262617_at	At1g06590	Expressed Protein	0.0070	0.0184	2.72
261742_at	At1g08390	Expressed Protein	0.0044	0.0150	3.34
264265_at	At1g09280	Expressed Protein	0.0043	0.0148	3.35
263709_at	At1g09310	Expressed Protein	0.0013	0.0086	2.76
264559_at	At1g09610	Expressed Protein	0.0062	0.0174	7.73
264658_at	At1g09910	Expressed Protein	0.0419	0.0455	2.70
264667_s_at	At1g09980	Expressed Protein	0.0218	0.0323	2.63
264407_at	At1g10180	Expressed Protein	0.0129	0.0251	2.64
264450_s_at	At1g10250	Expressed Protein	0.0042	0.0147	4.78
257450_at	At1g10530	Expressed Protein	0.0008	0.0071	13.47
261833_at	At1g10670	Expressed Protein	0.0026	0.0118	3.12
262788_at	At1g10690	Expressed Protein	0.0001	0.0026	25.40
260470_at	At1g11120	Expressed Protein	0.0352	0.0414	3.35
260992_at	At1g12150	Expressed Protein	0.0129	0.0250	18.79
262663_at	At1g13940	Expressed Protein	0.0035	0.0135	4.06
259488_at	At1g15780	Expressed Protein	0.0079	0.0194	6.99
261844_at	At1g15940	Expressed Protein	0.0308	0.0386	8.79
262710_at	At1g16210	Expressed Protein	0.0013	0.0087	9.84
255774_at	At1g18620	Expressed Protein	0.0161	0.0279	2.57
260665_at	At1g19360	Expressed Protein	0.0021	0.0105	2.68
261131_at	At1g19835	Expressed Protein	0.0013	0.0085	4.81
261221_at	At1g19960	Expressed Protein	0.0228	0.0331	2.76
262854_at	At1g20870	Expressed Protein	0.0119	0.0239	3.44
262493_at	At1g21660	Expressed Protein	0.0129	0.0250	3.50
262505_at	At1g21680	Expressed Protein	0.0207	0.0315	3.27
262494_at	At1g21810	Expressed Protein	0.0407	0.0448	4.96
262490_at	At1g21840	Expressed Protein	0.0121	0.0242	2.75
264207_at	At1g22750	Expressed Protein	0.0136	0.0256	3.16
263037_at	At1g23230	Expressed Protein	0.0214	0.0320	3.62
265180_at	At1g23590	Expressed Protein	0.0351	0.0413	11.58

265171_at	At1g23790	Expressed Protein	0.0088	0.0205	9.01
245638_s_at	At1g24996	Expressed Protein	0.0193	0.0303	2.59
245822_at	At1g26110	Expressed Protein	0.0339	0.0405	3.34
262322_at	At1g27590	Expressed Protein	0.0284	0.0371	2.93
245659_at	At1g28260	Expressed Protein	0.0339	0.0405	3.02
261445_at	At1g28380	Expressed Protein	0.0320	0.0393	2.64
259767_s_at	At1g29350	Expressed Protein	0.0000	0.0004	3.11
257419_at	At1g30800	Expressed Protein	0.0453	0.0476	3.52
261238_at	At1g32810	Expressed Protein	0.0041	0.0146	3.48
262562_at	At1g34220	Expressed Protein	0.0064	0.0177	4.53
262407_at	At1g34630	Expressed Protein	0.0302	0.0382	2.93
262014_at	At1g35660	Expressed Protein	0.0218	0.0323	9.96
261293_at	At1g36980	Expressed Protein	0.0189	0.0300	3.07
256543_at	At1g42480	Expressed Protein	0.0030	0.0127	2.71
265092_at	At1g03910	Expressed protein	0.0011	0.0079	3.10
262430_s_at	At1g47550	Expressed Protein	0.0008	0.0071	2.54
259806_at	At1g47900	Expressed Protein	0.0262	0.0356	2.85
261300_at	At1g48560	Expressed Protein	0.0048	0.0155	12.97
261632_at	At1g50120	Expressed Protein	0.0054	0.0164	2.91
261861_at	At1g50450	Expressed Protein	0.0000	0.0016	2.67
260639_at	At1g53180	Expressed Protein	0.0023	0.0109	4.02
260641_at	At1g53200	Expressed Protein	0.0054	0.0164	6.47
259653_at	At1g55240	Expressed Protein	0.0002	0.0036	9.72
245677_at	At1g56660	Expressed Protein	0.0016	0.0093	4.04
264915_at	At1g60790	Expressed Protein	0.0002	0.0036	5.62
265035_at	At1g61620	Expressed Protein	0.0096	0.0215	2.80
261948_at	At1g64680	Expressed Protein	0.0298	0.0379	2.60
245187_s_at	At1g67680	Expressed Protein	0.0157	0.0276	3.69
260031_at	At1g68790	Expressed Protein	0.0088	0.0205	6.49
259370_at	At1g69050	Expressed Protein	0.0467	0.0482	6.57
264701_at	At1g70160	Expressed Protein	0.0307	0.0385	2.57
259756_at	At1g71080	Expressed Protein	0.0001	0.0027	3.53
260377_at	At1g73930	Expressed Protein	0.0283	0.0370	4.72
259903_at	At1g74160	Expressed Protein	0.0080	0.0196	4.09
262967_at	At1g75730	Expressed Protein	0.0239	0.0340	10.56
262678_at	At1g75810	Expressed Protein	0.0233	0.0335	4.95
256329_at	At1g76850	Expressed Protein	0.0017	0.0097	3.35
259762_at	At1g77600	Expressed Protein	0.0188	0.0300	6.34
264126_at	At1g79280	Expressed Protein	0.0274	0.0363	11.06
260162_at	At1g79830	Expressed Protein	0.0001	0.0030	6.71
260302_at	At1g80310	Expressed Protein	0.0000	0.0004	2.68
260275_at	At1g80610	Expressed Protein	0.0017	0.0098	3.11
266795_at	At2g03070	Expressed Protein	0.0259	0.0354	3.28
263857_at	At2g04380	Expressed Protein	0.0004	0.0052	16.68
263100_at	At2g05210	Expressed Protein	0.0058	0.0170	5.78
265519_at	At2g06030	Expressed Protein	0.0031	0.0129	8.19
266427_at	At2g07170	Expressed Protein	0.0122	0.0243	3.65
266046_at	At2g07728	Expressed Protein	0.0236	0.0337	3.93
263567_at	At2g15440	Expressed Protein	0.0001	0.0028	4.12
263097_at	At2g16070	Expressed Protein	0.0196	0.0306	2.57
267278_at	At2g19350	Expressed Protein	0.0237	0.0338	5.64
267282_at	At2g19390	Expressed Protein	0.0256	0.0352	7.18
264024_at	At2g21180	Expressed Protein	0.0039	0.0142	3.87
264023_at	At2g21195	Expressed Protein	0.0031	0.0128	3.27
266001_at	At2g24150	Expressed Protein	0.0022	0.0107	4.22
265281_at	At2g28370	Expressed Protein	0.0300	0.0381	4.73
267193_at	At2g30900	Expressed Protein	0.0076	0.0192	15.18
266473_at	At2g31120	Expressed Protein	0.0095	0.0213	7.24
264115_at	At2g31290	Expressed Protein	0.0247	0.0345	4.13
265679_at	At2g32240	Expressed Protein	0.0209	0.0316	3.02
267642_at	At2g32910	Expressed Protein	0.0101	0.0220	3.93
263915_at	At2g36430	Expressed Protein	0.0147	0.0266	3.16
263910_at	At2g36550	Expressed Protein	0.0010	0.0078	18.71
266007_at	At2g37380	Expressed Protein	0.0002	0.0038	10.51
267180_at	At2g37570	Expressed Protein	0.0332	0.0401	2.50
267359_at	At2g40020	Expressed Protein	0.0024	0.0112	2.53
255874_at	At2g40550	Expressed Protein	0.0423	0.0456	2.70
266047_at	At2g40800	Expressed Protein	0.0186	0.0299	2.77
267583_at	At2g41960	Expressed Protein	0.0157	0.0276	4.86
265883_at	At2g42310	Expressed Protein	0.0387	0.0436	3.93
265854_at	At2g42370	Expressed Protein	0.0011	0.0079	11.91
267341_at	At2g44200	Expressed Protein	0.0048	0.0155	4.35
267343_at	At2g44260	Expressed Protein	0.0205	0.0314	3.72
263781_at	At2g46360	Expressed Protein	0.0326	0.0397	4.84

266483_at	At2g47910	Expressed Protein	0.0012	0.0084	3.19
259118_at	At3g01310	Expressed Protein	0.0269	0.0361	3.17
259180_at	At3g01680	Expressed Protein	0.0056	0.0168	3.52
259190_at	At3g01780	Expressed Protein	0.0140	0.0260	3.00
259132_at	At3g02250	Expressed Protein	0.0000	0.0002	2.57
258611_at	At3g02860	Expressed Protein	0.0043	0.0148	4.43
258875_at	At3g03160	Expressed Protein	0.0281	0.0369	3.41
258796_at	At3g04630	Expressed Protein	0.0020	0.0103	3.06
258562_at	At3g05980	Expressed Protein	0.0230	0.0332	6.23
257580_at	At3g06210	Expressed Protein	0.0440	0.0468	3.34
258836_at	At3g07210	Expressed Protein	0.0175	0.0290	6.24
259017_at	At3g07310	Expressed Protein	0.0095	0.0213	3.93
259015_at	At3g07350	Expressed Protein	0.0021	0.0105	4.41
259245_at	At3g07660	Expressed Protein	0.0152	0.0271	11.50
258946_at	At3g10650	Expressed Protein	0.0236	0.0337	4.05
257849_at	At3g13060	Expressed Protein	0.0017	0.0097	2.88
257708_at	At3g13330	Expressed Protein	0.0003	0.0041	3.11
258209_at	At3g14060	Expressed Protein	0.0240	0.0340	4.27
257063_s_at	At3g18240	Expressed Protein	0.0073	0.0187	3.12
257727_at	At3g18380	Expressed Protein	0.0468	0.0483	3.11
257719_at	At3g18440	Expressed Protein	0.0355	0.0415	2.50
256656_at	At3g18900	Expressed Protein	0.0043	0.0148	2.98
257991_at	At3g19870	Expressed Protein	0.0044	0.0150	3.24
257663_at	At3g20260	Expressed Protein	0.0311	0.0388	12.71
257086_at	At3g20490	Expressed Protein	0.0016	0.0095	7.52
256808_at	At3g21430	Expressed Protein	0.0297	0.0379	7.61
258444_at	At3g22380	Expressed Protein	0.0037	0.0140	2.71
258447_at	At3g22450	Expressed Protein	0.0017	0.0097	12.34
256932_at	At3g22520	Expressed Protein	0.0042	0.0147	6.01
258078_at	At3g25870	Expressed Protein	0.0417	0.0453	10.02
258255_at	At3g26800	Expressed Protein	0.0184	0.0297	2.64
257147_at	At3g27270	Expressed Protein	0.0179	0.0292	2.50
257145_at	At3g27320	Expressed Protein	0.0006	0.0061	4.15
257970_at	At3g27570	Expressed Protein	0.0061	0.0173	2.59
258238_at	At3g27670	Expressed Protein	0.0498	0.0499	2.90
257845_at	At3g28430	Expressed Protein	0.0001	0.0026	7.60
252682_at	At3g44370	Expressed Protein	0.0307	0.0385	3.53
252462_at	At3g47250	Expressed Protein	0.0032	0.0131	2.57
252424_at	At3g47610	Expressed Protein	0.0042	0.0147	5.56
252405_at	At3g48120	Expressed Protein	0.0084	0.0200	3.71
252353_at	At3g48200	Expressed Protein	0.0038	0.0141	3.67
252319_at	At3g48710	Expressed Protein	0.0078	0.0193	25.07
252276_at	At3g49490	Expressed Protein	0.0002	0.0034	2.89
252057_at	At3g52480	Expressed Protein	0.0334	0.0401	8.05
251990_at	At3g53320	Expressed Protein	0.0046	0.0152	8.63
251698_at	At3g56610	Expressed Protein	0.0031	0.0129	5.63
251556_at	At3g58840	Expressed Protein	0.0145	0.0264	2.91
251380_at	At3g60700	Expressed Protein	0.0327	0.0398	5.84
251117_at	At3g63390	Expressed Protein	0.0014	0.0088	3.29
255615_at	At4g01290	Expressed Protein	0.0012	0.0083	6.36
255495_at	At4g02720	Expressed Protein	0.0008	0.0070	8.93
255456_at	At4g02920	Expressed Protein	0.0001	0.0025	2.90
255409_at	At4g03090	Expressed Protein	0.0212	0.0319	7.24
255245_at	At4g05630	Expressed Protein	0.0420	0.0455	7.11
255023_at	At4g09850	Expressed Protein	0.0123	0.0245	4.93
254944_at	At4g10930	Expressed Protein	0.0050	0.0158	6.60
254965_at	At4g11090	Expressed Protein	0.0040	0.0145	5.30
254732_at	At4g13750	Expressed Protein	0.0242	0.0341	10.23
245588_at	At4g15030	Expressed Protein	0.0337	0.0404	2.66
245433_at	At4g17110	Expressed Protein	0.0007	0.0065	3.38
254621_at	At4g18600	Expressed Protein	0.0138	0.0258	2.63
254493_at	At4g20020	Expressed Protein	0.0001	0.0026	3.52
254369_at	At4g21720	Expressed Protein	0.0284	0.0371	3.42
254152_at	At4g24410	Expressed Protein	0.0129	0.0250	7.58
254017_at	At4g26170	Expressed Protein	0.0132	0.0253	14.14
253977_at	At4g26630	Expressed Protein	0.0333	0.0401	13.07
253923_at	At4g27060	Expressed Protein	0.0088	0.0205	4.21
253868_at	At4g27500	Expressed Protein	0.0003	0.0044	3.44
253849_at	At4g28080	Expressed Protein	0.0003	0.0042	6.62
253803_at	At4g28200	Expressed Protein	0.0087	0.0204	2.61
253817_at	At4g28310	Expressed Protein	0.0016	0.0095	3.25
253739_at	At4g28760	Expressed Protein	0.0047	0.0155	4.20
253752_at	At4g28910	Expressed Protein	0.0365	0.0421	4.51
253577_at	At4g31080	Expressed Protein	0.0110	0.0230	2.72

253497_at	At4g31880	Expressed Protein	0.0000	0.0015	2.77
253424_at	At4g32330	Expressed Protein	0.0000	0.0008	4.49
253478_at	At4g32350	Expressed Protein	0.0041	0.0146	3.34
253446_at	At4g32620	Expressed Protein	0.0022	0.0107	3.07
253317_at	At4g33960	Expressed Protein	0.0226	0.0330	2.90
246221_at	At4g37120	Expressed Protein	0.0001	0.0026	3.74
252895_at	At4g39450	Expressed Protein	0.0014	0.0089	3.68
251123_at	At5g01030	Expressed Protein	0.0004	0.0050	2.59
251093_at	At5g01360	Expressed Protein	0.0062	0.0174	7.79
251015_at	At5g02480	Expressed Protein	0.0174	0.0288	3.39
250920_at	At5g03390	Expressed Protein	0.0027	0.0120	5.93
250907_at	At5g03670	Expressed Protein	0.0042	0.0147	10.20
245709_at	At5g04320	Expressed Protein	0.0001	0.0026	53.13
250888_at	At5g04460	Expressed Protein	0.0005	0.0057	6.87
250852_at	At5g04670	Expressed Protein	0.0289	0.0373	7.25
250823_at	At5g05180	Expressed Protein	0.0085	0.0202	5.96
250742_at	At5g05800	Expressed Protein	0.0277	0.0366	2.52
250602_s_at	At5g07940	Expressed Protein	0.0048	0.0155	3.04
250573_at	At5g08220	Expressed Protein	0.0017	0.0098	6.24
250525_at	At5g08550	Expressed Protein	0.0415	0.0452	3.24
250461_at	At5g10010	Expressed Protein	0.0016	0.0093	2.75
250472_at	At5g10210	Expressed Protein	0.0159	0.0277	4.47
250360_at	At5g11360	Expressed Protein	0.0000	0.0005	3.53
245851_at	At5g13540	Expressed Protein	0.0027	0.0120	3.43
250232_at	At5g13950	Expressed Protein	0.0000	0.0008	2.84
246551_at	At5g15070	Expressed Protein	0.0172	0.0286	4.40
246513_at	At5g15680	Expressed Protein	0.0098	0.0217	3.05
246501_at	At5g16280	Expressed Protein	0.0310	0.0387	6.90
246416_at	At5g16920	Expressed Protein	0.0006	0.0061	9.18
249923_at	At5g19120	Expressed Protein	0.0009	0.0073	2.65
245972_at	At5g20680	Expressed Protein	0.0010	0.0077	2.61
249901_at	At5g22650	Expressed Protein	0.0029	0.0123	2.72
249753_at	At5g24610	Expressed Protein	0.0194	0.0304	2.99
249749_at	At5g24630	Expressed Protein	0.0024	0.0114	2.51
246660_at	At5g35180	Expressed Protein	0.0116	0.0236	3.45
249521_at	At5g38690	Expressed Protein	0.0180	0.0293	4.41
249528_at	At5g38720	Expressed Protein	0.0340	0.0406	4.16
249429_at	At5g39880	Expressed Protein	0.0307	0.0385	4.11
249378_at	At5g40450	Expressed Protein	0.0041	0.0146	6.30
249329_at	At5g40960	Expressed Protein	0.0000	0.0012	4.68
249339_at	At5g41100	Expressed Protein	0.0081	0.0198	2.77
249340_at	At5g41140	Expressed Protein	0.0004	0.0048	27.58
249315_at	At5g41190	Expressed Protein	0.0061	0.0173	3.75
249274_at	At5g41860	Expressed Protein	0.0071	0.0185	9.38
249237_at	At5g42050	Expressed Protein	0.0114	0.0234	4.25
249199_at	At5g42520	Expressed Protein	0.0045	0.0151	2.60
249181_at	At5g42920	Expressed Protein	0.0005	0.0058	3.50
248941_s_at	At5g45460	Expressed Protein	0.0427	0.0460	7.03
248942_at	At5g45480	Expressed Protein	0.0067	0.0180	3.31
248761_at	At5g47635	Expressed Protein	0.0370	0.0424	3.74
248757_at	At5g47680	Expressed Protein	0.0081	0.0198	4.72
248668_at	At5g48720	Expressed Protein	0.0470	0.0484	2.96
248585_at	At5g49640	Expressed Protein	0.0090	0.0207	7.94
248505_at	At5g50360	Expressed Protein	0.0150	0.0270	3.19
248526_at	At5g50730	Expressed Protein	0.0090	0.0208	4.49
248291_at	At5g53020	Expressed Protein	0.0039	0.0143	2.86
248266_at	At5g53440	Expressed Protein	0.0261	0.0355	5.25
248228_at	At5g53800	Expressed Protein	0.0225	0.0329	3.77
248032_at	At5g55860	Expressed Protein	0.0019	0.0101	3.50
247879_at	At5g57770	Expressed Protein	0.0327	0.0398	4.98
247846_at	At5g58100	Expressed Protein	0.0376	0.0429	7.07
247828_at	At5g58510	Expressed Protein	0.0034	0.0134	2.92
247741_at	At5g58960	Expressed Protein	0.0000	0.0017	4.68
247369_at	At5g63340	Expressed Protein	0.0000	0.0012	5.56
247077_at	At5g66420	Expressed Protein	0.0118	0.0238	3.26
247046_at	At5g66540	Expressed Protein	0.0398	0.0442	3.44
258844_at	At3g04740	Expressed Protein	0.0000	0.0006	3.45
266435_s_at	At2g07130	Hypothetical Protein	0.0006	0.0058	5.63
261432_at	At1g07680	Hypothetical Protein	0.0003	0.0042	18.96
260118_s_at	At1g33940	Hypothetical Protein	0.0352	0.0414	7.93
262560_at	At1g34280	Hypothetical Protein	0.0075	0.0190	2.73
262013_s_at	At1g35640	Hypothetical Protein	0.0331	0.0400	7.10
256166_at	At1g36920	Hypothetical Protein	0.0077	0.0193	5.19
257514_at	At1g43940	Hypothetical Protein	0.0030	0.0126	5.38

261856_at	At1g50530	Hypothetical Protein	0.0099	0.0218	7.18
262995_s_at	At1g54430	Hypothetical Protein	0.0353	0.0414	5.35
259917_at	At1g72580	Hypothetical Protein	0.0078	0.0193	6.38
261115_at	At1g75360	Hypothetical Protein	0.0048	0.0156	4.47
264129_at	At1g79170	Hypothetical Protein	0.0038	0.0141	3.92
265566_at	At2g05600	Hypothetical Protein	0.0443	0.0470	2.58
265554_at	At2g07505	Hypothetical Protein	0.0283	0.0370	9.72
263500_s_at	At2g07672	Hypothetical Protein	0.0361	0.0419	8.43
265239_s_at	At2g07692	Hypothetical Protein	0.0423	0.0456	4.51
265601_at	At2g14390	Hypothetical Protein	0.0451	0.0475	3.82
263561_at	At2g15360	Hypothetical Protein	0.0016	0.0094	3.17
265508_at	At2g15930	Hypothetical Protein	0.0031	0.0128	10.60
265808_at	At2g17960	Hypothetical Protein	0.0017	0.0095	4.07
266837_x_at	At2g25990	Hypothetical Protein	0.0017	0.0097	5.89
267022_at	At2g34230	Hypothetical Protein	0.0002	0.0039	6.55
256951_at	At3g19085	Hypothetical Protein	0.0040	0.0145	8.67
256565_at	At3g19516	Hypothetical Protein	0.0279	0.0368	10.20
257577_at	At3g30150	Hypothetical Protein	0.0049	0.0157	3.27
256606_at	At3g32960	Hypothetical Protein	0.0020	0.0104	4.74
252807_at	At3g42400	Hypothetical Protein	0.0267	0.0359	6.03
252752_s_at	At3g43480	Hypothetical Protein	0.0024	0.0113	8.80
252719_at	At3g43950	Hypothetical Protein	0.0188	0.0300	4.67
257448_s_at	At3g45800	Hypothetical Protein	0.0039	0.0144	6.01
252249_at	At3g49770	Hypothetical Protein	0.0311	0.0388	5.37
251419_at	At3g60470	Hypothetical Protein	0.0023	0.0111	5.70
255470_at	At4g03040	Hypothetical Protein	0.0083	0.0200	4.12
255192_at	At4g07380	Hypothetical Protein	0.0184	0.0297	3.89
255106_at	At4g08710	Hypothetical Protein	0.0020	0.0104	4.75
254514_at	At4g20250	Hypothetical Protein	0.0008	0.0069	6.92
254220_at	At4g23780	Hypothetical Protein	0.0000	0.0016	9.84
246248_at	At4g36560	Hypothetical Protein	0.0433	0.0464	5.27
251079_at	At5g02000	Hypothetical Protein	0.0241	0.0340	4.09
245978_at	At5g13130	Hypothetical Protein	0.0012	0.0082	9.77
246543_at	At5g15060	Hypothetical Protein	0.0430	0.0462	5.85
249819_at	At5g23640	Hypothetical Protein	0.0227	0.0331	6.65
249660_at	At5g36720	Hypothetical Protein	0.0092	0.0209	7.59
248650_at	At5g49250	Hypothetical Protein	0.0072	0.0187	3.55
248561_at	At5g49670	Hypothetical Protein	0.0057	0.0168	12.05
247961_at	At5g56570	Hypothetical Protein	0.0090	0.0207	5.10
247021_at	At5g67040	Hypothetical Protein	0.0156	0.0274	3.15
265457_at	At2g46550	Unknown protein	0.0253	0.0350	2.89
259275_at	At3g01060	Unknown protein	0.0097	0.0215	2.76
258949_at	At3g01370	Unknown protein	0.0043	0.0148	4.27
259181_at	At3g01690	Unknown protein	0.0283	0.0370	3.25
259240_at	At3g11590	Unknown protein	0.0053	0.0163	3.58
251753_at	At3g55760	Unknown protein	0.0019	0.0101	2.84
251684_at	At3g56410	Unknown protein	0.0134	0.0255	2.95
251148_at	At3g63180	Unknown protein	0.0393	0.0439	2.66
245517_at	At4g15840	Unknown protein	0.0219	0.0323	3.14
252976_s_at	At4g38550	Unknown protein	0.0383	0.0433	2.71
250784_at	At5g05480	Unknown protein	0.0037	0.0140	2.56
246558_at	At5g15540	Unknown protein	0.0467	0.0483	3.26
249936_at	At5g22450	Unknown protein	0.0073	0.0188	2.67
247977_at	At5g56850	Unknown protein	0.0098	0.0216	2.96
247773_at	At5g58630	Unknown protein	0.0011	0.0079	5.07
247649_at	At5g60030	Unknown protein	0.0001	0.0026	4.24
266680_s_at	At2g19850	Unknown protein	0.0045	0.0152	5.18
245990_at	At5g20640	Unknown protein	0.0398	0.0442	2.75
248711_at	At5g48270	Unknown protein	0.0033	0.0133	13.14
256542_at	At1g42550	Unknown protein	0.0005	0.0054	4.00
264147_at	At1g02200	Unknown Protein	0.0001	0.0028	13.23
264796_at	At1g08690	Unknown Protein	0.0290	0.0374	2.69
262651_at	At1g14100	Unknown Protein	0.0260	0.0354	6.01
261992_at	At1g33690	Unknown Protein	0.0016	0.0093	22.33
246357_x_at	At1g40550	Unknown Protein	0.0487	0.0494	3.89
262064_at	At1g56075	Unknown Protein	0.0008	0.0071	3.07
260448_at	At1g72400	Unknown Protein	0.0026	0.0118	4.19
263059_at	At2g07670	Unknown Protein	0.0005	0.0055	5.72
265980_at	At2g11160	Unknown Protein	0.0040	0.0145	8.08
245081_at	At2g23280	Unknown Protein	0.0381	0.0432	2.61
266833_at	At2g30030	Unknown Protein	0.0030	0.0127	13.50
265797_at	At2g35715	Unknown Protein	0.0322	0.0394	7.68
267067_at	At2g41030	Unknown Protein	0.0000	0.0014	6.76
266437_at	At2g43170	Unknown Protein	0.0090	0.0207	4.08

266871_at	At2g44720	Unknown Protein	0.0500	0.0500	5.49
259047_at	At3g03390	Unknown Protein	0.0093	0.0210	3.18
258187_at	At3g17870	Unknown Protein	0.0101	0.0220	2.64
258233_at	At3g27780	Unknown Protein	0.0239	0.0339	3.28
252171_at	At3g50600	Unknown Protein	0.0013	0.0085	4.96
255211_at	At4g07610	Unknown Protein	0.0039	0.0142	4.21
246084_at	At5g20530	Unknown Protein	0.0201	0.0311	3.93
246845_at	At5g26707	Unknown Protein	0.0042	0.0147	2.76
246694_at	At5g29080	Unknown Protein	0.0039	0.0143	4.65
246698_at	At5g30332	Unknown Protein	0.0054	0.0163	5.08
246675_at	At5g31411	Unknown Protein	0.0062	0.0174	3.13
248036_at	At5g55915	Unknown Protein	0.0121	0.0242	4.70

[a]Probe set ID represents Affymeterix probe set number.

[b]AGI represents *Arabidopsis* Genome Initiative (AGI) locus identifier corresponding to each gene represented on the array.

[c] q -value ≤ 0.05 (5% False discovery rate) was used to determine genes differentially expressed in PPV-infected leaves relative to mock-inoculated control leaves.

[d] p -values ($p \leq 0.05$) were derived from the ANOVA and used to calculate q -value after adjusing the values using Benjamini and Hochberg [21] multiple testing correction.

[e]Calculation of fold changes was defined in Methods.

[f] Determined followingt the method of the *Arabidopsi* s MIPS (Munich Information Centre for Protein Sequences) fucntional classification scheme.
