# Supplementary material for: Altered gene expression changes in Arabidopsis leaf tissues and protoplasts in response to Plum pox virus infection
Source: BMC Genomics. 2008 Jul 9;9:325. doi: 10.1186/1471-2164-9-325 (PMC2478689; doi:10.1186/1471-2164-9-325)
Supplement: Additional file 2 — Supplemental Table 2. Expression levels of genes repressed in PPV-infected Arabidopsis leaf tissues 17 days post inoculation. [file 1471-2164-9-325-S2.pdf]

Supplemental Table 2. Expression levels of genes repressed in PPV-infected *Arabidopsis* leaf tissues 17 days post inoculation

Probe set IDs[a]	AGI[b] locus	Description	*q* -value[c]	*p* -value[d]	Fold change[e]
A. Defence and virulence[f]					
249514_at	At5g38480	14-3-3 protein; GRF3	0.0241	0.0341	-3.1
266620_at	At2g35450	Amidohydrolase family	0.0463	0.0480	-3.5
266851_at	At2g26820	Avirulence induced gene; AIG1 family protein	0.0019	0.0102	-4.8
258002_at	At3g28930	AvrRpt2-induced AIG2 protein; AIG2	0.0460	0.0479	-2.8
253674_at	At4g29540	Bacterial transferase hexapeptide repeat-containing protein	0.0100	0.0218	-3.9
252170_at	At3g50480	Broad-spectrum mildew resistance RPW8 family protein	0.0237	0.0338	-3.2
260560_at	At2g43590	Chitinase, putative	0.0156	0.0274	-5.7
251895_at	At3g54420	Class IV chitinase; CHIV	0.0044	0.0150	-4.5
258897_at	At3g05730	Defensin like family protein	0.0000	0.0014	-6.8
258798_at	At3g04540	Defensin like family protein	0.0383	0.0433	-7.6
255319_at	At4g04220	Disease resistance family protein	0.0225	0.0329	-10.4
267546_at	At2g32680	Disease resistance family protein	0.0017	0.0096	-2.6
267548_at	At2g32660	Disease resistance family protein / LRR family protein	0.0023	0.0111	-13.4
259893_at	At1g71390	Disease resistance family protein / LRR family protein	0.0018	0.0099	-7.2
259534_at	At1g12290	Disease resistance protein; CC-NBS-LRR class, putative	0.0029	0.0123	-6.7
248836_at	At5g47260	Disease resistance protein; CC-NBS-LRR class, putative	0.0329	0.0399	-3.8
257276_at	At3g14460	Disease resistance protein; NBS-LRR class, putative	0.0022	0.0107	-7.7
264153_at	At1g65390	Disease resistance protein;TIR class, putative	0.0290	0.0374	-3.7
260713_at	At1g17615	Disease resistance protein;TIR-NBS class, putative	0.0048	0.0156	-16.5
248993_at	At5g45240	Disease resistance protein;TIR-NBS-LRR class, putative	0.0005	0.0057	-8.4
249311_at	At5g41540	Disease resistance protein;TIR-NBS-LRR class, putative	0.0192	0.0303	-3.5
254582_at	At4g19470	Disease resistance protein-related	0.0066	0.0180	-7.9
255937_at	At1g12610	DRE-binding protein, putative	0.0187	0.0300	-5.5
254074_at	At4g25490	DRE-binding protein; DREB1B	0.0123	0.0245	-3.9
255504_at	At4g02200	Drought-responsive family protein	0.0122	0.0243	-3.9
246824_at	At5g26990	Drought-responsive family protein	0.0056	0.0167	-3.5
257641_s_at	At3g25760	Early-responsive to dehydration stress protein; ERD12	0.0128	0.0249	-2.6
245505_at	At4g15690	Glutaredoxin family protein	0.0220	0.0324	-24.2
245506_at	At4g15700	Glutaredoxin family protein	0.0063	0.0175	-12.3
263168_at	At1g03020	Glutaredoxin family protein	0.0318	0.0392	-5.0
265067_at	At1g03850	Glutaredoxin family protein	0.0046	0.0153	-4.8
260831_at	At1g06830	Glutaredoxin family protein	0.0100	0.0219	-3.8
256583_at	At3g28850	Glutaredoxin family protein	0.0289	0.0373	-2.9
249996_at	At5g18600	Glutaredoxin family protein	0.0004	0.0048	-2.8
249380_at	At5g40370	Glutaredoxin family protein	0.0071	0.0185	-4.1
246092_at	At5g20500	Glutaredoxin family protein	0.0027	0.0119	-3.4
260225_at	At1g74590	Glutathione S-transferase, putative	0.0405	0.0446	-6.0
262120_at	At1g02950	Glutathione S-transferase, putative	0.0171	0.0286	-4.5
266267_at	At2g29460	Glutathione S-transferase, putative	0.0109	0.0229	-3.6
260406_at	At1g69920	Glutathione S-transferase, putative	0.0027	0.0119	-3.5
262119_s_at	At1g02930	Glutathione S-transferase, putative	0.0272	0.0362	-3.5
261311_at	At1g05770	Jacalin lectin family protein	0.0005	0.0054	-2.6
257206_at	At3g16530	Legume lectin family protein	0.0002	0.0037	-5.6
251065_at	At5g01870	Lipid transfer protein, putative	0.0229	0.0332	-5.2
260904_at	At1g02450	NPR1/NIM1-interacting protein 1; NIMIN-1	0.0499	0.0499	-4.9
261572_at	At1g01170	Ozone-responsive stress-related protein, putative	0.0022	0.0106	-4.0
265586_at	At2g19990	Pathogenesis-related protein 1;PR-1	0.0288	0.0373	-10.6
259925_at	At1g75040	Pathogenesis-related protein 5;PR-5	0.0003	0.0041	-7.7
258722_at	At3g09590	Pathogenesis-related protein, putative	0.0061	0.0173	-18.1
257077_at	At3g19690	Pathogenesis-related protein, putative	0.0187	0.0300	-9.8
249439_at	At5g40020	Pathogenesis-related thaumatin family protein	0.0009	0.0071	-8.8
260655_at	At1g19320	Pathogenesis-related thaumatin family protein	0.0059	0.0170	-4.3
257365_x_at	At2g26020	Plantdefensin-fusion protein, putative; PDF1.2b	0.0010	0.0076	-45.7
266141_at	At2g02120	Plantdefensin-fusion protein, putative; PDF2.1	0.0028	0.0123	-25.8
255554_at	At4g01895	SAR regulator protein NIMIN-1-related	0.0054	0.0164	-4.4
252135_at	At3g50830	Stress-responsive protein, putative	0.0223	0.0328	-3.6
256527_at	At1g66100	Thionin, putative	0.0183	0.0297	-4.8
249645_at	At5g36910	Thionin;THI2.2	0.0003	0.0043	-5.4
266048_at	At2g40790	Thioredoxin family protein	0.0015	0.0091	-13.7
261821_at	At1g11530	Thioredoxin family protein	0.0257	0.0353	-5.4
253693_at	At4g29670	Thioredoxin family protein	0.0035	0.0135	-4.4
258665_at	At3g08710	Thioredoxin family protein	0.0147	0.0266	-4.0
250649_at	At5g06690	Thioredoxin family protein	0.0037	0.0139	-3.3
262721_at	At1g43560	Thioredoxin family protein	0.0004	0.0053	-3.3

257495_at	At1g07960	Thioredoxin family protein	0.0128	0.0250	-2.7
249385_at	At5g39950	Thioredoxin H-type 2; TRX-H-2	0.0076	0.0191	-2.9
260943_at	At1g45145	Thioredoxin H-type 5; TRX-H-5	0.0017	0.0096	-11.5
250133_at	At5g16400	Thioredoxin, putative	0.0038	0.0140	-3.1
257910_at	At3g25580	Thioredoxin-related	0.0372	0.0426	-2.5
260551_at	At2g43510	Trypsin inhibitor, putative	0.0122	0.0244	-6.7
260541_at	At2g43530	Trypsin inhibitor, putative	0.0035	0.0135	-3.9
254775_at	At4g13450	Universal stress protein; USP family protein	0.0068	0.0181	-6.9
266458_at	At2g47710	Universal stress protein; USP family protein	0.0067	0.0180	-2.7
257892_at	At3g17020	Universal stress protein; USP family protein	0.0009	0.0072	-2.6
255807_at	At4g10270	Wound-responsive family protein	0.0309	0.0387	-6.9
248159_at	At5g54460	Wound-responsive protein-related	0.0271	0.0361	-10.2
258821_at	At3g07230	Wound-responsive protein-related	0.0004	0.0049	-7.5
A1. Protein folding/heat shock/chaperone activity					
260714_at	At1g14980	Chaperonin; CPN10	0.0070	0.0183	-2.8
259824_at	At1g66240	Copper chaperone, putative; CCH	0.0001	0.0026	-3.1
251733_at	At3g56240	Copper chaperone; CCH	0.0015	0.0091	-4.1
267462_at	At2g33735	DNAJ heat shock N-terminal domain-containing protein	0.0216	0.0321	-6.6
258716_at	At3g09700	DNAJ heat shock N-terminal domain-containing protein	0.0325	0.0397	-3.5
256999_at	At3g14200	DNAJ heat shock N-terminal domain-containing protein	0.0188	0.0300	-3.3
248637_at	At5g49060	DNAJ heat shock N-terminal domain-containing protein	0.0421	0.0456	-2.9
265826_at	At2g35795	DNAJ heat shock N-terminal domain-containing protein	0.0111	0.0231	-2.7
245800_at	At1g46264	Heat shock family protein	0.0062	0.0174	-5.4
266841_at	At2g26150	Heat shock family protein	0.0123	0.0245	-5.0
247691_at	At5g59720	Heat shock protein; HSP18	0.0101	0.0220	-8.3
254384_at	At4g21870	Heat shock protein; HSP26	0.0375	0.0428	-12.7
249575_at	At5g37670	Small heat shock protein-like; HSP15	0.0189	0.0301	-3.2
A2. Cell wall associated transcripts					
A2.1. Reassmebly of cell wall associated genes					
258895_at	At3g05600	Epoxide hydrolase, putative	0.0224	0.0328	-12.3
261245_at	At1g20130	Extracellular lipase, putative	0.0166	0.0282	-5.1
257441_at	At2g04020	GDSL-motif lipase/hydrolase family protein	0.0037	0.0139	-14.9
263789_at	At2g24560	GDSL-motif lipase/hydrolase family protein	0.0061	0.0173	-14.5
250519_at	At5g08460	GDSL-motif lipase/hydrolase family protein	0.0065	0.0178	-11.8
249277_at	At5g41890	GDSL-motif lipase/hydrolase family protein	0.0049	0.0157	-9.8
250043_at	At5g18430	GDSL-motif lipase/hydrolase family protein	0.0171	0.0286	-9.0
246526_at	At5g15720	GDSL-motif lipase/hydrolase family protein	0.0322	0.0394	-6.3
250918_at	At5g03610	GDSL-motif lipase/hydrolase family protein	0.0250	0.0348	-2.7
259935_at	At1g71250	GDSL-motif lipase/hydrolase family protein	0.0117	0.0237	-2.5
250082_at	At5g17200	Glycoside hydrolase family 28 protein	0.0022	0.0107	-18.1
254733_at	At4g13760	Glycoside hydrolase family 28 protein	0.0097	0.0216	-5.2
266558_at	At2g23900	Glycoside hydrolase family 28 protein	0.0088	0.0205	-4.5
254339_at	At4g22100	Glycosyl hydrolase family 1 protein	0.0300	0.0381	-13.3
248168_at	At5g54570	Glycosyl hydrolase family 1 protein	0.0461	0.0479	-8.8
255150_at	At4g08160	Glycosyl hydrolase family 10 protein	0.0069	0.0181	-4.8
267620_at	At2g39640	Glycosyl hydrolase family 17 protein	0.0295	0.0378	-4.7
258805_at	At3g04010	Glycosyl hydrolase family 17 protein	0.0056	0.0168	-3.7
251625_at	At3g57260	Glycosyl hydrolase family 17 protein	0.0016	0.0094	-3.7
253226_at	At4g35010	Glycosyl hydrolase family 35 protein	0.0328	0.0398	-4.2
253809_at	At4g28320	Glycosyl hydrolase family 5 protein	0.0006	0.0063	-6.4
252920_at	At4g39000	Glycosyl hydrolase family 9 protein	0.0003	0.0044	-3.6
254202_at	At4g24140	Hydrolase; alpha/beta fold family protein	0.0424	0.0457	-6.1
265341_at	At2g18360	Hydrolase; alpha/beta fold family protein	0.0240	0.0340	-4.7
261032_at	At1g17430	Hydrolase; alpha/beta fold family protein	0.0048	0.0155	-3.9
267126_s_at	At2g23590	Hydrolase; alpha/beta fold family protein	0.0158	0.0276	-2.6
260051_at	At1g78210	Hydrolase; alpha/beta fold family protein	0.0285	0.0371	-2.6
262475_at	At1g50340	Invertase/pectin methylesterase inhibitor family protein	0.0000	0.0006	-17.3
247246_at	At5g64620	Invertase/pectin methylesterase inhibitor family protein	0.0143	0.0263	-4.2
259616_at	At1g47960	Invertase/pectin methylesterase inhibitor family protein	0.0039	0.0144	-3.0
250299_at	At5g11910	Lipase family protein	0.0093	0.0211	-2.8
266475_at	At2g31100	Lipase, putative	0.0285	0.0371	-3.8
254689_at	At4g13710	Pectate lyase family protein	0.0003	0.0040	-4.6
260962_at	At1g44980	Pectinesterase family protein	0.0319	0.0392	-5.5
A2.2. Matrix polymers					
259997_at	At1g67880	Glycosyl transferase family 17 protein	0.0395	0.0440	-2.5

261877_at	At1g50580	Glycosyltransferase family protein	0.0022	0.0108	-3.4
263861_at	At2g04560	Glycotransferase family protein 19	0.0273	0.0363	-10.1
248725_at	At5g47980	Transferase family protein	0.0136	0.0257	-10.1
253721_at	At4g29250	Transferase family protein	0.0306	0.0385	-4.2
245068_at	At2g23260	UDP-glucoronosyl/UDP-glucosyl transferase family protein	0.0476	0.0487	-4.6
256053_at	At1g07260	UDP-glucoronosyl/UDP-glucosyl transferase family protein	0.0429	0.0461	-2.8
262652_at	At1g14110	Xyloglucan fucosyltransferase family protein	0.0059	0.0170	-8.7
263565_at	At2g15390	Xyloglucan fucosyltransferase, putative; FUT4	0.0089	0.0207	-2.8

A2.3.Structural proteins

257986_at	At3g20865	Arabinogalactan-protein, putative;AGP	0.0445	0.0471	-9.9
263453_at	At2g22180	Hydroxyproline-rich glycoprotein family protein	0.0260	0.0354	-5.3
258558_at	At3g06000	Leucine-rich repeat family protein	0.0078	0.0193	-9.5
257139_at	At3g28890	Leucine-rich repeat family protein	0.0327	0.0398	-8.2
261989_at	At1g33670	Leucine-rich repeat family protein	0.0260	0.0354	-8.0
250577_at	At5g07910	Leucine-rich repeat family protein	0.0040	0.0145	-4.3
265536_at	At2g15880	Leucine-rich repeat family protein / extensin family protein	0.0382	0.0433	-5.6
246366_at	At1g51850	Leucine-rich repeat protein, putative	0.0465	0.0481	-3.6
254467_at	At4g20450	Leucine-rich repeat protein, putative	0.0138	0.0258	-3.4
252518_at	At3g46350	Leucine-rich repeat protein, putative	0.0001	0.0026	-3.1
252517_at	At3g46340	Leucine-rich repeat protein, putative	0.0126	0.0247	-3.0
260750_at	At1g49100	Leucine-rich repeat protein, putative	0.0059	0.0171	-2.9
245992_at	At5g20690	Leucine-rich repeat protein, putative	0.0000	0.0006	-8.8
249013_at	At5g44700	Leucine-rich repeat protein, putative	0.0361	0.0419	-8.0
261527_at	At1g14390	Leucine-rich repeat protein, putative	0.0419	0.0454	-4.8
252197_at	At3g50230	Leucine-rich repeat protein, putative	0.0162	0.0280	-3.0
252940_at	At4g39270	Leucine-rich repeat protein, putative	0.0135	0.0256	-2.8
250348_at	At5g11990	Proline-rich family protein	0.0040	0.0145	-7.0
256352_at	At1g54970	Proline-rich family protein	0.0107	0.0226	-5.3
259720_at	At1g61080	Proline-rich family protein	0.0040	0.0145	-4.1
258543_at	At3g06870	Proline-rich family protein	0.0255	0.0351	-3.8
247560_at	At5g61090	Proline-rich family protein	0.0266	0.0358	-2.9
248636_at	At5g49080	Proline-rich -like family protein	0.0104	0.0223	-4.2

A2.4. Expansins

266215_at	At2g06850	Xyloglucan endotransglycosylase	0.0170	0.0285	-3.0
247925_at	At5g57560	Xyloglucan endotransglycosylase	0.0224	0.0328	-3.8
254598_at	At4g18990	Xyloglucan endotransglycosylase	0.0090	0.0208	-13.9
248732_at	At5g48070	Xyloglucan endotransglycosylase	0.0000	0.0017	-5.9
266376_at	At2g14620	Xyloglucan endotransglycosylase	0.0362	0.0420	-5.8
257203_at	At3g23730	Xyloglucan endotransglycosylase	0.0178	0.0291	-3.9
254801_at	At4g13080	Xyloglucan endotransglycosylase	0.0120	0.0240	-3.3
255433_at	At4g03210	Xyloglucan endotransglycosylase	0.0369	0.0424	-3.1
247162_at	At5g65730	Xyloglucan endotransglycosylase	0.0035	0.0135	-2.6

B. Cellular communication/Signal transduction mechanism/transmembrane signal transduction

254605_at	At4g18950	Ankyrin protein kinase, putative	0.0031	0.0128	-3.7
267118_at	At2g32590	Barren family protein	0.0206	0.0314	-4.3
248001_at	At5g55990	Calcineurin B-like protein 2;CBL2	0.0465	0.0481	-3.1
245300_at	At4g16350	Calcineurin B-like protein 6;CBL6	0.0101	0.0220	-12.6
248827_at	At5g47100	Calcineurin B-like protein 9;CBL9	0.0060	0.0172	-2.5
249783_at	At5g24270	Calcineurin B-like protein, putative	0.0277	0.0366	-4.4
259960_at	At1g53710	Calcineurin-like phosphoesterase family protein	0.0019	0.0101	-11.8
253491_at	At4g31770	Calcineurin-like phosphoesterase family protein	0.0376	0.0429	-3.5
253128_at	At4g36070	Calcium-dependent protein kinase family protein	0.0074	0.0188	-2.7
262639_at	At1g62820	Calmodulin, putative	0.0241	0.0341	-6.6
256755_at	At3g25600	Calmodulin, putative	0.0105	0.0224	-3.5
267064_at	At2g41110	Calmodulin-2/3/5;CAM2;Cal1	0.0063	0.0175	-4.6
246290_at	At3g56800	Calmodulin-2/3/5;CAM3	0.0382	0.0433	-2.9
266317_at	At2g27030	Calmodulin-2/3/5;CAM5;TCH1	0.0343	0.0408	-2.8
252037_at	At3g51920	Calmodulin-9; CAM9	0.0007	0.0064	-3.2
267076_at	At2g41090	Calmodulin-like calcium-binding protein; CaBP-22	0.0132	0.0253	-2.5
266447_at	At2g43290	Calmodulin-like protein; MSS3	0.0470	0.0484	-3.5
249583_at	At5g37770	Calmodulin-related protein 2	0.0003	0.0042	-4.2
258947_at	At3g01830	Calmodulin-related protein, putative	0.0050	0.0158	-6.6
249197_at	At5g42380	Calmodulin-related protein, putative	0.0198	0.0308	-5.9
265494_at	At2g15680	Calmodulin-related protein, putative	0.0142	0.0262	-2.5
267257_at	At2g23080	Casein kinase II alpha chain, putative	0.0258	0.0353	-2.5

255872_at	At2g30360	CBL-interacting protein kinase 11;CIPK11	0.0058	0.0169	-3.2
248450_at	At5g51290	Ceramide kinase-related	0.0027	0.0120	-2.7
261506_at	At1g71697	Choline kinase, putative	0.0352	0.0414	-3.0
248675_at	At5g48820	Cyclin-dependent kinase inhibitor 3; ICK3	0.0097	0.0215	-4.1
264244_at	At1g60440	Eukaryotic pantothenate kinase family protein	0.0166	0.0282	-2.6
259071_at	At3g11650	harpin-induced family protein / HIN1 family protein / harpin-responsive family [illegible]	0.0143	0.0262	-3.1
255577_at	At4g01410	Harpin-induced family protein; HIN1	0.0002	0.0037	-2.7
255404_at	At4g03153	Kinase interacting family protein	0.0004	0.0050	-8.0
259836_at	At1g52240	Kinase partner protein	0.0320	0.0393	-8.8
262932_at	At1g65820	Microsomal glutathione s-transferase, putative	0.0453	0.0476	-4.1
254924_at	At4g11330	Mitogen-activated protein kinase, putative; MPK5	0.0249	0.0347	-5.3
254958_at	At4g11010	Nucleoside diphosphate kinase 3; NDK3	0.0049	0.0157	-3.0
262398_at	At1g49350	pfkB-type carbohydrate kinase family protein	0.0000	0.0002	-2.6
254897_at	At4g11470	Protein kinase family Protein	0.0060	0.0172	-12.3
253227_at	At4g35030	Protein kinase family Protein	0.0182	0.0296	-9.2
261611_at	At1g49730	Protein kinase family Protein	0.0014	0.0089	-9.1
255653_at	At4g00960	Protein kinase family Protein	0.0061	0.0173	-9.0
266651_at	At2g25760	Protein kinase family Protein	0.0333	0.0401	-5.7
255820_at	At2g40580	Protein kinase family Protein	0.0284	0.0371	-5.0
252494_at	At3g46760	Protein kinase family Protein	0.0307	0.0385	-5.0
263748_at	At2g21480	Protein kinase family Protein	0.0440	0.0468	-3.6
254246_at	At4g23250	Protein kinase family Protein	0.0389	0.0437	-3.2
246146_at	At5g20050	Protein kinase family Protein	0.0160	0.0279	-3.0
254271_at	At4g23150	Protein kinase family Protein	0.0167	0.0283	-2.9
251603_at	At3g57760	Protein kinase family Protein	0.0299	0.0381	-2.8
248119_at	At5g54590	Protein kinase family Protein	0.0070	0.0183	-2.8
254869_at	At4g11890	Protein kinase family Protein	0.0021	0.0105	-2.7
250365_at	At5g11410	Protein kinase family Protein	0.0043	0.0148	-2.5
267582_at	At2g41970	Protein kinase, putative	0.0115	0.0235	-14.2
251648_at	At3g57720	Protein kinase, putative	0.0058	0.0170	-4.1
248821_at	At5g47070	Protein kinase, putative	0.0168	0.0283	-3.1
261089_at	At1g07570	Protein kinase; APK1a	0.0228	0.0332	-3.0
252212_at	At3g50310	Protein kinase-related	0.0098	0.0217	-11.2
256213_at	At1g50990	Protein kinase-related	0.0362	0.0420	-10.0
256044_at	At1g07160	Protein phosphatase 2C, putative; PP2C, putative	0.0383	0.0433	-3.7
254660_at	At4g18250	Receptor serine/threonine kinase, putative	0.0024	0.0113	-18.5
254265_s_at	At4g23140	Receptor-like protein kinase 5; RLK5	0.0059	0.0170	-4.9
254252_at	At4g23310	Receptor-like protein kinase, putative	0.0291	0.0374	-7.6
252549_at	At3g45860	Receptor-like protein kinase, putative	0.0071	0.0185	-3.3
247600_at	At5g60890	Receptor-like protein kinase; ATR1	0.0067	0.0181	-2.5
266655_at	At2g25880	Serine/threonine protein kinase, putative	0.0054	0.0164	-17.8
262240_at	At1g48220	Serine/threonine protein kinase, putative	0.0463	0.0480	-6.6
258281_at	At3g26900	Shikimate kinase family protein	0.0002	0.0036	-3.7
255419_at	At4g03230	S-locus lectin protein kinase family protein	0.0461	0.0479	-7.7
262480_at	At1g11340	S-locus lectin protein kinase family protein	0.0008	0.0070	-7.3
262507_at	At1g11330	S-locus lectin protein kinase family protein	0.0112	0.0233	-3.2
264762_at	At1g61460	S-locus protein kinase, putative	0.0372	0.0425	-4.5
256744_at	At3g29350	Two-component phosphorelay mediator 1; HP1	0.0252	0.0349	-2.8
249473_at	At5g39340	Two-component phosphorelay mediator 2; HP2	0.0164	0.0281	-5.5
266078_at	At2g40670	Two-component responsive regulator; ARR16	0.0086	0.0203	-3.2
265116_at	At1g62480	Vacuolar calcium-binding protein-related	0.0043	0.0149	-4.3
260657_at	At1g19390	Wall-associated kinase, putative	0.0399	0.0442	-3.1
C. Cell Cycle/ DNA processing/chromatin regulation and cytoskeleton reorganization					
264089_at	At2g31200	Actin-depolymerizing factor 6; ADF6	0.0192	0.0303	-4.1
253217_at	At4g34970	Actin-depolymerizing factor, putative	0.0015	0.0090	-10.9
252565_at	At3g46000	Actin-depolymerizing factor, putative; ADF2	0.0139	0.0259	-4.5
250454_at	At5g09830	BolA-like family protein	0.0015	0.0091	-4.6
246441_at	At5g17560	BolA-like family protein	0.0045	0.0151	-4.4
261631_at	At1g49940	β-Tubulin protein	0.0435	0.0465	-3.0
253142_at	At4g35520	DNA mismatch repair family protein	0.0178	0.0292	-6.9
260909_at	At1g02670	DNA repair protein, putative	0.0102	0.0222	-18.7
245525_at	At4g15930	Dynein light chain, putative	0.0045	0.0151	-4.2
245750_at	At1g51060	Histone H2A, putative	0.0217	0.0322	-3.1
264188_at	At1g54690	Histone H2A, putative	0.0046	0.0152	-2.5
251846_at	At3g54560	Histone H2A.F/Z	0.0008	0.0069	-2.6
252561_at	At3g45980	Histone H2B	0.0045	0.0151	-3.7
247180_at	At5g65350	Histone H3	0.0022	0.0107	-24.7

251924_at	At3g53730	Histone H4	0.0042	0.0147	-3.0
257115_at	At3g20150	Kinesin motor family protein	0.0004	0.0053	-31.0
266009_at	At2g37420	Kinesin motor protein-related	0.0053	0.0163	-4.8
245174_at	At2g47500	Kinesin motor protein-related	0.0002	0.0034	-4.3
252076_at	At3g51660	Macrophage migration inhibitory factor family protein; MIF	0.0086	0.0202	-4.1
247943_at	At5g57170	Macrophage migration inhibitory factor family protein; MIF	0.0007	0.0064	-2.7
253793_at	At4g28710	Myosin heavy chain, putative	0.0278	0.0367	-3.0
264763_at	At1g61450	Myosin II	0.0029	0.0123	-2.8
245162_at	At2g33240	Myosin, putative	0.0004	0.0050	-13.1
259089_at	At3g04960	NUF1 PROTEIN	0.0011	0.0080	-12.1
266394_at	At2g43130	Ras-related protein; ARA-4	0.0110	0.0230	-3.2
252472_at	At3g46830	Ras-related protein; RAB11A	0.0091	0.0208	-2.5
264209_at	At1g22740	Ras-related protein; RAB7	0.0179	0.0292	-9.2
248983_at	At5g45130	Ras-related protein;RHA1	0.0147	0.0266	-2.6
252428_at	At3g47660	Regulator of chromosome condensation; RCC1 family protein	0.0098	0.0217	-2.8
267494_at	At2g30410	Tubulin folding cofactor A	0.0016	0.0093	-3.3
D. Development/storage proteins					
253393_at	At4g32690	2-on-2 hemoglobin; GLB3	0.0332	0.0400	-3.8
260238_at	At1g74520	ABA-responsive protein;HVA22a	0.0116	0.0236	-3.1
262799_at	At1g20970	Adhesin-related	0.0305	0.0384	-2.5
260013_at	At1g68090	Annexin 5; ANN5	0.0498	0.0499	-6.1
250473_at	At5g10220	Annexin 6; ANN6	0.0003	0.0043	-17.7
262992_at	At1g54210	Autophagy 12a; APG12a	0.0024	0.0113	-16.2
264285_at	At1g62040	Autophagy 8c; APG8c	0.0456	0.0478	-4.1
265510_at	At2g05630	Autophagy 8d; APG8d	0.0083	0.0200	-3.3
266106_at	At2g45170	Autophagy 8e; APG8e	0.0338	0.0404	-3.7
245391_at	At4g16520	Autophagy 8f; APG8f	0.0031	0.0128	-3.4
251393_at	At3g60640	Autophagy 8g; APG8g	0.0130	0.0252	-5.9
257293_at	At3g15580	Autophagy 8i; APG8i	0.0014	0.0089	-7.8
247906_at	At5g57420	Auxin (indole-3-acetic acid) induced gene (IAA33)	0.0379	0.0431	-5.6
263890_at	At2g37030	Auxin-responsive family protein	0.0033	0.0132	-11.4
254809_at	At4g12410	Auxin-responsive family protein	0.0116	0.0236	-5.0
258653_at	At3g09870	Auxin-responsive family protein	0.0053	0.0163	-4.4
248243_at	At5g53590	Auxin-responsive family protein	0.0003	0.0042	-3.0
266322_at	At2g46690	Auxin-responsive family protein	0.0101	0.0220	-2.6
260152_at	At1g52830	Auxin (indole-3-acetic acid) induced gene (IAA6)	0.0002	0.0039	-2.6
247135_at	At5g66260	Auxin-responsive protein, putative	0.0014	0.0089	-15.6
257121_at	At3g20220	Auxin-responsive protein, putative	0.0002	0.0039	-5.4
260427_at	At1g72430	Auxin-responsive protein-related	0.0015	0.0090	-5.1
251579_at	At3g58170	Bet1-like SNARE 1-1	0.0030	0.0126	-2.5
266134_s_at	At2g45110	Beta-expansin, putative; EXPB4	0.0250	0.0348	-6.8
251336_at	At3g61190	BON1-associated protein 1;BAP1	0.0047	0.0154	-6.0
257363_at	At2g45760	BON1-associated protein;BAP1-related	0.0011	0.0078	-4.4
252409_at	At3g47650	Bundle-sheath defective protein 2 family / bsd2 family	0.0037	0.0139	-4.9
265624_at	At2g27250	CLAVATA3	0.0026	0.0118	-3.7
257513_s_at	At1g12340	Cornichon family protein	0.0315	0.0390	-2.9
262102_at	At1g02980	Cullin family protein	0.0015	0.0091	-3.1
259124_at	At3g02310	Developmental protein SEPALLATA2	0.0133	0.0254	-12.4
256847_at	At3g27950	Early nodule-specific protein, putative	0.0051	0.0160	-9.6
257927_at	At3g23240	Ethylene-responsive factor 1 / ethylene response factor 1; ERF1	0.0005	0.0058	-4.3
260783_at	At1g06160	Ethylene-responsive factor, putative	0.0007	0.0063	-2.8
257918_at	At3g23230	Ethylene-responsive factor, putative	0.0163	0.0280	-2.7
249042_at	At5g44350	Ethylene-responsive factor, putative	0.0046	0.0153	-3.0
263179_at	At1g05710	Ethylene-responsive factor, putative	0.0163	0.0280	-5.3
252563_at	At3g45970	Expansin family protein; EXPL1	0.0256	0.0352	-4.7
266070_at	At2g18660	Expansin family protein; EXPR3	0.0073	0.0188	-8.4
253616_at	At4g30380	Expansin-related	0.0202	0.0311	-3.7
251898_at	At3g54340	Floral homeotic protein APETALA3; AP3	0.0199	0.0309	-17.3
245226_at	At3g29970	Germination protein-related	0.0012	0.0082	-2.8
247657_at	At5g59845	Gibberellin-regulated family protein	0.0080	0.0196	-4.6
250109_at	At5g15230	Gibberellin-regulated protein 4; GASA4	0.0008	0.0069	-3.8
264195_at	At1g22690	Gibberellin-responsive protein, putative	0.0148	0.0268	-8.0
260221_at	At1g74670	Gibberellin-responsive protein, putative	0.0053	0.0163	-6.9
250415_at	At5g11210	Glutamate receptor family protein; GLR2	0.0001	0.0031	-11.9
250605_at	At5g07570	Glycine -rich protein	0.0386	0.0435	-15.9
263409_at	At2g04063	Glycine-rich protein	0.0004	0.0049	-14.7
249839_at	At5g23405	High mobility group; HMG1/2 family protein	0.0006	0.0063	-9.4

247634_at	At5g60520	Late embryogenesis abundant protein-related	0.0366	0.0422	-8.6
245340_at	At4g14420	Lesion inducing protein-related	0.0302	0.0382	-2.8
245267_at	At4g14060	Major latex protein-related	0.0361	0.0419	-5.4
265705_at	At2g03410	Mo25 family protein	0.0240	0.0340	-4.5
255017_at	At4g10100	Molybdenum cofactor synthesis family protein	0.0131	0.0253	-3.9
264617_at	At2g17660	Nitrate-responsive NOI protein, putative	0.0003	0.0043	-8.5
247317_at	At5g64060	No apical meristem; NAM family protein	0.0381	0.0432	-5.8
248469_at	At5g50820	No apical meristem; NAM family protein	0.0433	0.0463	-4.8
249940_at	At5g22380	No apical meristem; NAM family protein	0.0476	0.0487	-3.1
245987_at	At5g13180	No apical meristem; NAM family protein	0.0009	0.0073	-2.5
260851_at	At1g21890	Nodulin MtN21 family protein	0.0000	0.0000	-11.1
261868_s_at	At1g11460	Nodulin MtN21 family protein	0.0160	0.0279	-3.9
260433_at	At1g68170	Nodulin MtN21 family protein	0.0085	0.0202	-2.6
261829_at	At1g10680	P-glycoprotein, putative	0.0084	0.0201	-3.8
257842_at	At3g28390	P-glycoprotein, putative	0.0388	0.0436	-2.5
245757_at	At1g35140	Phosphate-responsive protein, putative	0.0309	0.0387	-3.5
255064_at	At4g08950	Phosphate-responsive protein, putative	0.0197	0.0307	-3.2
267317_at	At2g34700	Pollen Ole e 1 allergen family protein	0.0211	0.0318	-16.0
260888_at	At1g29140	Pollen Ole e 1 allergen family protein	0.0394	0.0439	-3.3
248149_at	At5g54855	Pollen Ole e 1 allergen family protein	0.0002	0.0032	-2.8
264399_at	At1g61780	Postsynaptic protein-related	0.0092	0.0210	-4.7
249083_at	At5g44140	Prohibitin, putative	0.0004	0.0051	-12.8
252346_at	At3g48650	Pseudogene; At14a-related protein	0.0347	0.0411	-4.1
245658_at	At1g28270	Rapid alkalinization factor; RALF family protein	0.0309	0.0386	-5.0
259106_at	At3g05490	Rapid alkalinization factor; RALF family protein	0.0146	0.0265	-4.1
245385_at	At4g14020	Rapid alkalinization factor; RALF family protein	0.0159	0.0277	-2.7
262249_at	At1g48380	Root hair initiation protein root hairless 1;RHL1	0.0110	0.0230	-2.6
246761_at	At5g27980	Seed maturation family protein	0.0155	0.0274	-11.4
264610_at	At1g04645	Self-incompatibility protein-related	0.0274	0.0363	-17.7
250325_s_at	At5g12060	Self-incompatibility protein-related	0.0038	0.0142	-13.4
249868_at	At5g23030	Senescence-associated family protein	0.0279	0.0367	-3.7
267293_at	At2g23810	Senescence-associated family protein	0.0104	0.0223	-3.0
260109_at	At1g63260	Senescence-associated family protein	0.0172	0.0286	-2.7
252348_at	At3g48140	Senescence-associated family protein	0.0015	0.0092	-2.8
247897_at	At5g57810	Senescence-associated family protein	0.0378	0.0430	-5.5
248820_at	At5g47060	Senescence-associated family protein	0.0399	0.0442	-2.5
252295_at	At3g49100	Signal recognition particle protein, putative; SRP9	0.0346	0.0410	-2.9
260569_at	At2g43640	Signal recognition particle; SRP14 family protein	0.0087	0.0204	-3.6
251492_at	At3g59280	Signaling molecule-related	0.0191	0.0302	-3.2
263794_at	At2g24620	S-locus glycoprotein family protein	0.0172	0.0286	-2.6
265862_at	At2g01780	S-locus glycoprotein, putative	0.0230	0.0332	-3.8
252416_at	At3g47460	SMC2-like condensin, putative	0.0392	0.0438	-3.9
262714_s_at	At1g16430	Surfeit locus protein 5 family protein	0.0168	0.0283	-3.0
262511_at	At1g11250	Syntaxin, putative; SYP125	0.0437	0.0466	-3.1
250209_at	At5g14030	Translocon-associated protein beta; TRAPB family protein	0.0008	0.0069	-3.1
265356_at	At2g16595	Translocon-associated protein;TRAP, putative	0.0490	0.0495	-3.9
D1. Chloroplast					
255046_at	At4g09650	ATP synthase delta chain chloroplast, putative	0.0343	0.0408	-2.6
255659_at	At4g00895	ATP synthase delta chain chloroplast, putative	0.0046	0.0153	-2.8
245924_at	At5g28750	Thylakoid assembly protein, putative	0.0312	0.0388	-3.3
251031_at	At5g02120	Thylakoid membrane one helix protein;OHP	0.0133	0.0254	-6.1
E. Metabolism/energy/membrane associated proteins					
E1. Carbohydrate/soluble sugar/startch/aminoacid metabolism					
265658_at	At2g13810	Aminotransferase class I and II family protein	0.0000	0.0006	-8.9
253680_at	At4g29620	Cytidine aminohydrolase, putative	0.0147	0.0267	-4.5
253676_at	At4g29570	Cytidine aminohydrolase, putative	0.0497	0.0499	-2.9
252369_at	At3g48540	Cytidine/deoxycytidylate deaminase family protein	0.0413	0.0451	-4.9
256633_at	At3g28340	Galactinol synthase, putative	0.0010	0.0074	-7.9
261239_at	At1g32930	Galactosyltransferase family protein	0.0014	0.0089	-11.2
249729_at	At5g24410	Glucosamine/galactosamine-6-phosphate isomerase-related	0.0203	0.0312	-9.1
248368_at	At5g51950	Glucose-methanol-choline;GMC oxidoreductase family protein	0.0353	0.0414	-4.5
260743_at	At1g15040	Glutamine amidotransferase-related	0.0029	0.0125	-6.4
249983_at	At5g18470	Mannose-binding lectin family protein	0.0001	0.0028	-3.0
259749_at	At1g71100	Ribose 5-phosphate isomerase-related	0.0107	0.0226	-6.8
246333_at	At3g44840	S-adenosyl-L-methionine:carboxyl methyltransferase family protein	0.0172	0.0286	-4.2
256319_at	At1g35910	Trehalose-6-phosphate phosphatase, putative	0.0257	0.0353	-3.3

259185_at	At3g01550	Triose phosphate/phosphate translocator, putative	0.0496	0.0498	-2.9
E2. Primary /secondary metabolism					
260324_at	At1g63970	2C-methyl-D-erythritol 2;4-cyclodiphosphate synthase, putative	0.0050	0.0158	-3.2
267571_at	At2g30650	3-hydroxyisobutyryl-coenzyme A hydrolase, putative	0.0001	0.0026	-6.3
250062_at	At5g17760	AAA-type ATPase family protein	0.0012	0.0084	-19.9
253846_at	At4g28000	AAA-type ATPase family protein	0.0070	0.0183	-13.5
267499_at	At2g45500	AAA-type ATPase family protein	0.0332	0.0400	-6.3
266830_at	At2g22810	ACC synthase 4; ACS4	0.0113	0.0233	-3.0
246172_s_at	At5g28360	ACC synthase, putative	0.0157	0.0276	-2.9
264162_at	At1g65290	Acyl carrier family protein / ACP family protein	0.0085	0.0201	-3.5
252780_at	At3g42960	Alcohol dehydrogenase; ATA1	0.0225	0.0329	-3.8
257644_at	At3g25780	Allene oxide cyclase, putative	0.0246	0.0344	-4.7
260706_at	At1g32350	Alternative oxidase, putative	0.0372	0.0425	-3.3
254335_at	At4g22260	Alternative oxidase, putative	0.0043	0.0147	-2.8
255299_at	At4g04880	AMP deaminase family protein	0.0127	0.0248	-3.1
259182_at	At3g01750	Ankyrin repeat family protein	0.0000	0.0006	-28.4
248123_at	At5g54720	Ankyrin repeat family protein	0.0000	0.0018	-18.1
257806_at	At3g18670	Ankyrin repeat family protein	0.0198	0.0308	-15.1
262447_at	At1g49250	ATP dependent DNA ligase family protein	0.0064	0.0177	-10.9
256374_at	At1g66730	ATP dependent DNA ligase family protein	0.0205	0.0314	-4.9
256184_at	At1g51650	ATP synthase epsilon chain; mitochondrial	0.0033	0.0132	-5.7
248098_at	At5g55290	ATP synthase subunit H family protein	0.0304	0.0383	-3.3
263546_at	At2g21550	Bifunctional dihydrofolate reductase-thymidylate synthase, putative	0.0265	0.0357	-7.0
262454_at	At1g11190	Bifunctional nuclease; BFN1	0.0020	0.0103	-5.9
265572_at	At2g28210	Carbonic anhydrase family protein	0.0042	0.0146	-4.4
255300_at	At4g04870	CDP-alcohol phosphatidyltransferase family protein	0.0077	0.0193	-2.9
248217_at	At5g53560	Cytochrome B5 isoform 1	0.0043	0.0148	-4.1
261011_at	At1g26340	Cytochrome B5, putative	0.0309	0.0387	-4.3
245044_at	At2g26500	Cytochrome B6F complex subunit, putative	0.0090	0.0207	-3.8
247520_at	At5g61310	Cytochrome C oxidase	0.0214	0.0320	-4.1
261317_at	At1g53030	Cytochrome c oxidase copper chaperone family protein	0.0058	0.0170	-5.3
255011_at	At4g10040	Cytochrome C, putative	0.0213	0.0319	-3.3
264727_at	At1g22840	Cytochrome C, putative	0.0046	0.0152	-3.2
267626_at	At2g42250	Cytochrome P450 family protein	0.0035	0.0136	-14.1
256019_at	At1g58260	Cytochrome P450 family protein	0.0043	0.0149	-10.3
248964_at	At5g45340	Cytochrome P450 family protein	0.0009	0.0073	-4.8
261185_at	At1g34540	Cytochrome P450 family protein	0.0047	0.0154	-4.6
258973_at	At3g01900	Cytochrome P450 family protein	0.0209	0.0317	-3.8
257142_at	At3g20090	Cytochrome P450 family protein	0.0281	0.0369	-3.6
267505_at	At2g45560	Cytochrome P450 family protein	0.0186	0.0299	-2.6
249484_at	At5g38970	Cytochrome P450, putative	0.0068	0.0181	-17.9
261878_at	At1g50560	Cytochrome P450, putative	0.0158	0.0276	-5.9
259429_at	At1g01600	Cytochrome P450, putative	0.0209	0.0316	-4.2
257623_at	At3g26210	Cytochrome P450, putative; CYP71B23	0.0288	0.0373	-2.9
245548_at	At4g15310	Cytochrome P450-related	0.0142	0.0262	-10.1
246486_at	At5g15910	Dehydrogenase-related	0.0143	0.0263	-2.7
247409_at	At5g62980	Dihydroneopterin aldolase, putative	0.0152	0.0271	-2.6
250129_at	At5g16450	Dimethylmenaquinone methyltransferase family protein	0.0168	0.0283	-3.7
247763_at	At5g59180	DNA-directed RNA polymerase II	0.0053	0.0163	-3.5
252041_at	At3g52090	DNA-directed RNA polymerase II ; RPB13.6	0.0034	0.0134	-2.7
245367_at	At4g16265	DNA-directed RNA polymerase II, putative	0.0000	0.0016	-4.4
264431_at	At1g61700	DNA-directed RNA polymerase II, putative; RPB10	0.0257	0.0353	-4.8
263596_at	At2g01900	Endonuclease/exonuclease/phosphatase family protein	0.0111	0.0231	-8.6
266011_at	At2g37440	Endonuclease/exonuclease/phosphatase family protein	0.0012	0.0084	-6.2
250406_at	At5g10810	Enhancer of rudimentary protein, putative	0.0021	0.0106	-4.3
266928_at	At2g45790	Eukaryotic phosphomannomutase family protein	0.0038	0.0141	-2.8
256468_at	At1g32550	Ferredoxin family protein	0.0056	0.0166	-2.8
263624_at	At2g04700	Ferredoxin thioredoxin reductase catalytic beta chain family protein	0.0076	0.0191	-3.2
251414_at	At3g60370	FKBP-type peptidyl-prolyl cis-trans isomerase family protein	0.0128	0.0249	-2.7
252853_at	At4g39710	FKBP-type peptidyl-prolyl cis-trans isomerase, putative	0.0011	0.0081	-2.9
260901_at	At1g21430	Flavin-containing monooxygenase family protein	0.0166	0.0282	-11.4
256012_at	At1g19250	Flavin-containing monooxygenase family protein	0.0047	0.0154	-8.6
265105_s_at	At1g63340	Flavin-containing monooxygenase-related	0.0026	0.0118	-5.3
255420_at	At4g03240	Frataxin protein-related	0.0051	0.0160	-12.8
255865_at	At2g30330	GCN5L1 family protein	0.0190	0.0301	-3.7
267145_at	At2g38130	GCN5-related N-acetyltransferase, putative	0.0291	0.0374	-2.6
250390_at	At5g11340	GCN5-related N-acetyltransferase;GNAT family protein	0.0023	0.0110	-4.0

246518_at	At5g15770	GCN5-related N-acetyltransferase;GNAT family protein	0.0034	0.0133	-3.3
262196_at	At1g77870	Geranyl Geranyl protein ATGP4	0.0450	0.0474	-2.6
266068_at	At2g18640	Geranylgeranyl pyrophosphate synthase, putative / GGPP synthetase, putative / f	0.0370	0.0425	-6.4
259445_at	At1g02400	Gibberellin 2-oxidase, putative / GA2-oxidase, putative	0.0359	0.0419	-4.8
261530_at	At1g63460	Glutathione peroxidase, putative	0.0410	0.0449	-5.5
254890_at	At4g11600	Glutathione peroxidase, putative	0.0237	0.0338	-3.7
263426_at	At2g31570	Glutathione peroxidase, putative	0.0422	0.0456	-2.5
264993_at	At1g67290	Glyoxal oxidase-related	0.0391	0.0438	-4.8
247727_at	At5g59490	Haloacid dehalogenase-like hydrolase family protein	0.0440	0.0468	-5.4
252876_at	At4g39970	Haloacid dehalogenase-like hydrolase family protein	0.0254	0.0351	-2.7
267210_at	At2g30920	Hexaprenyldihydroxybenzoate methyltransferase	0.0023	0.0111	-3.4
247314_at	At5g64000	Inositol polyphosphate 1-phosphatase, putative	0.0150	0.0270	-2.7
258619_at	At3g02780	Isopentenyl diphosphate:dimethylallyl diphosphate isomerase II;IPP2	0.0001	0.0021	-2.5
247931_at	At5g57040	Lactoylglutathione lyase family protein	0.0069	0.0182	-2.6
246332_at	At3g44830	Lecithin:cholesterol acyltransferase family protein	0.0153	0.0272	-9.5
262755_at	At1g16360	Ligand-effect modulator 3 family protein; LEM3	0.0004	0.0051	-15.9
257832_at	At3g26740	Light responsive protein-related	0.0167	0.0282	-3.6
258100_at	At3g23550	MATE efflux family protein	0.0004	0.0050	-6.1
267046_at	At2g34360	MATE efflux family protein	0.0337	0.0404	-4.6
265151_at	At1g51340	MATE efflux family protein	0.0067	0.0181	-4.1
266126_at	At2g45040	Matrix metalloproteinase	0.0226	0.0330	-4.9
245345_at	At4g16640	Matrix metalloproteinase, putative	0.0491	0.0495	-7.6
259247_at	At3g07570	Membrane protein, putative	0.0000	0.0009	-3.6
259926_at	At1g75090	Methyladenine glycosylase family protein	0.0019	0.0101	-2.7
260669_at	At1g19340	Methyltransferase MT-A70 family protein	0.0368	0.0423	-6.7
258941_at	At3g09940	Monodehydroascorbate reductase, putative	0.0009	0.0073	-3.6
248405_at	At5g51480	Multi-copper oxidase type I family protein	0.0022	0.0107	-8.7
265127_at	At1g55560	Multi-copper oxidase type I family protein	0.0151	0.0271	-3.7
260849_at	At1g21860	Multi-copper oxidase type I family protein	0.0263	0.0356	-3.1
248923_at	At5g45940	MutT/nudix family protein	0.0446	0.0472	-9.5
245777_at	At1g73540	MutT/nudix family protein	0.0003	0.0044	-6.5
257830_at	At3g26690	MutT/nudix family protein	0.0010	0.0074	-5.2
265872_at	At2g01670	MutT/nudix family protein	0.0346	0.0410	-5.1
248793_at	At5g47240	MutT/nudix family protein	0.0181	0.0294	-3.5
261212_at	At1g12880	MutT/nudix family protein	0.0054	0.0164	-3.2
254784_at	At4g12720	MutT/nudix family protein	0.0014	0.0087	-2.5
258870_at	At3g03080	NADP-dependent oxidoreductase, putative	0.0002	0.0038	-14.4
249600_s_at	At5g38000	NADP-dependent oxidoreductase, putative	0.0003	0.0043	-3.7
246465_at	At5g17000	NADP-dependent oxidoreductase, putative	0.0314	0.0389	-3.0
245820_at	At1g26320	NADP-dependent oxidoreductase, putative	0.0110	0.0230	-2.8
261449_at	At1g21120	O-methyltransferase, putative	0.0238	0.0339	-2.8
256616_at	At3g22260	OTU-like cysteine protease family protein	0.0211	0.0318	-3.0
266875_at	At2g44800	Oxidoreductase; 2OG-Fe;II oxygenase family protein	0.0003	0.0043	-7.7
264592_at	At2g17720	Oxidoreductase; 2OG-Fe;II oxygenase family protein	0.0001	0.0019	-2.9
246098_at	At5g20400	Oxidoreductase; 2OG-Fe;II oxygenase family protein	0.0006	0.0063	-2.5
255883_at	At1g20270	Oxidoreductase; 2OG-Fe;II oxygenase family protein	0.0089	0.0207	-2.5
255558_at	At4g01900	P II nitrogen sensing protein; GLB I	0.0016	0.0094	-3.2
261554_at	At1g63400	Pentatricopeptide;PPR repeat-containing protein	0.0039	0.0144	-45.7
266602_at	At2g46050	Pentatricopeptide;PPR repeat-containing protein	0.0095	0.0213	-20.4
250166_at	At5g15300	Pentatricopeptide;PPR repeat-containing protein	0.0030	0.0126	-17.4
250725_at	At5g06400	Pentatricopeptide;PPR repeat-containing protein	0.0080	0.0197	-15.6
249222_at	At5g42450	Pentatricopeptide;PPR repeat-containing protein	0.0009	0.0073	-14.7
259698_at	At1g68930	Pentatricopeptide;PPR repeat-containing protein	0.0005	0.0058	-14.3
259798_at	At1g64310	Pentatricopeptide;PPR repeat-containing protein	0.0010	0.0075	-4.7
253430_at	At4g32430	Pentatricopeptide;PPR repeat-containing protein	0.0042	0.0147	-4.2
257979_at	At3g20730	Pentatricopeptide;PPR repeat-containing protein	0.0258	0.0353	-4.2
253932_at	At4g26800	Pentatricopeptide;PPR repeat-containing protein	0.0357	0.0416	-3.5
259295_at	At3g05340	Pentatricopeptide;PPR repeat-containing protein	0.0081	0.0198	-2.8
264946_at	At1g77010	Pentatricopeptide;PPR repeat-containing protein	0.0277	0.0366	-2.7
254288_at	At4g22970	Peptidase C50 family protein	0.0269	0.0360	-4.2
245190_at	At1g67690	Peptidase M3 family protein	0.0000	0.0017	-4.1
262154_at	At1g52700	Phospholipase/carboxylesterase family protein	0.0165	0.0282	-15.6
259329_at	At3g16360	Phosphotransfer family protein	0.0027	0.0120	-2.7
248333_at	At5g52390	Photoassimilate-responsive protein, putative	0.0216	0.0322	-7.8
247320_at	At5g64040	Photosystem I reaction center subunit PSI-N	0.0001	0.0027	-2.9
257260_at	At3g22104	Phototropic-responsive NPH3 protein-related	0.0370	0.0424	-3.7
253924_at	At4g27110	Phytochelatin synthetase-related	0.0216	0.0321	-7.2
251026_at	At5g02200	Phytochrome A specific signal transduction component-related	0.0091	0.0208	-2.6

266799_at	At2g22860	Phytosulfokines 2; PSK2	0.0026	0.0118	-4.9
247109_at	At5g65870	Phytosulfokines 5; PSK5	0.0084	0.0200	-4.9
252624_at	At3g44735	Phytosulfokines-related	0.0010	0.0075	-6.0
256158_at	At1g13590	Phytosulfokines-related	0.0066	0.0179	-3.4
261834_at	At1g10640	Polygalacturonase, putative	0.0166	0.0282	-5.5
266147_at	At2g12230	Pseudogene; C-1-tetrahydrofolate synthase	0.0006	0.0063	-7.8
246715_at	At5g28230	Pseudogene; similar to glucose-6-phosphate/phosphate-translocator	0.0045	0.0151	-4.3
245866_s_at	At1g57990	Purine permease-related	0.0285	0.0371	-6.0
255587_at	At4g01480	Pyrophosphate phospho-hydrolase, putative	0.0022	0.0108	-2.6
245626_at	At1g56700	Pyrrolidone-carboxylate peptidase family protein	0.0161	0.0279	-2.6
249933_at	At5g22400	Rac GTPase activating protein, putative	0.0304	0.0383	-5.2
249085_at	At5g44170	Rapid response to Glucose protein 1	0.0464	0.0480	-2.5
263328_at	At2g15280	Reticulon family protein; RTNLB10	0.0397	0.0441	-2.8
265762_at	At2g01240	Reticulon family protein; RTNLB15	0.0285	0.0372	-4.6
262329_at	At1g64090	Reticulon family protein; RTNLB3	0.0166	0.0282	-2.5
253745_at	At4g29090	Reverse transcriptase, putative	0.0049	0.0157	-4.9
265926_at	At2g18600	RUB1-conjugating enzyme, putative	0.0015	0.0091	-2.9
246218_at	At4g36800	RUB1-conjugating enzyme, putative;RCE1	0.0288	0.0373	-3.6
256290_at	At3g12203	Serine carboxypeptidase S10 family protein	0.0150	0.0270	-3.9
266755_at	At2g47150	Short-chain dehydrogenase/reductase; SDR family protein	0.0303	0.0383	-8.3
248520_at	At5g50600	Short-chain dehydrogenase/reductase; SDR family protein	0.0162	0.0280	-3.4
266265_at	At2g29340	Short-chain dehydrogenase/reductase; SDR family protein	0.0395	0.0440	-3.1
260050_at	At1g29960	Signal peptidase I family protein / MADS-box protein-related	0.0168	0.0283	-10.1
262161_at	At1g52600	Signal peptidase, putative	0.0007	0.0068	-3.3
266709_at	At2g03120	Signal peptide peptidase family protein	0.0246	0.0345	-2.8
261799_at	At1g30470	SIT4 phosphatase-associated family protein	0.0114	0.0234	-3.2
266584_s_at	At2g14920	Sulfotransferase family protein	0.0096	0.0214	-11.9
264595_at	At1g04750	Synaptobrevin family protein	0.0014	0.0089	-3.2
245167_s_at	At2g33120	Synaptobrevin-related protein	0.0327	0.0398	-4.2
261976_at	At1g37063	Transposase-related	0.0148	0.0268	-10.6
247639_s_at	At5g60500	UPP synthetase family protein	0.0339	0.0405	-8.1
249685_at	At5g36120	YGGT family protein	0.0038	0.0141	-5.1
257522_at	At3g08990	Yippee family protein	0.0083	0.0199	-4.6
248187_at	At5g53940	Yippee family protein	0.0132	0.0253	-2.7
E3. Glycolysis					
252300_at	At3g49160	Pyruvate kinase family protein	0.0191	0.0302	-3.0
E4. Clathrin binding					
258710_s_at	At3g09800	Clathrin adaptor complex small chain family protein	0.0035	0.0135	-4.6
264847_at	At2g17380	Clathrin assembly protein AP19	0.0054	0.0164	-4.3
261733_at	At1g47830	Clathrin coat assembly protein, putative	0.0211	0.0318	-2.8
E5. Intracellular membrane/membrane bound organelle					
264150_at	At1g02090	COP9 signalosome complex subunit 7ii / CSN complex subunit 7ii;CSN7;COP1	0.0058	0.0169	-3.9
260418_s_at	At1g69750	Cox19 family protein	0.0072	0.0186	-3.5
258760_at	At3g10780	Emp24/gp25L/p24 family protein	0.0000	0.0010	-4.6
259256_at	At3g07680	Emp24/gp25L/p24 family protein	0.0227	0.0331	-2.9
258345_at	At3g22845	Emp24/gp25L/p24 family protein	0.0071	0.0185	-4.6
257104_at	At3g25040	ER lumen protein retaining receptor, putative / HDEL receptor, putative	0.0230	0.0332	-3.2
255816_at	At2g33470	Glycolipid transfer protein-related	0.0272	0.0362	-3.6
263839_at	At2g36900	Golgi SNAP receptor complex member	0.0049	0.0157	-2.5
248140_at	At5g54980	Integral membrane family protein	0.0041	0.0145	-4.0
264505_at	At1g09380	Integral membrane family protein	0.0295	0.0378	-3.5
259323_at	At3g05280	Integral membrane Yip1 family protein	0.0098	0.0217	-3.2
253452_at	At4g31930	Mitochondrial glycoprotein family protein	0.0227	0.0331	-2.7
267638_at	At2g42210	Mitochondrial import inner membrane translocase subunit Tim17/Tim22/Tim23	0.0041	0.0145	-2.6
266239_at	At2g29530	Mitochondrial import inner membrane translocase;TIM10	0.0030	0.0127	-3.3
265006_at	At1g61570	Mitochondrial import inner membrane translocase;TIM13	0.0056	0.0167	-3.5
249268_at	At5g41685	Mitochondrial import receptor subunit TOM7	0.0245	0.0343	-4.0
266890_at	At2g44620	NADH-ubiquinone oxidoreductase	0.0063	0.0175	-3.3
249959_at	At5g18800	NADH-ubiquinone oxidoreductase 19 kDa subunit;NDUFA8 family protein	0.0018	0.0098	-4.6
258881_at	At3g06310	NADH-ubiquinone oxidoreductase 19 kDa subunit;NDUFA8 family protein	0.0028	0.0120	-2.7
265219_at	At2g02050	NADH-ubiquinone oxidoreductase B18 subunit, putative	0.0049	0.0156	-2.7
248746_at	At5g47890	NADH-ubiquinone oxidoreductase B8 subunit, putative	0.0022	0.0108	-3.1
251186_at	At3g62790	NADH-ubiquinone oxidoreductase-related	0.0000	0.0004	-3.0
258846_at	At3g03070	NADH-ubiquinone oxidoreductase-related	0.0271	0.0362	-3.0
247011_at	At5g67590	NADH-ubiquinone oxidoreductase-related	0.0064	0.0177	-2.9

264923_s_at	At1g60740	Peroxiredoxin type 2, putative	0.0192	0.0303	-2.6
261739_at	At1g47750	Peroxisomal biogenesis factor 11 family protein	0.0203	0.0312	-3.7
255338_at	At4g04470	Peroxisomal membrane protein; PMP22	0.0247	0.0345	-3.2
248686_at	At5g48540	Secretory protein-related	0.0022	0.0108	-3.7
256544_at	At1g42560	Seven transmembrane MLO family protein	0.0003	0.0046	-6.3
260869_at	At1g43800	Stearoyl-ACP desaturase, putative	0.0135	0.0256	-7.4
249178_at	At5g42890	Sterol carrier protein 2;SCP-2 family protein	0.0391	0.0438	-4.4
254333_at	At4g22753	Sterol desaturase family protein	0.0043	0.0148	-2.6
249343_at	At5g40650	Succinate dehydrogenase; iron-sulphur subunit; mitochondrial;SDH2-2	0.0002	0.0034	-6.6
249373_at	At5g40670	Transmembrane family protein	0.0403	0.0445	-3.1
253436_at	At4g32470	Ubiquinol-cytochrome C reductase, putative	0.0328	0.0398	-3.2
262206_at	At2g01090	Ubiquinol-cytochrome C reductase, putative	0.0036	0.0138	-3.2
248221_at	At5g53530	Vacuolar protein sorting-associated protein 26	0.0086	0.0203	-3.0
267412_at	At2g34940	Vacuolar sorting receptor, putative	0.0207	0.0315	-3.0
258958_at	At3g01390	V-ATPase G subunit 1; VAG1	0.0009	0.0072	-3.0
254216_at	At4g23710	V-ATPase G subunit 2; VAG2	0.0135	0.0256	-3.6
256111_at	At1g16820	V-ATPase-related	0.0489	0.0494	-3.8
F. Proteins with binding function or cofactor requirement					
258971_at	At3g01990	ACT domain-containing protein;ACR6	0.0364	0.0421	-2.8
246267_at	At1g31812	Acyl-CoA binding protein / ACBP	0.0040	0.0145	-3.9
256838_at	At3g22950	ADP-ribosylation factor, putative	0.0083	0.0200	-3.6
248346_at	At5g52210	ADP-ribosylation factor, putative	0.0000	0.0017	-3.0
255927_at	At1g12630	AP2 domain-containing protein	0.0210	0.0317	-3.4
260856_at	At1g21910	AP2 domain-containing transcription factor family protein	0.0162	0.0280	-3.0
261101_at	At1g63030	AP2 domain-containing transcription factor, putative	0.0203	0.0312	-17.4
261984_at	At1g33760	AP2 domain-containing transcription factor, putative	0.0022	0.0107	-7.4
257581_s_at	At1g50680	AP2 domain-containing transcription factor, putative	0.0000	0.0019	-3.1
255827_at	At2g40600	Appr-1-p processing enzyme family protein	0.0000	0.0013	-3.4
254788_at	At4g12790	ATP-binding family protein	0.0169	0.0284	-2.7
252227_at	At3g49900	BTB/POZ domain-containing protein	0.0258	0.0353	-4.6
253061_at	At4g37610	BTB/POZ domain-containing protein	0.0021	0.0105	-8.3
251790_at	At3g55470	C2 domain-containing protein	0.0017	0.0098	-4.8
259879_at	At1g76650	Calcium-binding EF hand family protein	0.0019	0.0101	-6.6
253915_at	At4g27280	Calcium-binding EF hand family protein	0.0000	0.0004	-4.8
249417_at	At5g39670	Calcium-binding EF hand family protein	0.0328	0.0398	-4.2
257731_at	At3g18430	Calcium-binding EF hand family protein	0.0066	0.0179	-3.5
267366_at	At2g44310	Calcium-binding EF hand family protein	0.0047	0.0154	-3.2
262877_at	At1g64850	Calcium-binding EF hand family protein	0.0011	0.0078	-3.0
263003_at	At1g54450	Calcium-binding EF-hand family protein	0.0012	0.0084	-6.6
248164_at	At5g54490	Calcium-binding EF-hand protein, putative	0.0004	0.0053	-5.9
254782_at	At4g12860	Calcium-binding protein, putative	0.0027	0.0120	-12.2
265460_at	At2g46600	Calcium-binding protein, putative	0.0039	0.0142	-5.0
260076_at	At1g73630	Calcium-binding protein, putative	0.0122	0.0243	-4.7
260881_at	At1g21550	Calcium-binding protein, putative	0.0054	0.0164	-3.1
253282_at	At4g34120	CBS domain-containing protein	0.0414	0.0452	-2.5
262261_at	At1g70895	CLE17, putative	0.0343	0.0408	-3.6
254859_at	At4g12060	Clp amino terminal domain-containing protein	0.0092	0.0209	-4.2
256621_at	At3g24450	Copper-binding family protein	0.0165	0.0281	-13.1
263534_at	At2g24940	Cytochrome b5 domain-containing protein	0.0077	0.0192	-4.1
264926_at	At1g60660	Cytochrome b5 domain-containing protein	0.0050	0.0158	-2.7
249603_at	At5g37210	DC1 domain-containing protein	0.0001	0.0026	-9.6
248182_at	At5g54030	DC1 domain-containing protein	0.0087	0.0204	-8.3
259369_s_at	At1g69150	DC1 domain-containing protein	0.0239	0.0339	-7.1
258540_at	At3g06990	DC1 domain-containing protein	0.0163	0.0280	-7.0
252582_at	At3g45530	DC1 domain-containing protein	0.0003	0.0042	-5.6
267384_at	At2g44370	DC1 domain-containing protein	0.0299	0.0380	-4.5
258541_at	At3g07000	DC1 domain-containing protein	0.0403	0.0445	-4.3
267252_at	At2g23100	DC1 domain-containing protein	0.0187	0.0300	-4.3
259454_at	At1g44050	DC1 domain-containing protein	0.0021	0.0105	-3.4
260893_at	At1g29180	DC1 domain-containing protein	0.0328	0.0398	-2.6
267385_at	At2g44380	DC1 domain-containing protein	0.0019	0.0101	-2.5
247674_at	At5g59930	DC1 domain-containing protein / UV-B light-insensitive protein, putative	0.0397	0.0441	-4.3
259364_at	At1g13260	DNA-binding protein RAV1;RAV1	0.0004	0.0049	-2.5
251302_at	At3g61970	DNA-binding protein, putative	0.0066	0.0179	-3.3
248412_at	At5g51590	DNA-binding protein-related	0.0419	0.0454	-5.5
253108_at	At4g35900	DNA-binding protein-related	0.0075	0.0190	-3.1
247144_at	At5g65590	Dof-type zinc finger domain-containing protein	0.0086	0.0202	-6.2

254441_at	At4g21050	Dof-type zinc finger domain-containing protein	0.0163	0.0280	-5.6
262876_at	At1g64750	DSS1/SEM1 family protein	0.0429	0.0461	-3.7
248972_at	At5g45010	DSS1/SEM1 family protein	0.0027	0.0120	-3.4
261470_at	At1g28370	ERF domain protein 11;ERF11	0.0500	0.0500	-2.8
248799_at	At5g47230	Ethylene-responsive element-binding factor 5;ERF5	0.0333	0.0401	-3.0
256722_at	At2g34050	Eukaryotic putative RNA binding region RNP-1	0.0346	0.0410	-5.7
262817_at	At1g11770	FAD-binding domain-containing protein	0.0072	0.0187	-7.5
266711_at	At2g46740	FAD-binding domain-containing protein	0.0195	0.0305	-4.8
245538_at	At4g15200	Formin homology 2 domain-containing protein	0.0273	0.0363	-10.5
246034_at	At5g08350	GRAM domain-containing protein / ABA-responsive protein-related	0.0072	0.0186	-2.6
256224_at	At1g56330	GTP-binding protein;SAR1B	0.0420	0.0455	-2.5
250866_at	At5g03905	HesB-like domain-containing protein	0.0164	0.0281	-5.2
263256_at	At1g10500	HesB-like domain-containing protein	0.0072	0.0187	-3.4
258791_at	At3g04720	Hevein-like protein; HEL	0.0005	0.0055	-4.1
247344_at	At5g63750	IBR domain-containing protein	0.0211	0.0318	-3.4
267049_at	At2g34210	KOW domain-containing transcription factor family protein	0.0324	0.0396	-11.4
259056_at	At3g03420	Ku70-binding family protein	0.0423	0.0457	-2.7
245087_at	At2g39830	LIM domain-containing protein	0.0030	0.0127	-2.7
255907_at	At1g17920	Lipid-binding START domain-containing protein	0.0273	0.0363	-3.0
254330_at	At4g22700	LOB domain family protein / lateral organ boundaries domain family protein;LB	0.0008	0.0071	-15.0
260265_at	At1g68510	LOB domain protein 42 / lateral organ boundaries domain protein 42;LBD42	0.0431	0.0463	-3.0
264152_at	At1g02140	Mago nashi family protein	0.0111	0.0231	-2.9
249814_at	At5g23840	MD-2-related lipid recognition domain-containing protein / ML domain-containi	0.0355	0.0415	-13.9
251576_at	At3g58200	Meprin and TRAF homology domain-containing protein	0.0031	0.0128	-8.8
246855_at	At5g26280	Meprin and TRAF homology domain-containing protein	0.0091	0.0208	-4.5
248354_at	At5g52330	Meprin and TRAF homology domain-containing protein	0.0279	0.0368	-2.7
254387_at	At4g21850	Methionine sulfoxide reductase domain-containing protein	0.0013	0.0085	-7.0
254385_s_at	At4g21830	Methionine sulfoxide reductase domain-containing protein	0.0058	0.0170	-3.2
255298_at	At4g04840	Methionine sulfoxide reductase domain-containing protein	0.0340	0.0406	-2.8
249138_at	At5g43070	MFP1 attachment factor, putative	0.0013	0.0086	-13.0
246757_at	At5g27940	MFP1 attachment factor, putative	0.0213	0.0319	-3.2
247979_at	At5g56750	Ndr family protein	0.0046	0.0153	-3.3
248963_at	At5g45700	NLI interacting factor;NIF family protein	0.0239	0.0340	-10.9
251511_at	At3g59180	Novel domain with similarity to F-Box domain	0.0170	0.0285	-5.3
267371_at	At2g44510	p21Cip1-binding protein-related	0.0054	0.0164	-3.9
255307_at	At4g04900	p21-rho-binding domain-containing protein	0.0490	0.0495	-5.2
257397_at	At2g20430	p21-rho-binding domain-containing protein	0.0009	0.0071	-2.5
246895_at	At5g25540	PAM2 PABC INTERACTING DOMAIN	0.0259	0.0354	-2.6
263703_at	At1g31170	parB-like nuclease domain-containing protein	0.0151	0.0271	-2.6
250110_at	At5g15350	Plastocyanin-like domain-containing protein	0.0265	0.0357	-3.6
247888_at	At5g57920	Plastocyanin-like domain-containing protein	0.0035	0.0135	-2.7
246099_at	At5g20230	Plastocyanin-like domain-containing protein	0.0475	0.0486	-2.6
265987_at	At2g24240	Potassium channel tetramerisation domain-containing protein	0.0108	0.0228	-6.7
260080_at	At1g78160	Pumilio/Puf RNA-binding domain-containing protein	0.0000	0.0006	-40.4
248022_at	At5g56510	Pumilio/Puf RNA-binding domain-containing protein	0.0030	0.0127	-8.1
252825_at	At4g39890	Ras-related GTP-binding family protein	0.0056	0.0167	-5.1
248726_at	At5g47960	Ras-related GTP-binding family protein	0.0013	0.0086	-4.2
254641_at	At4g18800	Ras-related GTP-binding family protein	0.0002	0.0032	-4.0
263876_at	At2g21880	Ras-related GTP-binding protein, putative	0.0064	0.0177	-13.6
245299_at	At4g17530	Ras-related GTP-binding protein, putative	0.0455	0.0477	-2.8
263398_at	At2g31680	Ras-related GTP-binding protein, putative	0.0238	0.0339	-2.6
259611_at	At1g52280	Ras-related GTP-binding protein, putative	0.0270	0.0361	-2.5
250923_at	At5g03455	Rhodanese-like domain-containing protein	0.0422	0.0456	-2.8
257909_at	At3g25480	Rhodanese-like domain-containing protein	0.0461	0.0479	-2.8
252767_at	At3g42830	Ring-box protein Roc1/Rbx1/Hrt1, putative	0.0084	0.0201	-6.4
246089_at	At5g20570	Ring-box protein-related	0.0126	0.0247	-2.7
252559_at	At3g46020	RNA-binding protein, putative	0.0247	0.0345	-6.2
246527_at	At5g15750	RNA-binding S4 domain-containing protein	0.0026	0.0118	-3.0
264868_at	At1g24090	RNase H domain-containing protein	0.0016	0.0094	-8.6
263181_at	At1g05720	Selenoprotein family protein	0.0085	0.0202	-3.6
266020_at	At2g05900	SET domain-containing protein	0.0170	0.0285	-4.1
248607_at	At5g49480	Sodium-responsive calcium-binding protein; ACP1	0.0124	0.0246	-5.4
266097_at	At2g37970	SOUL heme-binding family protein	0.0047	0.0154	-2.9
252370_at	At3g48600	SWIB complex BAF60b domain-containing protein	0.0009	0.0071	-8.4
259649_at	At1g55300	TATA-binding protein-associated factor TAFII55 family protein	0.0215	0.0321	-4.8
262374_s_at	At1g72930	Toll-Interleukin-Resistance;TIR domain-containing protein	0.0026	0.0119	-3.9
265782_at	At2g07440	Two-component responsive regulator-related	0.0189	0.0301	-3.2
256522_at	At1g66160	U-box domain-containing protein	0.0126	0.0247	-4.5

257748_at	At3g18710	U-box domain-containing protein	0.0038	0.0141	-3.0
258105_at	At3g23605	UBX domain-containing protein	0.0253	0.0350	-2.5
266800_at	At2g22880	VQ motif-containing protein	0.0056	0.0167	-28.4
259552_at	At1g21320	VQ motif-containing protein	0.0026	0.0118	-13.6
251617_at	At3g58000	VQ motif-containing protein	0.0058	0.0169	-8.4
260261_at	At1g68450	VQ motif-containing protein	0.0159	0.0277	-6.1
260804_at	At1g78410	VQ motif-containing protein	0.0229	0.0332	-4.0
247230_at	At5g65170	VQ motif-containing protein	0.0406	0.0447	-2.7
248868_at	At5g46780	VQ motif-containing protein	0.0109	0.0229	-2.6
245814_at	At1g49910	WD-40 repeat family protein, putative	0.0032	0.0130	-14.0
248870_at	At5g46710	Zinc-binding family protein	0.0000	0.0012	-3.6
F1. Metal ion binding/metal binding					
248322_at	At5g52760	Heavy-metal-associated domain-containing protein	0.0013	0.0085	-13.7
248327_at	At5g52750	Heavy-metal-associated domain-containing protein	0.0014	0.0089	-10.1
245829_at	At1g57780	Heavy-metal-associated domain-containing protein	0.0150	0.0270	-9.9
248715_at	At5g48290	Heavy-metal-associated domain-containing protein	0.0382	0.0433	-8.7
248321_at	At5g52740	Heavy-metal-associated domain-containing protein	0.0154	0.0273	-7.7
255254_at	At4g05030	Heavy-metal-associated domain-containing protein	0.0138	0.0258	-2.7
246548_at	At5g14910	Heavy-metal-associated domain-containing protein	0.0192	0.0303	-2.6
259753_at	At1g71050	Heavy-metal-associated domain-containing protein	0.0003	0.0048	-3.7
257054_at	At3g15353	Metallothionein protein, putative	0.0021	0.0105	-5.5
261438_at	At1g07600	Metallothionein-like protein 1A	0.0005	0.0056	-2.9
261410_at	At1g07610	Metallothionein-like protein 1C	0.0025	0.0115	-3.4
G. Protein synthesis and translation					
255977_at	At1g34030	40S ribosomal protein S18;RPS18B	0.0131	0.0252	-2.5
255000_at	At4g09800	40S ribosomal protein S18;RPS18C	0.0066	0.0179	-3.7
246730_at	At5g28060	40S ribosomal protein S24;RPS24B	0.0069	0.0182	-3.7
266705_at	At2g19750	40S ribosomal protein S30;RPS30A	0.0249	0.0346	-2.8
253715_at	At4g29390	40S ribosomal protein S30;RPS30B	0.0019	0.0101	-4.2
247968_at	At5g56670	40S ribosomal protein S30;RPS30C	0.0003	0.0042	-9.2
247010_at	At5g67510	60S ribosomal protein L26;RPL26B	0.0072	0.0186	-3.0
265730_at	At2g32220	60S ribosomal protein L27;RPL27A	0.0004	0.0053	-7.6
256794_at	At3g22230	60S ribosomal protein L27;RPL27B	0.0131	0.0252	-3.6
266699_at	At2g19730	60S ribosomal protein L28;RPL28A	0.0237	0.0338	-2.8
258532_at	At3g06700	60S ribosomal protein L29;RPL29A	0.0001	0.0027	-3.8
258521_at	At3g06680	60S ribosomal protein L29;RPL29B	0.0058	0.0169	-3.4
254012_at	At4g26230	60S ribosomal protein L31;RPL31B	0.0011	0.0079	-2.8
258709_at	At3g09500	60S ribosomal protein L35;RPL35A	0.0034	0.0135	-3.6
266980_at	At2g39390	60S ribosomal protein L35;RPL35B	0.0013	0.0087	-2.6
251007_at	At5g02610	60S ribosomal protein L35;RPL35D	0.0384	0.0434	-2.9
251926_at	At3g53740	60S ribosomal protein L36;RPL36B	0.0297	0.0379	-2.5
251018_at	At5g02450	60S ribosomal protein L36;RPL36C	0.0051	0.0160	-3.9
258296_at	At3g23390	60S ribosomal protein L36a/L44;RPL36aA	0.0186	0.0299	-3.8
245311_at	At4g14320	60S ribosomal protein L36a/L44;RPL36aB	0.0030	0.0127	-3.5
259612_at	At1g52300	60S ribosomal protein L37;RPL37B	0.0001	0.0026	-2.9
263810_at	At2g04520	Eukaryotic translation initiation factor 1A, putative / eIF-1A, putative / eIF-4C, p	0.0069	0.0182	-3.0
249711_at	At5g35680	Eukaryotic translation initiation factor 1A, putative / eIF-1A, putative / eIF-4C, p	0.0186	0.0299	-2.6
266245_at	At2g27700	Eukaryotic translation initiation factor 2 family protein / eIF-2 family protein	0.0258	0.0353	-14.4
260350_at	At1g69410	Eukaryotic translation initiation factor 5A, putative / eIF-5A, putative	0.0189	0.0301	-3.1
248146_at	At5g54940	Eukaryotic translation initiation factor SUI1, putative	0.0055	0.0165	-4.1
248126_at	At5g54760	Eukaryotic translation initiation factor SUI1, putative	0.0133	0.0254	-2.7
262283_at	At1g68590	Plastid-specific 30S ribosomal protein 3, putative	0.0323	0.0395	-3.6
248878_at	At5g46160	Ribosomal protein L14 family protein	0.0013	0.0084	-3.9
261418_at	At1g07830	Ribosomal protein L29 family protein	0.0162	0.0280	-2.6
249975_s_at	At5g18790	Ribosomal protein L33 family protein	0.0158	0.0276	-2.8
265338_at	At2g18400	Ribosomal protein L6 family protein	0.0036	0.0137	-3.0
254831_at	At4g12600	Ribosomal protein L7 family protein	0.0005	0.0055	-7.2
254355_at	At4g22380	Ribosomal protein L7 family protein	0.0027	0.0120	-3.2
264364_at	At1g03330	Small nuclear ribonucleoprotein D, putative	0.0009	0.0071	-4.5
266482_at	At2g47640	Small nuclear ribonucleoprotein D2, putative	0.0067	0.0181	-2.8
266579_at	At2g23930	Small nuclear ribonucleoprotein G, putative	0.0044	0.0150	-4.3
259288_at	At3g11500	Small nuclear ribonucleoprotein G, putative	0.0203	0.0312	-3.3
248678_at	At5g48870	Small nuclear ribonucleoprotein, putative	0.0017	0.0097	-3.6
H. Protein fate					
247789_at	At5g58680	Armadillo/beta-catenin repeat family protein	0.0006	0.0058	-3.0

254792_at	At4g12920	Aspartyl protease family protein	0.0321	0.0394	-8.9
255590_at	At4g01610	Cathepsin B-like cysteine protease, putative	0.0162	0.0280	-2.9
245323_at	At4g16500	Cysteine protease inhibitor family protein	0.0032	0.0131	-3.0
250316_at	At5g12140	Cysteine protease inhibitor, putative	0.0253	0.0350	-5.0
245096_at	At2g40880	Cysteine protease inhibitor, putative	0.0009	0.0071	-4.9
256714_at	At2g34080	Cysteine proteinase, putative	0.0297	0.0379	-4.6
249363_at	At5g40560	DegP protease, putative	0.0090	0.0207	-10.8
257437_s_at	At2g16290	F-box family protein	0.0000	0.0017	-16.7
252299_at	At3g49150	F-box family protein	0.0228	0.0332	-14.6
248456_at	At5g51380	F-box family protein	0.0004	0.0052	-10.7
255445_at	At4g02740	F-box family protein	0.0247	0.0345	-8.9
266675_s_at	At2g29610	F-box family protein	0.0253	0.0350	-6.4
263283_at	At2g36090	F-box family protein	0.0368	0.0423	-5.5
245653_at	At4g13985	F-box family protein	0.0402	0.0444	-4.1
265632_at	At2g14290	F-box family protein	0.0076	0.0191	-3.8
257537_at	At3g22350	F-box family protein	0.0229	0.0332	-3.3
264758_at	At1g61340	F-box family protein	0.0301	0.0381	-3.2
256432_at	At3g10990	F-box family protein	0.0271	0.0362	-2.8
260310_at	At1g70590	F-box family protein	0.0112	0.0233	-2.6
265620_at	At2g27310	F-box family protein	0.0018	0.0098	-2.6
265140_at	At1g51320	F-box family protein;FBX11	0.0025	0.0115	-8.1
252455_at	At3g47140	F-box family protein-related	0.0426	0.0459	-5.6
259795_at	At1g64290	F-box protein-related	0.0298	0.0380	-5.4
258606_at	At3g02840	Immediate-early fungal elicitor family protein	0.0011	0.0081	-6.3
246709_s_at	At5g28160	Kelch repeat-containing F-box family protein	0.0062	0.0174	-19.4
254212_at	At4g23580	Kelch repeat-containing F-box family protein	0.0004	0.0049	-8.6
256750_at	At3g27150	Kelch repeat-containing F-box family protein	0.0260	0.0355	-2.7
254567_s_at	At4g19260	Kelch repeat-containing protein	0.0004	0.0052	-6.1
255101_at	At4g08670	Protease inhibitor	0.0154	0.0273	-15.9
248062_at	At5g55450	Protease inhibitor	0.0010	0.0078	-13.9
254805_at	At4g12480	Protease inhibitor	0.0448	0.0473	-6.7
245322_at	At4g14815	Protease inhibitor	0.0000	0.0012	-6.4
250764_at	At5g05960	Protease inhibitor	0.0156	0.0275	-4.6
248683_at	At5g48490	Protease inhibitor	0.0133	0.0254	-4.0
263195_at	At1g36150	Protease inhibitor	0.0177	0.0291	-3.7
247462_at	At5g62080	Protease inhibitor	0.0109	0.0228	-3.6
248684_at	At5g48485	Protease inhibitor	0.0133	0.0254	-3.0
252090_at	At3g52130	Protease inhibitor	0.0240	0.0340	-2.5
249101_at	At5g43580	Protease inhibitor, putative	0.0005	0.0056	-45.7
266168_at	At2g38870	Protease inhibitor, putative	0.0020	0.0105	-3.9
247721_at	At5g59140	SKP1 family protein	0.0279	0.0368	-4.5
266178_at	At2g02280	SKP1 INTERACTING PARTNER 3	0.0004	0.0050	-3.2
250345_at	At5g11940	Subtilase family protein	0.0000	0.0014	-6.9
247799_at	At5g58840	Subtilase family protein	0.0065	0.0178	-4.9
255904_at	At1g17860	Trypsin and protease inhibitor family protein	0.0259	0.0354	-7.9
260101_at	At1g73260	Trypsin and protease inhibitor family protein	0.0238	0.0339	-5.9
261235_x_at	At1g32840	Ulp1 protease family protein	0.0018	0.0099	-18.7
261287_at	At1g37020	Ulp1 protease family protein	0.0082	0.0199	-13.1
255217_s_at	At4g07680	Ulp1 protease famiy	0.0054	0.0164	-2.5

H1. Ubiquitin like conjugating enzyme activity

257943_at	At3g21840	E3 ubiquitin ligase SCF complex subunit; SKP1/ASK1	0.0031	0.0128	-3.2
257942_at	At3g21830	E3 ubiquitin ligase SCF complex subunit; SKP1/ASK2	0.0131	0.0252	-12.8
248103_at	At5g55160	Small ubiquitin-like modifier 2; SUMO	0.0004	0.0051	-4.6
257453_at	At1g65130	Ubiquitin carboxyl-terminal -related	0.0407	0.0448	-5.1
252056_at	At3g52590	Ubiquitin extension protein 1; UBQ1	0.0167	0.0283	-3.0
263289_at	At2g36170	Ubiquitin extension protein 2; UBQ2	0.0145	0.0265	-3.5
255464_s_at	At4g02970	Ubiquitin family protein	0.0366	0.0422	-5.0
254106_at	At4g24990	Ubiquitin family protein	0.0220	0.0324	-3.8
266626_at	At2g35360	Ubiquitin family protein	0.0218	0.0323	-3.4
257785_at	At3g26980	Ubiquitin family protein	0.0264	0.0356	-3.3
255951_at	At1g22050	Ubiquitin family protein	0.0068	0.0181	-3.2
249217_at	At5g42300	Ubiquitin family protein	0.0007	0.0064	-2.9
265357_at	At2g16740	Ubiquitin-conjugating enzyme, putative	0.0296	0.0379	-2.6
260964_at	At1g45050	Ubiquitin-conjugating enzyme 15;UBC15	0.0035	0.0135	-3.3
261110_at	At1g75440	Ubiquitin-conjugating enzyme 16;UBC16	0.0400	0.0443	-4.0
249299_at	At5g41340	Ubiquitin-conjugating enzyme 4;UBC4	0.0027	0.0119	-2.7
266604_at	At2g46030	Ubiquitin-conjugating enzyme 6;UBC6	0.0297	0.0379	-3.3

260180_at	At1g70660	Ubiquitin-conjugating enzyme family protein	0.0068	0.0181	-2.8
253826_s_at	At4g27960	Ubiquitin-conjugating enzyme; UBC9	0.0044	0.0150	-2.8
I. Transcription/splicing/RNA processing					
256363_at	At1g66510	AAR2 protein family	0.0026	0.0118	-4.7
266454_at	At2g22750	Basic helix-loop-helix; bHLH family protein	0.0188	0.0300	-8.4
260070_at	At1g73830	Basic helix-loop-helix; bHLH family protein	0.0021	0.0105	-7.6
261717_at	At1g18400	Basic helix-loop-helix; bHLH family protein	0.0031	0.0128	-3.9
259898_at	At1g71200	Basic helix-loop-helix; bHLH family protein	0.0176	0.0290	-3.6
248839_at	At5g46690	Basic helix-loop-helix; bHLH family protein	0.0149	0.0269	-2.9
261941_at	At1g22490	Basic helix-loop-helix; bHLH family protein	0.0234	0.0336	-2.7
256395_at	At3g06120	Basic helix-loop-helix; bHLH family protein	0.0367	0.0423	-2.6
245331_at	At4g14410	Basic helix-loop-helix; bHLH family protein	0.0034	0.0134	-2.5
246212_at	At4g36930	Basic helix-loop-helix; bHLH protein	0.0088	0.0205	-7.0
250155_at	At5g15160	BHLH family protein	0.0300	0.0381	-10.2
249383_at	At5g39860	BHLH family protein	0.0061	0.0173	-6.5
248606_at	At5g49450	bZIP family transcription factor	0.0000	0.0006	-4.9
265756_at	At2g13150	bZIP family transcription factor	0.0024	0.0112	-9.8
249534_at	At5g38800	bZIP transcription factor family protein	0.0046	0.0152	-8.5
263064_at	At2g18160	bZIP transcription factor family protein	0.0234	0.0336	-4.8
263647_at	At2g04690	Cellular repressor of E1A-stimulated genes; CREG family	0.0020	0.0104	-4.6
267588_at	At2g42060	CHP-rich zinc finger protein, putative	0.0074	0.0188	-3.6
255618_at	At4g01340	CHP-rich zinc finger protein-related	0.0006	0.0058	-18.5
251551_at	At3g58680	Ethylene-responsive transcriptional coactivator, putative	0.0447	0.0472	-2.9
247767_at	At5g58890	MADS-box family protein	0.0094	0.0212	-8.6
250476_at	At5g10140	MADS-box protein flowering locus F;FLF	0.0016	0.0094	-15.0
251312_at	At3g61120	MADS-box protein;AGL13	0.0241	0.0340	-2.9
254896_at	At4g11880	MADS-box protein;AGL14	0.0009	0.0072	-17.6
267403_at	At2g26320	MADS-box protein;AGL33	0.0013	0.0086	-24.9
247469_at	At5g62165	MADS-box protein;AGL42	0.0000	0.0009	-16.3
267528_at	At2g45650	MADS-box protein;AGL6	0.0187	0.0300	-6.0
263295_at	At2g14210	MADS-box protein;ANR1	0.0046	0.0152	-2.9
260281_at	At1g80500	MBP-1 Interacting protein 2A	0.0040	0.0144	-2.7
257163_at	At3g24310	Myb family transcription factor	0.0472	0.0485	-10.3
263549_at	At2g21650	Myb family transcription factor	0.0205	0.0314	-9.6
249820_at	At5g23650	Myb family transcription factor	0.0017	0.0098	-4.4
255037_at	At4g09460	Myb family transcription factor	0.0391	0.0438	-2.7
251223_at	At3g62610	Myb family transcription factor	0.0058	0.0169	-2.6
263615_at	At2g25230	Myb family transcription factor; Myb100	0.0340	0.0406	-7.1
267087_at	At2g32460	Myb family transcription factor; Myb101	0.0002	0.0034	-16.2
266469_at	At2g31180	Myb family transcription factor; Myb14	0.0073	0.0188	-3.5
257919_at	At3g23250	Myb family transcription factor; Myb15	0.0005	0.0057	-3.9
259822_at	At1g66230	Myb family transcription factor; Myb20	0.0272	0.0363	-5.1
251974_at	At3g53200	Myb family transcription factor; Myb27	0.0035	0.0135	-3.4
258237_at	At3g27810	Myb family transcription factor; Myb3	0.0052	0.0162	-11.8
250051_at	At5g17800	Myb family transcription factor; Myb56	0.0006	0.0058	-3.6
256155_at	At3g08500	Myb family transcription factor; Myb83	0.0131	0.0252	-4.3
248246_at	At5g53200	Myb family transcription factor; TRIPTYCHON	0.0273	0.0363	-3.5
247772_at	At5g58610	PHD finger transcription factor, putative	0.0050	0.0158	-4.1
250410_at	At5g11010	Pre-mRNA cleavage complex-related	0.0186	0.0299	-3.2
245942_at	At5g19490	Repressor protein-related	0.0000	0.0014	-3.1
265641_at	At2g27330	RNA recognition motif;RRM-containing protein	0.0031	0.0128	-12.9
259409_at	At1g13330	TBP-1 INTERACTING PROTEIN	0.0001	0.0022	-2.9
264671_at	At1g09920	TRAF-type zinc finger-related	0.0135	0.0256	-2.8
249246_at	At5g42290	Transcription activator-related	0.0450	0.0474	-5.9
258079_at	At3g25940	Transcription factor S-II; TFIIS	0.0443	0.0470	-10.1
250932_s_at	At5g03220	Transcriptional co-activator-related	0.0193	0.0303	-3.4
253375_at	At4g33280	Transcriptional factor B3 family protein	0.0060	0.0172	-8.7
250026_at	At5g18090	Transcriptional factor B3 family protein	0.0327	0.0398	-7.8
250071_at	At5g18000	Transcriptional factor B3 family protein	0.0037	0.0139	-7.2
261260_at	At1g26680	Transcriptional factor B3 family protein	0.0392	0.0438	-3.6
254910_at	At4g11175	Translation initiation factor IF-1, putative	0.0037	0.0139	-3.8
253514_at	At4g31805	WRKY family transcription factor	0.0155	0.0274	-4.5
254231_at	At4g23810	WRKY family transcription factor	0.0028	0.0120	-3.6
267469_at	At2g30590	WRKY family transcription factor	0.0313	0.0389	-2.8
259320_at	At3g01080	WRKY family transcription factor	0.0368	0.0423	-2.5
265333_at	At2g18350	Zinc finger homeobox family protein / ZF-HD homeobox family protein	0.0132	0.0253	-3.0
265485_at	At2g15550	Zinc finger protein	0.0265	0.0357	-8.4

254423_at	At4g21610	Zinc finger protein, putative	0.0043	0.0148	-2.6
258230_s_at	At3g27710	Zinc finger protein-related	0.0168	0.0283	-11.7
265607_at	At2g25370	Zinc finger protein-related	0.0118	0.0238	-4.5
254861_at	At4g12040	Zinc finger; AN1-like family protein	0.0287	0.0372	-4.1
256231_at	At3g12630	Zinc finger; AN1-like family protein	0.0185	0.0298	-3.3
256576_at	At3g28210	Zinc finger; AN1-like family protein	0.0021	0.0106	-2.8
263537_at	At2g24790	Zinc finger; B-box type family protein	0.0038	0.0141	-2.8
257262_at	At3g21890	Zinc finger; B-box type family protein	0.0317	0.0391	-2.7
248160_at	At5g54470	Zinc finger; B-box type family protein	0.0255	0.0352	-2.5
252567_at	At3g46070	Zinc finger; C2H2 type family protein	0.0072	0.0186	-14.9
253187_at	At4g35280	Zinc finger; C2H2 type family protein	0.0360	0.0419	-5.5
249370_at	At5g40710	Zinc finger; C2H2 type family protein	0.0117	0.0237	-4.7
250396_at	At5g10970	Zinc finger; C2H2 type family protein	0.0110	0.0230	-4.3
265081_at	At1g03840	Zinc finger; C2H2 type family protein	0.0109	0.0229	-3.6
247655_at	At5g59820	Zinc finger; C2H2 type family protein; ZAT12	0.0310	0.0387	-3.3
251634_at	At3g57480	Zinc finger; C2H2 type; AN1-like family protein	0.0244	0.0343	-3.3
256356_s_at	At1g66500	Zinc finger; C2H2-type family protein	0.0020	0.0104	-5.8
252704_at	At3g43750	Zinc finger; C3HC4-type RING finger family protein	0.0001	0.0031	-23.2
261265_at	At1g26800	Zinc finger; C3HC4-type RING finger family protein	0.0407	0.0448	-13.7
267417_at	At2g34990	Zinc finger; C3HC4-type RING finger family protein	0.0338	0.0405	-11.0
257656_at	At3g13228	Zinc finger; C3HC4-type RING finger family protein	0.0239	0.0340	-6.7
265852_at	At2g42350	Zinc finger; C3HC4-type RING finger family protein	0.0002	0.0032	-6.0
258261_at	At3g15740	Zinc finger; C3HC4-type RING finger family protein	0.0021	0.0105	-5.4
247595_at	At5g60820	Zinc finger; C3HC4-type RING finger family protein	0.0002	0.0038	-4.6
261481_at	At1g14260	Zinc finger; C3HC4-type RING finger family protein	0.0001	0.0020	-4.3
252474_at	At3g46620	Zinc finger; C3HC4-type RING finger family protein	0.0073	0.0187	-4.0
262167_at	At1g74990	Zinc finger; C3HC4-type RING finger family protein	0.0391	0.0438	-3.7
255511_at	At4g02075	Zinc finger; C3HC4-type RING finger family protein	0.0011	0.0078	-3.3
260008_at	At1g68070	Zinc finger; C3HC4-type RING finger family protein	0.0072	0.0187	-3.0
249862_at	At5g22920	Zinc finger; C3HC4-type RING finger family protein	0.0032	0.0131	-2.9
262252_at	At1g53820	Zinc finger; C3HC4-type RING finger family protein	0.0208	0.0316	-2.8
265853_at	At2g42360	Zinc finger; C3HC4-type RING finger family protein	0.0042	0.0147	-2.7
245329_at	At4g14365	Zinc finger; C3HC4-type RING finger family protein	0.0441	0.0469	-3.0
258529_at	At3g06740	Zinc finger; GATA type family protein	0.0467	0.0482	-3.1
258630_at	At3g02820	Zinc knuckle; CCHC-type family protein	0.0062	0.0173	-3.5
I1. Helicase activity					
262553_at	At1g31360	Helicase, putative; RECQl2	0.0443	0.0470	-4.2
I2. RNA metabolism					
249976_at	At5g18810	SC35-like splicing factor; 28 kD;SCL28	0.0057	0.0169	-4.0
262927_at	At1g65810	tRNA-splicing endonuclease positive effector-related	0.0174	0.0288	-4.7
J. Transporters					
256672_at	At3g52310	ABC transporter family protein	0.0198	0.0308	-18.2
255855_at	At1g66950	ABC transporter family protein	0.0006	0.0063	-11.3
247613_at	At5g60740	ABC transporter family protein	0.0263	0.0356	-6.1
266107_at	At2g37910	Cation/hydrogen exchanger, putative; CHX21	0.0085	0.0201	-3.0
249883_at	At5g22900	Cation/hydrogen exchanger, putative; CHX3	0.0005	0.0058	-4.7
260623_at	At1g08090	High-affinity nitrate transporter; ACH1	0.0478	0.0488	-5.1
250487_at	At5g09690	Magnesium transporter CorA-like family protein; MRS2-7	0.0000	0.0008	-11.5
247207_at	At5g65000	Nucleotide-sugar transporter family protein	0.0038	0.0140	-3.7
251752_at	At3g55740	Proline transporter 2; ProT2	0.0472	0.0485	-3.5
254083_at	At4g24920	Protein transport protein SEC61 gamma subunit, putative	0.0000	0.0015	-7.7
252328_at	At3g48570	Protein transport protein SEC61 gamma subunit, putative	0.0210	0.0317	-3.5
260400_at	At1g69860	Proton-dependent oligopeptide transport; POT family protein	0.0057	0.0168	-13.7
252538_at	At3g45720	Proton-dependent oligopeptide transport; POT family protein	0.0485	0.0493	-9.1
252537_at	At3g45710	Proton-dependent oligopeptide transport; POT family protein	0.0407	0.0448	-4.2
250378_at	At5g11570	Proton-dependent oligopeptide transport; POT family protein	0.0356	0.0416	-3.1
247415_at	At5g63060	SEC14 cytosolic factor, putative	0.0010	0.0076	-3.3
246887_at	At5g26250	Sugar transporter, putative	0.0349	0.0412	-7.7
248127_at	At5g54750	Transport protein particle;TRAPP component Bet3, putative	0.0455	0.0477	-2.7
264373_at	At1g11890	Vesicle transport protein SEC22, putative	0.0246	0.0345	-2.9
K. Transposable elements, viral and plasmid proteins					
265755_at	At2g10630	CACTA-like transposase family;En/Spm	0.0325	0.0397	-5.7
263051_s_at	At2g04210	CACTA-like transposase family;Ptta/En/Spm	0.0018	0.0098	-6.3
246648_at	At5g35140	CACTA-like transposase family;Ptta/En/Spm	0.0193	0.0303	-5.6

265754_x_at	At2g10640	CACTA-like transposase family;Ptta/En/Spm	0.0038	0.0142	-3.0
255313_at	At4g04590	CACTA-like transposase family;Tnp2/En/Spm	0.0141	0.0261	-11.2
262008_s_at	At1g35590	CACTA-like transposase family;Tnp2/En/Spm	0.0057	0.0168	-7.5
265890_s_at	At2g14980	CACTA-like transposase family;Tnp2/En/Spm	0.0196	0.0306	-7.5
259459_at	At1g44070	CACTA-like transposase family;Tnp2/En/Spm	0.0428	0.0460	-6.2
254947_at	At4g10990	Copia-like retrotransposon family	0.0202	0.0311	-18.6
265241_at	At2g07693	Copia-like retrotransposon family	0.0051	0.0160	-12.1
261184_at	At1g34545	Copia-like retrotransposon family	0.0220	0.0324	-8.2
255323_at	At4g04280	Copia-like retrotransposon family	0.0159	0.0277	-6.8
255463_at	At4g02960	Copia-like retrotransposon family	0.0476	0.0487	-5.1
265971_at	At2g11220	Copia-like retrotransposon family	0.0296	0.0379	-4.9
261997_at	At1g33817	Copia-like retrotransposon family	0.0212	0.0319	-4.8
256032_at	At1g34090	Copia-like retrotransposon family	0.0001	0.0023	-4.6
257347_at	At3g29792	Copia-like retrotransposon family	0.0078	0.0193	-3.7
254973_at	At4g10460	Copia-like retrotransposon family	0.0164	0.0280	-3.6
265299_at	At2g13940	Copia-like retrotransposon family	0.0324	0.0396	-3.1
263746_at	At2g21460	Copia-like retrotransposon family	0.0052	0.0161	-2.9
246604_at	At5g35310	Copia-like retrotransposon family	0.0190	0.0301	-2.8
264074_at	At2g10780	Gypsy-like retrotransposon family	0.0154	0.0273	-15.5
263965_at	At2g12870	Gypsy-like retrotransposon family	0.0096	0.0214	-13.2
265525_at	At2g06170	Gypsy-like retrotransposon family	0.0310	0.0387	-8.5
265361_at	At2g13230	Gypsy-like retrotransposon family	0.0195	0.0305	-7.4
255209_at	At4g07590	Gypsy-like retrotransposon family	0.0475	0.0487	-4.4
265565_at	At2g05610	Gypsy-like retrotransposon family	0.0019	0.0102	-2.7
263991_at	At2g13020	Gypsy-like retrotransposon family; Athila	0.0002	0.0035	-20.9
255156_at	At4g07780	Gypsy-like retrotransposon family; Athila	0.0313	0.0388	-11.3
252794_at	At3g42260	Gypsy-like retrotransposon family; Athila	0.0064	0.0177	-4.1
263187_at	At1g36130	Gypsy-like retrotransposon family; Athila	0.0396	0.0441	-3.7
265303_at	At2g14040	Gypsy-like retrotransposon family; Athila	0.0015	0.0090	-2.8
252725_at	At3g43050	Mutator-like transposase family	0.0006	0.0060	-29.6
251435_at	At3g59860	Mutator-like transposase family	0.0012	0.0082	-7.9
261730_s_at	At1g47816	Mutator-like transposase family	0.0029	0.0125	-7.8
255058_s_at	At4g09410	Mutator-like transposase family	0.0415	0.0452	-3.8
263770_at	At2g06410	Mutator-like transposase family	0.0360	0.0419	-3.5
255154_at	At4g08220	Mutator-like transposase family	0.0012	0.0081	-3.4
255476_at	At4g02490	Non-LTR retrotransposon family; LINE	0.0139	0.0259	-5.0
246678_at	At5g33200	PREDICTED REPLICATION A1 PROTEINS	0.0014	0.0088	-9.7
265695_at	At2g24490	Replication protein, putative	0.0052	0.0161	-2.6
249654_at	At5g37090	Replication protein-related	0.0000	0.0006	-22.4
255188_at	At4g07340	Replication protein-related	0.0339	0.0405	-13.7
266116_at	At2g02180	Tobamovirus multiplication protein 3; TOM3	0.0107	0.0226	-3.2
L. Unknown proteins					
250152_at	At5g15120	Expressed protein	0.0001	0.0028	-31.1
249896_at	At5g22530	Expressed protein	0.0021	0.0105	-28.6
258391_at	At3g15420	Expressed protein	0.0123	0.0245	-27.2
250881_at	At5g04080	Expressed protein	0.0003	0.0042	-26.9
260668_at	At1g19530	Expressed protein	0.0079	0.0195	-26.9
266502_at	At2g47720	Expressed protein	0.0027	0.0120	-23.2
260389_at	At1g74055	Expressed protein	0.0176	0.0290	-20.4
259120_at	At3g02240	Expressed protein	0.0289	0.0374	-19.5
247375_at	At5g63135	Expressed protein	0.0003	0.0041	-17.8
256135_at	At1g48730	Expressed protein	0.0002	0.0035	-17.6
265327_at	At2g18210	Expressed protein	0.0042	0.0147	-16.8
257724_at	At3g18510	Expressed protein	0.0005	0.0057	-16.3
252946_at	At4g39235	Expressed protein	0.0144	0.0264	-16.0
257402_at	At1g23570	Expressed protein	0.0125	0.0246	-14.4
251345_at	At3g60940	Expressed protein	0.0040	0.0145	-14.0
248774_at	At5g47830	Expressed protein	0.0399	0.0442	-13.8
263102_at	At2g05270	Expressed protein	0.0335	0.0402	-13.8
263182_at	At1g05575	Expressed protein	0.0015	0.0092	-13.2
256283_at	At3g12540	Expressed protein	0.0035	0.0135	-12.9
264958_at	At1g76960	Expressed protein	0.0431	0.0462	-12.5
252218_at	At3g50150	Expressed protein	0.0322	0.0394	-12.5
257542_at	At3g26050	Expressed protein	0.0097	0.0215	-12.1
265518_at	At2g06040	Expressed protein	0.0143	0.0263	-12.0
247146_at	At5g65610	Expressed protein	0.0040	0.0145	-11.7
252882_at	At4g39675	Expressed protein	0.0005	0.0057	-11.3

251114_at	At5g01380	Expressed protein	0.0100	0.0218	-11.2
246270_at	At4g36500	Expressed protein	0.0103	0.0222	-11.2
265164_at	At1g23600	Expressed protein	0.0024	0.0114	-11.2
267332_at	At2g19290	Expressed protein	0.0009	0.0072	-11.0
245906_at	At5g11070	Expressed protein	0.0008	0.0070	-10.8
262602_at	At1g15270	Expressed protein	0.0020	0.0103	-10.8
249756_at	At5g24313	Expressed protein	0.0263	0.0356	-10.3
248509_at	At5g50335	Expressed protein	0.0479	0.0489	-10.2
247431_at	At5g62520	Expressed protein	0.0118	0.0238	-10.2
257716_at	At3g18300	Expressed protein	0.0000	0.0001	-10.0
255955_at	At1g22030	Expressed protein	0.0489	0.0494	-10.0
261461_at	At1g07860	Expressed protein	0.0037	0.0138	-9.9
267133_at	At2g23440	Expressed protein	0.0006	0.0062	-9.7
267321_at	At2g19320	Expressed protein	0.0417	0.0453	-9.5
249861_at	At5g22875	Expressed protein	0.0366	0.0422	-9.2
253117_at	At4g35905	Expressed protein	0.0105	0.0224	-8.8
253168_at	At4g35070	Expressed protein	0.0468	0.0483	-8.8
265231_s_at	At2g07713	Expressed protein	0.0220	0.0324	-8.6
252524_at	At3g46430	Expressed protein	0.0021	0.0106	-8.4
260038_at	At1g68875	Expressed protein	0.0284	0.0371	-8.3
260744_at	At1g15010	Expressed protein	0.0189	0.0301	-8.3
258370_at	At3g14395	Expressed protein	0.0091	0.0208	-8.3
257134_at	At3g12870	Expressed protein	0.0190	0.0301	-8.2
264488_s_at	At1g27330	Expressed protein	0.0003	0.0047	-8.1
250076_at	At5g16660	Expressed protein	0.0009	0.0073	-8.0
247070_at	At5g66815	Expressed protein	0.0028	0.0123	-7.9
248430_at	At5g51800	Expressed protein	0.0308	0.0386	-7.9
263674_at	At2g04790	Expressed protein	0.0020	0.0105	-7.8
263524_x_at	At2g24920	Expressed protein	0.0278	0.0367	-7.8
249288_at	At5g41050	Expressed protein	0.0399	0.0442	-7.7
263369_at	At2g20480	Expressed protein	0.0456	0.0478	-7.6
253086_at	At4g36370	Expressed protein	0.0124	0.0245	-7.6
257579_at	At3g11000	Expressed protein	0.0485	0.0493	-7.5
256159_at	At1g30135	Expressed protein	0.0054	0.0164	-7.4
266472_at	At2g31150	Expressed protein	0.0487	0.0494	-7.4
267345_at	At2g44240	Expressed protein	0.0003	0.0043	-7.3
260437_at	At1g68380	Expressed protein	0.0302	0.0382	-7.3
259479_at	At1g19020	Expressed protein	0.0016	0.0093	-7.2
265820_at	At2g17940	Expressed protein	0.0497	0.0499	-7.0
255960_at	At1g22140	Expressed protein	0.0040	0.0145	-6.9
250882_at	At5g04000	Expressed protein	0.0363	0.0421	-6.9
256499_at	At1g36640	Expressed protein	0.0061	0.0173	-6.9
256796_at	At3g22210	Expressed protein	0.0000	0.0004	-6.8
247922_at	At5g57500	Expressed protein	0.0095	0.0213	-6.8
249943_at	At5g22280	Expressed protein	0.0068	0.0181	-6.7
259325_at	At3g05320	Expressed protein	0.0004	0.0048	-6.4
264297_at	At1g78710	Expressed protein	0.0121	0.0242	-6.3
255978_at	At1g34010	Expressed protein	0.0006	0.0061	-6.2
257540_at	At3g21520	Expressed protein	0.0458	0.0479	-6.1
255243_at	At4g05590	Expressed protein	0.0040	0.0145	-6.1
263812_at	At2g09960	Expressed protein	0.0252	0.0349	-6.1
257793_at	At3g26960	Expressed protein	0.0154	0.0273	-6.1
254257_s_at	At4g23350	Expressed protein	0.0182	0.0295	-6.0
255285_at	At4g04630	Expressed protein	0.0243	0.0342	-5.9
265837_at	At2g14560	Expressed protein	0.0000	0.0017	-5.9
245333_at	At4g14615	Expressed protein	0.0023	0.0110	-5.8
263396_at	At2g31710	Expressed protein	0.0009	0.0071	-5.8
249379_at	At5g40460	Expressed protein	0.0116	0.0236	-5.8
254037_at	At4g25760	Expressed protein	0.0207	0.0315	-5.7
260895_at	At1g29250	Expressed protein	0.0059	0.0170	-5.7
260012_at	At1g67865	Expressed protein	0.0009	0.0073	-5.7
267261_at	At2g23120	Expressed protein	0.0020	0.0104	-5.6
247143_at	At5g65580	Expressed protein	0.0150	0.0270	-5.6
265025_at	At1g24575	Expressed protein	0.0356	0.0416	-5.6
260656_at	At1g19380	Expressed protein	0.0064	0.0177	-5.6
257609_at	At3g13845	Expressed protein	0.0073	0.0188	-5.6
252908_at	At4g39670	Expressed protein	0.0103	0.0222	-5.6
257956_at	At3g25400	Expressed protein	0.0015	0.0091	-5.5

253044_at	At4g37290	Expressed protein	0.0007	0.0068	-5.5
252419_at	At3g47510	Expressed protein	0.0113	0.0233	-5.5
256047_at	At1g07060	Expressed protein	0.0285	0.0372	-5.5
258262_at	At3g15770	Expressed protein	0.0001	0.0022	-5.5
253917_at	At4g27380	Expressed protein	0.0467	0.0483	-5.4
249021_at	At5g44820	Expressed protein	0.0003	0.0042	-5.3
252165_at	At3g50550	Expressed protein	0.0002	0.0034	-5.3
246018_at	At5g10695	Expressed protein	0.0231	0.0334	-5.3
264933_at	At1g61160	Expressed protein	0.0423	0.0457	-5.2
262853_at	At1g20890	Expressed protein	0.0348	0.0411	-5.2
260946_at	At1g06010	Expressed protein	0.0247	0.0345	-5.2
249578_at	At5g37730	Expressed protein	0.0365	0.0422	-5.2
252612_at	At3g45160	Expressed protein	0.0161	0.0279	-5.1
259675_at	At1g77710	Expressed protein	0.0041	0.0146	-5.1
260754_at	At1g49000	Expressed protein	0.0025	0.0117	-5.0
258362_at	At3g14280	Expressed protein	0.0168	0.0283	-5.0
259979_at	At1g76600	Expressed protein	0.0323	0.0395	-5.0
262236_at	At1g48330	Expressed protein	0.0335	0.0402	-5.0
260005_at	At1g67920	Expressed protein	0.0010	0.0076	-5.0
259271_at	At3g01170	Expressed protein	0.0006	0.0061	-5.0
249941_at	At5g22270	Expressed protein	0.0008	0.0070	-5.0
264830_at	At1g03710	Expressed protein	0.0185	0.0298	-4.9
262373_at	At1g73120	Expressed protein	0.0389	0.0437	-4.9
245840_at	At1g58420	Expressed protein	0.0170	0.0285	-4.9
247305_at	At5g63905	Expressed protein	0.0009	0.0073	-4.9
248133_at	At5g54850	Expressed protein	0.0064	0.0177	-4.9
262832_s_at	At1g14870	Expressed protein	0.0077	0.0193	-4.9
266901_at	At2g34600	Expressed protein	0.0209	0.0316	-4.8
259497_at	At1g15860	Expressed protein	0.0062	0.0174	-4.8
262286_at	At1g68585	Expressed protein	0.0299	0.0380	-4.8
259889_at	At1g76405	Expressed protein	0.0068	0.0181	-4.8
250301_at	At5g11970	Expressed protein	0.0046	0.0152	-4.8
259894_at	At1g71430	Expressed protein	0.0349	0.0412	-4.7
259841_at	At1g52200	Expressed protein	0.0012	0.0082	-4.7
263799_at	At2g24550	Expressed protein	0.0016	0.0093	-4.7
265569_at	At2g05620	Expressed protein	0.0024	0.0112	-4.7
255745_at	At1g32030	Expressed protein	0.0242	0.0341	-4.7
264635_at	At1g65500	Expressed protein	0.0305	0.0384	-4.6
255954_at	At1g22090	Expressed protein	0.0178	0.0292	-4.6
259847_at	At1g72170	Expressed protein	0.0060	0.0172	-4.6
247930_at	At5g57060	Expressed protein	0.0202	0.0311	-4.6
253045_at	At4g37445	Expressed protein	0.0486	0.0494	-4.6
247725_at	At5g59410	Expressed protein	0.0124	0.0245	-4.5
255809_at	At4g10300	Expressed protein	0.0027	0.0119	-4.5
257707_at	At3g12650	Expressed protein	0.0123	0.0244	-4.5
258058_at	At3g28980	Expressed protein	0.0477	0.0487	-4.5
247610_at	At5g60630	Expressed protein	0.0119	0.0240	-4.5
253670_at	At4g30010	Expressed protein	0.0026	0.0118	-4.5
259766_at	At1g64360	Expressed protein	0.0103	0.0222	-4.4
265184_at	At1g23710	Expressed protein	0.0172	0.0286	-4.4
255028_at	At4g09890	Expressed protein	0.0003	0.0040	-4.4
249889_at	At5g22540	Expressed protein	0.0263	0.0356	-4.4
253292_at	At4g33985	Expressed protein	0.0419	0.0454	-4.3
261535_at	At1g01725	Expressed protein	0.0165	0.0282	-4.3
250289_at	At5g13190	Expressed protein	0.0070	0.0183	-4.3
267239_at	At2g02510	Expressed protein	0.0035	0.0135	-4.3
263638_at	At2g25310	Expressed protein	0.0051	0.0160	-4.3
256506_at	At1g75160	Expressed protein	0.0278	0.0367	-4.3
263981_at	At2g42870	Expressed protein	0.0380	0.0431	-4.3
267259_at	At2g23090	Expressed protein	0.0101	0.0220	-4.3
257477_at	At1g10660	Expressed protein	0.0263	0.0356	-4.3
263370_at	At2g20500	Expressed protein	0.0184	0.0297	-4.3
260651_at	At1g32460	Expressed protein	0.0114	0.0234	-4.3
245642_at	At1g25275	Expressed protein	0.0013	0.0085	-4.2
267225_at	At2g44000	Expressed protein	0.0386	0.0435	-4.2
265628_at	At2g27290	Expressed protein	0.0034	0.0133	-4.2
265571_s_at	At2g28230	Expressed protein	0.0006	0.0060	-4.1
251650_at	At3g57360	Expressed protein	0.0189	0.0301	-4.1

248889_at	At5g46230	Expressed protein	0.0076	0.0192	-4.1
254779_at	At4g12760	Expressed protein	0.0000	0.0005	-4.1
254751_at	At4g13150	Expressed protein	0.0021	0.0106	-4.1
266916_at	At2g45860	Expressed protein	0.0114	0.0234	-4.1
261297_at	At1g48500	Expressed protein	0.0017	0.0096	-4.1
249107_at	At5g43680	Expressed protein	0.0151	0.0271	-4.1
258793_at	At3g04780	Expressed protein	0.0326	0.0397	-4.0
258201_at	At3g13910	Expressed protein	0.0006	0.0058	-4.0
261304_at	At1g48440	Expressed protein	0.0000	0.0017	-4.0
267383_at	At2g44360	Expressed protein	0.0299	0.0380	-4.0
252073_at	At3g51750	Expressed protein	0.0196	0.0306	-4.0
252412_at	At3g47295	Expressed protein	0.0422	0.0456	-4.0
265481_at	At2g15960	Expressed protein	0.0006	0.0063	-4.0
253762_at	At4g28830	Expressed protein	0.0095	0.0213	-4.0
250796_at	At5g05300	Expressed protein	0.0039	0.0143	-3.9
258468_at	At3g06070	Expressed protein	0.0004	0.0053	-3.9
249297_at	At5g41320	Expressed protein	0.0450	0.0474	-3.9
256934_at	At3g22530	Expressed protein	0.0051	0.0160	-3.9
258049_at	At3g16220	Expressed protein	0.0012	0.0083	-3.9
250511_at	At5g09960	Expressed protein	0.0162	0.0280	-3.9
248415_at	At5g51620	Expressed protein	0.0082	0.0199	-3.9
263023_at	At1g23960	Expressed protein	0.0000	0.0008	-3.9
262868_at	At1g64980	Expressed protein	0.0028	0.0122	-3.9
248186_at	At5g53880	Expressed protein	0.0014	0.0089	-3.9
246479_at	At5g16060	Expressed protein	0.0002	0.0040	-3.9
258413_at	At3g17300	Expressed protein	0.0158	0.0276	-3.9
250018_at	At5g18150	Expressed protein	0.0468	0.0483	-3.9
258935_at	At3g10120	Expressed protein	0.0138	0.0258	-3.8
246631_at	At1g50740	Expressed protein	0.0063	0.0175	-3.8
261987_at	At1g33710	Expressed protein	0.0260	0.0354	-3.8
249988_at	At5g18310	Expressed protein	0.0087	0.0203	-3.8
266521_at	At2g24020	Expressed protein	0.0000	0.0008	-3.7
264738_at	At1g62250	Expressed protein	0.0016	0.0095	-3.7
257421_at	At1g12030	Expressed protein	0.0343	0.0408	-3.7
261940_at	At1g22520	Expressed protein	0.0018	0.0100	-3.7
251036_at	At5g02160	Expressed protein	0.0195	0.0305	-3.7
263410_at	At2g04039	Expressed protein	0.0202	0.0311	-3.6
261802_at	At1g30550	Expressed protein	0.0304	0.0383	-3.6
264164_at	At1g65295	Expressed protein	0.0008	0.0070	-3.6
249778_at	At5g24165	Expressed protein	0.0068	0.0181	-3.6
246539_at	At5g15460	Expressed protein	0.0054	0.0164	-3.6
263785_at	At2g46390	Expressed protein	0.0219	0.0323	-3.6
254158_at	At4g24380	Expressed protein	0.0081	0.0197	-3.6
255648_at	At4g00910	Expressed protein	0.0345	0.0410	-3.6
245491_at	At4g16320	Expressed protein	0.0442	0.0470	-3.6
249076_at	At5g43970	Expressed protein	0.0007	0.0065	-3.5
264879_at	At1g61260	Expressed protein	0.0078	0.0193	-3.5
247800_at	At5g58570	Expressed protein	0.0068	0.0181	-3.5
254161_at	At4g24370	Expressed protein	0.0010	0.0076	-3.5
248028_at	At5g55620	Expressed protein	0.0434	0.0464	-3.5
266811_at	At2g44850	Expressed protein	0.0003	0.0040	-3.5
264241_at	At1g54840	Expressed protein	0.0239	0.0339	-3.5
255810_at	At4g10140	Expressed protein	0.0407	0.0448	-3.5
246630_at	At1g50730	Expressed protein	0.0037	0.0140	-3.5
253827_at	At4g28085	Expressed protein	0.0145	0.0264	-3.4
267575_at	At2g30690	Expressed protein	0.0090	0.0208	-3.4
266806_at	At2g30000	Expressed protein	0.0001	0.0021	-3.4
252661_at	At3g44450	Expressed protein	0.0067	0.0180	-3.4
260200_at	At1g67620	Expressed protein	0.0139	0.0259	-3.4
254190_at	At4g23885	Expressed protein	0.0093	0.0210	-3.4
267108_at	At2g14830	Expressed protein	0.0381	0.0432	-3.4
263710_at	At1g09330	Expressed protein	0.0083	0.0200	-3.4
261422_at	At1g18730	Expressed protein	0.0000	0.0008	-3.4
255733_at	At1g25400	Expressed protein	0.0092	0.0209	-3.4
258244_at	At3g27770	Expressed protein	0.0316	0.0391	-3.4
256456_at	At1g75180	Expressed protein	0.0353	0.0414	-3.4
249332_at	At5g40980	Expressed protein	0.0080	0.0196	-3.4
253537_at	At4g31560	Expressed protein	0.0015	0.0090	-3.3

249358_at	At5g40510	Expressed protein	0.0036	0.0138	-3.3
254629_at	At4g18425	Expressed protein	0.0153	0.0272	-3.3
249752_at	At5g24660	Expressed protein	0.0042	0.0147	-3.3
257953_at	At3g21865	Expressed protein	0.0118	0.0238	-3.3
253325_at	At4g33925	Expressed protein	0.0397	0.0441	-3.3
250225_at	At5g14105	Expressed protein	0.0175	0.0289	-3.3
249522_at	At5g38700	Expressed protein	0.0113	0.0233	-3.3
257074_at	At3g19660	Expressed protein	0.0134	0.0255	-3.3
249555_at	At5g38300	Expressed protein	0.0318	0.0392	-3.3
251241_s_at	At3g62460	Expressed protein	0.0029	0.0123	-3.3
262000_at	At1g33810	Expressed protein	0.0145	0.0264	-3.3
263772_at	At2g06255	Expressed protein	0.0063	0.0175	-3.3
263305_at	At2g01930	Expressed protein	0.0091	0.0208	-3.3
266101_at	At2g37940	Expressed protein	0.0121	0.0242	-3.3
247083_at	At5g66290	Expressed protein	0.0355	0.0415	-3.2
256337_at	At1g72060	Expressed protein	0.0324	0.0396	-3.2
258393_at	At3g15480	Expressed protein	0.0147	0.0267	-3.2
258681_at	At3g08610	Expressed protein	0.0145	0.0264	-3.2
260174_at	At1g71940	Expressed protein	0.0296	0.0379	-3.2
258057_at	At3g28970	Expressed protein	0.0159	0.0277	-3.2
254626_at	At4g18400	Expressed protein	0.0091	0.0208	-3.2
263178_at	At1g05550	Expressed protein	0.0162	0.0280	-3.2
247293_at	At5g64510	Expressed protein	0.0002	0.0037	-3.2
250689_at	At5g06610	Expressed protein	0.0346	0.0410	-3.2
249918_at	At5g19240	Expressed protein	0.0113	0.0233	-3.2
259909_at	At1g60870	Expressed protein	0.0009	0.0072	-3.1
264735_s_at	At1g62060	Expressed protein	0.0409	0.0449	-3.1
247072_at	At5g66490	Expressed protein	0.0163	0.0280	-3.1
246931_at	At5g25170	Expressed protein	0.0042	0.0147	-3.1
251655_at	At3g57160	Expressed protein	0.0001	0.0028	-3.1
249377_at	At5g40690	Expressed protein	0.0126	0.0247	-3.1
266039_s_at	At2g07739	Expressed protein	0.0248	0.0345	-3.1
245755_at	At1g35210	Expressed protein	0.0457	0.0478	-3.0
264579_at	At1g05205	Expressed protein	0.0003	0.0042	-3.0
260555_at	At2g41780	Expressed protein	0.0168	0.0283	-3.0
263673_at	At2g04800	Expressed protein	0.0029	0.0125	-3.0
247177_at	At5g65300	Expressed protein	0.0011	0.0079	-3.0
246584_at	At5g14730	Expressed protein	0.0002	0.0035	-3.0
248196_at	At5g54150	Expressed protein	0.0371	0.0425	-3.0
265849_at	At2g35736	Expressed protein	0.0231	0.0334	-3.0
249285_at	At5g41960	Expressed protein	0.0018	0.0098	-3.0
255808_at	At4g10280	Expressed protein	0.0204	0.0313	-3.0
248302_at	At5g53160	Expressed protein	0.0244	0.0343	-3.0
265674_at	At2g32190	Expressed protein	0.0006	0.0060	-3.0
261748_at	At1g76070	Expressed protein	0.0064	0.0177	-3.0
258091_at	At3g14560	Expressed protein	0.0014	0.0089	-2.9
246008_at	At5g08320	Expressed protein	0.0167	0.0283	-2.9
257800_at	At3g15900	Expressed protein	0.0310	0.0387	-2.9
253119_at	At4g35980	Expressed protein	0.0031	0.0128	-2.9
254930_at	At4g11450	Expressed protein	0.0036	0.0137	-2.9
249313_at	At5g41560	Expressed protein	0.0123	0.0245	-2.9
266801_at	At2g22870	Expressed protein	0.0256	0.0352	-2.9
265386_at	At2g20930	Expressed protein	0.0092	0.0209	-2.9
246708_at	At5g28150	Expressed protein	0.0229	0.0332	-2.9
260211_at	At1g74440	Expressed protein	0.0317	0.0391	-2.9
266094_at	At2g37975	Expressed protein	0.0181	0.0295	-2.9
267370_at	At2g44525	Expressed protein	0.0329	0.0399	-2.8
252676_at	At3g44280	Expressed protein	0.0171	0.0286	-2.8
265767_at	At2g48110	Expressed protein	0.0128	0.0249	-2.8
266334_at	At2g32380	Expressed protein	0.0284	0.0371	-2.8
267623_at	At2g39650	Expressed protein	0.0092	0.0209	-2.8
252541_at	At3g45750	Expressed protein	0.0089	0.0206	-2.8
253694_at	At4g29735	Expressed protein	0.0167	0.0282	-2.8
254240_at	At4g23496	Expressed protein	0.0017	0.0097	-2.8
253870_at	At4g27530	Expressed protein	0.0428	0.0460	-2.8
262217_at	At1g74770	Expressed protein	0.0167	0.0282	-2.8
262481_at	At1g17080	Expressed protein	0.0330	0.0399	-2.8
266556_at	At2g46230	Expressed protein	0.0485	0.0493	-2.8

255672_at	At4g00310	Expressed protein	0.0410	0.0449	-2.8
260072_at	At1g73650	Expressed protein	0.0057	0.0169	-2.7
257055_at	At3g15351	Expressed protein	0.0166	0.0282	-2.7
252603_at	At3g45050	Expressed protein	0.0038	0.0141	-2.7
257710_at	At3g27350	Expressed protein	0.0132	0.0253	-2.7
255331_at	At4g04330	Expressed protein	0.0019	0.0101	-2.7
253691_at	At4g29660	Expressed protein	0.0252	0.0349	-2.7
249519_at	At5g38660	Expressed protein	0.0076	0.0191	-2.7
262287_at	At1g68660	Expressed protein	0.0315	0.0390	-2.7
262622_at	At1g06510	Expressed protein	0.0043	0.0148	-2.7
256766_at	At3g22231	Expressed protein	0.0196	0.0306	-2.7
262199_at	At1g53800	Expressed protein	0.0103	0.0222	-2.7
257951_at	At3g21700	Expressed protein	0.0009	0.0074	-2.7
263442_at	At2g28605	Expressed protein	0.0029	0.0124	-2.6
265459_at	At2g46540	Expressed protein	0.0096	0.0214	-2.6
263737_at	At1g60010	Expressed protein	0.0355	0.0415	-2.6
262801_at	At1g21010	Expressed protein	0.0141	0.0261	-2.6
257780_at	At3g27100	Expressed protein	0.0112	0.0232	-2.6
262399_at	At1g49500	Expressed protein	0.0276	0.0365	-2.6
247213_at	At5g64900	Expressed protein	0.0005	0.0058	-2.6
246020_at	At5g10710	Expressed protein	0.0216	0.0322	-2.6
260916_at	At1g02475	Expressed protein	0.0409	0.0449	-2.6
260004_at	At1g67860	Expressed protein	0.0000	0.0006	-2.6
261038_at	At1g17490	Expressed protein	0.0352	0.0414	-2.6
261951_at	At1g64490	Expressed protein	0.0280	0.0368	-2.6
254429_at	At4g21105	Expressed protein	0.0334	0.0401	-2.6
246010_at	At5g08440	Expressed protein	0.0317	0.0391	-2.6
252789_s_at	At3g42150	Expressed protein	0.0303	0.0382	-2.6
251727_at	At3g56290	Expressed protein	0.0009	0.0073	-2.6
248401_at	At5g52110	Expressed protein	0.0192	0.0302	-2.6
254557_at	At4g19140	Expressed protein	0.0268	0.0360	-2.5
248044_at	At5g56020	Expressed protein	0.0174	0.0289	-2.5
265226_at	At2g28430	Expressed protein	0.0494	0.0497	-2.5
247258_at	At5g64816	Expressed protein	0.0043	0.0148	-2.5
248819_at	At5g47050	Expressed protein	0.0058	0.0169	-2.5
249230_at	At5g42070	Expressed protein	0.0006	0.0060	-2.5
247399_at	At5g62960	Expressed protein	0.0454	0.0477	-2.5
249727_at	At5g35490	Expressed protein	0.0184	0.0297	-4.6
261980_at	At1g33820	Hypothetical protein	0.0035	0.0135	-48.2
265293_at	At2g14020	Hypothetical protein	0.0001	0.0027	-47.1
257133_at	At3g17190	Hypothetical protein	0.0000	0.0006	-32.0
256642_at	At3g32280	Hypothetical protein	0.0026	0.0118	-30.9
248617_at	At5g49590	Hypothetical protein	0.0082	0.0199	-19.2
247913_at	At5g57510	Hypothetical protein	0.0026	0.0118	-18.5
261778_at	At1g76220	Hypothetical protein	0.0109	0.0229	-18.0
257575_at	At3g29265	Hypothetical protein	0.0000	0.0007	-17.1
256668_at	At3g32190	Hypothetical protein	0.0071	0.0185	-16.9
257561_at	At3g24270	Hypothetical protein	0.0170	0.0285	-16.7
257839_at	At3g25080	Hypothetical protein	0.0105	0.0225	-14.9
261706_at	At1g32680	Hypothetical protein	0.0040	0.0145	-14.3
246873_at	At5g26100	Hypothetical protein	0.0061	0.0173	-14.0
257070_at	At3g28190	Hypothetical protein	0.0009	0.0073	-13.9
262792_at	At1g10710	Hypothetical protein	0.0155	0.0274	-13.3
254302_at	At4g22800	Hypothetical protein	0.0441	0.0469	-12.7
245648_at	At1g24938	Hypothetical protein	0.0371	0.0425	-12.3
256925_at	At3g29600	Hypothetical protein	0.0441	0.0469	-12.2
256718_at	At2g34120	Hypothetical protein	0.0111	0.0231	-12.2
257118_at	At3g20180	Hypothetical protein	0.0042	0.0146	-11.9
261760_at	At1g15600	Hypothetical protein	0.0077	0.0193	-11.5
255744_at	At1g32040	Hypothetical protein	0.0458	0.0479	-11.5
267424_at	At2g34800	Hypothetical protein	0.0068	0.0181	-10.8
263308_at	At2g10380	Hypothetical protein	0.0100	0.0218	-10.7
249531_at	At5g38770	Hypothetical protein	0.0152	0.0272	-10.7
263309_at	At2g10370	Hypothetical protein	0.0217	0.0323	-10.1
259621_at	At1g42710	Hypothetical protein	0.0294	0.0377	-9.8
255092_s_at	At4g09400	Hypothetical protein	0.0001	0.0028	-9.4
262719_at	At1g43590	Hypothetical protein	0.0312	0.0388	-9.3
248776_at	At5g47900	Hypothetical protein	0.0159	0.0277	-9.3

259772_at	At1g29480	Hypothetical protein	0.0400	0.0443	-8.7
256687_x_at	At3g32120	Hypothetical protein	0.0058	0.0169	-7.9
259779_s_at	At1g29620	Hypothetical protein	0.0174	0.0288	-7.6
259389_at	At1g06320	Hypothetical protein	0.0154	0.0273	-7.6
255246_at	At4g05640	Hypothetical protein	0.0058	0.0169	-7.5
259465_at	At1g19030	Hypothetical protein	0.0375	0.0428	-7.4
265529_at	At2g06230	Hypothetical protein	0.0075	0.0189	-7.3
249001_at	At5g44990	Hypothetical protein	0.0328	0.0398	-7.2
263860_at	At2g04330	Hypothetical protein	0.0225	0.0329	-7.2
265215_at	At1g05040	Hypothetical protein	0.0489	0.0495	-7.1
266049_at	At2g40780	Hypothetical protein	0.0002	0.0034	-7.1
257742_at	At3g27370	Hypothetical protein	0.0438	0.0467	-6.9
262902_x_at	At1g59930	Hypothetical protein	0.0001	0.0019	-6.8
261251_at	At1g05920	Hypothetical protein	0.0200	0.0309	-6.8
263742_at	At2g20625	Hypothetical protein	0.0068	0.0181	-6.3
265812_at	At2g18070	Hypothetical protein	0.0073	0.0187	-6.2
248645_at	At5g49150	Hypothetical protein	0.0262	0.0356	-6.1
255918_at	At5g28570	Hypothetical protein	0.0096	0.0215	-6.0
252757_at	At3g42700	Hypothetical protein	0.0348	0.0411	-5.9
263338_at	At2g05000	Hypothetical protein	0.0250	0.0347	-5.9
257418_at	At1g30850	Hypothetical protein	0.0001	0.0028	-5.9
263091_at	At2g16200	Hypothetical protein	0.0019	0.0101	-5.8
254700_at	At4g18000	Hypothetical protein	0.0397	0.0441	-5.7
260658_at	At1g19410	Hypothetical protein	0.0072	0.0186	-5.7
264943_at	At1g76910	Hypothetical protein	0.0323	0.0395	-5.7
252581_at	At3g45500	Hypothetical protein	0.0374	0.0427	-5.6
260198_at	At1g67635	Hypothetical protein	0.0002	0.0039	-5.4
254139_at	At4g24600	Hypothetical protein	0.0419	0.0454	-5.4
249455_s_at	At5g39540	Hypothetical protein	0.0469	0.0483	-5.2
246720_at	At5g28950	Hypothetical protein	0.0040	0.0145	-5.2
257301_at	At3g30190	Hypothetical protein	0.0007	0.0067	-5.1
254712_at	At4g18080	Hypothetical protein	0.0196	0.0306	-5.0
257316_at	At3g30750	Hypothetical protein	0.0470	0.0484	-4.9
245203_at	At3g33080	Hypothetical protein	0.0001	0.0024	-4.9
261288_at	At1g37010	Hypothetical protein	0.0206	0.0314	-4.9
265785_at	At2g07290	Hypothetical protein	0.0005	0.0055	-4.8
254511_at	At4g20220	Hypothetical protein	0.0001	0.0024	-4.7
257489_at	At1g07330	Hypothetical protein	0.0008	0.0071	-4.6
252749_at	At3g43390	Hypothetical protein	0.0498	0.0499	-4.6
245220_at	At1g59171	Hypothetical protein	0.0405	0.0446	-4.2
249062_at	At5g44270	Hypothetical protein	0.0084	0.0200	-4.1
248141_at	At5g55010	Hypothetical protein	0.0017	0.0098	-3.9
257962_at	At3g19790	Hypothetical protein	0.0161	0.0279	-3.9
249648_at	At5g37050	Hypothetical protein	0.0137	0.0257	-3.8
249394_at	At5g40180	Hypothetical protein	0.0073	0.0188	-3.8
256692_at	At3g32050	Hypothetical protein	0.0000	0.0014	-3.7
267203_at	At2g31035	Hypothetical protein	0.0380	0.0431	-3.7
246823_at	At5g26970	Hypothetical protein	0.0450	0.0474	-3.6
249615_x_at	At5g37420	Hypothetical protein	0.0433	0.0464	-3.6
265638_at	At2g27340	Hypothetical protein	0.0001	0.0028	-3.4
260456_at	At1g72490	Hypothetical protein	0.0144	0.0263	-3.4
254322_at	At4g22600	Hypothetical protein	0.0108	0.0228	-3.3
262327_at	At1g64130	Hypothetical protein	0.0046	0.0152	-3.3
255339_at	At4g04480	Hypothetical protein	0.0040	0.0145	-3.2
260315_at	At1g63820	Hypothetical protein	0.0171	0.0286	-3.1
265746_at	At2g06630	Hypothetical protein	0.0089	0.0206	-3.1
264412_at	At1g43260	Hypothetical protein	0.0192	0.0303	-3.0
260642_at	At1g53260	Hypothetical protein	0.0327	0.0398	-3.0
252873_at	At4g40020	Hypothetical protein	0.0132	0.0253	-2.6
256685_at	At3g32100	Hypothetical protein	0.0389	0.0437	-2.6
250652_at	At5g06920	Hypothetical protein	0.0066	0.0179	-2.5
255712_at	At4g00280	Hypothetical protein	0.0015	0.0091	-2.5
252783_at	At3g43020	Pseudogene; Hypothetical protein	0.0129	0.0251	-5.4
262271_x_at	At1g42360	Pseudogene; Hypothetical protein	0.0014	0.0090	-4.3
246128_at	At5g32540	Pseudogene; Hypothetical protein	0.0225	0.0329	-4.0
263272_at	At2g11650	Pseudogene; Hypothetical protein	0.0492	0.0496	-4.0
265409_at	At2g16830	Pseudogene; Hypothetical protein	0.0354	0.0415	-2.6
256195_at	At1g36820	Unknown protein	0.0154	0.0273	-18.0

246360_at	At1g40952	Unknown protein	0.0160	0.0278	-6.6
264213_at	At1g65400	Unknown protein	0.0073	0.0187	-3.6
264072_at	At2g10750	Unknown protein	0.0005	0.0055	-9.1
264112_at	At2g13675	Unknown protein	0.0000	0.0004	-2.7
265578_at	At2g20090	Unknown protein	0.0416	0.0453	-6.2
266907_at	At2g34550	Unknown protein	0.0042	0.0147	-4.6
245095_at	At2g40870	Unknown protein	0.0412	0.0450	-3.5
258349_at	At3g17610	Unknown protein	0.0075	0.0190	-4.7
257784_at	At3g26970	Unknown protein	0.0073	0.0188	-5.4
257220_at	At3g27812	Unknown protein	0.0173	0.0287	-6.9
252028_at	At3g52650	Unknown protein	0.0256	0.0352	-3.0
251953_at	At3g53660	Unknown protein	0.0179	0.0292	-11.7
252897_at	At4g39490	Unknown protein	0.0016	0.0093	-3.7
246839_at	At5g26718	Unknown protein	0.0011	0.0078	-5.1
246670_at	At5g29584	Unknown protein	0.0148	0.0267	-2.8
249667_at	At5g35850	Unknown protein	0.0192	0.0303	-5.9
254521_at	At5g44810	Unknown protein	0.0155	0.0274	-6.0

[a] Probe set ID represents Affymeterix probe set number.

[b] AGI represents *Arabidopsis* Genome Initiative (AGI) locus identifier that corresponds to each gene represented on the array.

[c] q-value ≤ 0.05 (5% False discovery rate) was used to determine genes differentially expressed in PPV-infected leaves relative to mock-inoculated control leaves.

[d] p-values ($p \leq 0.05$) from the ANOVA were used to calculate q-value after adjusing the values using Benjamini and Hochberg [21] multiple testing correction.

[e] Calculation of fold changes was defined in Methods.

[f] Determined followingt the method of the *Arabidopsis* MIPS (Munich Information Centre for Protein Sequences) fucntional classification scheme.
