# Supplementary material for: Altered gene expression changes in Arabidopsis leaf tissues and protoplasts in response to Plum pox virus infection
Source: BMC Genomics. 2008 Jul 9;9:325. doi: 10.1186/1471-2164-9-325 (PMC2478689; doi:10.1186/1471-2164-9-325)
Supplement: Additional file 4 — Supplemental Table 4. Expression levels of common genes induced in PPV-infected Arabidopsis protoplasts and in infected leaf tissues. [file 1471-2164-9-325-S4.pdf]

Supplemental Table 4. Expression levels of common genes induced in PPV-infected *Arabidopsis* protoplasts and in infected leaf tissues

Probe set IDs [a]	AGI[b] locus	Description	Expression levels in pPPV-SK68 transfected protoplast cells					Expression levels in PPV infected leaf tissues		
			q -value[c]	*p* -value[d]	Fold Change[e]			*q* -value	*p* -value	Fold Change[e]
					3 hpt[f]	6 hpt	12 hpt			17 dpi[g]
A. Defence and virulence[h]										
260584_at	At2g43660	β-1,3-glucanase, putative	0.0434	0.0093	-1.06	-7.69	4.60	0.0343	0.0408	4.67
266877_at	At2g44570	Glycosyl hydrolase family 9 protein	0.0397	0.0037	5.21	1.79	-1.52	0.0354	0.0415	2.76
245849_at	At5g13520	Hydrolase-like protein	0.0475	0.0376	-1.56	-3.13	4.35	0.0284	0.0371	18.81
248989_at	At5g45200	Disease resistance protein, TIR-NBS-LRR	0.0431	0.0085	-1.49	6.39	2.12	0.0437	0.0466	10.84
B. Cellular communication/Signal transduction mechanism/transmembrane signal transduction										
257229_at	At3g16490	Calmodulin-binding family protein	0.0495	0.0485	4.01	-8.33	2.48	0.0096	0.0215	4.37
E. Metabolism/energy/membrane associated proteins										
256375_at	At1g66720	Carboxyl methyltransferase family protein	0.0475	0.0319	6.18	-1.28	-2.86	0.0194	0.0304	5.37
263068_at	At2g17580	Polynucleotide adenylyltransferase protein	0.0447	0.0183	-3.03	-1.61	3.68	0.0275	0.0365	3.10
258962_at	At3g10570	Cytochrome P450, putative	0.0401	0.0044	-1.20	-1.01	6.66	0.0126	0.0247	9.08
257143_at	At3g20110	Cytochrome P450, putative	0.0441	0.0120	1.34	4.49	-5.00	0.0448	0.0473	3.31
253502_at	At4g31940	Cytochrome P450-like protein	0.0405	0.0046	-14.29	6.81	-2.17	0.0018	0.0099	14.44
F. Proteins with binding function or cofactor requirement										
261029_at	At1g17330	Metal-dependent phosphohydrolase protein	0.0417	0.0068	1.19	3.60	-1.52	0.0102	0.0221	4.51
260921_at	At1g21540	AMP-binding protein, putative	0.0460	0.0228	-2.22	1.68	5.50	0.0456	0.0478	3.61
258028_at	At3g27473	DC1 domain containing protein	0.0479	0.0397	3.61	-3.70	-1.39	0.0275	0.0365	3.49
254771_at	At4g13380	Heavy metal domain containing protein	0.0475	0.0378	6.90	-4.00	1.34	0.0167	0.0283	2.59
247941_at	At5g57200	ENTH domain-containing protein	0.0461	0.0230	2.82	-11.11	2.00	0.0274	0.0363	2.69
H. Protein fate										
250162_at	At5g15250	FtsH protease, putative	0.0479	0.0398	1.56	10.47	-1.75	0.0397	0.0442	3.93
I. Transcription/splicing/RNA processing										
246790_at	At5g27610	MYB family transcription factor	0.0453	0.0210	-5.26	-1.04	3.43	0.0059	0.0170	3.69
267177_at	At2g37580	Zinc RING finger family protein, C3HC4-type	0.0474	0.0293	13.22	2.20	-1.09	0.0410	0.0443	4.06
K. Transposable elements, viral and plasmid proteins										
266586_s_at	At4g08060	CACTA-like transposase family	0.0388	0.0005	-5.00	8.51	-2.22	0.0068	0.0181	2.51
266470_at	At2g31080	Non-LTR retrotransposon family	0.0445	0.0162	-1.20	-5.00	5.05	0.0402	0.0444	3.21
O. Unknown										
261414_at	At1g07795	Unknown protein	0.0475	0.0318	-1.92	1.24	3.24	0.0108	0.0228	2.94
261529_at	At1g14430	Unknown protein	0.0434	0.0089	-1.52	10.14	1.50	0.0062	0.0174	3.11
262465_at	At1g50270	Unknown protein	0.0494	0.0468	-1.37	6.27	2.04	0.0299	0.0381	4.35
245655_at	At1g56530	Unknown protein	0.0434	0.0091	7.94	-2.50	-6.67	0.0235	0.0336	6.62
263672_at	At2g04820	Unknown protein	0.0405	0.0048	-3.70	8.74	-2.17	0.0135	0.0256	4.12
266128_at	At2g45000	Unknown protein	0.0401	0.0042	6.49	-1.28	1.02	0.0410	0.0449	3.50
263781_at	At2g46360	Unknown protein	0.0445	0.0159	2.19	7.61	-2.70	0.0326	0.0397	4.01
257302_at	At3g30220	Unknown protein	0.0434	0.0091	3.52	-3.57	1.12	0.0017	0.0098	2.68
246811_at	At5g27170	Unknown protein	0.0482	0.0407	-3.23	1.37	3.66	0.0280	0.0368	2.62
249099_at	At5g43550	Unknown protein	0.0493	0.0456	5.14	1.99	-3.13	0.0228	0.0332	4.01
248941_s_at	At5g45460	Unknown protein	0.0457	0.0219	-1.72	1.08	4.22	0.0427	0.0460	5.82
248706_at	At5g48530	Unknown protein	0.0476	0.0388	-3.23	-2.86	3.99	0.0403	0.0445	3.16

[a] Probe set ID represents Affymeterix probe set number.

[b] AGI represents *Arabidopsis* Genome Initiative (AGI) locus identifier that corresponds to each gene represented on the array.

[c] *q* -value ≤ 0.05 (5% False discovery rate) was used to determine genes differentially expressed in PPV-infected leaves relative to mock-inoculated control leaves.

[d] *p* -values ($p \leq 0.05$) from the ANOVA were used to calculate *q* -value after adjusing the values using Benjamini and Hochberg [21] multiple testing correction.

[e] Calculation of fold changes was defined in Methods.

[f] hpt, hours post transfection.

[g] dpi, days post inoculation.

[h] Determined followingt the method of the *Arabidopsis* MIPS (Munich Information Centre for Protein Sequences) fucntional classification scheme.
