# Supplementary material for: Altered gene expression changes in Arabidopsis leaf tissues and protoplasts in response to Plum pox virus infection
Source: BMC Genomics. 2008 Jul 9;9:325. doi: 10.1186/1471-2164-9-325 (PMC2478689; doi:10.1186/1471-2164-9-325)
Supplement: Additional file 5 — Supplemental Table 5. Expression levels of common genes repressed in PPV-infected Arabidopsis protoplasts and in infected leaf tissues. [file 1471-2164-9-325-S5.pdf]

Supplemental Table 5. Expression levels of common genes repressed in PPV-infected *Arabidopsis* protoplasts and in infected leaf tissues

Probe set IDs [a]	AGI[b] locus	Description	Expression levels in pPPV-SK68 transfected protoplast cells					Expression levels in PPV infected leaf tissues		
			q -value[c]	*p* -value[d]	Fold Change[e]			*q* -value	*p* -value	Fold Change[e]
					3 hpt[f]	6 hpt	12 hpt			17 dpi[g]
A. Defence and virulence[h]										
257441_at	At2g04020	Gdsl-Motif Lipase/Hydrolase Family Protein	0.0441	0.0113	2.36	2.96	-5.39	0.0037	0.0139	-14.9
253226_at	At4g35010	Glycosyl Hydrolase Family 35 Protein	0.0465	0.0237	-3.12	-2.75	1.10	0.0328	0.0398	-4.2
C. Cell Cycle/ DNA processing/chromatin regulation and cytoskeleton reorganization										
253142_at	At4g35520	Dna Mismatch Repair Family Protein	0.0475	0.037	-1.38	1.00	-3.32	0.0178	0.0292	-6.9
252428_at	At3g47660	Regulator Of Chromosome Condensation (Rcc1) Protein	0.0466	0.0252	-3.49	3.14	1.27	0.0098	0.0217	-2.8
D. Development/storage proteins										
245658_at	At1g28270	Rapid Alkalinization Factor (Ralf) Family Protein	0.0473	0.0341	-1.39	-3.22	2.81	0.0309	0.0386	-5.0
245267_at	At4g14060	Major Latex Protein-Related / Mlp-Related	0.0407	0.0054	2.66	-6.08	-1.76	0.0361	0.0419	-5.4
E. Metabolism/energy/membrane associated proteins										
266875_at	At2g44800	Oxidoreductase, 2Og-Fe(Ii) Oxygenase Family Protein	0.0445	0.0172	3.93	-1.10	-5.30	0.0003	0.0043	-7.7
252780_at	At3g42960	Alcohol Dehydrogenase (Ata1)	0.0474	0.0301	-4.93	-1.62	-1.44	0.0225	0.0329	-3.8
255359_at	At4g03950	Glucose-6-Phosphate/Phosphate Translocator, Putative	0.039	0.00167	-9.89	2.14	1.72	0.0000	0.0015	-1.7
249729_at	At5g24410	Glucosamine/Galactosamine-6-Phosphate Isomerase-Related	0.0445	0.0153	-5.24	2.42	-5.96	0.0203	0.0312	-9.1
249484_at	At5g38970	Cytochrome P450, Putative (Metabolism)	0.0390	0.0022	-1.22	-4.66	6.39	0.0068	0.0181	-17.9
F. Proteins with binding function or cofactor requirement										
257397_at	At2g20430	P21-Rho-Binding Domain Protein	0.0459	0.0225	4.40	-1.56	-3.33	0.0009	0.0071	-2.5
255149_at	At4g08150	Homeobox Protein Knotted-1 Like 1 (Knat1)	0.0447	0.0186	-9.21	1.73	-2.63	0.0165	0.0282	-2.1
245538_at	At4g15200	Fh2 Domain Containing Protein	0.0434	0.00966	-1.32	4.26	-6.15	0.0273	0.0363	-10.5
246963_at	At5g24820	Dna-Binding Protein Cnd41, Putative	0.0468	0.0267	3.27	-3.03	-1.05	0.0018	0.0099	-2.0
H. Protein fate										
248062_at	At5g55450	Protease Inhibitor	0.0445	0.0138	-1.24	1.00	-15.26	0.0010	0.0078	-13.9
I. Transcription/splicing/RNA processing										
256395_at	At3g06120	Basic Helix-Loop-Helix (Bhlh) Family Protein	0.0397	0.0035	2.75	-8.17	-1.53	0.0367	0.0423	-2.6
257656_at	At3g13228	Zinc Finger (C3Hc4-Type Ring Finger) Family Protein	0.0474	0.0309	-1.51	2.08	-7.82	0.0239	0.0340	-6.7
K. Transposable elements, viral and plasmid proteins										
263746_at	At2g21460	Copia-Like Retrotransposon Family	0.0448	0.0198	-2.95	8.04	-2.09	0.0052	0.0161	-2.9
255463_at	At4g02960	Copia-Like Retrotransposon Family	0.0445	0.0163	7.56	-5.19	-1.94	0.0476	0.0487	-5.1
255188_at	At4g07340	Replication Protein-Related	0.0401	0.00427	2.34	-10.28	-1.28	0.0339	0.0405	-13.7
255156_at	At4g07780	Gypsy-Like Retrotransposon Family (Athila)	0.0441	0.0106	1.61	2.42	-18.75	0.0313	0.0388	-11.3
245942_at	At5g19490	Repressor Protein-Related	0.0493	0.0464	2.26	-1.01	-4.80	0.0000	0.0014	-3.1
O. Unknown										
264735_s_at	At1g62060	Expressed Protein	0.0475	0.0460	1.76	-2.96	2.68	0.0409	0.0449	-3.1
260198_at	At1g67635	Expressed Protein	0.0496	0.0493	2.02	-2.78	-5.66	0.0002	0.0039	-5.4
263337_at	At2g04990	Unknown Protein	0.0475	0.0339	-2.91	2.80	-3.24	0.0171	0.0285	-1.5
265746_at	At2g06630	Hypothetical Protein	0.0445	0.0142	1.19	1.22	-40.97	0.0089	0.0206	-3.0
252665_at	At3g44140	Expressed Protein	0.0449	0.0203	1.06	-3.13	1.09	0.0035	0.0135	-2.1
255029_x_at	At4g09470	Expressed Protein	0.0465	0.0243	1.43	-1.60	-3.65	0.0014	0.0088	-1.8
254240_at	At4g23496	Expressed Protein	0.0478	0.0394	1.58	-3.73	1.20	0.0017	0.0097	-2.8
249756_at	At5g24313	Expressed Protein	0.0453	0.0204	-2.86	2.27	3.84	0.0263	0.0356	-10.3
249531_at	At5g38770	Hypothetical Protein	0.0447	0.0185	1.28	-8.76	2.30	0.0152	0.0272	-10.7
248904_at	At5g46295	Expressed Protein	0.0445	0.0169	1.53	1.94	-3.08	0.0164	0.0280	-2.4

[a]Probe set ID represents Affymeterix probe set number.

[b] AGI represents *Arabidopsis* Genome Initiative (AGI) locus identifier that corresponds to each gene represented on the array.

[c] *q* -value ≤ 0.05 (5% False discovery rate) was used to determine genes differentially expressed in PPV-infected leaves relative to mock-inoculated control leaves.

[d] *p* -values ($p \leq 0.05$) from the ANOVA were used to calculate *q* -value after adjusing the values using Benjamini and Hochberg [21] multiple testing correction.

[e] Calculation of fold changes was defined in Methods.

[f] hpt, hours post transfection.

[g] dpi, days post inoculation.

[h]Determined followingt the method of the *Arabidopsis* MIPS (Munich Information Centre for Protein Sequences) fucntional classification scheme.
