# Supplementary figures and images for: Altered gene expression changes in Arabidopsis leaf tissues and protoplasts in response to Plum pox virus infection
Source: BMC Genomics. 2008 Jul 9;9:325. doi: 10.1186/1471-2164-9-325 (PMC2478689; doi:10.1186/1471-2164-9-325)

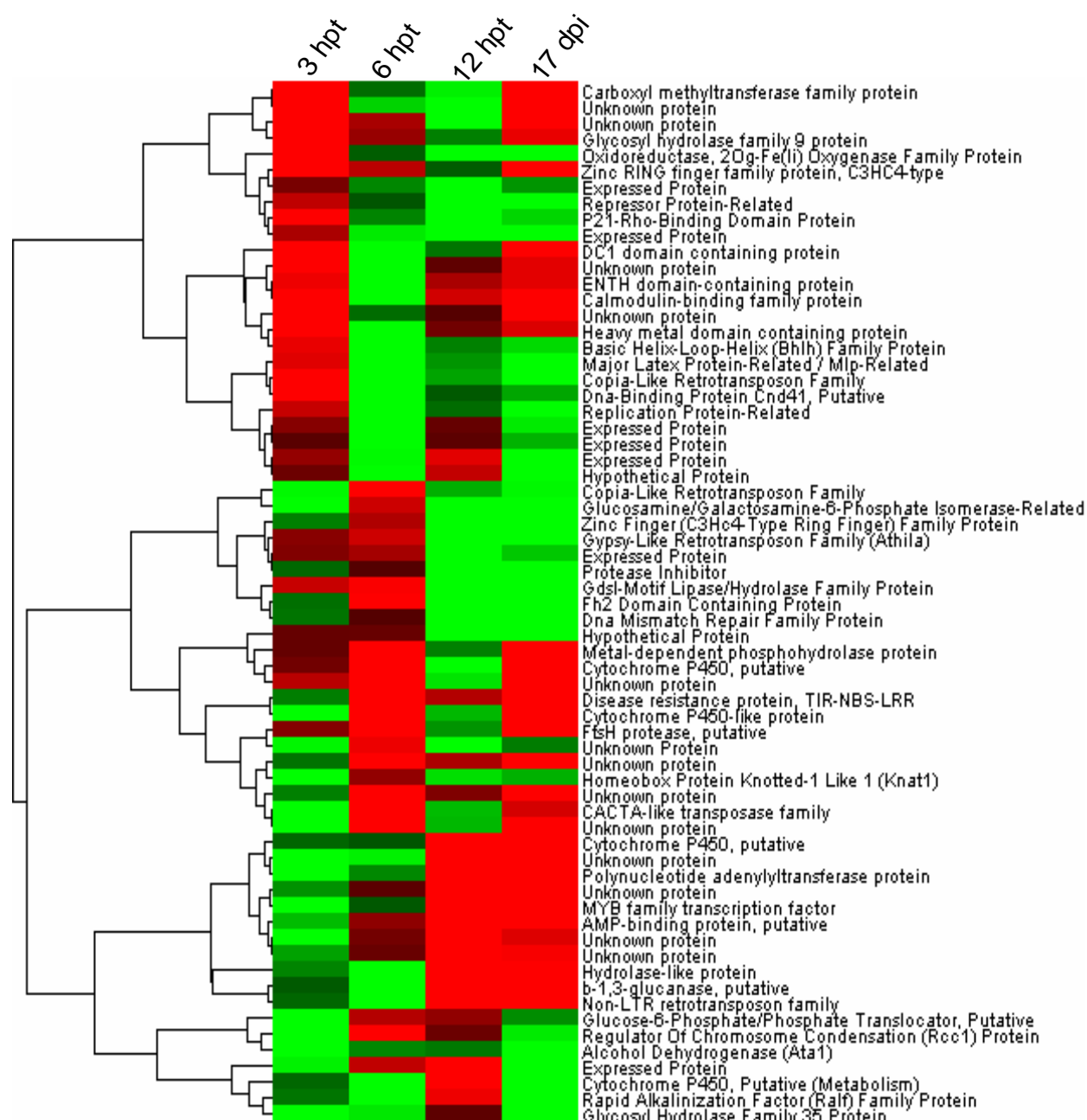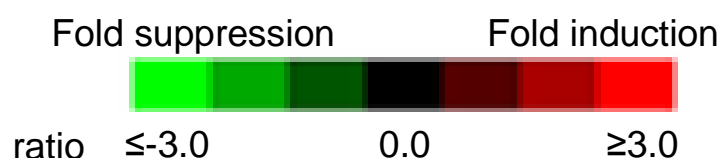

Supplement: Additional file 6 — Supplemental Figure 1. Hierarchical clustering of common genes that were significantly differentially regulated by PPV in infected leaf tissues and in transfected protoplast cells. Expression levels are color-coded with red indicating upregulation by pPPV-SK68; green indicating downregulation by pPPV-SK68 and black indicating no change in expression. The intensity of color represents the degree of gene expression levels. The putative function of each gene is shown on the right side of the cluster. [file 1471-2164-9-325-S6.pdf]

**A.**

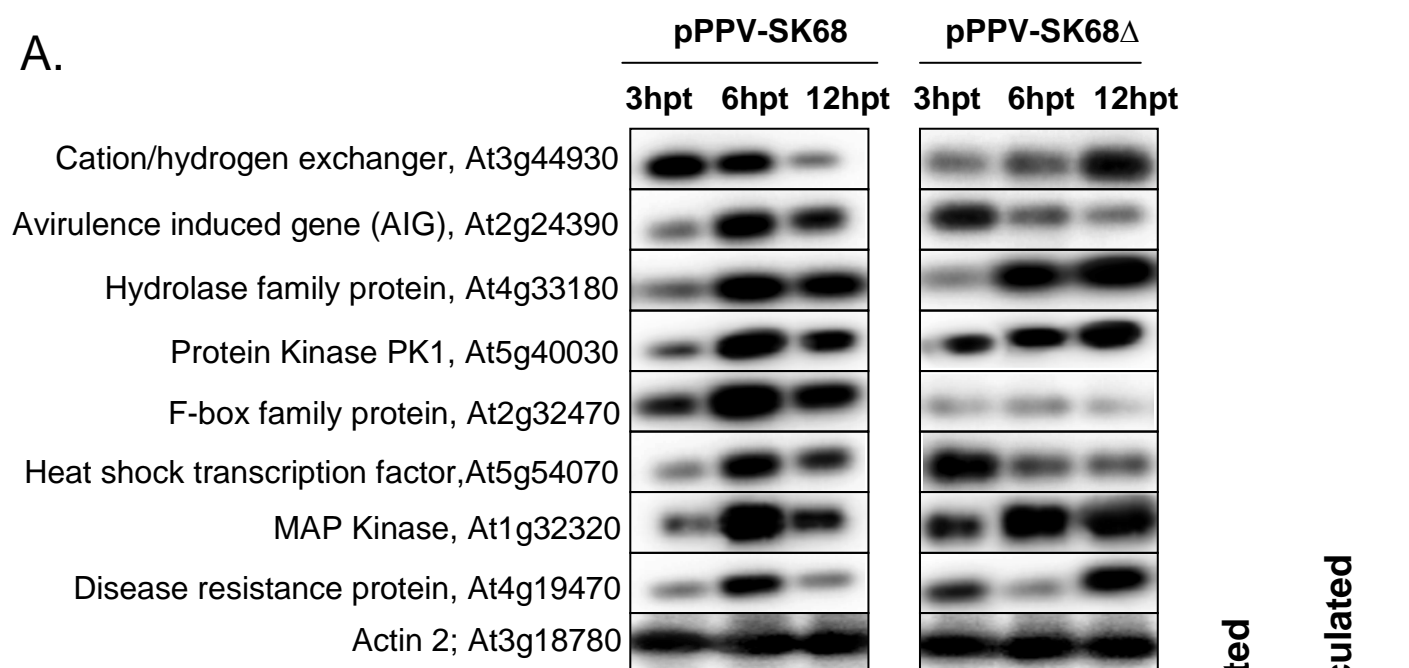

**B.**

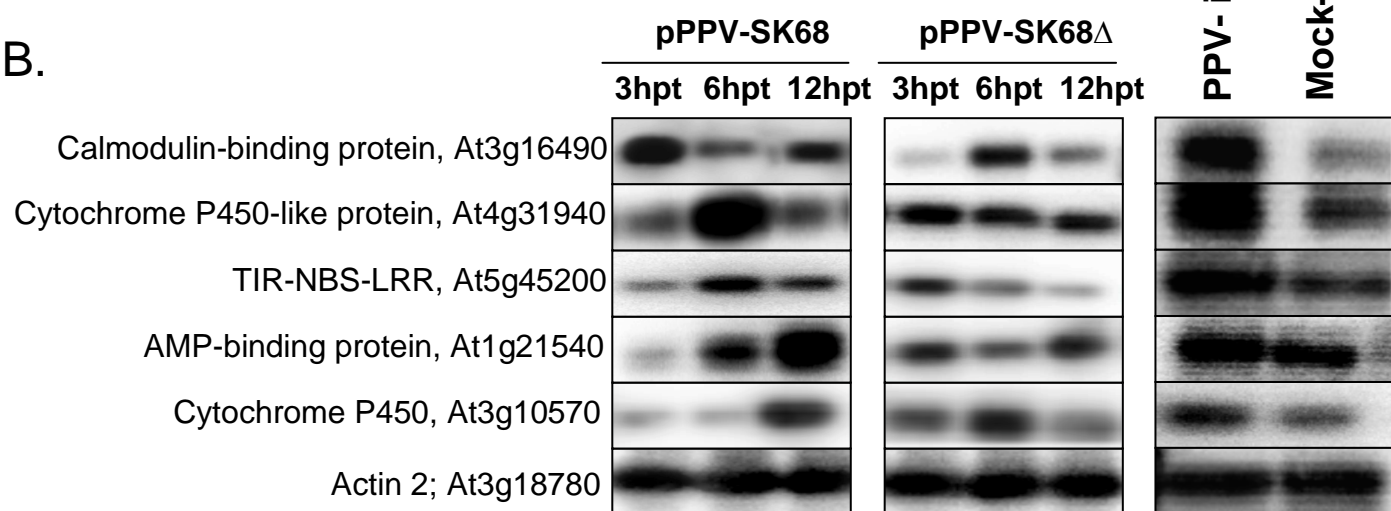

Supplement: Additional file 11 — Supplemental Figure 3. Confirmation of microarray data by sqRT-PCR and Northern hybridizations. Panel A shows confirmation of microarray data using sqRT-PCR for genes induced in PPV-infected Arabidopsis protoplasts. Panel B shows confirmation of microarray data using sqRT-PCR (left panel) and Northern hybridizations (right panel) for Arabidopsis genes differentially regulated in PPV-infected protoplasts and in PPV-infected leaves. Probes for sqRT-PCR and Northern hybridizations were generated by PCR amplification of Arabidopsis cDNA using gene specific primers shown in Table 3. sqRT-PCR of the constitutively expressed Actin 2 gene (At3g18780) was used as a loading control. pPPV-SK68, a PPV infectious cDNA clone used to transfect protoplasts; pPPV-SK68Δ, a mutant non-infectious clone of pPPV-SK68 was used as a control; hpt, hours post transfection. [file 1471-2164-9-325-S11.pdf]
