# Supplementary material for: Altered gene expression changes in Arabidopsis leaf tissues and protoplasts in response to Plum pox virus infection
Source: BMC Genomics. 2008 Jul 9;9:325. doi: 10.1186/1471-2164-9-325 (PMC2478689; doi:10.1186/1471-2164-9-325)
Supplement: Additional file 7 — Supplemental Figure 2. K-means profiling of gene expression using 411 Arabidopsis genes differentially regulated by PPV in transfected protoplasts at three different time points. The expression profiles were grouped into twelve distinct cluster groups. The AGI locus identifier of each gene differentially regulated by PPV in the transfected protoplasts at different time points in each cluster group is shown on the right side of the cluster. Values on the y-axis indicate the relative expression level of the gene, while the x-axis represents hours post transfection. Number of genes belonging to each cluster is shown in the cluster inset. [file 1471-2164-9-325-S7.pdf]

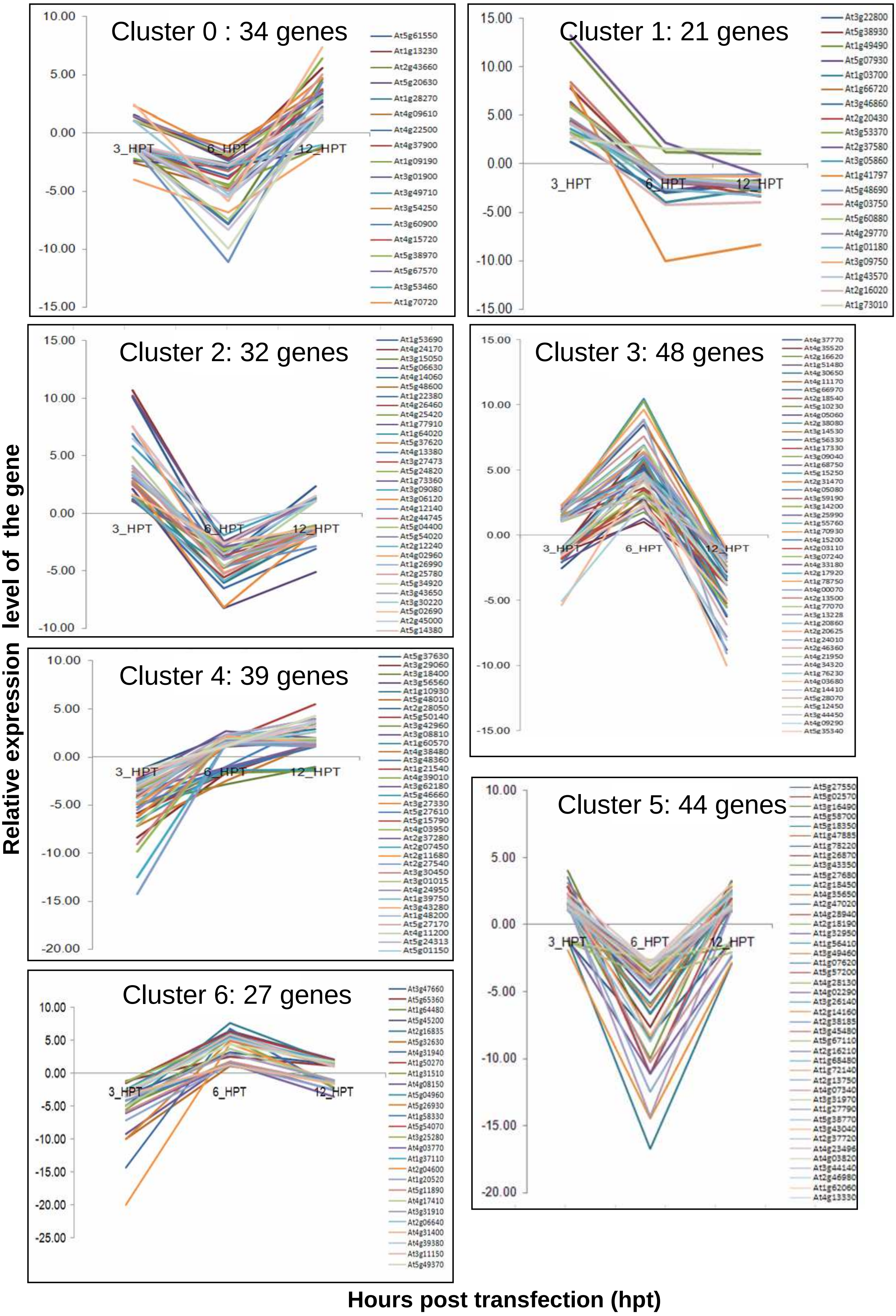

Cluster 0 : 34 genes
Cluster 1: 21 genes
Cluster 2: 32 genes
Cluster 3: 48 genes
Cluster 4: 39 genes
Cluster 5: 44 genes
Cluster 6: 27 genes
3_HPT
6_HPT
12_HPT
10.00
5.00
0.00
-5.00
-10.00
-15.00
-20.00
-25.00
15.00
At5g61550
At1g13230
At2g43660
At5g20630
At1g28270
At4g09610
At4g22500
At4g37900
At1g09190
At3g01900
At3g49710
At3g54250
At3g60900
At4g15720
At5g38970
At5g67570
At3g53460
At1g70720
At3g22800
At5g38930
At1g49490
At5g07930
At1g03700
At1g66720
At3g46860
At2g20430
At3g53370
At2g37580
At3g05860
At1g41797
At5g48690
At4g03750
At5g60880
At4g29770
At1g01180
At3g09750
At1g43570
At2g16020
At1g73010
Relative expression level of the gene
Hours post transfection (hpt)

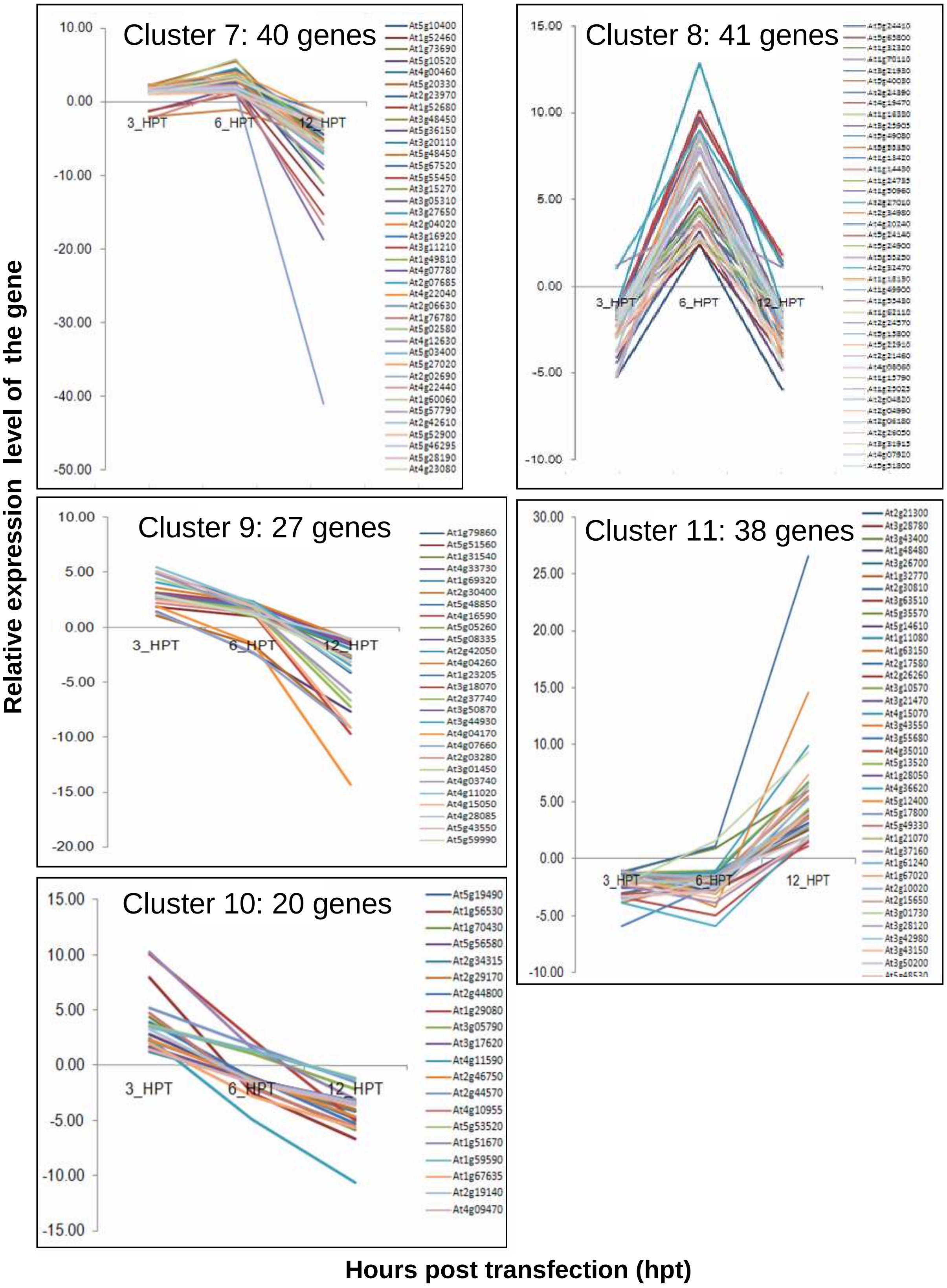
Cluster 7: 40 genes
Cluster 8: 41 genes
Cluster 9: 27 genes
Cluster 10: 20 genes
Cluster 11: 38 genes
3_HPT
6_HPT
12_HPT
Relative expression level of the gene
Hours post transfection (hpt)
