# Supplementary material for: Altered gene expression changes in Arabidopsis leaf tissues and protoplasts in response to Plum pox virus infection
Source: BMC Genomics. 2008 Jul 9;9:325. doi: 10.1186/1471-2164-9-325 (PMC2478689; doi:10.1186/1471-2164-9-325)
Supplement: Additional file 8 — Supplemental Table 6. Gene expression profiles of Arabidopsis genes differentially regulated by PPV infectious clone in transfected protoplasts belonging to twelve distinct cluster groups. [file 1471-2164-9-325-S8.pdf]

Supplemental Table 6. Expression profiles of *Arabidopsis* genes differentially regulated by PPV infectious clone in transfected protoplasts belonging to twelve distinct cluster groups

Probe set IDs	AGI[b] locus	Description	*q*-value[c]	*p*-value[d]	Cluster groups[e]	Fold Change[f] 3 hpt[g]	6 hpt	12 hpt	Functional category according to *Arabidopsis* MIPS database[h]
**CLUSTER 0: Majority of the genes repressed at 3 hpt and significantly repressed (*q*-value ≤ 0.05; ≤ 2.5) at 6 hpt followed by significant induction (*q*-value ≤ 0.05; ≥ 2.5) at 12 hpt**									
247531_at	At5g61550	Protein kinase 1, PK1	0.0445	0.0160	0	1.55	-2.00	3.35	Cellular communication/Signal transduction mechanism/transmembrane signal transduction
262774_at	At1g13230	Chloroplast nucleoid DNA binding protein, putative	0.0448	0.0195	0	1.53	-2.27	5.58	Chloroplast
260584_at	At2g43660	β-1,3-Glucanase, Putative	0.0434	0.0093	0	-1.07	-7.84	4.60	Defence and virulence
246004_at	At5g20630	Germin-Like Protein; Ger3	0.0488	0.0444	0	-2.38	-2.94	-1.39	Defence and virulence
245658_at	At1g28270	Rapid Alkalinization Factor (Ralf) Family Protein	0.0473	0.0341	0	-1.39	-3.22	2.81	Development/storage proteins
255049_at	At4g09610	Gibberellin-Regulated Protein 2 (Gasa2) / Gibberellin-Responsive Protein	0.0492	0.0453	0	-2.55	-4.79	2.23	Development/storage proteins
254316_at	At4g22500	Pseudogene, Glycine-Rich Protein Family	0.0475	0.0336	0	-1.30	-3.70	1.33	Development/storage proteins
253012_at	At4g37900	Glycine-rich protein	0.0445	0.0158	0	1.01	-2.08	3.61	Development/storage proteins
264251_at	At1g09190	Pentatricopeptide (Ppr) Repeat-Containing Protein	0.0475	0.0380	0	-2.27	-4.48	-1.22	Primary /secondary metabolism
258973_at	At3g01900	Cytochrome P450 family protein	0.0475	0.0337	0	1.34	-2.44	2.62	Primary /secondary metabolism
252245_at	At3g49710	Pentatricopeptide (Ppr) Repeat-Containing Protein	0.0431	0.0085	0	-1.20	-2.63	1.23	Primary /secondary metabolism
251881_at	At3g54250	Mevalonate diphosphate decarboxylase, putative	0.0476	0.0383	0	1.04	-1.12	3.80	Primary /secondary metabolism
251394_at	At3g60900	Fasciclin-Like Arabinogalactan-Protein (Fla10)	0.0441	0.0105	0	-1.52	-7.87	2.29	Primary /secondary metabolism
245508_at	At4g15720	Pentatricopeptide (Ppr) Repeat-Containing Protein	0.0397	0.0038	0	-1.45	-4.00	3.71	Primary /secondary metabolism
249484_at	At5g38970	Cytochrome P450, Putative (Metabolism)	0.0390	0.0022	0	-1.22	-4.66	6.39	Primary /secondary metabolism
247004_at	At5g67570	Pentatricopeptide (Ppr) Repeat-Containing Protein	0.0461	0.0229	0	-1.52	-5.00	1.18	Primary /secondary metabolism
251956_at	At3g53460	RNA-Binding Protein; Cp 29	0.0493	0.0462	0	-1.56	-2.86	1.10	Proteins with binding function or cofactor requirement
260178_at	At1g70720	invertase/pectin methylesterase inhibitor protein	0.0434	0.0062	0	2.33	-1.72	4.71	Re-assembly of cell wall associated genes
260573_at	At2g47280	Pectinesterase Family Protein	0.0474	0.0303	0	-1.27	-11.11	4.37	Re-assembly of cell wall associated genes
247477_at	At5g62340	Invertase/Pectin Methylesterase Inhibitor Family Protein	0.0445	0.0164	0	-1.28	-3.33	1.77	Re-assembly of cell wall associated genes
266841_at	At2g26150	Heat shock transcription factor, putative	0.0475	0.0346	0	1.11	-2.00	3.16	Transcription/splicing/RNA processing
256918_s_at	At3g18960	Transcriptional factor B3 family protein	0.0441	0.0129	0	1.44	-1.61	3.51	Transcription/splicing/RNA processing
249606_at	At5g37260	Myb Family Transcription Factor	0.0407	0.0055	0	-1.64	-2.86	-1.04	Transcription/splicing/RNA processing
260193_at	At1g67640	Lysine And Histidine Specific Transporter, Putative	0.0453	0.0208	0	-3.98	-6.83	-1.43	Transporters
266213_s_at	At2g06870	Mutator-Like Transposase Family	0.0441	0.0109	0	-1.22	-2.87	1.55	Transposable elements, viral and plasmid proteins
266470_at	At2g31080	Non-Ltr Retrotransposon Family (Line)	0.0445	0.0162	0	-1.20	-5.00	5.05	Transposable elements, viral and plasmid proteins
246667_at	At5g34840	Mutator-Like Transposase Family	0.0465	0.0242	0	-1.55	-7.45	1.06	Transposable elements, viral and plasmid proteins
262625_at	At1g06440	Expressed Protein	0.0465	0.0239	0	-1.32	-5.56	1.33	Unknown proteins
246353_s_at	At1g39190	Unknown Protein	0.0485	0.0388	0	1.03	-5.26	3.07	Unknown proteins
265655_at	At2g13920	Unknown Protein	0.0485	0.0391	0	2.43	-5.92	7.37	Unknown proteins
265421_s_at	At2g20616	Expressed Protein	0.0482	0.0413	0	-1.10	-5.56	1.92	Unknown proteins
256599_at	At3g14760	Expressed Protein	0.0441	0.0108	0	-1.04	-2.78	2.06	Unknown proteins
255531_at	At4g02160	Hypothetical Protein	0.0390	0.0024	0	-1.16	-10.00	1.81	Unknown proteins
262002_at	At1g64450	Proline-Rich Family Protein	0.0390	0.0030	0	-1.28	-8.33	1.25	Structural proteins
**CLUSTER 1: Majority of the genes singificantly induced (*q*-value ≤ 0.05; ≥ 2.5) at 3 hpt followed by significant repression (q-value ≤ 0.05; ≤ 2.5) at 6 or 12 hpt**									
258342_at	At3g22800	Leucine-Rich Repeat Family Protein / Extensin Family Protein	0.0441	0.0106	1	2.27	-3.03	-2.00	Cellular communication/Signal transduction mechanism/transmembrane signal transduction
249477_s_at	At5g38930	Germin-Like Protein, Putative	0.0475	0.0319	1	7.80	-1.85	-2.50	Defence and virulence
262393_at	At1g49490	Extensin family protein	0.0494	0.0442	1	12.55	1.16	1.01	Development/storage proteins
250579_at	At5g07930	Terminal Ear1 Protein, Putative	0.0458	0.0224	1	6.35	-2.78	-1.64	Development/storage proteins
264842_at	At1g03700	Integral Membrane Family Protein	0.0390	0.0025	1	4.38	-3.95	-2.56	Intracellular membrane/membrane bound
256375_at	At1g66720	S-Adenosyl-L-Methionine:Carboxyl Methyltransferase Family Protein	0.0475	0.0319	1	6.18	-1.28	-2.86	Primary /secondary metabolism
252500_at	At3g46860	Serine protease inhibitor protein	0.0495	0.0483	1	4.23	-1.32	-1.23	Protein fate
257397_at	At2g20430	P21-Rho-Binding Domain Protein	0.0459	0.0225	1	4.40	-1.56	-3.33	Proteins with binding function or cofactor requirement
251964_at	At3g53370	DNA-binding family protein, S1FA	0.0390	0.0026	1	3.30	-1.39	-1.22	Proteins with binding function or cofactor requirement
267177_at	At2g37580	Zinc RING finger family protein, C3HC4-type	0.0474	0.0293	1	13.22	2.20	-1.09	Transcription/splicing/RNA processing
258734_at	At3g05860	Mads-Box Protein (Agl45)	0.0474	0.0299	1	3.63	-1.41	-2.70	Transcription/splicing/RNA processing
259988_at	At1g41797	Athila Orf 1, Putative	0.0441	0.0108	1	8.03	-10.06	-8.32	Transposable elements, viral and plasmid proteins
248662_at	At5g48690	similar to ubiquitin-associated protein	0.0474	0.0341	1	5.95	-1.23	-1.11	Ubiquitin like conjugating enzyme activity
261573_at	At1g01180	Expressed Protein	0.0476	0.0475	1	4.25	-2.63	-3.24	Unknown proteins
262718_at	At1g43570	Expressed protein	0.0390	0.0023	1	4.09	-1.37	-2.22	Unknown proteins
262369_at	At1g73010	Expressed protein	0.0390	0.0013	1	2.60	1.50	1.35	Unknown proteins

263094_at	At2g16020	Hypothetical Protein	0.0475	0.0262	1	3.04	-4.20	-3.95	Unknown proteins
258703_at	At3g09750	Expressed protein	0.0471	0.0339	1	4.14	-1.30	-1.30	Unknown proteins
255399_at	At4g03750	Hypothetical protein	0.0388	0.0008	1	8.43	-1.79	-2.27	Unknown proteins
253689_at	At4g29770	Expressed protein	0.0496	0.0426	1	4.67	-1.82	-2.44	Unknown proteins
247599_at	At5g60880	Expressed protein	0.0475	0.0364	1	5.93	-1.41	-1.96	Unknown proteins

CLUSTER 2: Majority of the genes singificantly induced (*q* -value ≤ 0.05; ≥ 2.5) at 3 hpt followed by significant repression (q-value ≤ 0.05; ≤ 2.5) at 6hpt and repression at 12 hpt.

259962_at	At1g53690	Dna-Directed RNA Polymerases I, Ii, And Iii Putative	0.0390	0.0033	2	10.13	-4.00	2.38	Cell Cycle/ DNA processing/chromatin regulation and cytoskeleton reorganization
254205_at	At4g24170	Kinesin Motor Family Protein	0.0425	0.0078	2	10.70	-2.63	1.18	Cell Cycle/ DNA processing/chromatin regulation and cytoskeleton reorganization
257233_at	At3g15050	Calmodulin-Binding Family Protein	0.0445	0.0145	2	2.70	-5.88	-1.85	Cellular communication/Signal transduction mechanism/transmembrane signal transduction
250682_x_at	At5g06630	Extensin-Like Family Protein	0.0474	0.0308	2	2.11	-8.25	-5.11	Development/storage proteins
245267_at	At4g14060	Major Latex Protein-Related / Mlp-Related	0.0407	0.0054	2	2.66	-6.08	-1.76	Development/storage proteins
248658_at	At5g48600	Structural Maintenance Of Chromosomes (Smc) Family Protein	0.0465	0.0238	2	1.13	-3.33	-1.30	Development/storage proteins
261923_at	At1g22380	Udp-Glucoronosyl/Udp-Glucosyl Transferase Family Protein	0.0493	0.0463	2	1.51	-6.55	-3.14	Matrix polymers
253962_at	At4g26460	Similar To S-Adenosyl-L-Methionine Carboxyl Methyltransferase	0.0475	0.0351	2	1.25	-5.56	-1.33	Primary /secondary metabolism
254065_at	At4g25420	Gibberellin 20-Oxidase	0.0474	0.0296	2	1.50	-4.00	-1.61	Primary /secondary metabolism
262184_at	At1g77910	NADH Dehydrogenase	0.0477	0.0489	2	10.21	-2.44	1.19	Primary /secondary metabolism
262333_at	At1g64020	Serine Protease Inhibitor-Related	0.0475	0.0372	2	1.11	-4.76	-1.47	Protein fate
249571_at	At5g37620	Dc1 Domain-Containing Protein	0.0390	0.0025	2	2.81	-4.76	-1.22	Proteins with binding function or cofactor requirement
254771_at	At4g13380	Heavy-Metal-Associated Domain-Containing Protein	0.0475	0.0378	2	6.90	-4.00	1.34	Proteins with binding function or cofactor requirement
258028_at	At3g27473	Dc1 Domain-Containing Protein	0.0479	0.0397	2	3.61	-3.70	-1.39	Proteins with binding function or cofactor requirement
246963_at	At5g24820	Dna-Binding Protein Cnd41, Putative	0.0468	0.0267	2	3.27	-3.03	-1.05	Proteins with binding function or cofactor requirement
245726_at	At1g73360	Lipid-Binding Start Domain-Containing Protein	0.0482	0.0409	2	1.02	-2.94	-1.41	Proteins with binding function or cofactor requirement
259201_at	At3g09080	Transducin family protein	0.0474	0.0345	2	5.82	-1.92	1.31	Proteins with binding function or cofactor requirement
256395_at	At3g06120	Basic Helix-Loop-Helix (BHLH) Family Protein	0.0397	0.0035	2	2.75	-8.17	-1.53	Transcription/splicing/RNA processing
254855_s_at	At4g12140	Zinc Finger (C3Hc4-Type Ring Finger) Family Protein	0.0405	0.0050	2	2.58	-4.61	-2.84	Transcription/splicing/RNA processing
266886_at	At2g44745	Wrky Family Transcription Factor	0.0466	0.0249	2	2.49	-4.55	-1.09	Transcription/splicing/RNA processing
245707_at	At5g04400	Nac2-Like Protein	0.0494	0.0470	2	3.08	-3.23	-1.33	Transcription/splicing/RNA processing
248212_at	At5g54020	Zinc Finger Protein-Like, Chp Type	0.0441	0.0127	2	3.09	-2.78	1.18	Transcription/splicing/RNA processing
266148_x_at	At2g12240	Cacta-Like Transposase Family (Ptta/En/Spm)	0.0475	0.0335	2	1.49	-5.88	-1.15	Transposable elements, viral and plasmid proteins
255463_at	At4g02960	Copia-Like Retrotransposon Family	0.0445	0.0163	2	7.56	-5.19	-1.94	Transposable elements, viral and plasmid proteins
265004_at	At1g26990	Copia-Like Retrotransposon Family	0.0448	0.0192	2	3.36	-4.04	-1.95	Transposable elements, viral and plasmid proteins
257367_at	At2g25780	Hypothetical Protein	0.0390	0.0016	2	3.92	-5.26	-1.49	Unknown proteins
246642_s_at	At5g34920	Unknown Protein	0.0409	0.0176	2	4.90	-4.62	1.05	Unknown proteins
252696_at	At3g43650	Unknown Protein	0.0445	0.0182	2	4.13	-4.04	-1.16	Unknown proteins
257302_at	At3g30220	Expressed Protein	0.0434	0.0091	2	3.52	-3.57	1.12	Unknown proteins
251003_at	At5g02690	Expressed Protein	0.0465	0.0244	2	1.67	-2.56	-1.25	Unknown proteins
266128_at	At2g45000	Expressed protein	0.0401	0.0042	2	6.49	-1.28	1.02	Unknown proteins
250174_at	At5g14380	Arabinogalactan-Protein, Agp6	0.0466	0.0258	2	7.52	-2.70	1.52	Structural proteins

CLUSTER 3: Majority of the genes induced at 3 hpt and significantly induced (*q* -value ≤ 0.05; ≥ 2.5) at 6hpt followed by significant repression (q-value ≤ 0.05; ≤ 2.5) at 12hpt

253066_at	At4g37770	1-Aminocyclopropane-1-Carboxylate Synthase, Putative	0.0482	0.0414	3	-2.54	2.87	-8.84	Carbohydrate/soluble sugar/startch/aminoacid metabolism
253142_at	At4g35520	Dna Mismatch Repair Family Protein	0.0475	0.0370	3	-1.38	1.00	-3.32	Cell Cycle/ DNA processing/chromatin regulation and cytoskeleton reorganization
265410_at	At2g16620	Protein Kinase-Related	0.0495	0.0485	3	-1.85	3.41	-5.56	Cellular communication/Signal transduction mechanism/transmembrane signal transduction
260514_at	At1g51480	Disease Resistance Protein, CC-NBS-LRR class, Putative	0.0482	0.0406	3	-1.02	5.48	-6.25	Defence and virulence
253627_at	At4g30650	Hydrophobic protein, putative	0.0401	0.0043	3	1.35	5.25	-1.59	Defence and virulence
254905_at	At4g11170	Disease Resistance Protein, TIR-NBS-LRR, Putative	0.0498	0.0497	3	-1.06	2.65	-3.13	Defence and virulence
247016_at	At5g66970	Signal recognition particle 54 K protein	0.0448	0.0194	3	1.93	8.47	-1.11	Development/storage proteins
265949_at	At2g18540	Cupin Family Protein	0.0494	0.0470	3	-1.25	6.88	-5.00	Development/storage proteins
250474_at	At5g10230	Annexin 7; Ann7	0.0428	0.0083	3	-1.43	4.16	-5.00	Development/storage proteins
255258_at	At4g05060	Vesicle Associated Membrane Family Protein	0.0445	0.0174	3	1.10	5.13	-3.48	Intracellular membrane/membrane bound
267094_at	At2g38080	Diphenol Oxidase, Putative	0.0485	0.0422	3	1.16	6.26	-3.85	Primary /secondary metabolism
258121_s_at	At3g14530	Geranylgeranyl Pyrophosphate Synthase, Putative	0.0448	0.0195	3	-1.79	5.64	-5.26	Primary /secondary metabolism
248013_at	At5g56330	Carbonic Anhydrase Family Protein	0.0474	0.0307	3	1.20	4.97	-2.70	Primary /secondary metabolism
261029_at	At1g17330	Metal-dependent phosphohydrolase protein	0.0417	0.0068	3	1.19	3.60	-1.52	Primary /secondary metabolism
259206_at	At3g09040	Pentatricopeptide (Ppr) Repeat-Containing Protein	0.0388	0.0008	3	-1.10	1.79	-3.22	Primary /secondary metabolism
260032_at	At1g68750	Phosphoenolpyruvate Carboxylase Family Protein	0.0458	0.0225	3	-2.05	1.28	-3.17	Primary /secondary metabolism

250162_at	At5g15250	FtsH protease, putative	0.0479	0.0398	3	1.56	10.47	-1.75	Protein fate
263245_at	At2g31470	F-Box Family Protein	0.0485	0.0422	3	2.29	8.79	-2.78	Protein fate
255256_at	At4g05080	F-Box Family Protein	0.0497	0.0496	3	-2.12	4.89	-5.53	Protein fate
251512_at	At3g59190	F-Box Family Protein	0.0475	0.0359	3	-1.12	2.31	-3.85	Protein fate
256999_at	At3g14200	DNAJ heat shock protein	0.0482	0.0413	3	1.45	10.29	-2.08	Protein folding/heat shock/chaperone activity
258068_at	At3g25990	Dna-Binding Protein Gt-1-Related	0.0471	0.0274	3	1.21	6.32	-2.86	Proteins with binding function or cofactor requirement
264562_at	At1g55760	Btb/Poz Domain-Containing Protein	0.0445	0.0162	3	-1.56	5.89	-5.00	Proteins with binding function or cofactor requirement
262303_at	At1g70930	Homeobox protein, putative	0.0490	0.0450	3	1.56	4.29	-1.10	Proteins with binding function or cofactor requirement
245538_at	At4g15200	Fh2 Domain Containing Protein	0.0434	0.0097	3	-1.32	4.26	-6.15	Proteins with binding function or cofactor requirement
266730_at	At2g03110	RNA binding Protein, Putative	0.0475	0.0329	3	-1.89	3.52	-3.86	Proteins with binding function or cofactor requirement
257531_at	At3g07240	RNA binding Protein, Putative	0.0497	0.0495	3	-1.03	3.24	-5.56	Proteins with binding function or cofactor requirement
253366_at	At4g33180	Hydrolase family protein	0.0484	0.0417	3	1.31	4.09	-1.09	Re-assembly of cell wall associated genes
264786_at	At2g17920	Zinc Finger Protein	0.0445	0.0145	3	1.86	6.88	-3.33	Transcription/splicing/RNA processing
264294_at	At1g78750	Heat shock transcription factor-like	0.0475	0.0355	3	1.10	6.41	-2.04	Transcription/splicing/RNA processing
257488_s_at	At4g00070	Zinc finger protein, related	0.0475	0.0372	3	1.26	5.99	-2.38	Transcription/splicing/RNA processing
263723_at	At2g13500	Zinc finger protein	0.0494	0.0427	3	2.08	4.66	-2.44	Transcription/splicing/RNA processing
264945_at	At1g77070	MADS-box protein MADS2, putative	0.0493	0.0457	3	1.02	3.25	-1.19	Transcription/splicing/RNA processing
257656_at	At3g13228	Zinc Finger (C3Hc4-Type Ring Finger) Family Protein	0.0474	0.0309	3	-1.51	2.08	-7.82	Transcription/splicing/RNA processing
262855_at	At1g20860	Phosphate transporter family protein	0.0409	0.0060	3	1.35	4.79	-1.25	Transporters
263742_at	At2g20625	Expressed protein	0.0390	0.0027	3	2.19	9.64	-1.02	Unknown proteins
263027_at	At1g24010	Expressed Protein	0.0405	0.0049	3	1.77	8.86	-9.09	Unknown proteins
263781_at	At2g46360	Expressed Protein	0.0445	0.0159	3	2.19	7.61	-2.70	Unknown proteins
254383_at	At4g21950	Expressed Protein	0.0475	0.0363	3	1.76	6.79	-2.65	Unknown proteins
253289_at	At4g34320	Expressed protein	0.0405	0.0048	3	1.76	6.29	-1.82	Unknown proteins
261779_at	At1g76230	Expressed Protein	0.0494	0.0431	3	-1.04	4.80	-3.85	Unknown proteins
255392_at	At4g03680	Expressed Protein	0.0475	0.0471	3	-5.39	4.70	-10.05	Unknown proteins
265834_at	At2g14410	Unknown protein	0.0445	0.0145	3	1.06	4.46	-2.00	Unknown proteins
246729_at	At5g28070	Hypothetical Protein	0.0445	0.0151	3	-1.49	4.31	-6.87	Unknown proteins
245153_at	At5g12450	Expressed Protein	0.0479	0.0298	3	-1.08	4.05	-3.70	Unknown proteins
252661_at	At3g44450	Expressed protein	0.0390	0.0022	3	1.25	2.87	-1.22	Unknown proteins
255085_at	At4g09290	Expressed Protein	0.0407	0.0163	3	-5.07	2.55	-8.07	Unknown proteins
246605_at	At5g35340	Unknown Protein	0.0465	0.0246	3	-1.61	2.34	-5.00	Unknown proteins

CLUSTER 4: Majority of the genes significantly repressed (*q* -value ≤ 0.05; ≤ 2.5) at 3hpt and induced at 6 or 12 hpt

249572_at	At5g37630	Chromosome condensation family protein	0.0466	0.0261	4	-1.49	2.24	3.92	Cell Cycle/ DNA processing/chromatin regulation and cytoskeleton reorganization
257106_at	At3g29060	Exs Family Protein / Erd1/Xpr1/Syg1 Family Protein	0.0485	0.0426	4	-8.38	-1.68	-1.23	Cellular communication/Signal transduction mechanism/transmembrane signal transduction
257718_at	At3g18400	No Apical Meristem (Nam) Family Protein	0.0474	0.0306	4	-5.00	-2.86	-1.04	Development/storage proteins
251694_s_at	At3g56560	No Apical Meristem (Nam) Family Protein	0.0477	0.0391	4	-4.20	1.50	1.76	Development/storage proteins
260463_at	At1g10930	Helicase	0.0495	0.0483	4	-4.17	1.68	2.89	Helicase activity
248729_at	At5g48010	Pentacyclic Triterpene Synthase, Putative	0.0475	0.0361	4	-6.27	1.22	1.68	Primary /secondary metabolism
266164_at	At2g28050	Pentatricopeptide (Ppr) Repeat-Containing Protein	0.0465	0.0237	4	-5.88	-1.18	1.11	Primary /secondary metabolism
248536_at	At5g50140	Ankyrin Repeat Family Protein	0.0441	0.0112	4	-5.88	-1.72	1.41	Primary /secondary metabolism
252780_at	At3g42960	Alcohol Dehydrogenase (Ata1)	0.0474	0.0301	4	-4.93	-1.62	-1.44	Primary /secondary metabolism
258670_at	At3g08810	Kelch Repeat-Containing F-Box Family Protein	0.0425	0.0080	4	-4.74	1.11	1.58	Protein fate
264966_at	At1g60570	Kelch Repeat-Containing F-Box Family Protein	0.0434	0.0096	4	-2.50	1.09	1.96	Protein fate
252994_at	At4g38480	Transducin Family Protein / Wd-40 Repeat Family Protein	0.0445	0.0154	4	-7.24	-2.56	1.40	Proteins with binding function or cofactor requirement
252367_at	At3g48360	Speckle-Type Poz Protein-Related	0.0485	0.0420	4	-4.17	-1.35	1.09	Proteins with binding function or cofactor requirement
260921_at	At1g21540	AMP-binding protein, putative	0.0460	0.0228	4	-2.22	1.68	5.50	Proteins with binding function or cofactor requirement
252930_at	At4g39010	Glycosyl Hydrolase Family 9 Protein	0.0473	0.0279	4	-3.45	1.04	2.59	Re-assembly of cell wall associated genes
251250_at	At3g62180	Pectin methylesterase, putative	0.0448	0.0200	4	-2.38	2.64	1.92	Re-assembly of cell wall associated genes
248859_at	At5g46660	CHP-Rich Zinc Finger Protein, Putative	0.0474	0.0308	4	-6.67	-1.41	-1.32	Transcription/splicing/RNA processing
257740_at	At3g27330	Zinc Finger (C3Hc4-Type Ring Finger) Family Protein	0.0475	0.0372	4	-6.25	1.26	1.78	Transcription/splicing/RNA processing
246790_at	At5g27610	Myb Family Transcription Factor	0.0453	0.0210	4	-5.26	-1.04	3.43	Transcription/splicing/RNA processing
246520_at	At5g15790	Zinc Finger (C3Hc4-Type Ring Finger) Family Protein	0.0475	0.0375	4	-4.00	1.74	1.27	Transcription/splicing/RNA processing
255359_at	At4g03950	Glucose-6-Phosphate/Phosphate Translocator, Putative	0.0390	0.0017	4	-9.89	2.14	1.72	Transporters
265955_at	At2g37280	Abc Transporter Family Protein	0.0465	0.0241	4	-3.57	-1.11	1.34	Transporters
265783_at	At2g07450	Gypsy-Like Retrotransposon Family (Athila)	0.0405	0.0048	4	-12.50	1.32	1.18	Transposable elements, viral and plasmid proteins

263389_at	At2g11680	Gypsy-Like Retrotransposon Family	0.0466	0.0257	4	-5.00	1.21	2.00	Transposable elements, viral and plasmid proteins
266251_s_at	At2g27540	Expressed Protein	0.0460	0.0227	4	-14.29	1.31	1.04	Unknown proteins
257111_x_at	At3g30450	Hypothetical Protein	0.0455	0.0216	4	-9.09	2.07	1.17	Unknown proteins
258442_at	At3g01015	Expressed Protein	0.0475	0.0365	4	-7.14	1.78	3.50	Unknown proteins
254138_at	At4g24950	Expressed Protein	0.0435	0.0101	4	-5.56	1.73	1.26	Unknown proteins
246365_at	At1g39750	Unknown Protein	0.0434	0.0062	4	-4.76	2.20	3.18	Unknown proteins
252741_at	At3g43280	Expressed Protein	0.0473	0.0279	4	-4.35	2.15	1.46	Unknown proteins
260715_at	At1g48200	Expressed Protein	0.0445	0.0143	4	-3.85	1.53	1.57	Unknown proteins
246811_at	At5g27170	Expressed Protein	0.0482	0.0407	4	-3.23	1.37	3.66	Unknown proteins
254908_at	At4g11200	Expressed Protein	0.0474	0.0298	4	-3.03	1.88	1.87	Unknown proteins
249756_at	At5g24313	Expressed Protein	0.0453	0.0204	4	-2.86	2.27	3.84	Unknown proteins
251129_at	At5g01150	Hypothetical Protein	0.0475	0.0463	4	-2.70	1.47	2.61	Unknown proteins
261414_at	At1g07795	Expressed protein	0.0475	0.0318	4	-1.92	1.24	3.24	Unknown proteins
254484_at	At4g20750	Unknown protein	0.0475	0.0355	4	-1.72	1.04	3.64	Unknown proteins
248941_s_at	At5g45460	Expressed protein	0.0457	0.0219	4	-1.72	1.08	4.22	Unknown proteins
257901_at	At3g28440	Unknown protein	0.0441	0.0092	4	-1.39	1.11	4.25	Unknown proteins

CLUSTER 5: Majority of the genes induced at 3hpt and significantly repressed (*q* -value ≤ 0.05; ≤ 2.5) at 6 hpt followed by induction at 12 hpt

246776_at	At5g27550	Kinesin Motor Protein-Related	0.0458	0.0225	5	-1.23	-8.33	-1.22	Cell Cycle/ DNA processing/chromatin regulation and cytoskeleton reorganization
250997_at	At5g02570	Histone H2B, Putative	0.0390	0.0028	5	1.44	-7.69	3.22	Cell Cycle/ DNA processing/chromatin regulation and cytoskeleton reorganization
257229_at	At3g16490	Calmodulin-Binding Family Protein	0.0495	0.0485	5	4.01	-8.62	2.48	Cellular communication/Signal transduction mechanism/transmembrane signal transduction
247776_at	At5g58700	Phospholipase C	0.0465	0.0247	5	1.41	-5.26	2.64	Cellular communication/Signal transduction mechanism/transmembrane signal transduction
250037_at	At5g18350	Disease Resistance Protein (TIR-NBS-LRR class), Putative	0.0469	0.0485	5	-1.15	-16.72	-2.91	Defence and virulence
261737_at	At1g47885	Disease Resistance Protein, Putative	0.0460	0.0228	5	2.81	-6.67	2.21	Defence and virulence
260052_at	At1g78220	14-3-3 Protein Gf14 Pi; Grf13	0.0414	0.0065	5	2.81	-6.67	1.41	Defence and virulence
263683_at	At1g26870	No Apical Meristem (Nam) Family Protein	0.0475	0.0350	5	1.44	-3.57	1.92	Development/storage proteins
261343_s_at	At3g43350	Helicase-Related	0.0445	0.0160	5	1.39	-10.00	3.17	Helicase activity
246734_at	At5g27680	Dead/Deah Box Helicase, Putative	0.0475	0.0334	5	1.94	-4.76	1.37	Helicase activity
265329_at	At2g18450	Succinate Dehydrogenase	0.0445	0.0178	5	1.61	-6.63	1.15	Primary /secondary metabolism
253149_at	At4g35650	Nad+ Isocitrate Dehydrogenase, Putative	0.0424	0.0072	5	2.22	-4.55	1.67	Primary /secondary metabolism
266751_at	At2g47020	Peptide Chain Release Factor, Putative	0.0445	0.0158	5	1.11	-4.17	1.68	Primary /secondary metabolism
253743_at	At4g28940	Nucleosidase-Related	0.0441	0.0118	5	1.36	-3.03	1.01	Primary /secondary metabolism
263061_at	At2g18190	Aaa-Type Atpase Family Protein	0.0494	0.0478	5	-1.28	-2.84	-1.63	Primary /secondary metabolism
261241_at	At1g32950	Subtilase Family Protein	0.0397	0.0034	5	1.40	-4.17	2.25	Protein fate
259631_at	At1g56410	Heat Shock Cognate 70 Kda Protein, Putative	0.0445	0.0155	5	1.20	-5.88	1.69	Protein folding/heat shock/chaperone activity
252259_at	At3g49460	60S Acidic Ribosomal Protein-Related	0.0466	0.0256	5	1.38	-6.17	2.83	Protein synthesis and translation
261435_at	At1g07620	Gtp-Binding Protein, Putative	0.0447	0.0183	5	3.52	-11.11	1.13	Proteins with binding function or cofactor requirement
247941_at	At5g57200	Enth Domain-Containing Protein	0.0461	0.0230	5	2.82	-11.11	2.00	Proteins with binding function or cofactor requirement
253853_at	At4g28130	Diacylglycerol Kinase Accessory Domain-Containing Protein	0.0495	0.0485	5	1.38	-3.41	1.21	Proteins with binding function or cofactor requirement
255517_at	At4g02290	Glycosyl Hydrolase Family 9 Protein	0.0447	0.0183	5	-1.04	-11.11	-2.38	Re-assembly of cell wall associated genes
257629_at	At3g26140	Glycosyl Hydrolase Family 5 Protein / Cellulase Family Protein	0.0482	0.0414	5	1.09	-4.73	2.57	Re-assembly of cell wall associated genes
263281_at	At2g14160	RNA Recognition Motif (Rrm)-Containing Protein	0.0445	0.0174	5	-1.93	-14.45	-2.86	Transcription/splicing/RNA processing
267091_at	At2g38185	Zinc Finger (C3Hc4-Type Ring Finger) Family Protein	0.0448	0.0197	5	1.56	-4.55	1.47	Transcription/splicing/RNA processing
252578_at	At3g45480	Zinc Finger (C3Hc4-Type Ring Finger) Family Protein	0.0475	0.0318	5	3.10	-4.35	2.27	Transcription/splicing/RNA processing
247035_at	At5g67110	Basic Helix-Loop-Helix (BHLH) Family Protein	0.0473	0.0282	5	-1.30	-4.00	-2.08	Transcription/splicing/RNA processing
263092_at	At2g16210	Transcriptional Factor B3 Family Protein	0.0475	0.0319	5	1.31	-3.87	1.58	Transcription/splicing/RNA processing
260263_at	At1g68480	Zinc Finger (C2H2 Type) Family Protein	0.0445	0.0175	5	1.58	-3.03	2.44	Transcription/splicing/RNA processing
259846_at	At1g72140	Proton-Dependent Oligopeptide Transport (Pot) Family Protein	0.0493	0.0466	5	1.23	-4.35	2.33	Transporters
264104_at	At2g13750	Cacta-Like Transposase Family (Ptta/En/Spm)	0.0492	0.0454	5	1.40	-12.50	-2.27	Transposable elements, viral and plasmid proteins
255188_at	At4g07340	Replication Protein-Related	0.0401	0.0043	5	2.34	-10.28	-1.28	Transposable elements, viral and plasmid proteins
256845_x_at	At3g31970	Gypsy-Like Retrotransposon Family	0.0476	0.0387	5	-1.15	-2.63	-1.41	Transposable elements, viral and plasmid proteins
261645_at	At1g27790	Hypothetical Protein	0.0494	0.0475	5	1.53	-14.30	1.09	Unknown proteins
249531_at	At5g38770	Hypothetical Protein	0.0447	0.0185	5	1.28	-8.76	2.30	Unknown proteins
252784_at	At3g43040	Unknown Protein	0.0475	0.0333	5	2.02	-8.33	1.66	Unknown proteins
267166_at	At2g37720	Expressed Protein	0.0441	0.0128	5	1.21	-4.76	1.56	Unknown proteins
254240_at	At4g23496	Expressed Protein	0.0478	0.0394	5	1.58	-3.73	1.20	Unknown proteins
255348_at	At4g03820	Expressed Protein	0.0474	0.0295	5	1.36	-3.70	1.58	Unknown proteins

252665_at	At3g44140	Expressed Protein	0.0449	0.0203	5	1.06	-3.13	1.09	Unknown proteins
266754_at	At2g46980	Expressed Protein	0.0425	0.0079	5	1.97	-3.03	2.18	Unknown proteins
264735_s_at	At1g62060	Expressed Protein	0.0475	0.0460	5	1.76	-2.96	2.68	Unknown proteins
254769_at	At4g13330	Expressed Protein	0.0474	0.0294	5	1.76	-2.86	1.32	Unknown proteins
253656_at	At4g30090	Expressed Protein	0.0486	0.0392	5	2.34	-2.86	3.01	Unknown proteins
**CLUSTER 6: Majority of the genes significantly repressed (*q* -value ≤ 0.05; ≤ 2.5) at 3hpt and significantly induced (q-value ≤ 0.05; ≥ 2.5) at 6 hpt followed by induction or repression at 12 hpt**									
252428_at	At3g47660	Regulator Of Chromosome Condensation (Rcc1) Protein	0.0466	0.0252	6	-3.49	3.14	1.27	Cell Cycle/ DNA processing/chromatin regulation and cytoskeleton reorganization
247192_at	At5g65360	Histone H3	0.0434	0.0093	6	-1.20	2.55	1.07	Cell Cycle/ DNA processing/chromatin regulation and cytoskeleton reorganization
262004_at	At1g64480	Calcineurin B-Like Protein 8 (Cbl8)	0.0474	0.0310	6	-4.87	1.74	-1.60	Cellular communication/Signal transduction mechanism/transmembrane signal transduction
248989_at	At5g45200	Disease resistance protein, TIR-NBS-LRRj	0.0431	0.0085	6	-1.49	6.39	2.12	Defence and virulence
266531_at	At2g16835	Water Channel Protein, Putative	0.0405	0.0046	6	-5.00	7.66	1.66	Development/storage proteins
252840_at	At5g32630	Putative Helicase	0.0445	0.0148	6	-10.00	1.06	-1.14	Helicase activity
253502_at	At4g31940	Cytochrome P450, Putative	0.0405	0.0046	6	-14.29	6.81	-2.17	Primary /secondary metabolism
262465_at	At1g50270	Pentatricopeptide (PPR) repeat-containing protein	0.0494	0.0468	6	-1.37	6.27	2.04	Primary /secondary metabolism
256496_at	At1g31510	F-Box Family Protein-Related	0.0397	0.0038	6	-5.56	5.22	-2.13	Protein fate
255149_at	At4g08150	Homeobox Protein Knotted-1 Like 1 (Knat1)	0.0447	0.0186	6	-9.21	1.73	-2.63	Proteins with binding function or cofactor requirement
250801_at	At5g04960	Pectinesterase Family Protein	0.0475	0.0331	6	-2.50	5.98	1.09	Re-assembly of cell wall associated genes
246798_at	At5g26930	Zinc Finger (Gata Type) Family Protein	0.0441	0.0113	6	-10.00	4.89	1.23	Transcription/splicing/RNA processing
256023_at	At1g58330	Transcription Factor-Related	0.0488	0.0442	6	-4.12	1.13	-1.08	Transcription/splicing/RNA processing
248188_at	At5g54070	Heat Shock Transcription Factor Family Protein	0.0468	0.0267	6	-2.94	5.72	1.36	Transcription/splicing/RNA processing
257811_at	At3g25280	Nitrate transporter, putative	0.0494	0.0479	6	-1.33	5.21	1.35	Transporters
255374_at	At4g03770	Gypsy-Like Retrotransposon Family (Athila)	0.0474	0.0288	6	-6.01	1.43	-3.52	Transposable elements, viral and plasmid proteins
262035_at	At1g37110	Copia-Like Retrotransposon Family	0.0475	0.0359	6	-3.33	5.46	1.80	Transposable elements, viral and plasmid proteins
263055_at	At2g04600	Expressed Protein	0.0401	0.0041	6	-20.00	4.48	-1.30	Unknown proteins
259566_at	At1g20520	Expressed Protein	0.0441	0.0116	6	-7.14	1.30	-2.50	Unknown proteins
250300_at	At5g11890	Expressed Protein	0.0475	0.0336	6	-5.88	1.82	-1.52	Unknown proteins
245420_at	At4g17410	Expressed Protein	0.0434	0.0068	6	-5.00	3.73	-1.04	Unknown proteins
256591_at	At3g31910	Expressed Protein	0.0493	0.0404	6	-4.17	2.95	-1.02	Unknown proteins
265745_at	At2g06640	Unknown Protein	0.0493	0.0464	6	-3.56	1.30	-1.42	Unknown proteins
253518_at	At4g31400	Expressed Protein	0.0475	0.0353	6	-3.37	1.24	-1.64	Unknown proteins
252889_at	At4g39380	Expressed Protein	0.0441	0.0107	6	-3.24	5.28	1.28	Unknown proteins
256436_at	At3g11150	Expressed Protein	0.0441	0.0113	6	-3.10	5.85	1.04	Unknown proteins
248598_at	At5g49370	Hypothetical Protein	0.0441	0.0100	6	-2.56	4.37	1.78	Unknown proteins
**CLUSTER 7: Majority of the genes induced at 3 and 6 hpt followed by significant repression (*q* -value ≤ 0.05; ≤ 2.5) at 12 hpt**									
250433_at	At5g10400	Histone H3	0.0464	0.0235	7	1.81	3.40	-11.11	Cell Cycle/ DNA processing/chromatin regulation and cytoskeleton reorganization
262141_s_at	At1g52460	Phospholipase	0.0475	0.0373	7	1.88	1.96	-12.64	Cellular communication/Signal transduction mechanism/transmembrane signal transduction
260061_at	At1g73690	Cell Division Protein Kinase, Putative	0.0475	0.0345	7	1.12	4.45	-5.26	Cellular communication/Signal transduction mechanism/transmembrane signal transduction
250443_at	At5g10520	Protein Kinase Family Protein	0.0441	0.0119	7	1.95	3.51	-4.55	Cellular communication/Signal transduction mechanism/transmembrane signal transduction
255757_at	At4g00460	Kinase Partner Protein-Like	0.0481	0.0306	7	2.25	3.31	-3.23	Cellular communication/Signal transduction mechanism/transmembrane signal transduction
246096_at	At5g20330	b-1,3-Glucanase; Bg4	0.0407	0.0055	7	2.29	5.53	-6.25	Defence and virulence
266562_at	At2g23970	Defense-Related Protein, Putative	0.0495	0.0485	7	1.66	4.23	-3.45	Defence and virulence
262156_at	At1g52680	Late Embryogenesis Abundant Protein-Related / Lea Protein-Related	0.0434	0.0097	7	1.17	1.40	-6.81	Development/storage proteins
252364_at	At3g48450	Nitrate-Responsive Noi Protein, Putative	0.0475	0.0373	7	1.47	3.37	-5.00	Development/storage proteins
249687_at	At5g36150	Pentacyclic Triterpene Synthase, Putative	0.0481	0.0403	7	-1.23	2.00	-9.09	Primary /secondary metabolism
257143_at	At3g20110	Cytochrome P450 Family Protein	0.0441	0.0120	7	1.34	4.49	-5.00	Primary /secondary metabolism
248704_at	At5g48450	Multi-Copper Oxidase Type I Family Protein	0.0487	0.0433	7	-2.04	-1.03	-5.00	Primary /secondary metabolism
247005_at	At5g67520	Adenylylsulfate kinase, putative	0.0474	0.0300	7	1.93	2.67	-1.45	Primary /secondary metabolism
248062_at	At5g55450	Protease Inhibitor	0.0445	0.0138	7	-1.24	1.00	-15.26	Protein fate
257051_at	At3g15270	Squamosa Promoter-Binding Protein-Like 5; Spl5	0.0448	0.0200	7	1.71	1.98	-5.56	Proteins with binding function or cofactor requirement
259324_at	At3g05310	Gtp-Binding Protein-Related	0.0475	0.0315	7	2.38	2.81	-4.35	Proteins with binding function or cofactor requirement
258241_at	At3g27650	Lob Domain Protein 25	0.0468	0.0266	7	1.38	2.11	-2.70	Proteins with binding function or cofactor requirement
257441_at	At2g04020	Gdsl-Motif Lipase/Hydrolase Family Protein	0.0441	0.0113	7	2.36	2.96	-5.39	Re-assembly of cell wall associated genes
257896_at	At3g16920	Glycoside Hydrolase Family 19 Protein	0.0445	0.0168	7	1.20	2.06	-3.78	Re-assembly of cell wall associated genes
256415_at	At3g11210	Gdsl-Motif Lipase/Hydrolase Family Protein	0.0447	0.0185	7	1.03	1.05	-2.63	Re-assembly of cell wall associated genes
259810_at	At1g49810	Na+/H+ Antiporter, Putative	0.0390	0.0013	7	1.05	3.68	-3.85	Transporters

255156_at	At4g07780	Gypsy-Like Retrotransposon Family (Athila)	0.0441	0.0106	7	1.61	2.42	-18.75	Transposable elements, viral and plasmid proteins
263508_s_at	At2g07685	Gypsy-Like Retrotransposon Family (Athila)	0.0482	0.0411	7	1.58	1.80	-7.14	Transposable elements, viral and plasmid proteins
254366_s_at	At4g22040	Retrovirus-related polyprotein	0.0465	0.0241	7	2.22	4.03	-1.61	Transposable elements, viral and plasmid proteins
265746_at	At2g06630	Hypothetical Protein	0.0445	0.0142	7	1.19	1.22	-40.97	Unknown proteins
259870_at	At1g76780	Expressed Protein	0.0441	0.0106	7	-2.27	1.67	-16.67	Unknown proteins
251012_at	At5g02580	Expressed Protein	0.0441	0.0091	7	1.62	5.73	-11.11	Unknown proteins
254825_at	At4g12630	Unknown Protein	0.0390	0.0030	7	1.95	1.64	-8.62	Unknown proteins
250945_at	At5g03400	Hypothetical Protein	0.0478	0.0394	7	1.99	1.66	-6.76	Unknown proteins
246795_at	At5g27020	Hypothetical Protein	0.0475	0.0332	7	1.66	1.41	-6.61	Unknown proteins
267479_at	At2g02690	Expressed Protein	0.0401	0.0044	7	1.05	1.61	-6.24	Unknown proteins
254311_at	At4g22440	Hypothetical Protein	0.0466	0.0256	7	2.00	2.22	-5.56	Unknown proteins
263738_at	At1g60060	Expressed Protein	0.0441	0.0105	7	1.11	1.25	-5.56	Unknown proteins
247883_at	At5g57790	Expressed Protein	0.0390	0.0032	7	1.82	1.88	-3.73	Unknown proteins
263498_at	At2g42610	Expressed Protein	0.0447	0.0184	7	1.34	1.98	-3.70	Unknown proteins
248282_at	At5g52900	Expressed Protein	0.0475	0.0371	7	1.05	1.26	-3.45	Unknown proteins
248904_at	At5g46295	Expressed Protein	0.0445	0.0169	7	1.53	1.94	-3.08	Unknown proteins
246711_at	At5g28190	Hypothetical Protein	0.0445	0.0133	7	1.72	3.41	-2.61	Unknown proteins
254295_at	At4g23080	Expressed Protein	0.0417	0.0053	7	1.02	2.97	-2.50	Unknown proteins
253050_at	At4g37450	Arabinogalactan-Protein (Agp18)	0.0454	0.0215	7	1.49	1.40	-6.12	Structural proteins
**CLUSTER 8: Majority of the genes repressed at 3 hpt followed by significant induction (*q* -value ≤ 0.05; ≥ 2.5) at 6 hpt and repression at 12 hpt**									
249729_at	At5g24410	Glucosamine/Galactosamine-6-Phosphate Isomerase-Related	0.0445	0.0153	8	-5.24	2.42	-5.96	Carbohydrate/soluble sugar/startch/aminoacid metabolism
247159_at	At5g65800	1-Aminocyclopropane-1-Carboxylate Synthase, Putative	0.0445	0.0142	8	-2.91	2.38	-4.04	Carbohydrate/soluble sugar/startch/aminoacid metabolism
260699_at	At1g32320	MAP kinase, putative	0.0475	0.0345	8	-2.22	3.59	-1.05	Cellular communication/Signal transduction mechanism/transmembrane signal transduction
264719_at	At1g70110	Lectin protein kinase family protein	0.0466	0.0251	8	-1.30	4.62	-1.75	Cellular communication/Signal transduction mechanism/transmembrane signal transduction
257261_s_at	At3g21930	Receptor-Like Protein Kinase-Related	0.0454	0.0214	8	-1.67	4.12	-3.03	Cellular communication/Signal transduction mechanism/transmembrane signal transduction
249440_at	At5g40030	Protein kinase PK1- like	0.0441	0.0104	8	-1.37	6.90	-2.22	Cellular communication/Signal transduction mechanism/transmembrane signal transduction
265682_at	At2g24390	Avirulence induced gene (AIG) protein, related	0.0424	0.0072	8	-1.92	9.78	1.19	Defence and virulence
254582_at	At4g19470	Disease resistance protein, related	0.0475	0.0369	8	-1.82	5.13	-2.44	Defence and virulence
262752_at	At1g16330	Cyclin family protein	0.0441	0.0119	8	-1.59	4.33	-1.41	Development/storage proteins
258082_at	At3g25905	Cle27, Putative	0.0450	0.0322	8	-4.10	3.22	-4.82	Development/storage proteins
248636_at	At5g49080	Extensin-like family protein	0.0390	0.0025	8	-1.09	4.64	-2.08	Development/storage proteins
248112_at	At5g55350	Wax Synthase-Like Protein	0.0441	0.0111	8	-1.96	7.13	-3.85	Development/storage proteins
259388_at	At1g13420	Sulfotransferase Family Protein	0.0494	0.0474	8	-4.42	3.70	-3.30	Primary /secondary metabolism
261529_at	At1g14430	glyoxal oxidase-related	0.0434	0.0089	8	-1.52	10.14	1.50	Primary /secondary metabolism
245650_at	At1g24735	Caffeoyl-CoA 3-O-methyltransferase, putative	0.0475	0.0321	8	-1.18	2.65	-1.54	Primary /secondary metabolism
256211_at	At1g50960	Gibberellin 20-oxidase-related	0.0494	0.0469	8	-1.28	8.86	-1.43	Primary /secondary metabolism
266308_at	At2g27010	Cytochrome P450 family protein	0.0475	0.0378	8	-1.16	12.86	-1.18	Primary /secondary metabolism
267416_at	At2g34980	Phosphatidylinositol-glycan synthase, putative	0.0441	0.0124	8	-1.14	5.72	-1.75	Primary /secondary metabolism
254513_at	At4g20240	Cytochrome P450, Putative (Metabolism)	0.0488	0.0443	8	-1.72	2.73	-2.70	Primary /secondary metabolism
249773_at	At5g24140	Squalene monooxygenase 2, SM 2	0.0434	0.0096	8	-1.43	9.58	1.80	Primary /secondary metabolism
246970_at	At5g24900	Cytochrome P450 Family Protein	0.0407	0.0056	8	-2.78	7.01	-1.22	Primary /secondary metabolism
248104_at	At5g55250	S-adenosyl-L-methionine, putative	0.0465	0.0243	8	-1.43	3.57	-1.27	Primary /secondary metabolism
267056_at	At2g32470	F-box family protein, related	0.0407	0.0056	8	1.06	8.98	1.34	Protein fate
256120_at	At1g18130	TRNA Synthetase-Related -Related	0.0475	0.0252	8	-2.72	8.78	-2.99	RNA metabolism
259814_at	At1g49900	Zinc finger family protein, C2H2 type	0.0445	0.0180	8	-1.09	5.58	-2.44	Transcription/splicing/RNA processing
265124_at	At1g55430	Zinc finger protein-like CHP type, putative	0.0448	0.0200	8	-1.82	6.99	-1.37	Transcription/splicing/RNA processing
257414_at	At1g62110	Mitochondrial transcription factor mTERF family protein	0.0475	0.0380	8	-2.33	4.56	-1.56	Transcription/splicing/RNA processing
263797_at	At2g24570	WRKY family transcription factor	0.0390	0.0029	8	1.19	3.62	1.08	Transcription/splicing/RNA processing
246531_at	At5g15800	MADS box protein, AGL2	0.0448	0.0193	8	-1.39	6.00	-1.82	Transcription/splicing/RNA processing
249884_at	At5g22910	Na+/H+ Antiporter-Like Protein	0.0475	0.0316	8	-3.81	3.65	-3.64	Transporters
263746_at	At2g21460	Copia-Like Retrotransposon Family	0.0448	0.0198	8	-2.95	8.04	-2.09	Transposable elements, viral and plasmid proteins
266586_s_at	At4g08060	Cacta-Like Transposase Family (Tnp1/En/Spm)	0.0388	0.0005	8	-5.00	8.51	-2.22	Transposable elements, viral and plasmid proteins
259489_at	At1g15790	Expressed protein	0.0453	0.0201	8	-1.61	2.89	-1.39	Unknown proteins
245646_s_at	At1g25025	Expressed Protein	0.0409	0.0176	8	-5.26	7.78	-1.59	Unknown proteins
263672_at	At2g04820	Unknown Protein	0.0405	0.0048	8	-3.70	8.74	-2.17	Unknown proteins
263337_at	At2g04990	Unknown Protein	0.0471	0.0339	8	-2.91	2.80	-3.24	Unknown proteins

265524_at	At2g06180	Unknown protein	0.0409	0.0178	8	-2.38	5.94	-1.27	Unknown proteins
266894_at	At2g26050	Expressed Protein	0.0466	0.0330	8	-2.93	4.17	-4.14	Unknown proteins
256592_at	At3g31915	Expressed Protein	0.0466	0.0327	8	-2.14	6.84	-4.57	Unknown proteins
255167_at	At4g07920	Unknown Protein	0.0457	0.0212	8	-1.51	6.74	-3.40	Unknown proteins
248430_at	At5g51800	Expressed protein	0.0477	0.0491	8	-1.82	6.76	-1.72	Unknown proteins

CLUSTER 9: Majority of the genes significantly induced (*q* -value ≤ 0.05; ≥ 2.5) at 3 hpt followed by induction or repression at 6 hpt and significant repression (q-value ≤ 0.05; ≤ 2.5) at 12 hpt

260161_at	At1g79860	Kinase Partner Protein-Like	0.0474	0.0219	9	4.92	2.32	-4.17	Cellular communication/Signal transduction mechanism/transmembrane signal transduction
248420_at	At5g51560	Leucine-Rich Repeat Transmembrane Protein Kinase, Putative	0.0466	0.0254	9	1.86	1.00	-2.70	Cellular communication/Signal transduction mechanism/transmembrane signal transduction
256487_at	At1g31540	Disease resistance protein, TIR-NBS-LRR class, putative	0.0475	0.0314	9	2.97	1.37	-1.96	Defence and virulence
253353_at	At4g33730	Pathogenesis-Related Protein, Putative	0.0475	0.0376	9	1.45	-2.33	-7.69	Defence and virulence
260346_at	At1g69320	Cle10, Putative	0.0494	0.0439	9	2.85	1.33	-2.63	Development/storage proteins
267493_at	At2g30400	Ovate Family Protein	0.0479	0.0399	9	1.08	-1.56	-9.09	Development/storage proteins
248676_at	At5g48850	male sterility MS5 family protein	0.0475	0.0258	9	2.59	2.26	-1.45	Development/storage proteins
245465_at	At4g16590	Glucosyltransferase, related	0.0445	0.0181	9	3.15	2.23	-1.37	Matrix polymers
250783_at	At5g05260	Cytochrome P450 (Metabolism)	0.0407	0.0055	9	3.17	1.03	-2.55	Primary /secondary metabolism
246009_at	At5g08335	Isoprenylcysteine carboxyl methyltransferase family protein	0.0475	0.0380	9	2.80	1.59	-1.59	Primary /secondary metabolism
267589_at	At2g42050	Peptides precurssor	0.0441	0.0120	9	3.11	1.82	-2.00	Proteins with binding function or cofactor requirement
255321_at	At4g04260	BAH domain containing protein	0.0475	0.0350	9	3.62	2.34	-1.11	Proteins with binding function or cofactor requirement
264898_at	At1g23205	Invertase/Pectin Methylesterase Inhibitor Family Protein	0.0441	0.0122	9	2.86	1.83	-2.86	Re-assembly of cell wall associated genes
258147_at	At3g18070	Glycosyl Hydrolase Family 1 Protein	0.0438	0.0127	9	3.06	1.25	-9.70	Re-assembly of cell wall associated genes
267167_at	At2g37740	Zinc Finger (C2H2 Type) Family Protein	0.0474	0.0302	9	2.79	1.54	-7.25	Transcription/splicing/RNA processing
252128_at	At3g50870	Zinc finger family protein, GATA type	0.0434	0.0094	9	3.00	2.04	-1.54	Transcription/splicing/RNA processing
246338_s_at	At3g44930	Cation/Hydrogen Exchanger, Putative (Chx10)	0.0466	0.0253	9	4.13	2.42	-3.45	Transporters
255316_at	At4g04170	Cacta-Like Transposase Family (Ptta/En/Spm)	0.0390	0.0015	9	1.95	-1.47	-14.29	Transposable elements, viral and plasmid proteins
255215_at	At4g07660	Gypsy-Like Retrotransposon Family (Athila)	0.0474	0.0311	9	1.35	-2.22	-9.09	Transposable elements, viral and plasmid proteins
266733_at	At2g03280	Expressed Protein	0.0390	0.0032	9	2.25	1.31	-2.70	Unknown proteins
258955_s_at	At3g01450	Expressed Protein	0.0480	0.0302	9	4.48	1.86	-6.67	Unknown proteins
255398_at	At4g03740	Expressed Protein	0.0493	0.0413	9	4.90	1.53	-5.96	Unknown proteins
254949_at	At4g11020	Expressed Protein	0.0441	0.0126	9	5.47	2.22	-4.00	Unknown proteins
264461_s_at	At4g15050	Expressed Protein	0.0466	0.0261	9	2.49	1.38	-9.09	Unknown proteins
253827_at	At4g28085	Expressed protein	0.0441	0.0122	9	2.77	1.22	-1.05	Unknown proteins
249099_at	At5g43550	Expressed Protein	0.0493	0.0456	9	5.14	1.99	-3.17	Unknown proteins
247646_at	At5g59990	Expressed Protein	0.0475	0.0354	9	2.97	1.19	-3.03	Unknown proteins

CLUSTER 10: Majority of the genes significantly induced (*q* -value ≤ 0.05; ≥ 2.5) at 3 hpt followed by repression at 6 hpt and significant repression (q-value ≤ 0.05; ≤ 2.5) at 12 hpt

245942_at	At5g19490	Repressor Protein-Related	0.0493	0.0464	10	2.26	-1.01	-4.80	Cell Cycle/ DNA processing/chromatin regulation and cytoskeleton reorganization
245655_at	At1g56530	Hydroxyproline-Rich Glycoprotein Family Protein	0.0434	0.0091	10	7.94	-2.50	-6.67	Cellular communication/Signal transduction mechanism/transmembrane signal transduction
264369_at	At1g70430	Ste-20 Kinase Spak, Putative	0.0495	0.0485	10	4.37	-1.92	-5.88	Cellular communication/Signal transduction mechanism/transmembrane signal transduction
247962_at	At5g56580	Mitogen-Activated Protein Kinase Kinase, Putative	0.0471	0.0277	10	2.78	-1.27	-3.45	Cellular communication/Signal transduction mechanism/transmembrane signal transduction
267041_at	At2g34315	Disease Resistance Protein-Related	0.0445	0.0159	10	1.27	-1.32	-4.17	Defence and virulence
266286_at	At2g29170	Dehydrogenase/Reductase (Sdr) Family Protein	0.0445	0.0180	10	1.57	-1.37	-4.00	Primary /secondary metabolism
266875_at	At2g44800	Oxidoreductase, 2Og-Fe(Ii) Oxygenase Family Protein	0.0445	0.0172	10	3.93	-1.10	-5.30	Primary /secondary metabolism
260891_at	At1g29080	Cysteine Protease, Spcp1	0.0475	0.0348	10	10.06	2.38	-5.00	Protein fate
258741_at	At3g05790	Lon protease, putative	0.0485	0.0426	10	3.67	1.03	-2.17	Protein fate
258407_at	At3g17620	F-Box Family Protein	0.0475	0.0335	10	1.72	-1.33	-3.23	Protein fate
254875_at	At4g11590	F-Box Family Protein	0.0448	0.0191	10	2.43	-4.95	-10.60	Protein fate
266712_at	At2g46750	Fad-Binding Domain-Containing Protein	0.0414	0.0065	10	2.36	-1.21	-4.66	Proteins with binding function or cofactor requirement
266877_at	At2g44570	Glycosyl hydrolase family 9 protein	0.0397	0.0037	10	5.21	1.79	-1.52	Re-assembly of cell wall associated genes
254959_at	At4g10955	Lipase Class 3 Family Protein	0.0445	0.0137	10	4.74	-2.08	-5.56	Re-assembly of cell wall associated genes
248275_at	At5g53520	Oligopeptide transporter OPT family protein	0.0488	0.0444	10	3.63	1.36	-1.11	Transporters
256175_at	At1g51670	Expressed Protein	0.0475	0.0374	10	10.29	1.53	-3.13	Unknown proteins
262072_at	At1g59590	Expressed protein	0.0445	0.0146	10	3.43	1.33	-1.22	Unknown proteins
260198_at	At1g67635	Expressed Protein	0.0496	0.0493	10	2.02	-2.78	-5.66	Unknown proteins
267491_at	At2g19140	Unknown Protein	0.0488	0.0312	10	3.27	-1.39	-3.33	Unknown proteins
255029_x_at	At4g09470	Expressed Protein	0.0465	0.0243	10	1.43	-1.60	-3.65	Unknown proteins

CLUSTER 11: Majority of the genes repressed at 3 and 6 hpt followed by significant induction (*q* -value ≤ 0.05; ≥ 2.5) at 12 hpt

263751_at	At2g21300	Kinesin Motor Family Protein	0.0390	0.0031	11	-1.20	-2.94	5.21	Cell Cycle/ DNA processing/chromatin regulation and cytoskeleton reorganization
256587_at	At3g28780	Histone H4 protein -like	0.0466	0.0257	11	-1.35	-1.61	2.52	Cell Cycle/ DNA processing/chromatin regulation and cytoskeleton reorganization
264991_s_at	At3g43400	Phagocytosis and cell motility protein ELMO1, related	0.0441	0.0132	11	-1.09	1.00	5.99	Cell Cycle/ DNA processing/chromatin regulation and cytoskeleton reorganization
261308_at	At1g48480	Leucine-Rich Repeat Transmembrane Protein Kinase, Putative	0.0448	0.0200	11	-2.50	-2.70	1.51	Cellular communication/Signal transduction mechanism/transmembrane signal transduction
257825_at	At3g26700	Protein kinase, putative	0.0424	0.0072	11	-1.45	-1.19	3.19	Cellular communication/Signal transduction mechanism/transmembrane signal transduction
261703_at	At1g32770	No Apical Meristem (Nam) Family Protein	0.0476	0.0387	11	-3.23	-1.11	1.97	Development/storage proteins
267198_at	At2g30810	Gibberellin-regulated family protein	0.0467	0.0264	11	-1.22	1.10	26.53	Development/storage proteins
251119_at	At3g63510	Nitrogen regulation family protein	0.0499	0.0499	11	-1.19	-2.27	4.16	Development/storage proteins
249702_at	At5g35570	Auxin-independent growth promoter protein, Axi 1	0.0489	0.0445	11	-1.52	-1.67	2.72	Development/storage proteins
250138_at	At5g14610	DEAD box RNA helicase, putative	0.0492	0.0453	11	-1.15	-1.75	3.10	Helicase activity
260475_at	At1g11080	Serine carboxypeptidase S10 family protein	0.0398	0.0040	11	-1.22	-1.30	6.75	Primary /secondary metabolism
259696_at	At1g63150	Pentatricopeptide (Ppr) Repeat-Containing Protein	0.0445	0.0136	11	-3.85	-1.82	5.55	Primary /secondary metabolism
263068_at	At2g17580	Polynucleotide Adenylyltransferase Family Protein	0.0447	0.0183	11	-3.03	-1.61	3.68	Primary /secondary metabolism
267434_at	At2g26260	3-Beta Hydroxysteroid Dehydrogenase/Isomerase Family Protein	0.0465	0.0248	11	-3.38	-4.96	1.45	Primary /secondary metabolism
258962_at	At3g10570	Cytochrome P450, putative	0.0401	0.0044	11	-1.20	-1.01	6.66	Primary /secondary metabolism
258174_at	At3g21470	Pentatricopeptide (PPR) repeat-containing protein	0.0494	0.0428	11	-1.52	-1.75	3.58	Primary /secondary metabolism
248692_s_at	At4g15070	DC1 domain containing protein	0.0466	0.0258	11	-2.38	-1.04	9.94	Proteins with binding function or cofactor requirement
252756_s_at	At3g43550	Gdsl-Motif Lipase, Putative	0.0405	0.0050	11	-1.06	-4.17	14.58	Re-assembly of cell wall associated genes
251748_at	At3g55680	Invertase/Pectin Methylesterase Inhibitor Family Protein	0.0475	0.0320	11	-5.85	-2.19	3.96	Re-assembly of cell wall associated genes
253226_at	At4g35010	Glycosyl Hydrolase Family 35 Protein	0.0465	0.0237	11	-3.12	-2.75	1.10	Re-assembly of cell wall associated genes
245849_at	At5g13520	Hydrolase-Like Protein	0.0475	0.0376	11	-1.56	-3.13	4.35	Re-assembly of cell wall associated genes
259595_at	At1g28050	Zinc finger B-box type family protein	0.0492	0.0455	11	-1.02	-2.08	3.83	Transcription/splicing/RNA processing
246274_at	At4g36620	Zinc Finger (Gata Type) Family Protein	0.0475	0.0372	11	-3.81	-5.85	2.10	Transcription/splicing/RNA processing
245179_at	At5g12400	PHD finger transcription factor, putative	0.0465	0.0238	11	-1.14	-1.69	3.60	Transcription/splicing/RNA processing
250051_at	At5g17800	MYB family transcription factor, MYB56	0.0424	0.0071	11	-1.04	-2.04	2.97	Transcription/splicing/RNA processing
248596_at	At5g49330	MYB family transcription factor	0.0401	0.0043	11	-1.43	-1.92	6.03	Transcription/splicing/RNA processing
261455_at	At1g21070	Transporter-related	0.0449	0.0203	11	-2.00	-2.00	4.04	Transporters
262031_x_at	At1g37160	Gypsy-Like Retrotransposon Family (Athila)	0.0475	0.0351	11	-2.38	-3.85	1.70	Transposable elements, viral and plasmid proteins
264932_at	At1g61240	Expressed protein	0.0475	0.0268	11	-1.54	-1.41	4.04	Unknown proteins
255853_at	At1g67020	Hypothetical Protein	0.0477	0.0477	11	-1.89	-3.03	7.37	Unknown proteins
263815_at	At2g10020	Expressed protein	0.0457	0.0208	11	-1.14	-1.79	2.93	Unknown proteins
265491_s_at	At2g15650	Unknown protein	0.0407	0.0052	11	-2.27	-1.67	4.05	Unknown proteins
259195_at	At3g01730	Expressed protein	0.0446	0.0185	11	-2.22	1.62	9.32	Unknown proteins
257308_at	At3g28120	Expressed Protein	0.0482	0.0380	11	-3.49	-2.60	6.37	Unknown proteins
252778_at	At3g42980	Hypothetical protein	0.0474	0.0300	11	-1.69	-2.44	5.10	Unknown proteins
246341_x_at	At3g43150	Hypothetical Protein	0.0474	0.0292	11	-2.13	-3.45	1.74	Unknown proteins
252196_at	At3g50200	Expressed Protein	0.0494	0.0474	11	-3.68	-1.67	2.00	Unknown proteins
248706_at	At5g48530	Expressed Protein	0.0476	0.0388	11	-3.24	-2.88	3.99	Unknown proteins

[a]Probe set ID represents Affymeterix probe set number.

[b]AGI represents *Arabidopsis* Genome Initiative (AGI) locus identifier corresponding to each gene represented on the array.

[c] *q* -value ≤ 0.05 (5% False discovery rate) was used to determine genes differentially expressed in protoplasts transfected with an PPV infectious clone, pPPV-SK68 relative to its non-infectious mutant, pPPV-SK68Δ.

[d] *p* -values (*p* ≤ 0.05) from the ANOVA were used to calculate *q* -value after adjusing the values using Benjamini and Hochberg [21] multiple testing correction.

[e] Hierarchical clustering and changes in gene expression of 411 significantly (*q* -value ≤ 0.05) differentially expressed *Arabidopsis* genes at 3, 6 and 12 hours post transfection (hpt).

[f] Calculation of fold changes was defined in Methods.

[g] hpt, hours post transfection.

[h] Determined followingt the method of the *Arabidopsis* MIPS (Munich Information Centre for Protein Sequences) fucntional classification scheme.
