# Supplementary material for: Altered gene expression changes in Arabidopsis leaf tissues and protoplasts in response to Plum pox virus infection
Source: BMC Genomics. 2008 Jul 9;9:325. doi: 10.1186/1471-2164-9-325 (PMC2478689; doi:10.1186/1471-2164-9-325)
Supplement: Additional file 9 — Supplemental Table 7. Identification of Prunus persica orthologs to Arabidopsis genes induced by PPV infection in the leaf tissues at 17 days post inoculation. [file 1471-2164-9-325-S9.pdf]

Supplemental Table 7. Identification of *Prunus persica* orthologs to *Arabidopsis* genes differentially regulated by PPV infection in the leaf tissues at 17 days post inoculation.

AGI[a] Locus	ATH1[b] gene annotation	Functional groups[c]	Annotation of Peach Orthologs[d]	PPL[e] EST identifier	HSP[f]	E-value	Identity[g]	GenBank Identifier[h]
**I. *Arabidopsis* gene sequences significantly induced by PPV in the infected leaf tissues show sequence similarity to *Prunus persica* uniESTs**								
**A. Defence and virulence**								
AT2G14580	Pathogenesis-Related Protein	Defence and virulence	Unknown Protein	PPL-01-E12E12	108	5.00E-48	68	DN552939
			Unknown Protein	PPL-22-H11	129	7.00E-58	66	DN556049
			Tubulin Alpha-2/Alpha-4 Chain	PPL-19-E07	158	1.00E-57	65	DN555644
			Unknown Protein	PPL-16-F04	114	1.00E-52	70	DN556381
			Unknown Protein	PPL-12-F08	161	3.00E-67	68	DN554655
			Unknown Protein	PPL-14-C07	158	2.00E-66	65	DN554801
			Water Channel - Like Protein	PPL-11-G06	158	1.00E-57	65	DN554576
			Pathogenesis-Related Protein 1	PPL-05-D08	103	2.00E-23	70	DN553262
			Unknown Protein	PPL-08-_T3__F05	132	2.00E-60	67	DN554179
			Flavonoid 3'-Hydroxylase - Like Protein	PPL-06-C03	108	4.00E-32	68	DN553869
AT5G63530	Copper Chaperone (Cch)-Related	Defence and virulence	Unknown Protein	PPL-08-_T3__C10	135	6.00E-33	46	DN554149
AT5G13000	Glycosyl Transferase Family 48 Protein	Defence and virulence	Unknown Protein	PPL-08-_T3__D12 ; PPL-17-A08	123	2.00E-29	98	DN554162; DN555510
AT2G31960	Glycosyl Transferase Family 48 Protein	Defence and virulence	Unknown Protein	PPL-08-_T3__D12 ; PPL-17-A08	122	2.00E-29	98	DN554162; DN555510
AT4G35090	Catalase 2	Defence and virulence	Unknown Protein	PPL-09-D02	403	E-113	87	DN554240
			Unknown Protein	PPL-19-G10	126	2.00E-30	87	DN555670
			Pectinesterase Like Protein	PPL-18-G11	369	E-103	88	DN555854
			Unknown Protein	PPL-14-F10 ; PPL-15-D10	366	E-102	84; 87	DN554833; DN554896
			Unknown Protein	PPL-13-E04	404	E-114	86	DN554727
AT1G20630	Catalase 1	Defence and virulence	Unknown Protein	PPL-09-D02	391	E-110	84	DN554240
			Unknown Protein	PPL-19-G10	115	3.00E-27	72	DN555670
			Pectinesterase Like Protein	PPL-18-G11	357	E-100	86	DN555854
			Unknown Protein	PPL-14-F10	349	2.00E-97	79	DN554833
			Unknown Protein	PPL-15-D10	351	6.00E-98	83	DN554896
			Unknown Protein	PPL-13-E04	384	E-108	83	DN554727
AT5G39190	Germin-Like Protein (Ger2)	Defence and virulence	Unknown Protein	PPL-09-E12	104	1.00E-23	47	DN554260
AT1G75800	Pathogenesis-Related Thaumatin Family Protein	Defence and virulence	Unknown Protein	PPL-11-E12	101	2.00E-25	50	DN554559
			Unknown Protein	PPL-23-C03	156	7.00E-44	58	DN556075
			S-Adenosylmethionine Synthase 2	PPL-23-C01	156	5.00E-42	58	DN556073
AT4G38670	Pathogenesis-Related Thaumatin Family Protein	Defence and virulence	Unknown Protein	PPL-11-E12	101	7.00E-24	49	DN554559
AT5G02500	Heat Shock Cognate 70 Kda Protein	Defence and virulence	Unknown Protein	PPL-11-F07	116	5.00E-36	87	DN554565
			Pectinesterase Like Protein	PPL-25-G11	183	3.00E-51	86	DN556311
			Unknown Protein	PPL-23-B12	125	7.00E-39	86	DN556072
			Unknown Protein	PPL-19-B05	157	1.00E-43	85	DN555607
			Unknown Protein	PPL-13-D02	229	3.00E-61	89	DN554713
AT3G09440	Heat Shock Cognate 70 Kda Protein	Defence and virulence	Unknown Protein	PPL-11-F07	105	2.00E-32	84	DN554565
			Pectinesterase Like Protein	PPL-25-G11	182	3.00E-47	70	DN556311
			Unknown Protein	PPL-23-B12	110	5.00E-34	84	DN556072
			Unknown Protein	PPL-19-B05	158	4.00E-40	68	DN555607
			Unknown Protein	PPL-13-D02	229	6.00E-65	75	DN554713
AT3G12580	Heat Shock Cognate 70 Kda Protein	Defence and virulence	Unknown Protein	PPL-11-F07	110	2.00E-25	68	DN554565
			Pectinesterase Like Protein	PPL-25-G11	199	2.00E-52	83	DN556311
			Unknown Protein	PPL-23-B12	116	2.00E-27	70	DN556072
			Unknown Protein	PPL-19-B05	175	3.00E-45	83	DN555607
			Unknown Protein	PPL-13-D02	244	7.00E-66	85	DN554713
AT2G14580	Pathogenesis-Related Protein	Defence and virulence	Unknown Protein	PPL-13-B05 ; PPL-17-E01	158	2.00E-66	65	DN554697; DN555547
AT4G38660	Thaumatin, Putative	Defence and virulence	S-Adenosylmethionine Synthase 2	PPL-23-C01	108	2.00E-45	55	DN556073
AT3G14650	Cytochrome P450	Defence and virulence	Unknown Protein	PPL-14-B12	102	5.00E-23	47	DN554794
AT2G16890	Udp-Glucoronosyl/Udp-Glucosyl Transferase	Defence and virulence	Unknown Protein	PPL-15-A10	130	2.00E-31	52	DN554865
AT2G31790	Udp-Glucoronosyl/Udp-Glucosyl Transferase	Defence and virulence	Unknown Protein	PPL-16-C09	134	4.00E-49	65	DN556353
AT1G05530	Udp-Glucoronosyl/Udp-Glucosyl Transferase	Defence and virulence	Unknown Protein	PPL-16-C09	112	7.00E-35	55	DN556353
AT4G38270	Glycosyl Transferase Family 8 Protein	Defence and virulence	Unknown Protein	PPL-23-F12	188	7.00E-49	64	DN556118
AT3G08910	Dnaj Heat Shock Protein	Defence and virulence	Unknown Protein	PPL-23-H12	142	1.00E-45	57	DN556142
AT2G20560	Dnaj Heat Shock Protein	Defence and virulence	Unknown Protein	PPL-23-H12	135	9.00E-43	56	DN556142
AT1G49860	Glutathione S-Transferase	Defence and virulence	Calcium-Dependent Protein Kinase (Cdpk6)	PPL-20-A06	140	1.00E-34	44	DN555689
AT1G78360	Glutathione S-Transferase	Defence and virulence	Water Channel - Like Protein	PPL-20-D09	133	2.00E-32	67	DN555725
			Chlorophyll A/B-Binding Protein	PPL-21-B10	138	9.00E-34	68	DN555888
AT5G18820	Chaperonin, Putative	Defence and virulence	Nucleoid Dna-Binding-Like Protein	PPL-24-C09	184	4.00E-47	68	DN556174
AT3G13470	Chaperonin, Putative	Defence and virulence	Nucleoid Dna-Binding-Like Protein	PPL-24-C09	109	2.00E-35	56	DN556174
AT3G44110	DNA J Heat Shock Protein, Putative	Defence and virulence	Unknown Protein	PPL-03-E10	125	6.00E-30	74	DN553106
AT2G40280	Dehydration-Responsive Family Protein	Defence and virulence	Unknown Protein	PPL-20-H05	100	1.00E-39	69	DN555767
AT5G62640	Proline-Rich Family Protein	Defence and virulence	Translationally Controlled Tumor Protein	PPL-18-H01	161	8.00E-43	64	DN555856
**B. Cellular communication/Signal transduction mechanism/transmembrane signal transduction**								
AT4G00720	Shaggy-Related Protein Kinase	Cellular communication/Signal transduction mechanism/transmembrane signal transductio	Unknown Protein	PPL-03-F10	262	4.00E-71	93	DN553117
AT1G09840	Shaggy-Related Protein Kinase	Cellular communication/Signal transduction mechanism/transmembrane signal transductio	Unknown Protein	PPL-03-F10	252	4.00E-68	88	DN553117
AT1G07430	Protein Phosphatase 2C, Putative	Cellular communication/Signal transduction mechanism/transmembrane signal transductio	Unknown Protein	PPL-21-B03	185	5.00E-48	62	DN555881
AT5G10900	Calcineurin-Like Phosphoesterase Family Protein	Cellular communication/Signal transduction mechanism/transmembrane signal transductio	Unknown Protein	PPL-06-H02	111	1.00E-33	67	DN553999
AT3G22800	Leucine-Rich Repeat Family Protein	Cellular communication/Signal transduction mechanism/transmembrane signal transductio	Unknown Protein	PPL-10-E11 ; PPL-15-F08	113	2.00E-26	65	DN554465; DN554917
AT5G11850	Protein Kinase Family Protein	Cellular communication/Signal transduction mechanism/transmembrane signal transductio	Endoxyloglucan Transferase-Like Protein	PPL-12-B03	303	2.00E-83	90	DN554604
AT4G23050			Endoxyloglucan Transferase-Like Protein	PPL-12-B03	175	5.00E-45	53	DN554604
AT2G17700			Endoxyloglucan Transferase-Like Protein	PPL-12-B03	116	2.00E-27	40	DN554604
AT4G15530	Pyruvate,Orthophosphate Dikinase	Cellular communication/Signal transduction mechanism/transmembrane signal transductio	Pyruvateorthophosphate Dikinase	PPL-14-F02	364	E-102	88	DN554825
AT1G49490	Leucine-Rich Repeat Family Protein	Cellular communication/Signal transduction mechanism/transmembrane signal transductio	Flavanone 3-Hydroxylase (Fh3)	PPL-17-G04	135	6.00E-49	52	DN555571
AT3G22800	Leucine-Rich Repeat Family Protein	Cellular communication/Signal transduction mechanism/transmembrane signal transductio	Flavanone 3-Hydroxylase (Fh3)	PPL-17-G04	110	2.00E-41	44	DN555571

AT3G13670	Protein Kinase Family Protein	Cellular communication/Signal transduction mechanism/transmembrane signal transductio	Unknown Protein	PPL-19-F01	329	2.00E-91	84	DN555650
AT5G51760	Protein Phosphatase 2C, Putative	Cellular communication/Signal transduction mechanism/transmembrane signal transductio	Unknown Protein	PPL-21-B03	181	9.00E-47	63	DN555881
**C. Cell Cycle/ DNA processing/chromatin regulation and cytoskeleton reorganization**								
AT5G10470	Kinesin Motor Protein-Related	Cell Cycle/ DNA processing/chromatin regulation and cytoskeleton reorganization	Kinesin-Related Protein	PPL-12-B09	234	6.00E-74	74	DN554610
			Unknown Protein	PPL-19-H10	217	4.00E-57	75	DN555681
AT4G13570	Histone H2A	Cell Cycle/ DNA processing/chromatin regulation and cytoskeleton reorganization	Unknown Protein	PPL04-D03	122	6.00E-29	89	DN553174
AT1G04820	Tubulin Alpha-2	Cell Cycle/ DNA processing/chromatin regulation and cytoskeleton reorganization	Unknown Protein	PPL-09-D12	393	E-110	96	DN554249
			Unknown Protein	PPL-21-E12	162	3.00E-41	91	DN555925
			Unknown Protein	PPL-09-G12	258	5.00E-70	94	DN554282
AT5G19770	Tubulin Alpha-3	Cell Cycle/ DNA processing/chromatin regulation and cytoskeleton reorganization	Unknown Protein	PPL-09-D12	383	E-107	92	DN554249
			Unknown Protein	PPL-21-E12	158	4.00E-40	85	DN555925
			Unknown Protein	PPL-09-G12	254	9.00E-69	89	DN554282
**D. Development/storage proteins**								
AT2G35490	Plastid-Lipid Associated Protein	Development/storage proteins	Pectinesterase Like Protein	PPL-18-C01	168	2.00E-42	44	DN555797
AT4G09610	Gibberellin-Regulated Protein	Development/storage proteins	Water Channel - Like Protein	PPL-18-F10	112	2.00E-26	54	DN555842
AT1G59750	Auxin-Responsive Factor (Arf1)	Development/storage proteins	Unknown Protein	PPL-13-A04	132	2.00E-31	59	DN554685
**E. Metabolism/energy/membrane associated proteins**								
AT3G54050	Fructose-1,6-Bisphosphatase	Metabolism/energy/membrane associated proteins	Unknown Protein	PPL-01-B03B12; PPL-05-A05	142	2.00E-35	85	DN555080; DN553227
			Retrotransposon Like Protein	PPL-06-A01	138	9.00E-34	55	DN553867
			Fructose 16-Bisphosphatase Putative	PPL-22-C02	224	1.00E-59	59	DN555986
			Unknown Protein	PPL-16-G12	134	8.00E-53	56	DN556401
			Water Channel - Like Protein	PPL-12-D01	249	2.00E-67	57	DN554625
			Unknown Protein	PPL-09-F04	250	2.00E-67	56	DN554263
AT3G54050	Fructose-1,6-Bisphosphatase	Metabolism/energy/membrane associated proteins	Unknown Protein	PPL-01-D01D10	141	5.00E-35	84	DN552965
AT3G19000	Oxidoreductase, 2Og-Fe(Ii) Oxygenase Family Protein	Metabolism/energy/membrane associated proteins	Air1A-Like Protein	PPL-02-B07	224	7.00E-60	67	DN552997
AT2G39730	Ribulose Bisphosphate Carboxylase/Oxygenase Activase	Metabolism/energy/membrane associated proteins	Unknown Protein	PPL-06-D04	371	E-104	94	DN553870
			Pectinesterase Like Protein	PPL-25-E07	218	6.00E-58	93	DN556284
			Unknown Protein	PPL-22-D09	122	4.00E-29	74	DN556005
			Pectinesterase Like Protein	PPL-21-A06	202	4.00E-53	79	DN555872
			Water Channel - Like Protein	PPL-17-G08	203	2.00E-53	75	DN555575
			Unknown Protein	PPL-16-F09	152	5.00E-38	77	DN556386
			Unknown Protein	PPL-08-_T3__F08	361	E-101	85	DN554182
AT4G13890	Glycine Hydroxymethyltransferase	Metabolism/energy/membrane associated proteins	Unknown Protein	PPL-06-D05	111	7.00E-26	50	DN553957
			Water Channel - Like Protein	PPL-24-C08	124	1.00E-45	52	DN556173
			Histon H3 Protein	PPL-18-E06	207	2.00E-68	68	DN555826
			Unknown Protein	PPL-15-B04	221	3.00E-58	70	DN554871
			H+-Transporting Atpase - Like Protein	PPL-14-C11	132	1.00E-47	51	DN554805
			Unknown Protein	PPL-08-_T3__E08	150	2.00E-37	73	DN554170
AT2G26080	Glycine Dehydrogenase	Metabolism/energy/membrane associated proteins	Putative Glycine Dehydrogenase	PPL-06-D12	404	E-114	94	DN553964
			Unknown Protein	PPL-20-G03	209	3.00E-55	94	DN555754
			Profilin 1	PPL-18-G06	351	6.00E-98	90	DN555850
			Unknown Protein	PPL-24-F04	361	E-101	91	DN556203
			Cinnamoyl-Coa Reductase	PPL-16-C11	154	1.00E-38	90	DN556355
			Pectinesterase Like Protein	PPL-17-H12	335	3.00E-93	91	DN555590
			Unknown Protein	PPL-06-G02	292	4.00E-80	92	DN553988
			Unknown Protein	PPL-09-E06	400	E-112	91	DN554254
			Unknown Protein	PPL-12-D04	393	E-110	89	DN554628
			Unknown Protein	PPL-08-_T3__C12	386	E-108	90	DN554151
AT4G33010	Glycine Dehydrogenase	Metabolism/energy/membrane associated proteins	Putative Glycine Dehydrogenase	PPL-06-D12	403	E-113	94	DN553964
			Unknown Protein	PPL-24-F04	358	E-100	90	DN556203
			Unknown Protein	PPL-20-G03	209	4.00E-55	94	DN555754
			Cinnamoyl-Coa Reductase	PPL-16-C11	148	6.00E-37	78	DN556355
			Profilin 1	PPL-18-G06	349	2.00E-97	90	DN555850
			Pectinesterase Like Protein	PPL-17-H12	332	2.00E-92	85	DN555590
			Unknown Protein	PPL-12-D04	394	E-111	90	DN554628
			Unknown Protein	PPL-06-G02	289	3.00E-79	85	DN553988
			Unknown Protein	PPL-09-E06	402	E-113	92	DN554254
			Unknown Protein	PPL-08-_T3__C12	394	E-111	91	DN554151
AT3G14415	Glycolate Oxidase, Putative	Metabolism/energy/membrane associated proteins	Glycolate Oxidase Like Protein	PPL-06-E08	355	4.00E-99	89	DN553971
			Pectinesterase Like Protein	PPL-21-G03	178	4.00E-46	92	DN555940
			Glycolate Oxidase Like Protein	PPL-09-H06	376	E-105	90	DN554288
AT2G21330	Fructose-Bisphosphate Aldolase	Metabolism/energy/membrane associated proteins	Fructose Bisphosphate Aldolase Like Protein	PPL-06-E12	216	2.00E-57	82	DN553975
			Unknown Protein	PPL-17-A02	128	3.00E-31	74	DN555504
			Hsp 70-Like Protein	PPL-17-C09	366	E-102	89	DN555533
			Unknown Protein	PPL-16-C07	231	6.00E-62	83	DN556351
			Pectinesterase Like Protein	PPL-13-F10	344	8.00E-96	82	DN554745
			Fructose-Bisphosphate Aldolase Like Protein	PPL-06-F07; PPL-13-G11	230	3.00E-64	90	DN553981; DN554758
			Unknown Protein	PPL-08-_T3__A02	251	5.00E-68	87	DN554119
			Unknown Protein	PPL-07-_T3__E03	380	E-106	89	DN554075
AT2G01140	Fructose-Bisphosphate Aldolase	Metabolism/energy/membrane associated proteins	Fructose Bisphosphate Aldolase Like Protein	PPL-06-E12	187	2.00E-48	46	DN553975
			Unknown Protein	PPL-17-A02	100	1.00E-22	65	DN555504
			Hsp 70-Like Protein	PPL-17-C09	346	3.00E-96	83	DN555533
			Unknown Protein	PPL-16-C07	201	6.00E-53	77	DN556351
			Pectinesterase Like Protein	PPL-13-F10	305	4.00E-84	77	DN554745
			Fructose-Bisphosphate Aldolase Like Protein	PPL-06-F07; PPL-13-G11	201	6.00E-53	73	DN553975; DN554758
			Unknown Protein	PPL-08-_T3__A02	231	4.00E-62	80	DN554119

			Unknown Protein	PPL-07-_T3__E03	359	E-100	84	DN554075
AT3G52930	Fructose-Bisphosphate Aldolase	Metabolism/energy/membrane associated proteins	Fructose-Bisphosphate Aldolase Like Protein	PPL-06-F07	163	2.00E-41	59	DN553975
			Hsp 70-Like Protein	PPL-17-C09	107	2.00E-49	54	DN555533
			Unknown Protein	PPL-08-_T3__A02	79	5.00E-24	65	DN554119
			Fructose-Bisphosphate Aldolase Like Protein	PPL-13-G11	145	5.00E-36	59	DN554758
			Unknown Protein	PPL-10-H02	180	1.00E-47	74	DN554490
			Unknown Protein	PPL-07-_T3__E03	107	6.00E-51	54	DN554075
AT4G26530	Fructose-Bisphosphate Aldolase	Metabolism/energy/membrane associated proteins	Unknown Protein	PPL-07-_T3__E03; PPL-17-C09	105	2.00E-50	54	DN554075 ; DN555533
			Fructose-Bisphosphate Aldolase Like Protein	PPL-13-G11	163	2.00E-41	54	DN554758
			Unknown Protein	PPL-10-H02	214	8.00E-57	84	DN554490
AT1G56190	Phosphoglycerate Kinase, Putative	Metabolism/energy/membrane associated proteins	14-3-3 Protein Epsilon	PPL-06-G05	155	4.00E-39	85	DN553991
			Unknown Protein	PPL-23-C11	230	1.00E-61	86	DN556083
			14-3-3 Protein	PPL-06-G05	139	3.00E-34	89	DN553991
			Proteasome Component C5	PPL-11-H07	297	7.00E-82	86	DN554585
			Unknown Protein	PPL-09-H04	309	7.00E-85	85	DN554286
AT1G79550	Phosphoglycerate Kinase, Putative	Metabolism/energy/membrane associated proteins	Unknown Protein	PPL-09-H04	264	1.00E-71	78	DN554286
			Unknown Protein	PPL-23-C11	212	3.00E-56	91	DN556083
			Unknown Protein	PPL-11-H07	311	7.00E-86	85	DN554585
AT3G04120	Glyceraldehyde-3-Phosphate Dehydrogenase	Metabolism/energy/membrane associated proteins	Unknown Protein	PPL-06-H12	159	4.00E-40	50	DN554007
AT4G39350	Cellulose Synthase	Metabolism/energy/membrane associated proteins	Cellulose Synthase Catalytic Subunit	PPL-07-_T3__A09	383	E-107	94	DN554039
AT5G05170					318	6.00E-88	75	DN554039
AT4G32410					315	3.00E-87	77	DN554039
AT4G18780					220	7.00E-82	69	DN554039
AT3G52200	Dihydrolipoamide S-Acetyltransferase	Metabolism/energy/membrane associated proteins	Unknown Protein	PPL-07-_T3__B07	348	2.00E-96	83	DN554046
AT3G03780	5-Methyltetrahydropteroyltriglutamate--Homocysteine	Metabolism/energy/membrane associated proteins	Unknown Protein	PPL-07-_T3__F01	376	E-104	90	DN554084
AT4G27440	Protochlorophyllide Reductase B	Metabolism/energy/membrane associated proteins	Water Channel - Like Protein	PPL-07-_T3__F07	125	3.00E-44	77	DN554089
AT4G23460	Beta-Adaptin	Metabolism/energy/membrane associated proteins	Unknown Protein	PPL-07-_T3__F12	135	1.00E-33	69	DN554094
AT4G11380	Beta-Adaptin	Metabolism/energy/membrane associated proteins	Unknown Protein	PPL-07-_T3__F12	128	2.00E-31	66	DN554094
AT1G77380	Amino Acid Carrier, Putative	Metabolism/energy/membrane associated proteins	Unknown Protein	PPL-07-_T3__G08	158	8.00E-40	77	DN554102
AT1G68010	Glycerate Dehydrogenase	Metabolism/energy/membrane associated proteins	Unknown Protein	PPL-08-_T3__A12	215	5.00E-57	84	DN554129
AT5G04140	Glutamate Synthase (Glu1)	Metabolism/energy/membrane associated proteins	Unknown Protein	PPL-09-A06	369	E-103	93	DN554214
AT2G41220	Glutamate Synthase (Glu1)	Metabolism/energy/membrane associated proteins	Unknown Protein	PPL-09-A06	359	E-100	90	DN554214
AT5G53460	Glutamate Synthase (Glu1)	Metabolism/energy/membrane associated proteins	Unknown Protein	PPL-09-A06	221	1.00E-58	53	DN554214
AT3G17820	Glutamine Synthetase (Gs1)	Metabolism/energy/membrane associated proteins	Unknown Protein	PPL-09-F06	143	3.00E-35	95	DN554265
			Water Channel - Like Protein	PPL-20-E09	348	2.00E-96	87	DN555737
			Unknown Protein	PPL-18-D02	106	2.00E-24	71	DN555810
			Unknown Protein	PPL-16-D10	345	8.00E-96	78	DN556365
			Phenylalanine Ammonia Lyase (Pal1)	PPL-12-A06	261	6.00E-71	88	DN554596
AT5G36890	Glycosyl Hydrolase Family 1 Protein	Metabolism/energy/membrane associated proteins	Putative Prunasin Hydrolase Isoform Ph-L1 Precursor	PPL-09-F11	127	1.00E-30	53	DN554270
AT3G18090	Dna-Directed Rna Polymerase Family Protein	Metabolism/energy/membrane associated proteins	Unknown Protein	PPL-10-B09	300	1.00E-82	83	DN554428
AT1G11840	Lactoylglutathione Lyase, Putative	Metabolism/energy/membrane associated proteins	Glyoxalase I	PPL-08-_T3__D07	188	4.00E-49	80	DN554157
			Adp-Ribosylation Factor 1	PPL-22-E09	315	5.00E-87	86	DN556017
			Beta-Galactosidase Like Protein	PPL-18-B02	170	2.00E-43	83	DN555788
			Unknown Protein	PPL-11-F04	317	7.00E-87	80	DN554562
			Pollen-Specific Protein	PPL-11-B03	297	4.00E-81	85	DN554516
AT5G13630	Magnesium-Chelatase Subunit	Metabolism/energy/membrane associated proteins	Unknown Protein	PPL-10-H06	357	E-100	91	DN554494
AT5G56630	Phosphofructokinase Family Protein	Metabolism/energy/membrane associated proteins	Putative 60S Ribosomal Protein L35	PPL-11-A04	193	3.00E-57	75	DN554505
AT4G25420	Gibberellin 20-Oxidase	Metabolism/energy/membrane associated proteins	Gibberellin 20-Oxidase	PPL-12-C05	138	2.00E-50	70	DN554617
AT2G39730	Ribulose Bisphosphate Carboxylase	Metabolism/energy/membrane associated proteins	Rubisco Activase	PPL-12-C09	380	E-107	85	DN554621
			Unknown Protein	PPL-25-H10	159	2.00E-40	74	DN556321
			Unknown Protein	PPL-22-F10	203	2.00E-53	78	DN556028
			Cysteine Proteinase Inhibitor Like Protein	PPL-21-G07	215	7.00E-69	77	DN555944
			Probable Photosystem I Chain Xi Precursor	PPL-19-B07	218	6.00E-58	93	DN555609
AT1G62380	1-Aminocyclopropane-1-Carboxylate Oxidase	Metabolism/energy/membrane associated proteins	Pectinesterase Like Protein	PPL-12-H03	285	9.00E-78	62	DN554673
			Pectinesterase Like Protein	PPL-25-B01	202	8.00E-53	55	DN556244
AT1G12010	1-Aminocyclopropane-1-Carboxylate Oxidase	Metabolism/energy/membrane associated proteins	Pectinesterase Like Protein	PPL-12-H03	285	9.00E-78	64	DN554673
			Pectinesterase Like Protein	PPL-25-B01	206	1.00E-54	59	DN556244
AT1G02500	S-Adenosylmethionine Synthetase 1	Metabolism/energy/membrane associated proteins	Unknown Protein	PPL-13-A09	189	5.00E-50	91	DN554690
AT4G01850	S-Adenosylmethionine Synthetase 2	Metabolism/energy/membrane associated proteins	Unknown Protein	PPL-13-A09	189	2.00E-49	89	DN554690
AT3G47860	Apolipoprotein D-Related	Metabolism/energy/membrane associated proteins	Unknown Protein	PPL-13-F05	286	1.00E-90	80	DN554740
AT3G50990	Similar To Peroxidase 72 (Per72)	Metabolism/energy/membrane associated proteins	Unknown Protein	PPL-14-B06	126	3.00E-31	52	DN554788
AT3G57520	Alkaline Alpha Galactosidase, Putative	Metabolism/energy/membrane associated proteins	Unknown Protein	PPL-15-D05	359	E-100	89	DN554891
AT3G16240	Delta Tonoplast Integral Protein	Metabolism/energy/membrane associated proteins	Water Channel - Like Protein	PPL-17-H11	296	2.00E-81	81	DN555589
AT2G36830	Major Intrinsic Family Protein	Metabolism/energy/membrane associated proteins	Water Channel - Like Protein	PPL-17-H11	231	9.00E-62	64	DN555589
AT4G39800	Myo-Inositol-1-Phosphate Synthase 1	Metabolism/energy/membrane associated proteins	Putative Beta-1,3-Glucanase	PPL-19-C09	177	2.00E-45	91	DN555623
AT5G53370	Pectinesterase Family Protein	Metabolism/energy/membrane associated proteins	Water Channel - Like Protein	PPL-19-E12	243	1.00E-65	64	DN555649
AT3G43270	Pectinesterase Family Protein	Metabolism/energy/membrane associated proteins	Water Channel - Like Protein	PPL-19-E12	219	2.00E-58	58	DN555649
AT5G09760	Pectinesterase Family Protein	Metabolism/energy/membrane associated proteins	Water Channel - Like Protein	PPL-19-E12	130	3.00E-45	56	DN555649
AT4G04610	5'-Adenylylsulfate Reductase	Metabolism/energy/membrane associated proteins	Unknown Protein	PPL-19-F10	137	1.00E-33	53	DN555659
AT3G17820	Glutamine Synthetase (Gs1)	Metabolism/energy/membrane associated proteins	Nad Dependent Epimerase, Putative	PPL-19-G02	332	2.00E-92	87	DN555663
AT1G31330	Photosystem I Reaction Center Subunit	Metabolism/energy/membrane associated proteins	Water Channel - Like Protein	PPL-19-H01	130	2.00E-32	81	DN555673
			Unknown Protein	PPL-23-A05	278	5.00E-76	85	DN556055
AT4G04610	5'-Adenylylsulfate Reductase	Metabolism/energy/membrane associated proteins	Prh26	PPL-18-H04	157	9.00E-78	82	DN555859
			Unknown Protein	PPL-23-E09	131	8.00E-32	79	DN556103
AT5G24300	Starch Synthase	Metabolism/energy/membrane associated proteins	Water Channel - Like Protein	PPL-18-H07	312	1.00E-85	78	DN555862
AT1G56190	Phosphoglycerate Kinase	Metabolism/energy/membrane associated proteins	Subtilisin-Like Proteinase	PPL-19-A10	101	6.00E-23	83	DN555600
			Unknown Protein	PPL-19-B12	322	3.00E-89	89	DN555614
AT1G79550	Phosphoglycerate Kinase	Metabolism/energy/membrane associated proteins	Unknown Protein	PPL-19-B12	318	5.00E-88	89	DN555614
AT1G15690	Pyrophosphate-Energized Vacuolar	Metabolism/energy/membrane associated proteins	Unknown Protein	PPL-21-E10	180	1.00E-46	75	DN555923

			Unknown Protein	PPL-22-C12	116	3.00E-27	85	DN555996
AT3G03780	5-Methyltetrahydropteroyltriglutamate--Homocysteine	Metabolism/energy/membrane associated proteins	Pectinesterase Like Protein	PPL-21-E11	308	3.00E-83	85	DN555924
AT1G56190	Phosphoglycerate Kinase, Putative	Metabolism/energy/membrane associated proteins	Endoxyloglucan Transferase-Like Protein	PPL-21-F11	144	1.00E-35	86	DN555936
AT1G79550	Phosphoglycerate Kinase, Putative	Metabolism/energy/membrane associated proteins	Endoxyloglucan Transferase-Like Protein	PPL-21-F11	128	7.00E-31	91	DN555936
AT4G00430	Plasma Membrane Intrinsic Protein	Metabolism/energy/membrane associated proteins	Unknown Protein	PPL-21-B06	209	3.00E-55	92	DN555884
					226	1.00E-60	92	DN556279
AT1G31710	Copper Amine Oxidase	Metabolism/energy/membrane associated proteins	Unknown Protein	PPL-24-E10	108	2.00E-41	58	DN556197
			Pectinesterase Like Protein	PPL-24-F10	110	7.00E-42	58	DN556209
AT3G25230	Peptidyl-Prolyl Cis-Trans Isomerase	Metabolism/energy/membrane associated proteins	Pectinesterase Like Protein	PPL-24-F09	292	9.00E-78	81	DN556208
AT5G48570	Peptidyl-Prolyl Cis-Trans Isomerase	Metabolism/energy/membrane associated proteins	Pectinesterase Like Protein	PPL-24-F09	291	2.00E-77	79	DN556208
AT2G18940	Pentatricopeptide (Ppr Repeat-Containing Protein	Metabolism/energy/membrane associated proteins	Flavonoid 3',5'-Hydroxylase -Like Protein	PPL-25-A11	269	3.00E-73	76	DN556242
AT3G17240	Dihydrolipoamide Dehydrogenase 2	Metabolism/energy/membrane associated proteins	Water Channel - Like Protein	PPL-23-B07	136	2.00E-33	88	DN556067
AT3G16910	Acetyl-Coa Synthetase	Metabolism/energy/membrane associated proteins	Unknown Protein	PPL-23-C03	108	5.00E-47	55	DN556075
AT5G48300	Glucose-1-Phosphate Adenylyltransferase	Metabolism/energy/membrane associated proteins	Cucumisin Precursor	PPL-23-D02	122	5.00E-29	69	DN556086
			Pectinesterase Like Protein	PPL-23-F06	151	6.00E-38	97	DN556112
AT5G06290	2-Cys Peroxiredoxin, Chloroplast	Metabolism/energy/membrane associated proteins	Unknown Protein	PPL-25-F12	177	8.00E-46	87	DN556300
**F. Proteins with binding function or cofactor requirement**								
AT3G56940	Dicarboxylate Diiron Protein, Putative	Proteins with binding function or cofactor requirement	Unknown Protein	PPL-01-F09F09	101	5.00E-23	71	DN552942
			Water Channel - Like Protein	PPL-25-E04	377	E-106	97	DN556281
			Water Channel - Like Protein	PPL-14-G11	294	9.00E-81	85	DN554843
			S-Adenosylmethionine Synthetase	PPL-08-_T3__B12	417	E-118	97	DN554141
AT1G48630	Activated Protein Kinase C Receptor, Putative	Proteins with binding function or cofactor requirement	Guanine Nucleotide-Binding Protein Beta Subunit-Like Protein	PPL-06-D02	363	E-101	86	DN553955
AT1G18080	Wd-40 Repeat Family Protein	Proteins with binding function or cofactor requirement	Guanine Nucleotide-Binding Protein Beta Subunit-Like Protein	PPL-06-D02	303	2.00E-96	83	DN553955
AT1G20980	Spl1-Related2 Protein	Proteins with binding function or cofactor requirement	Squamosa Promoter Binding Protein-Like	PPL-06-D10	145	4.00E-35	59	DN553962
			Unknown Protein	PPL-10-D09	148	8.00E-37	60	DN554452
AT3G60030	Squamosa Promoter-Binding Protein	Proteins with binding function or cofactor requirement	Squamosa Promoter Binding Protein-Like	PPL-06-D10	141	7.00E-34	71	DN553962
			Unknown Protein	PPL-10-D09	145	5.00E-36	65	DN554452
AT3G17880	Tetratricoredoxin (Tdx)	Proteins with binding function or cofactor requirement	Thioredoxin H	PPL-06-F09	123	3.00E-29	41	DN553983
AT1G35550	Elongation Factor Tu C-Terminal Domain	Proteins with binding function or cofactor requirement	Unknown Protein	PPL-10-C01	256	2.00E-69	80	DN554432
			Translation Elongation Factor Eef-1 Alpha Chain	PPL-13-E06	102	5.00E-23	77	DN554729
AT4G17520	Nuclear Rna-Binding Protein	Proteins with binding function or cofactor requirement	Unknown Protein	PPL-08-_T3__D02	107	1.00E-27	44	DN554153
AT2G24820	Rieske (2Fe-2S) Domain	Proteins with binding function or cofactor requirement	Ps I Antenna Protein Lhca2 Fragment	PPL-14-B09	142	5.00E-35	67	DN554791
			Unknown Protein	PPL-16-G03	116	3.00E-27	65	DN556392
AT4G24800	Ma3 Domain-Containing Protein	Proteins with binding function or cofactor requirement	Unknown Protein	PPL-13-D07	166	1.00E-47	82	DN554718
AT1G46768	Erf/Ap2 Transcription Factor	Proteins with binding function or cofactor requirement	Ribosomal Protein L29, Putative	PPL-17-B09	100	2.00E-22	62	DN555522
AT1G63480	Dna-Binding Family Protein	Proteins with binding function or cofactor requirement	Rps7A - Ribosomal Protein	PPL-20-B10	136	3.00E-33	55	DN555702
AT5G67470	Formin Homology 2 Domain	Proteins with binding function or cofactor requirement	Water Channel - Like Protein	PPL-17-G06	174	3.00E-47	86	DN555573
AT3G62300	Agenet Domain-Containing Protein	Proteins with binding function or cofactor requirement	Water Channel - Like Protein	PPL-23-A10	141	2.00E-34	52	DN556059
AT2G28000	Rubisco Subunit Binding-Protein	Proteins with binding function or cofactor requirement	Nucleoid Dna-Binding-Like Protein	PPL-24-C09	247	2.00E-71	86	DN556174
AT2G43770	Transducin Family Protein	Proteins with binding function or cofactor requirement	Unknown Protein	PPL-25-G04	319	1.00E-88	89	DN556304
**G. Protein synthesis and translation**								
AT1G15810	Ribosomal Protein S15 Family Protein	Protein synthesis and translation	Chlorophyll A/B-Binding Protein	PPL-06-F03	178	5.00E-46	92	DN553978
			Unknown Protein	PPL-25-B09	242	3.00E-65	86	DN556252
			Pectinesterase Like Protein	PPL-24-C01	237	5.00E-64	86	DN556166
			Unknown Protein	PPL-23-G01	271	6.00E-74	89	DN556119
			Unknown Protein	PPL-23-B01	208	3.00E-55	84	DN556062
			Unknown Protein	PPL-17-B10	105	1.00E-24	86	DN555523
			Pectinesterase Like Protein	PPL-18-E08	253	9.00E-69	90	DN555828
			Unknown Protein	PPL-18-E10	104	3.00E-24	90	DN555830
			Unknown Protein	PPL-18-D07	341	7.00E-95	87	DN555815
			Pectinesterase Like Protein	PPL-17-E08	274	6.00E-75	87	DN555554
			Unknown Protein	PPL-09-A03	234	8.00E-63	90	DN554211
			Putative Ribosomal Protein L28	PPL-11-F12	188	4.00E-49	92	DN554570
			Unknown Protein	PPL-12-G05	215	3.00E-57	85	DN554663
			Unknown Protein	PPL-09-C07	199	3.00E-52	92	DN554233
			Unknown Protein	PPL-08-_T3__B10	267	7.00E-73	87	DN554139
AT1G43170	60S Ribosomal Protein L3	Protein synthesis and translation	Unknown Protein	PPL-08-_T3__F01	180	1.00E-46	80	DN554175
			Unknown Protein	PPL-19-G08	157	1.00E-39	80	DN555668
AT1G79850	30S Ribosomal Protein S17	Protein synthesis and translation	Pollen Allergen -Like Protein	PPL-06-A11	174	5.00E-44	62	DN553934
AT5G60390	Elongation Factor 1-Alpha	Protein synthesis and translation	Unknown Protein	PPL-10-C01	352	2.00E-98	94	DN554432
			Translation Elongation Factor Eef-1 Alpha Chain	PPL-13-E06	133	1.00E-32	91	DN554729
			Unknown Protein	PPL-10-C10	350	1.00E-97	94	DN554441
AT1G62750	Elongation Factor Tu Family Protein	Protein synthesis and translation	Flavanone 3-Hydroxylase (Fh3)	PPL-10-G03	152	2.00E-38	89	DN554480
AT3G63490	Ribosomal Protein L1 Family Protein	Protein synthesis and translation	Unknown Protein	PPL-15-E08	234	7.00E-63	85	DN554905
AT3G13580	60S Ribosomal Protein L7	Protein synthesis and translation	Unknown Protein	PPL-21-F09	127	5.00E-31	92	DN555934
AT1G02780	60S Ribosomal Protein L19	Protein synthesis and translation	Water Channel - Like Protein	PPL-22-C01	117	1.00E-27	75	DN555985
**H. Protein fate**								
AT1G62510	Protease Inhibitor	Protein fate	Unknown Protein	PPL-25-C03	127	2.00E-31	51	DN556257
AT5G20620	Polyubiquitin (Ubq4)	Protein fate	Unknown Protein	PPL-08-_T3__G05	256	2.00E-69	100	DN554190
			Unknown Protein	PPL-23-H10	326	1.00E-89	97	DN556140
			Unknown Protein	PPL-14-H11	391	E-108	99	DN554855
AT1G53930	Ubiquitin Family Protein	Protein fate	Unknown Protein	PPL-08-_T3__G05	131	8.00E-32	75	DN554190
			Unknown Protein	PPL-23-H10	130	3.00E-31	76	DN556140
			Unknown Protein	PPL-14-H11	131	1.00E-31	75	DN554855
AT1G09130	Atp-Dependent Clp Protease	Protein fate	Clpp Protease Complex Subunit Clpr1	PPL-08-_T3__H01	164	3.00E-41	51	DN554198
AT4G20850	Subtilase Family Protein	Protein fate	Pectinesterase Like Protein	PPL-19-A11	101	9.00E-23	66	DN555601

AT3G22120	Protease Inhibitor	Protein fate	Unknown Protein	PPL-25-C03	118	8.00E-54	73	DN556257
AT2G10940	Protease Inhibitor	Protein fate	Unknown Protein	PPL-25-C03	167	8.00E-43	50	DN556257
**I. Transcription/splicing/RNA processing/modification**								
AT2G42280	Basic Helix-Loop-Helix (Bhlh) Family Protein	Transcription/splicing/RNA processing/modification	Peroxidase - Like Protein	PPL04-H11	156	2.00E-39	71	DN553224
AT1G51140	Basic Helix-Loop-Helix (Bhlh) Family Protein	Transcription/splicing/RNA processing/modification	Peroxidase - Like Protein	PPL04-H11	153	2.00E-38	72	DN553224
AT1G68520	Zinc Finger (B-Box Type) Family	Transcription/splicing/RNA processing/modification	Zinc Finger Protein	PPL-06-C08	109	3.00E-25	90	DN553950
AT1G32750	Hac13 Protein (Hac13)	Transcription/splicing/RNA processing/modification	Unknown Protein	PPL-10-F12	161	8.00E-41	74	DN554477
			Unknown Protein	PPL-25-B10	246	2.00E-66	74	DN556253
AT3G02380	Zinc Finger Protein Constans	Transcription/splicing/RNA processing/modification	Unknown Protein	PPL-17-H01	109	4.00E-25	71	DN555580
AT5G15850	Zinc Finger Protein Constans	Transcription/splicing/RNA processing/modification	Unknown Protein	PPL-17-H01	105	8.00E-24	75	DN555580
**J. Transporters**								
AT3G62770	Transport Protein-Related	Transporters	Unknown Protein	PPL-07-_T3__D05	166	6.00E-73	81	DN554066
AT1G06950	Protein Import Into The Chloroplast	Transporters	Unknown Protein	PPL-07-_T3__E07	113	2.00E-40	77	DN554079
AT3G27020	Oligopeptide Transporter	Transporters	Unknown Protein	PPL-07-_T3__E10	224	3.00E-74	60	DN554081
AT4G39170	Sec14 Cytosolic Factor, Putative	Transporters	Unknown Protein	PPL-16-E12	137	2.00E-33	88	DN556377
AT5G64840	Abc Transporter Family Protein	Transporters	Abc Transporter	PPL-22-C03	331	5.00E-92	87	DN555987
**L. Unknown**								
AT3G01060	Expressed Protein	Unknown	Unknown Protein	PPL-18-A01	327	7.00E-91	92	DN555775
AT1G21680	Expressed Protein	Unknown	Aquaporin 4	PPL-19-H06	154	4.00E-41	77	DN555677
AT4G28910	Expressed Protein	Unknown	Pectinesterase Like Protein	PPL-20-F10	176	6.00E-49	81	DN555749
AT2G42310	Expressed Protein	Unknown	Unknown Protein	PPL-10-C09	182	5.00E-47	80	DN554440
AT5G04460	Expressed Protein	Unknown	Subtilisin-Like Proteinase	PPL-10-F11	115	3.00E-35	69	DN554476
			Cucumisin Precursor	PPL-17-A09	146	3.00E-36	91	DN555511
AT5G57930	Expressed Protein	Unknown	Flavonol Synthase -Like	PPL-10-G08	138	5.00E-47	59	DN554485
AT2G30900	Expressed Protein	Unknown	Unknown Protein	PPL-11-H04	100	3.00E-47	51	DN554583
AT5G16730	Expressed Protein	Unknown	Unknown Protein	PPL-13-D06	127	8.00E-30	60	DN554717
AT1G09310	Expressed Protein	Unknown	Water Channel - Like Protein	PPL-17-F04	182	4.00E-53	69	DN555560
			Unknown Protein	PPL-21-C05	107	5.00E-32	71	DN555895
			Water Channel - Like Protein	PPL-17-F04	131	6.00E-31	49	DN555560
AT3G46780	Expressed Protein	Unknown	Unknown Protein	PPL-07-_T3__C10	143	6.00E-36	69	DN554060
**II. *Arabidopsis* gene sequences significantly repressed by PPV in the infected leaf tissues show sequence similarity to *Prunus persica* uniESTs**								
**A. Defence and virulence**								
AT5G38480	14-3-3 protein; GRF3	Defence and virulence	General regulatory factor 3	PPL-10-A05	186	3.00E-51	82	DN554412
			General regulatory factor 3	PPL-24-G03	186	3.00E-51	82	DN556214
			General regulatory factor 3	PPL-17-F01	141	2.00E-37	83	DN555558
AT5G16400	Thioredoxin, putative	Defence and virulence	Thioredoxin, putative	PPL-20-E02	111	1.00E-28	80	DN555730
			Thioredoxin, putative	PPL-16-E02	137	2.00E-36	81	DN556368
**C. Cell Cycle/ DNA processing/chromatin regulation and cytoskeleton reorganization**								
AT3G45980	Histone H2B	Cell Cycle/ DNA processing/chromatin regulation and cytoskeleton reorganization	Histone H2B	PPL-04-A09	129	2.00E-34	87	DN553147
AT3G54560	Histone H2A.F/Z		Histone H2A.F/Z	PPL-04-D03	194	7.00E-54	87	DN553174
**E. Metabolism/energy/membrane associated proteins**								
AT5G64040	Photosystem I reaction center subunit PSI-N	Primary /secondary metabolism	Photosystem I reaction center subunit PSI-N	PPL-15-E03	258	6.00E-73	87	DN554901
**F. Proteins with binding function or cofactor requirement**								
AT3G42830	Ring-box protein Roc1/Rbx1/Hrt1, putative	Proteins with binding function or cofactor requirement	Ring-box protein Roc1/Rbx1/Hrt1, putative	PPL-16-H04	151	5.00E-41	84	DN556405
AT4G17530	Ras-related GTP-binding protein, putative	Proteins with binding function or cofactor requirement	Ras-related GTP-binding protein, putative	PPL-16-E07	254	1.00E-71	85	DN556372
AT5G20570	Ring-box protein-related	Proteins with binding function or cofactor requirement	Ring-box protein-related	PPL-16-H04	208	5.00E-58	85	DN556405
**G. Protein synthesis and translation**								
AT2G19750	40S ribosomal protein S30;RPS30A	Protein synthesis and translation	40S ribosomal protein S30	PPL-25-D04	161	3.00E-44	86	DN556270
			40S ribosomal protein S30	PPL-01-G02G11	119	7.00E-32	86	DN556431
				PPL-17-H07	161	3.00E-44	86	DN555585
AT3G06700	60S ribosomal protein L29;RPL29A	Protein synthesis and translation	60S ribosomal protein L29-1; RPL29A	PPL-24-B06	105	3.00E-27	84	DN556159
AT4G29390	40S ribosomal protein S30;RPS30B	Protein synthesis and translation	40S ribosomal protein S30;RPS30B	PPL-25-D04	141	7.00E-38	84	DN556270
			40S ribosomal protein S30;RPS30B	PPL-01-G02G11	105	3.00E-27	85	DN556431
			40S ribosomal protein S30;RPS30B	PPL-17-H07	141	7.00E-38	84	DN555585
AT5G56670	40S ribosomal protein S30;RPS30C	Protein synthesis and translation	40S ribosomal protein S30;RPS30B	PPL-25-D04	153	6.00E-42	85	DN556270
			40S ribosomal protein S30;RPS30B	PPL-17-H07	153	7.00E-42	85	DN555585
**H. Protein fate**								
AT2G36170	Ubiquitin extension protein 2; UBQ2	Ubiquitin like conjugating enzyme activity	Ubiquitin extension protein 2; UBQ2	PPL-03-B07	133	1.00E-35	88	DN553076
			Ubiquitin extension protein 2; UBQ2	PPL-14-H11	141	1.00E-37	86	DN554855
					131	1.00E-34	84	DN554855
AT3G52590	Ubiquitin extension protein 1; UBQ1	Ubiquitin like conjugating enzyme activity	Ubiquitin extension protein 1; UBQ1	PPL-03-B07	105	3.00E-27	85	DN553076
			Ubiquitin extension protein 1; UBQ1	PPL-08-_T3__G05	113	2.00E-29	82	DN554190
			Ubiquitin extension protein 1; UBQ1	PPL-14-H11	170	1.00E-46	85	DN554855
AT5G55160	Small ubiquitin-like modifier 2; SUMO	Ubiquitin like conjugating enzyme activity	Small ubiquitin-like modifier 2; SUMO	PPL-10-A04	228	5.00E-64	87	DN554411
**L. Unknown**								
AT1G68660	Expressed protein	Unknown proteins	Unknown protein	PPL-06-G06	103	3.00E-26	80	DN553992
AT2G07739	Expressed protein	Unknown proteins	Unknown protein	PPL-02-H02	424	4.00E-123	95	DN553049
AT5G64816	Expressed protein	Unknown proteins	Unknown protein	PPL-01-B11B11	141	8.00E-38	82	DN552912

[a]AGI represents *Arabidopsis* Genome Initiative (AGI) locus identifier that corresponds to each gene represented on the array.

[b] *Arabidopsis* -genome array, containing 22,810 probe sets representing approximately 24,000 gene sequences.

[c] Determined followingt the method of the *Arabidopsis* MIPS (Munich Information Centre for Protein Sequences) fucntional classification scheme.

[d] Gene description of peach orthologs was from the PPL EST sequences extracted from NCBI non-redundant dbESTs.

[e] PPL ESTs represent sequences derived from PPV-infected peach leaf EST library [63].

[f] HSP, High-scoring pair. The BLAST opuput for each transcript was parsed for HSP and its associated E-value. Any hits with the existence of HSP$\geq$ 100 and with an E-value$\leq$ 10-20 was taken as an indicative of significant similarity [69].

[g] Percent sequence similarity of PPV induced *Arabidopsis* transcripts to the PPL uniEST sequences.

[h] GenBank identifiers of the peach orthologs.
