# Supplementary material for: Altered gene expression changes in Arabidopsis leaf tissues and protoplasts in response to Plum pox virus infection
Source: BMC Genomics. 2008 Jul 9;9:325. doi: 10.1186/1471-2164-9-325 (PMC2478689; doi:10.1186/1471-2164-9-325)
Supplement: Additional file 10 — Supplemental Table 8. Cross comparison of genes significantly differentially regulated by PPV (≥ 2.5- or ≤ -2.5-fold) in this study with the Arabidopsis genes regulated by other positive sense RNA viruses. [file 1471-2164-9-325-S10.pdf]

Supplemental Table 8. Cross comparison of genes significantly differentially regulated by PPV (≥ 2.5- or ≤ -2.5-fold) in this study with the *Arabidopsis* genes regulated by other positive sense RNA viruses

AGI locus	Arabidopsis gene differentially regulated in this study	FC[a]	FC[b]	FC; hpi, ssRNA virus	References
**Group A. Genes induced by infections of PPV and other positive sense RNA viruses**					
At1g12010	1-Aminocyclopropane-1-Carboxylate Oxidase, Putative / Acc Oxidase, Putative	3.58		1.7 ; 48; CMV-Y	Ishihara et al. (2004)
At5g20000	26S Proteasome Aaa-Atpase Subunit, Putative	2.72		1.65 ; 48; CMV-Y	Ishihara et al. (2004)
At5g05780	26S Proteasome Non-Atpase Regulatory Subunit 7, Putative	1.95		3.83 ; 48; CMV-Y	Ishihara et al. (2004)
At2g37220	29 Kda Ribonucleoprotein, Chloroplast, Putative / Rna-Binding Protein Cp29, Putative	3.16		1.15 ; 48; CMV-Y	Ishihara et al. (2004)
At4g39980	2-Dehydro-3-Deoxyphosphoheptonate Aldolase 1	1.68		1.94 ; 48; CMV-Y	Ishihara et al. (2004)
At5g23010	2-Isopropylmalate Synthase 3 (Ims3)	2.55		1.48 ; 6; CMV-Y	Ishihara et al. (2004)
At2g25450	2-Oxoglutarate-Dependent Dioxygenase, Putative	2.31		10.3 ; 12; CMV-Y	Ishihara et al. (2004)
At1g79850	30S Ribosomal Protein S17, Chloroplast / Cs17 (Rps17)	2.53		1.99 ; 48; CMV-Y	Ishihara et al. (2004)
At2g31610	40S Ribosomal Protein S3 (Rps3A)	1.56		4.64 ; 48 ; CMV-Y	Ishihara et al. (2004)
At3g14990	4-Methyl-5(B-Hydroxyethyl)-Thiazole Monophosphate Biosynthesis Protein, Putative	1.56		2.26 ; 48 ; CMV-Y	Ishihara et al. (2004)
At1g07320	50S Ribosomal Protein L4, Chloroplast (Cl4)	1.59		1.11 ; 24 ; CMV-Y	Ishihara et al. (2004)
At3g44590	60S Acidic Ribosomal Protein P2 (Rpp2D)	2.25		2.49 ; 12 ; CMV-Y	Ishihara et al. (2004)
At3g55130	ABC Transporter Family Protein	2.14		0.7 ; 3, 5 and 18 ; CMV-Y	Marathe et al. (2004)
At5g64840	ABC Transporter Family Protein	4.34		1.13 ; 6 ; CMV-Y	Ishihara et al. (2004)
At1g49240	Actin 8 (Act8)	2.74		1.63 ; 24 ; CMV-Y	Ishihara et al. (2004)
At2g38440	Actin Microfilament	5.24		1.02 ; 6 ; CMV-Y	Ishihara et al. (2004)
At3g05420	Acyl-Coa Binding Family Protein	2.91		4.74 ; 12 ; CMV-Y	Ishihara et al. (2004)
At4g27780	Acyl-Coa Binding Protein 2 (Acbp2)	4.24		1 ; 6 ; CMV-Y	Ishihara et al. (2004)
At4g16760	Acyl-Coa Oxidase (Acx1)	3.72		1.87 ; 48 ; CMV-Y	Ishihara et al. (2004)
At3g23810	Adenosylhomocysteinase, Putative	1.98		1.26 ; 6 ; CMV-Y	Ishihara et al. (2004)
At4g17330	Agenet Domain-Containing Protein	6.61		3.28 ; 12 ; CMV-Y	Ishihara et al. (2004)
At1g54100	Aldehyde Dehydrogenase, Putative / Antiquitin, Putative	2.35		1.81 ; 24 ; CMV-Y	Ishihara et al. (2004)
At2g37770	Aldo/Keto Reductase Family Protein	3.18		2.1–5.4; 4 ; TMV	Golem and Culver (2003)
At1g11860	Aminomethyltransferase, Putative	2.62		1.39 ; 6; CMV-Y	Ishihara et al. (2004)
At4g13510	Ammonium Transporter 1, Member 1 (Amt1.1)	3.43		1.63 ; 48 ; CMV-Y	Ishihara et al. (2004)
At2g38290	Ammonium Transporter 2 (Amt2)	2.71		3.3 ; 48 ; CMV-Y	Ishihara et al. (2004)
At4g26370	Antitermination Nusb Domain-Containing Protein	2.12		1.24 ; 6 ; CMV-Y	Ishihara et al. (2004)
At5g65010	Asparagine Synthetase 2 (Asn2)	3.66		2.14 ; 12 ; CMV-Y	Ishihara et al. (2004)
At5g19550	Aspartate Aminotransferase, Cytoplasmic Isozyme 1 / Transaminase A (Asp2)	2.99		1.82 ; 48 ; CMV-Y	Ishihara et al. (2004)
At3g54400	Aspartyl Protease Family Protein	1.42		0.75 ; 3, 5 and 18 ; CMV-Y	Marathe et al. (2004)
At4g30190	Atpase 2, Plasma Membrane-Type, Putative	2.64		1.05 ; 48 ; CMV-Y	Ishihara et al. (2004)
At2g42280	Basic Helix-Loop-Helix (Bhlh) Family Protein	10.59		1.77 ; 3, 5 and 18 ; CMV-Y	Marathe et al. (2004)
At4g16430	Basic Helix-Loop-Helix (Bhlh) Family Protein	1.71		1.88 ; 48 ; CMV-Y	Ishihara et al. (2004)
At4g36540	Basic Helix-Loop-Helix (Bhlh) Family Protein	1.98		1.34 ; 3, 5 and 18 ; CMV-Y	Marathe et al. (2004)
At4g15210	Beta-Amylase (Bmy1)	1.71		1.65 ; 48 ; CMV-Y	Ishihara et al. (2004)
At2g26250	Beta-Ketoacyl-Coa Synthase Family (Fiddlehead) (Fdh)	4.36		1.12 ; 6 ; CMV-Y	Ishihara et al. (2004)
At3g49680	Branched-Chain Amino Acid Aminotransferase 3	2.14		1.97 ; 24 ; CMV-Y	Ishihara et al. (2004)
At5g62390	Calmodulin-Binding Family Protein	2.26		1.53 ; 6 ; CMV-Y	Ishihara et al. (2004)
At5g61790	Calnexin 1 (Cnx1)	3.87		4.49 ; 48 ; CMV-Y	Ishihara et al. (2004)
At1g56340	Calreticulin 1 (Crt1)	3.28		5.15 ; 48 ; CMV-Y	Ishihara et al. (2004)
At4g35090	Catalase 2	3.56		1.54 ; 6 ; CMV-Y	Ishihara et al. (2004)
At5g01810	Cbl-Interacting Protein Kinase 15 (Cipk15)	2.24		2.13 ; 48 ; CMV-Y	Ishihara et al. (2004)
At1g72830	CCAAT-Binding Transcription Factor (Cbf-B/Nf-Ya) Family Protein	1.43		2.63 ; 48 ; CMV-Y	Ishihara et al. (2004)
At5g47640	CCAAT-Box Binding Transcription Factor Subunit B	1.25		-5.03 ; 48; CMV-Y	Ishihara et al. (2004)
At3g19820	Cell Elongation Protein / Dwarf1 / Diminuto (Dim)	5.46		1.49 ; 24; CMV-Y	Ishihara et al. (2004)
At5g05170	Cellulose Synthase, Catalytic Subunit (Ath-B)	3.25		1.24 ; 6; CMV-Y	Ishihara et al. (2004)
At1g02205	Cer1 Protein	7.04		0.63 ; 3, 5 and 18; CMV-Y	Marathe et al. (2004)

At5g13930	Chalcone Synthase / Naringenin-Chalcone Synthase	9.13	1.3 ; 12; CMV-Y	Ishihara et al. (2004)
At2g38000	Chaperone Protein Dnaj-Related	2.06	2.1–2.6; 5; CMV-Y	Ishihara et al. (2004)
At2g28000	Chaperonin Alpha Subunit	3.06	1.99 ; 24; CMV-Y	Ishihara et al. (2004)
At3g02530	Chaperonin, Putative	2.02	2.08 ; 24; CMV-Y	Ishihara et al. (2004)
At3g51820	Chlorophyll Synthetase, Putative	1.82	1.23 ; 12; CMV-Y	Ishihara et al. (2004)
At2g30490	Cinnamic Acid 4-Hydroxylase (C4H)	1.84	2.09 ; 48; CMV-Y	Ishihara et al. (2004)
At2g44350	Citrate Synthase, Mitochondrial, Putative	1.70	1.55 ; 6; CMV-Y	Ishihara et al. (2004)
At5g61900	Copine Bonzai1 (Bon1)	1.20	3.64 ; 48; CMV-Y	Ishihara et al. (2004)
At4g37430	Cytochrome P450 81F1 (Cyp81F1) (Cyp91A2)	1.48	5.0–24.4 ; 5 ; TVCV, ORMV, PVX, CMV, TuMV	Whitham et al. (2003)
At4g13770	Cytochrome P450 Family Protein	2.07	1.16 ; 6; CMV-Y	Ishihara et al. (2004)
At4g37330	Cytochrome P450 Family Protein	1.60	2.23 ; 24; CMV-Y	Ishihara et al. (2004)
At1g60420	Dc1 Domain-Containing Protein	1.91	2.3 ; 24; CMV-Y	Ishihara et al. (2004)
At3g58510	Dead Box Rna Helicase, Putative (Rh11)	2.52	2.57 ; 12; CMV-Y	Ishihara et al. (2004)
At4g00660	Dead/Deah Box Helicase, Putative	2.29	1.82 ; 12; CMV-Y	Ishihara et al. (2004)
At2g47940	Degp2 Protease (Degp2)	3.41	1.1 ; 12; CMV-Y	Ishihara et al. (2004)
At1g72040	Deoxynucleoside Kinase Family	3.84	1.29 ; 6; CMV-Y	Ishihara et al. (2004)
At5g23450	Diacylglycerol Kinase Family Protein	2.57	-1.59 ; 48; CMV-Y	Ishihara et al. (2004)
At3g56940	Dicarboxylate Diiron Protein, Putative (Crd1)	2.53	1.41 ; 24; CMV-Y	Ishihara et al. (2004)
At4g33300	Disease Resistance Protein (Cc-Nbs-Lrr Class), Putative	2.10	4.57 ; 48; CMV-Y	Ishihara et al. (2004)
At2g20560	DNAJ Heat Shock Family Protein	3.75	0.62 ; 3, 5 and 18; CMV-Y	Marathe et al. (2004)
At3g44110	DNAJ Heat Shock Protein, Putative (J3)	4.66	1.4 ; 24; CMV-Y	Ishihara et al. (2004)
At1g59610	Dynamin-Like Protein, Putative (Adl3)	4.87	3.25 ; 48; CMV-Y	Ishihara et al. (2004)
At4g24510	Eceriferum Protein (Cer2)	1.83	0.6 ; 3, 5 and 18; CMV-Y	Marathe et al. (2004)
At4g29060	Elongation Factor Ts Family Protein	3.50	1.08 ; 12; CMV-Y	Ishihara et al. (2004)
At4g20360	Elongation Factor Tu / Ef-Tu (Tufa)	1.44	1.39 ; 6; CMV-Y	Ishihara et al. (2004)
At2g46280	Eukaryotic Translation Initiation Factor 3 ; Eif-3	2.06	1.25 ; 48; CMV-Y	Ishihara et al. (2004)
At1g26770	Expansin, Putative (Exp10)	2.68	0.68 ; 3, 5 and 18; CMV-Y	Marathe et al. (2004)
At1g09340	Expressed Protein	2.17	1.23 ; 6; CMV-Y	Ishihara et al. (2004)
At1g18620	Expressed Protein	2.57	1.39 ; 3, 5 and 18; CMV-Y	Marathe et al. (2004)
At1g27200	Expressed Protein	2.05	2.7–4.4; 5 ; TVCV, ORMV, PVX, CMV, TuMV	Whitham et al. (2003)
At1g56660	Expressed Protein	4.04	2.23 ; 24; CMV-Y	Ishihara et al. (2004)
At1g64680	Expressed Protein	2.60	1.18 ; 6; CMV-Y	Ishihara et al. (2004)
At1g71970	Expressed Protein	3.46	1.36 ; 3, 5 and 18; CMV-Y	Marathe et al. (2004)
At1g77500	Expressed Protein	2.24	1.24 ; 24; CMV-Y	Ishihara et al. (2004)
At1g79270	Expressed Protein	2.09	0.68 ; 3, 5 and 18; CMV-Y	Marathe et al. (2004)
At2g37660	Expressed Protein	1.83	1.42 ; 6; CMV-Y	Ishihara et al. (2004)
At3g04630	Expressed Protein	3.06	0.68 ; 3, 5 and 18; CMV-Y	Marathe et al. (2004)
At3g58600	Expressed Protein	1.77	5.99 ; 48; CMV-Y	Ishihara et al. (2004)
At3g63170	Expressed Protein	2.15	1.02 ; 12; CMV-Y	Ishihara et al. (2004)
At4g28080	Expressed Protein	6.62	1.03 ; 24; CMV-Y	Ishihara et al. (2004)
At4g28910	Expressed Protein	4.51	1.03 ; 6; CMV-Y	Ishihara et al. (2004)
At5g05250	Expressed Protein	1.59	1.6 ; 3, 5 and 18; CMV-Y	Marathe et al. (2004)
At3g17710	F-Box Family Protein	2.21	-2.1; 14; TMV	Ishihara et al. (2004)
At5g54160	Flavonol 3-O-Methyltransferase 1	2.40	3.02 ; 24; CMV-Y	Ishihara et al. (2004)
At3g52930	Fructose-Bisphosphate Aldolase, Putative	5.32	2.02 ; 12; CMV-Y	Ishihara et al. (2004)
At1g74470	Geranylgeranyl Reductase	1.91	1.31 ; 12; CMV-Y	Ishihara et al. (2004)
At1g31910	Ghmp Kinase Family Protein	2.65	5.3 ; 48; CMV-Y	Ishihara et al. (2004)
At5g14920	Gibberellin-Regulated Family Protein	3.04	1.84 ; 24; CMV-Y	Ishihara et al. (2004)
At2g13440	Glucose-Inhibited Division Family A Protein	1.64	7.12 ; 12; CMV-Y	Ishihara et al. (2004)
At1g65960	Glutamate Decarboxylase 2 (Gad 2)	1.74	4.94 ; 12; CMV-Y	Ishihara et al. (2004)
At5g04140	Glutamate Synthase (Glu1) / Ferredoxin-Dependent Glutamate Synthase (Fd-Gogat 1)	2.90	1.22 ; 6; CMV-Y	Ishihara et al. (2004)
At5g63570	Glutamate-1-Semialdehyde 2,1-Aminomutase 1 (Gsa 1)	2.33	3.62 ; 48; CMV-Y	Ishihara et al. (2004)

At4g23100	Glutamate-Cysteine Ligase / Gamma-Glutamylcysteine Synthetase (Gsh1)	2.48		1.45 ; 12; CMV-Y	Ishihara et al. (2004)
At1g30400	Glutathione S-Conjugate Abc Transporter (Mrp1)	4.59		1.32 ; 24; CMV-Y	Ishihara et al. (2004)
At1g78370	Glutathione S-Transferase, Putative	1.43		1.22 ; 6; CMV-Y	Ishihara et al. (2004)
At3g04120	Glyceraldehyde-3-Phosphate Dehydrogenase, Cytosolic (Gapc)	3.52		1.81 ; 48; CMV-Y	Ishihara et al. (2004)
At2g26080	Glycine Dehydrogenase (Decarboxylating), Putative	6.53		1.38 ; 24; CMV-Y	Ishihara et al. (2004)
At4g33010	Glycine Dehydrogenase (Decarboxylating), Putative	4.58		1.3 ; 6; CMV-Y	Ishihara et al. (2004)
At4g37930	Glycine Hydroxymethyltransferase	1.85		1.21 ; 12; CMV-Y	Ishihara et al. (2004)
At1g52400	Glycosyl Hydrolase Family 1 Protein / Beta-Glucosidase, Putative (Bg1)	2.80		4.78 ; 12; CMV-Y	Ishihara et al. (2004)
At2g18700	Glycosyl Transferase Family 20 Protein / Trehalose-Phosphatase Family Protein	3.55		1.8 ; 4; TMV	Golem and Culver (2003)
At3g07160	Glycosyl Transferase Family 48 Protein	2.34		3.33 ; 48; CMV-Y	Ishihara et al. (2004)
At5g56030	Heat Shock Protein 81-2 (Hsp81-2)	4.63		0.67 ; 3, 5 and 18; CMV-Y	Marathe et al. (2004)
At5g56000	Heat Shock Protein 81-4 (Hsp81-4)	4.75		0.65 ; 3, 5 and 18; CMV-Y	Marathe et al. (2004)
At1g56530	Hydroxyproline-Rich Glycoprotein Family Protein	8.00	7.94	0.73 ; 3, 5 and 18; CMV-Y	Marathe et al. (2004)
At2g39050	Hydroxyproline-Rich Glycoprotein Family Protein	2.53		1.67 ; 12; CMV-Y	Ishihara et al. (2004)
At5g09530	Hydroxyproline-Rich Glycoprotein Family Protein	90.14		4.32 ; 48; CMV-Y	Ishihara et al. (2004)
At5g56650	Iaa-Amino Acid Hydrolase 3 (Iar3) (Ill1)	1.62		1.35 ; 12; CMV-Y	Ishihara et al. (2004)
At5g10170	Inositol-3-Phosphate Synthase, Putative	2.12		1.28 ; 24; CMV-Y	Ishihara et al. (2004)
At4g39240	Kelch Repeat-Containing F-Box Family Protein	1.28		1.26 ; 6; CMV-Y	Ishihara et al. (2004)
At3g16630	Kinesin Motor Family Protein	2.23		1.32 ; 48; CMV-Y	Ishihara et al. (2004)
At3g12740	Lem3 (Ligand-Effect Modulator 3) Family Protein / Cdc50 Family Protein	1.62		1.48 ; 24; CMV-Y	Ishihara et al. (2004)
At1g28440	Leucine-Rich Repeat Transmembrane Protein Kinase, Putative	1.90		2.76 ; 48; CMV-Y	Ishihara et al. (2004)
At1g73080	Leucine-Rich Repeat Transmembrane Protein Kinase, Putative	1.83		0.74 ; 3, 5 and 18; CMV-Y	Marathe et al. (2004)
At5g28540	Luminal Binding Protein 1 (Bip-1) (Bp1)	4.60		4.8 ; 48; CMV-Y	Ishihara et al. (2004)
At2g36830	Major Intrinsic Family Protein / Mip Family Protein	5.91		1.27 ; 12; CMV-Y	Ishihara et al. (2004)
At5g09660	Malate Dehydrogenase, Glyoxysomal	1.58		1.36 ; 12; CMV-Y	Ishihara et al. (2004)
At3g18830	Mannitol Transporter, Putative	1.80		1.48 ; 48; CMV-Y	Ishihara et al. (2004)
At5g12080	Mechanosensitive Ion Channel Domain-Containing Protein	2.29		1.85 ; 24; CMV-Y	Ishihara et al. (2004)
At1g15340	Methyl-Cpg-Binding Domain-Containing Protein	4.97		1.41 ; 6; CMV-Y	Ishihara et al. (2004)
At4g34030	Methylcrotonyl-Coa Carboxylase Beta Chain	2.86		1.02 ; 6; CMV-Y	Ishihara et al. (2004)
At1g25380	Mitochondrial Substrate Carrier Family Protein	1.42		2.24 ; 48; CMV-Y	Ishihara et al. (2004)
At2g43790	Mitogen-Activated Protein Kinase, Putative / Mapk, Putative (Mpk6)	1.45		3.42 ; 12; CMV-Y	Ishihara et al. (2004)
At1g28010	Multidrug Resistance P-Glycoprotein, Putative	3.11		0.63 ; 3, 5 and 18; CMV-Y	Marathe et al. (2004)
At1g05320	Myosin-Related	2.53		1.13 ; 48; CMV-Y	Ishihara et al. (2004)
At1g54010	Myrosinase-Associated Protein, Putative	5.50		4.44 ; 24; CMV-Y	Ishihara et al. (2004)
At5g17770	Nadh-Cytochrome B5 Reductase	2.03		1.67 ; 6; CMV-Y	Ishihara et al. (2004)
At5g04950	Nicotianamine Synthase, Putative	2.21		1.96 ; 6; CMV-Y	Ishihara et al. (2004)
At1g37130	Nitrate Reductase 2 (Nr2)	3.09		1.3 ; 6; CMV-Y	Ishihara et al. (2004)
At5g63310	Nucleotide Diphosphate Kinase Ii, Chloroplast (Ndpk2)	1.61		1.82 ; 12; CMV-Y	Ishihara et al. (2004)
At5g64290	Oxoglutarate/Malate Translocator, Putative	4.90		1.15 ; 6; CMV-Y	Ishihara et al. (2004)
At3g09300	Oxysterol-Binding Family Protein	1.98		1.9 ; 24; CMV-Y	Ishihara et al. (2004)
At3g54920	Pectate Lyase, Putative / Powdery Mildew Susceptibility Protein (Pmr6)	1.40		1.03 ; 12; CMV-Y	Ishihara et al. (2004)
At1g11580	Pectin Methylesterase, Putative	1.79		2.2–3.8; 4; CMV-Y	Ishihara et al. (2004)
At4g33220	Pectinesterase Family Protein	1.49		6.99 ; 48; CMV-Y	Ishihara et al. (2004)
At2g15690	Pentatricopeptide (Ppr) Repeat-Containing Protein	3.78		1.02 ; 12; CMV-Y	Ishihara et al. (2004)
At3g04760	Pentatricopeptide (Ppr) Repeat-Containing Protein	1.80		1.13 ; 48; CMV-Y	Ishihara et al. (2004)
At1g12470	Pep3/Vps18/Deep Orange Family Protein	2.58		2 ; 24; CMV-Y	Ishihara et al. (2004)
At2g42600	Phosphoenolpyruvate Carboxylase, Putative / Pep Carboxylase, Putative (Ppc2)	5.26		1.53 ; 12; CMV-Y	Ishihara et al. (2004)
At3g18000	Phosphoethanolamine N-Methyltransferase 1 / Peamt 1 (Nmt1)	2.73		1.68 ; 24; CMV-Y	Ishihara et al. (2004)
At5g56630	Phosphofructokinase Family Protein	2.56		2.09 ; 24; CMV-Y	Ishihara et al. (2004)
At5g61580	Phosphofructokinase Family Protein	1.31		3.17 ; 48; CMV-Y	Ishihara et al. (2004)
At1g01610	Phospholipid/Glycerol Acyltransferase Family Protein	4.83		0.69 ; 3, 5 and 18; CMV-Y	Marathe et al. (2004)
At1g32060	Phosphoribulokinase (Prk) / Phosphopentokinase	4.77		1.22 ; 6; CMV-Y	Ishihara et al. (2004)

At3g19850	Phototropic-Responsive Nph3 Family Protein	1.55	1.55 ; 3, 5 and 18; CMV-Y	Marathe et al. (2004)
At5g17870	Plastid-Specific Ribosomal Protein-Related	3.03	1.18 ; 6; CMV-Y	Ishihara et al. (2004)
At1g76100	Plastocyanin	1.96	1.51 ; 24; CMV-Y	Ishihara et al. (2004)
At5g65260	Polyadenylate-Binding Protein Family Protein / Pabp Family Protein	2.13	1.94 ; 12; CMV-Y	Ishihara et al. (2004)
At2g40540	Potassium Transporter, Putative (Kt2)	2.38	1.33 ; 6; CMV-Y	Ishihara et al. (2004)
At3g43720	Protease Inhibitor/Seed Storage/Lipid Transfer Protein (Ltp) Family Protein	1.99	0.7 ; 3, 5 and 18; CMV-Y	Marathe et al. (2004)
At1g21750	Protein Disulfide Isomerase, Putative	4.63	6.77 ; 48; CMV-Y	Ishihara et al. (2004)
At1g77510	Protein Disulfide Isomerase, Putative	4.22	3.86 ; 48; CMV-Y	Ishihara et al. (2004)
At3g08760	Protein Kinase Family Protein	1.96	2.0–2.5 ; 4 ; TVCV, ORMV, PVX, CMV, TuMV	Whitham et al. (2003)
At4g32250	Protein Kinase Family Protein	1.92	2.08 ; 24; CMV-Y	Ishihara et al. (2004)
At5g02760	Protein Phosphatase 2C Family Protein / Pp2C Family Protein	1.63	1.67 ; 3, 5 and 18; CMV-Y	Marathe et al. (2004)
At3g05640	Protein Phosphatase 2C, Putative / Pp2C, Putative	2.55	0.53 ; 3, 5 and 18; CMV-Y	Marathe et al. (2004)
At4g01690	Protoporphyrinogen Oxidase (Ppox)	3.91	1.03 ; 12; CMV-Y	Ishihara et al. (2004)
At2g15190	Pseudogene, Ulp1 Protease Family	21.63	3.22 ; 3, 5 and 18; CMV-Y	Marathe et al. (2004)
At2g46790	Pseudo-Response Regulator 9 (Aprr9) / Timing Of Cab Expression 1-Like Protein (Tl1)	1.61	0.73 ; 3, 5 and 18; CMV-Y	Marathe et al. (2004)
At2g36580	Pyruvate Kinase, Putative	3.83	2.27 ; 48; CMV-Y	Ishihara et al. (2004)
At1g19880	Regulator Of Chromosome Condensation (Rcc1) Family Protein	1.63	1.29 ; 6; CMV-Y	Ishihara et al. (2004)
At2g40760	Rhodanese-Like Domain-Containing Protein	1.79	2.2–3.4; 4; TVCV, ORMV, PVX, CMV, TuMV	Whitham et al. (2003)
At2g39730	Ribulose Bisphosphate Carboxylase/Oxygenase Activase / Rubisco Activase	4.11	1.03 ; 6; CMV-Y	Ishihara et al. (2004)
At1g32490	Rna Helicase, Putative	3.99	1.42 ; 48; CMV-Y	Ishihara et al. (2004)
At5g54900	Rna-Binding Protein 45 (Rbp45), Putative	1.79	1.04 ; 6; CMV-Y	Ishihara et al. (2004)
At3g52380	RNA-Binding Protein Cp33, Putative	1.53	1.7 ; 24; CMV-Y	Ishihara et al. (2004)
At4g17230	Scarecrow-Like Transcription Factor 13 (Scl13)	2.92	4.28 ; 48; CMV-Y	Ishihara et al. (2004)
At3g17410	Serine/Threonine Protein Kinase, Putative	3.36	1.95 ; 48; CMV-Y	Ishihara et al. (2004)
At3g58040	Seven In Absentia (Sina) Family Protein	2.88	1.62 ; 48; CMV-Y	Ishihara et al. (2004)
At5g49530	SIN-Like Family Protein	2.23	2.43 ; 12; CMV-Y	Ishihara et al. (2004)
At5g53120	Spermidine Synthase, Putative / Putrescine Aminopropyltransferase, Putative	2.30	1.92 ; 12; CMV-Y	Ishihara et al. (2004)
At3g10420	Sporulation Protein-Related	3.27	3.47 ; 48; CMV-Y	Ishihara et al. (2004)
At5g66760	Succinate Dehydrogenase (Ubiquinone) Flavoprotein	3.01	1.48 ; 24; CMV-Y	Ishihara et al. (2004)
At1g12520	Superoxide Dismutase Copper Chaperone, Putative	2.93	2.6–4.3 ; 5; TVCV, ORMV, PVX, CMV, TuMV	Whitham et al. (2003)
At1g22930	T-Complex Protein 11	2.26	1.66 ; 48; CMV-Y	Ishihara et al. (2004)
At3g62770	Transport Protein-Related	2.62	2.67 ; 48; CMV-Y	Ishihara et al. (2004)
At5g24380	Transporter, Putative	1.28	1.25 ; 48; CMV-Y	Ishihara et al. (2004)
At5g38530	Tryptophan Synthase-Related	5.85	3.06 ; 48; CMV-Y	Ishihara et al. (2004)
At1g20010	Tubulin Beta-5 Chain (Tub5)	1.94	1.29 ; 24; CMV-Y	Ishihara et al. (2004)
At1g24100	Udp-Glucoronosyl/Udp-Glucosyl Transferase Family Protein	2.38	1.5 ; 48; CMV-Y	Ishihara et al. (2004)
At2g38850	Unknown Protein	1.59	2.5–6.5 ; 2 ; TVCV, ORMV, PVX, CMV, TuMV	Whitham et al. (2003)
At3g18890	UV-B regulated protein 1	4.40	1.06 ; 24; CMV-Y	Ishihara et al. (2004)
At1g21380	Vhs Domain-Containing Protein / Gat Domain-Containing Protein	1.41	0.74 ; 3, 5 and 18; CMV-Y	Marathe et al. (2004)
At1g32230	Wwe Domain-Containing Protein / Ceo Protein, Putative (Ceo)	2.55	1.67 ; 48; CMV-Y	Ishihara et al. (2004)
At1g68520	Zinc Finger (B-Box Type) Family Protein	3.20	2.16 ; 6; CMV-Y	Ishihara et al. (2004)
At4g34100	Zinc Finger (C3Hc4-Type Ring Finger) Family Protein	3.70	3.09 ; 48; CMV-Y	Ishihara et al. (2004)
At2g25900	Zinc Finger (Ccch-Type) Family Protein	3.78	1.36 ; 3, 5 and 18; CMV-Y	Marathe et al. (2004)
At4g37910	Heat Shock Protein 70, Mitochondrial, Putative / Hsp70, Mitochondrial, Putative	3.64	1.16 ;10; ORMV / 1.07; ; 10; TuMV	Yang et al. (2007)
At2g47800	Glutathione-Conjugate Transporter (Mrp4)	8.79	1.48;10; ORMV /1.19; 10; TuMV	Yang et al. (2007)
At1g23800	Aldehyde Dehydrogenase, Mitochondrial (Aldh3)	11.08	1.79;10; ORMV /1.92; 10; TuMV	Yang et al. (2007)
At5g09590	Heat Shock Protein 70 / Hsp70 (Hsc70-5)	6.67	1.98;10; ORMV /1.59; 10; TuMV	Yang et al. (2007)
At3g13380	Leucine-Rich Repeat Family Protein	5.09	1.99;10; ORMV /1.63; 10; TuMV	Yang et al. (2007)
At1g09210	Calreticulin 2 (Crt2)	2.71	2.14;10; ORMV /1.43; 10; TuMV	Yang et al. (2007)
At1g30700	Fad-Binding Domain-Containing Protein	4.36	4.97;10; ORMV /3.43; 10; TuMV	Yang et al. (2007)
At5g59990	Expressed protein	2.97	0.742; 3, 5 and 18; CMV-Y	Marathe et al. (2004)

Group B. Genes induced during PPV infection but repressed by infections of other positive sense RNA viruses

At1g62380	1-Aminocyclopropane-1-Carboxylate Oxidase, Putative / Acc Oxidase, Putative	2.62		-2.23 ; 12; CMV-Y	Ishihara et al. (2004)
At3g53460	29 Kda Ribonucleoprotein, Chloroplast / Rna-Binding Protein Cp 29	2.21	-2.86	-1.5 ; 12; CMV-Y	Ishihara et al. (2004)
At1g18450	Actin-Related Protein 4 (Arp4)	1.50		-2.01 ; 6; CMV-Y	Ishihara et al. (2004)
At5g64960	Cyclin-Dependent Kinase, Putative / Cdk, Putative	2.04		-2.32 ; 12; CMV-Y	Ishihara et al. (2004)
At5g25610	Dehydration-Responsive Protein (Rd22)	1.51		-2.43 ; 24; CMV-Y	Ishihara et al. (2004)
At4g10060	Expressed Protein	2.17		–2.1 ; 14; TMV	Ishihara et al. (2004)
At2g21330	Fructose-Bisphosphate Aldolase, Putative	2.69		-4.98 ; 48; CMV-Y	Ishihara et al. (2004)
At5g35630	Glutamine Synthetase (Gs2)	1.37		-2.54 ; 48; CMV-Y	Ishihara et al. (2004)
At1g42970	Glyceraldehyde-3-Phosphate Dehydrogenase	2.10		-1.32 ; 48; CMV-Y	Ishihara et al. (2004)
At5g65310	Homeobox-Leucine Zipper Protein 5 (Hb-5)	1.78		-7.23 ; 48; CMV-Y	Ishihara et al. (2004)
At1g11840	Lactoylglutathione Lyase, Putative / Glyoxalase I, Putative	2.62		-1.66 ; 6; CMV-Y	Ishihara et al. (2004)
At5g47210	Nuclear Rna-Binding Protein, Putative	2.44		-2.04 ; 12; CMV-Y	Ishihara et al. (2004)
At5g46390	Peptidase S41 Family Protein	1.54		-1.84 ; 48; CMV-Y	Ishihara et al. (2004)
At1g21880	Peptidoglycan-Binding Lysm Domain-Containing Protein	1.93		-1.59 ; 48; CMV-Y	Ishihara et al. (2004)
At3g07100	Protein Transport Protein Sec24, Putative	4.39		-1.69 ; 12; CMV-Y	Ishihara et al. (2004)
At1g15290	Tetratricopeptide Repeat (Tpr)-Containing Protein	4.20		-2.13 ; 48; CMV-Y	Ishihara et al. (2004)
At2g29630	Thiamine Biosynthesis Family Protein / Thic Family Protein	2.78		-2.32 ; 48; CMV-Y	Ishihara et al. (2004)
At5g01210	Transferase Family Protein	1.63		-1.42 ; 24; CMV-Y	Ishihara et al. (2004)
At3g02660	Trna Synthetase Class I (W And Y) Family Protein	1.45		-3.5 ; 12; CMV-Y	Ishihara et al. (2004)
At5g67030	Zeaxanthin Epoxidase (Zep) (Aba1)	2.41		-2.77 ; 48; CMV-Y	Ishihara et al. (2004)
At3g51950	Zinc Finger (CCCH-Type) Family Protein	1.29		-1.57 ; 12; CMV-Y	Ishihara et al. (2004)
At1g09750	Chloroplast Nucleoid Dna-Binding Protein-Related	4.53		-13.12; 10; ORMV / -10.24;10; ORMV	Yang et al. (2007)
At4g29020	Glycine-Rich Protein	3.40		-9.67; 10; ORMV / -8.61; 10; ORMV	Yang et al. (2007)
At2g37640	Expansin, Putative (Exp3)	4.33		-7.51; 10; ORMV / -4.91; 10; ORMV	Yang et al. (2007)
At2g20750	Beta-Expansin, Putative (Expb1)	6.08		-5.69; 10; ORMV / -5.99; 10; ORMV	Yang et al. (2007)
At2g32100	Ovate Protein-Related	3.02		-3.87; 10; ORMV / -1.94; 10; ORMV	Yang et al. (2007)
At5g01240	Amino Acid Permease, Putative	2.80		-3.38; 10; ORMV / -2.07; 10; ORMV	Yang et al. (2007)
At2g26330	Leucine-Rich Repeat Family Protein	4.76		-2.97; 10; ORMV / -2.13; 10; ORMV	Yang et al. (2007)
At1g71695	Peroxidase 12 (Per12) (P12) (Prxr6)	3.68		-2.31; 10; ORMV / -1.49; 10; ORMV	Yang et al. (2007)
At5g03650	1,4-Alpha-Glucan Branching Enzyme / Starch Branching Enzyme Class Ii (Sbe2-2)	2.57		-2.26; 10; ORMV / -1.46; 10; ORMV	Yang et al. (2007)
At1g66150	Leucine-Rich Repeat Family Protein	3.94		-1.96; 10; ORMV /-1.63; 10; ORMV	Yang et al. (2007)
At4g32330	Expressed Protein	4.49		-1.94; 10; ORMV / -1.71; 10; ORMV	Yang et al. (2007)
At3g50650	Scarecrow-Like Transcription Factor 7 (Scl7)	3.36		-1.93; 10; ORMV / -1.18; 10; ORMV	Yang et al. (2007)
At5g15450	Heat Shock Protein 100, Putative	5.13		-1.64; 10; ORMV / -1.30; 10; ORMV	Yang et al. (2007)
At5g16820	Heat Shock Factor Protein 3 (Hsf3)	2.60		-1.54; 10; ORMV /-1.26; 10; ORMV	Yang et al. (2007)
At4g24280	Heat Shock Protein 70, Putative / Hsp70, Putative	4.68		-1.42; 10; ORMV / -1.42; 10; ORMV	Yang et al. (2007)
At5g23570	Xs Domain-Containing Protein / Xs Zinc Finger Domain-Containing Protein-Related	3.25		-1.36; 10; ORMV / -1.46; 10; ORMV	Yang et al. (2007)
At2g20610	Aminotransferase, Putative	4.41		-1.17; 10; ORMV / -1.09; 10; ORMV	Yang et al. (2007)
At1g10290	Dynamin-Like Protein 6 (Adl6)	2.69		-1.16; 10; ORMV / -1.1; 10; ORMV	Yang et al. (2007)
At5g49910	Heat Shock Protein 70 / Hsp70 (Hsc70-7)	2.63		-1.06; 10; ORMV /-1.18; 10; ORMV	Yang et al. (2007)
At4g31670	Ubiquitin Carboxyl-Terminal Hydrolase Family Protein	3.94		-1.02; 10; ORMV /-1.08; 10; ORMV	Yang et al. (2007)

Group C. Genes repressed during PPV infection but induced by infections of other positive sense RNA viruses

At1g59860	17.6 Kda Class I Heat Shock Protein (Hsp17.6A-Ci)	-2.31		0.71 ; 3, 5 and 18; CMV-Y	Marathe et al. (2004)
At4g21870	26.5 Kda Class P-Related Heat Shock Protein (Hsp26.5-P)	-12.74		0.74 ; 3, 5 and 18; CMV-Y	Marathe et al. (2004)
At1g33850	40S Ribosomal Protein S15, Putative	-1.92		0.09 ; 3, 5 and 18; CMV-Y	Marathe et al. (2004)
At1g66580	60S Ribosomal Protein L10 (Rpl10C)	-1.55		1.65 ; 48; CMV-Y	Ishihara et al. (2004)
At5g27850	60S Ribosomal Protein L18 (Rpl18C)	-1.49		3.02 ; 48; CMV-Y	Ishihara et al. (2004)
At1g57660	60S Ribosomal Protein L21 (Rpl21E)	-1.95		3.62 ; 48; CMV-Y	Ishihara et al. (2004)
At1g69700	Aba-Responsive Protein (Hva22C)	-2.49		1.9 ; 4; TMV	Golem and Culver (2003)
At5g54720	Ankyrin Repeat Family Protein	-18.14		0.74 ; 3, 5 and 18; CMV-Y	Marathe et al. (2004)

At4g03450	Ankyrin Repeat Family Protein	-2.11		5.5-13.2, 4 ; 4.0–11.5 ; 5; TVCV, ORMV, PVX, CMV, TuMV	Whitham et al. (2003)
At1g64380	Ap2 Domain-Containing Transcription Factor, Putative	-1.72		0.64 ; 3, 5 and 18; CMV-Y	Marathe et al. (2004)
At4g09030	Arabinogalactan-Protein (Agp10)	-1.50		2.77 ; 6; CMV-Y	Ishihara et al. (2004)
At4g12790	Atp-Binding Family Protein	-2.71		1.63 ; 12; CMV-Y	Ishihara et al. (2004)
At3g28930	Avrrpt2-Induced Aig2 Protein (Aig2)	-2.75		4.6–7.6 ; 1; CMV-Y	Ishihara et al. (2004)
At4g14410	Basic Helix-Loop-Helix (Bhlh) Family Protein	-2.51		1.82 ; 12; CMV-Y	Ishihara et al. (2004)
At3g13790	Beta-Fructosidase (Bfruct1) / Beta-Fructofuranosidase	-1.67		2.1–6.0 ; 4; TVCV, ORMV, PVX, CMV, TuMV	Whitham et al. (2003)
At3g47650	Bundle-Sheath Defective Protein 2 Family / Bsd2 Family	-4.86		1.16 ; 6; CMV-Y	Ishihara et al. (2004)
At5g39670	Calcium-Binding Ef Hand Family Protein	-4.19		3.67 ; 48; CMV-Y	Ishihara et al. (2004)
At1g21550	Calcium-Binding Protein, Putative	-3.09		0.63 ; 3, 5 and 18; CMV-Y	Marathe et al. (2004)
At2g41100	Calmodulin-2/3/5 (Cam2) (Cal1)	-4.60		3.2 – 8.8 ; 5; TVCV, ORMV, PVX, CMV, TuMV	Whitham et al. (2003)
At2g43290	Calmodulin-Like Protein (Mss3)	-3.47		4.0–7.3; 5 ; TVCV, ORMV, PVX, CMV, TuMV	Whitham et al. (2003)
At3g50770	Calmodulin-Related Protein, Putative	-2.25		1.34 ; 3, 5 and 18; CMV-Y	Marathe et al. (2004)
At2g30360	Cbl-Interacting Protein Kinase 11 (Cipk11)	-3.21		0.7 ; 3, 5 and 18; CMV-Y	Marathe et al. (2004)
At2g04690	Cellular Repressor Of E1A-Stimulated Genes (Creg) Family	-4.58		1.37 ; 3, 5 and 18; CMV-Y	Marathe et al. (2004)
At4g01700	Chitinase, Putative	-1.50		3.4–8.1 ; 4; TVCV, ORMV, PVX, CMV, TuMV	Whitham et al. (2003)
At4g10340	Chlorophyll A-B Binding Protein Cp26, Chloroplast	-1.21		1.68 ; 24; CMV-Y	Ishihara et al. (2004)
At1g71697	Choline Kinase, Putative	-2.99		0.66 ; 3, 5 and 18; CMV-Y	Marathe et al. (2004)
At4g16500	Cysteine Protease Inhibitor Family Protein / Cystatin Family Protein	-3.01		1.56 ; 48; CMV-Y	Ishihara et al. (2004)
At2g26500	Cytochrome B6F Complex Subunit (Petm), Putative	-3.76		1.6 ; 24; CMV-Y	Ishihara et al. (2004)
At2g41430	Dehydration-Induced Protein (Erd15)	-1.88		1.9 ; 4; TMV	Golem and Culver (2003)
At1g72900	Disease Resistance Protein (Tir-Nbs Class), Putative	-1.14		2.66 ; 48; CMV-Y	Ishihara et al. (2004)
At1g68840	DNA-Binding Protein Rav2 (Rav2)	-2.16		1.34 ; 3, 5 and 18; CMV-Y	Marathe et al. (2004)
At3g14200	Dnaj Heat Shock N-Terminal Domain-Containing Protein	-3.31	10.29	0.45 ; 3, 5 and 18; CMV-Y	Marathe et al. (2004)
At3g21840	E3 Ubiquitin Ligase Scf Complex Subunit Skp1/Ask1 (At7), Putative	-3.15		1.37 ; 3, 5 and 18; CMV-Y	Marathe et al. (2004)
At3g25040	Er Lumen Protein Retaining Receptor, Putative / Hdel Receptor, Putative	-3.18		2.72 ; 48; CMV-Y	Ishihara et al. (2004)
At5g47230	Ethylene-Responsive Element-Binding Factor 5 (Erf5)	-3.03		1.62 ; 3, 5 and 18; CMV-Y	Marathe et al. (2004)
At4g17490	Ethylene-Responsive Element-Binding Protein, Putative	-1.98		1.58 ; 3, 5 and 18; CMV-Y	Marathe et al. (2004)
At1g56580	Expressed Protein	-1.42		0.59 ; 3, 5 and 18; CMV-Y	Marathe et al. (2004)
At5g62520	Expressed Protein	-10.18		0.75 ; 3, 5 and 18; CMV-Y	Marathe et al. (2004)
At5g59080	Expressed Protein	-2.43		1.41 ; 3, 5 and 18; CMV-Y	Marathe et al. (2004)
At5g53160	Expressed Protein	-2.97		1.39 ; 3, 5 and 18; CMV-Y	Marathe et al. (2004)
At5g46295	Expressed Protein	-2.36	-3.08	2.16 ; 3, 5 and 18; CMV-Y	Marathe et al. (2004)
At5g22270	Expressed Protein	-4.95		1.42 ; 48; CMV-Y	Ishihara et al. (2004)
At5g12010	Expressed Protein	-1.59		0.71 ; 3, 5 and 18; CMV-Y	Marathe et al. (2004)
At3g60910	Expressed Protein	-2.24		2.48 ; 24; CMV-Y	Ishihara et al. (2004)
At3g10930	Expressed Protein	-2.20		1.61 ; 3, 5 and 18; CMV-Y	Marathe et al. (2004)
At3g22240	Expressed Protein	-1.14		3.28 ; 48; CMV-Y	Ishihara et al. (2004)
At3g08610	Expressed Protein	-3.23		2.07 ; 12; CMV-Y	Ishihara et al. (2004)
At1g67910	Expressed Protein	-1.90		1.39 ; 3, 5 and 18; CMV-Y	Marathe et al. (2004)
At1g67920	Expressed Protein	-4.97		0.69 ; 3, 5 and 18; CMV-Y	Marathe et al. (2004)
At1g69610	Expressed Protein	-1.95		0.75 ; 3, 5 and 18; CMV-Y	Marathe et al. (2004)
At2g24550	Expressed Protein	-4.71		1.9 ; 4; TMV	Golem and Culver (2003)
At1g55480	Expressed Protein	-1.60		1.6 ; 6; CMV-Y	Ishihara et al. (2004)
At2g36630	Expressed Protein	-1.61		0.7 ; 3, 5 and 18; CMV-Y	Marathe et al. (2004)
At2g32190	Expressed Protein	-2.96		0.68 ; 3, 5 and 18; CMV-Y	Marathe et al. (2004)
At2g34600	Expressed Protein	-4.84		0.64 ; 3, 5 and 18; CMV-Y	Marathe et al. (2004)
At2g44090	Expressed Protein	-1.22		2.68 ; 12; CMV-Y	Ishihara et al. (2004)
At2g23120	Expressed Protein	-5.62		0.71 ; 3, 5 and 18; CMV-Y	Marathe et al. (2004)
At4g08980	F-Box Family Protein (Fbw2)	-2.03		1.59 ; 6; CMV-Y	Ishihara et al. (2004)
At1g77810	Galactosyltransferase Family Protein	-1.56		2.45 ; 48; CMV-Y	Ishihara et al. (2004)
At5g45950	Gdsl-Motif Lipase/Hydrolase Family Protein	-1.95		0.56 ; 3, 5 and 18; CMV-Y	Marathe et al. (2004)

At1g28480	Glutaredoxin Family Protein	-1.38		2.3–9.0 ; 4; TVCV, ORMV, PVX, CMV, TuMV	Whitham et al. (2003)
At2g31570	Glutathione Peroxidase, Putative	-2.53		2.52 ; 12; CMV-Y	Ishihara et al. (2004)
At1g02930	Glutathione S-Transferase, Putative	-3.47		1.38 ; 48; CMV-Y	Ishihara et al. (2004)
At4g19880	Glutathione S-Transferase-Related	-1.44		2.56 ; 48; CMV-Y	Ishihara et al. (2004)
At3g04640	Glycine-Rich Protein	-1.29		3.14 ; 48; CMV-Y	Ishihara et al. (2004)
At1g74230	Glycine-Rich Rna-Binding Protein	-1.93		1.23 ; 12; CMV-Y	Ishihara et al. (2004)
At4g22720	Glycoprotease M22 Family Protein	-1.89		2.2– 8.8; 4 ; TVCV, ORMV, PVX, CMV, TuMV	Whitham et al. (2003)
At1g70090	Glycosyl Transferase Family 8 Protein	-1.39		1.8 ; 14; TMV	Golem and Culver (2003)
At3g04720	Hevein-Like Protein (Hel)	-4.14		1.15 ; 24; CMV-Y	Ishihara et al. (2004)
At5g49590	Hypothetical Protein	-19.17		0.58 ; 3, 5 and 18; CMV-Y	Marathe et al. (2004)
At2g06630	Hypothetical Protein	-3.05	-40.97	0.61 ; 3, 5 and 18; CMV-Y	Marathe et al. (2004)
At1g52240	Kinase partner protein	-8.76		1.05 ; 24; CMV-Y	Ishihara et al. (2004)
At4g02380	Late Embryogenesis Abundant 3 Family Protein / Lea3 Family Protein	-2.33		4.0–27.3 ; 5; TVCV, ORMV, PVX, CMV, TuMV	Whitham et al. (2003)
At1g01470	Late Embryogenesis Abundant Protein, Putative / Lea Protein, Putative	-2.07		0.62 ; 3, 5 and 18; CMV-Y	Marathe et al. (2004)
At5g24210	Lipase Class 3 Family Protein	-1.17		1.87 ; 6; CMV-Y	Ishihara et al. (2004)
At4g21830	Methionine Sulfoxide Reductase Domain-Containing Protein	-3.24		0.71 ; 3, 5 and 18; CMV-Y	Marathe et al. (2004)
At4g21850	Methionine Sulfoxide Reductase Domain-Containing Protein	-7.00		2.88 ; 48; CMV-Y	Ishihara et al. (2004)
At4g04830	Methionine Sulfoxide Reductase Domain-Containing Protein	-2.02		1.61 ; 3, 5 and 18; CMV-Y	Marathe et al. (2004)
At1g65820	Microsomal Glutathione S-Transferase, Putative	-4.09		2.26 ; 48; CMV-Y	Ishihara et al. (2004)
At2g22500	Mitochondrial Substrate Carrier Family Protein	-1.83		6.4–21.8 ; 5; TVCV, ORMV, PVX, CMV, TuMV	Whitham et al. (2003)
At1g61990	Mitochondrial Transcription Termination Factor-Related / Mterf-Related	-1.67		4.9–7.9 ; 5; TVCV, ORMV, PVX, CMV, TuMV	Whitham et al. (2003)
At3g09940	Monodehydroascorbate Reductase, Putative	-3.64		2.48 ; 12; CMV-Y	Ishihara et al. (2004)
At2g37130	Peroxidase 21 (Per21) (P21) (Prxr5)	-2.11		1.09 ; 24; CMV-Y	Ishihara et al. (2004)
At3g18160	Peroxin-3 Family Protein	-1.64		3.71 ; 48; CMV-Y	Ishihara et al. (2004)
At2g20260	Photosystem I Reaction Center Subunit IV, Chloroplast, Putative	-2.39		1.84 ; 12; CMV-Y	Ishihara et al. (2004)
At1g30380	Photosystem I Reaction Center Subunit PSAK, Chloroplast, Putative	-1.71		1.41 ; 6; CMV-Y	Ishihara et al. (2004)
At1g52230	Photosystem I Reaction Center Subunit Vi, Chloroplast, Putative	-2.03		1.66 ; 12; CMV-Y	Ishihara et al. (2004)
At2g30570	Photosystem I Reaction Center W Protein-Related	-1.47		2.35 ; 12; CMV-Y	Ishihara et al. (2004)
At5g65870	Phytosulfokines 5 (Psk5)	-4.91		0.73 ; 3, 5 and 18; CMV-Y	Marathe et al. (2004)
At1g20340	Plastocyanin	-1.45		1.89 ; 24; CMV-Y	Ishihara et al. (2004)
At1g10640	Polygalacturonase, Putative / Pectinase, Putative	-5.53		1.56 ; 12; CMV-Y	Ishihara et al. (2004)
At5g40670	Pq-Loop Repeat Family Protein / Transmembrane Family Protein	-3.09		1.69 ; 48; CMV-Y	Ishihara et al. (2004)
At4g07390	Pq-Loop Repeat Family Protein / Transmembrane Family Protein	-2.32		2.7 ; 12; CMV-Y	Ishihara et al. (2004)
At4g12480	Protease Inhibitor/Seed Storage/Lipid Transfer Protein (Ltp) Family Protein	-6.69		1.45 ; 3, 5 and 18; CMV-Y	Marathe et al. (2004)
At4g11890	Protein Kinase Family Protein	-2.73		0.54 ; 3, 5 and 18; CMV-Y	Marathe et al. (2004)
At4g28400	Protein Phosphatase 2C, Putative / Pp2C, Putative	-1.87		4.58 ; 48; CMV-Y	Ishihara et al. (2004)
At2g16830	Pseudogene, Hypothetical Protein	-2.59		1.48 ; 3, 5 and 18; CMV-Y	Marathe et al. (2004)
At1g57990	Purine Permease-Related	-5.96		1.8 ; 4; TMV	Golem and Culver (2003)
At1g23440	Pyrrolidone-Carboxylate Peptidase Family Protein	-1.94		1.2 ; 48; CMV-Y	Ishihara et al. (2004)
At3g44860	S-Adenosyl-L-Methionine:Carboxyl Methyltransferase Family Protein	-1.72		0.56 ; 3, 5 and 18; CMV-Y	Marathe et al. (2004)
At5g45890	Senescence-Specific Sag12 Protein (Sag12) / Cysteine Proteinase, Putative	-1.95		0.6 ; 3, 5 and 18; CMV-Y	Marathe et al. (2004)
At4g21390	S-Locus Lectin Protein Kinase Family Protein	-2.02		0.67 ; 3, 5 and 18; CMV-Y	Marathe et al. (2004)
At5g49480	Sodium-Inducible Calcium-Binding Protein (Acp1)	-5.44		0.62 ; 3, 5 and 18; CMV-Y	Marathe et al. (2004)
At4g34190	Stress Enhanced Protein 1 (Sep1)	-1.56		1.64 ; 6; CMV-Y	Ishihara et al. (2004)
At3g16050	Stress-Responsive Protein, Putative	-1.68		3.0–4.4 ; 5; TVCV, ORMV, PVX, CMV, TuMV	Whitham et al. (2003)
At3g27380	Succinate Dehydrogenase, Iron-Sulphur Subunit, Mitochondrial (Sdh2-1)	-2.10		1.45 ; 48; CMV-Y	Ishihara et al. (2004)
At4g32150	Synaptobrevin Family Protein	-1.52		1.42 ; 12; CMV-Y	Ishihara et al. (2004)
At3g52400	Syntaxin, Putative (Syp122)	-2.37		2.59 ; 12; CMV-Y	Ishihara et al. (2004)
At1g19050	Two-Component Responsive Regulator / Response Regulator 7 (Arr7)	-2.32		3.5–7.5 ; 1; TVCV, ORMV, PVX, CMV, TuMV	Whitham et al. (2003)
At1g22400	Udp-Glucoronosyl/Udp-Glucosyl Transferase Family Protein	-2.24		1.75 ; 48; CMV-Y	Ishihara et al. (2004)
At3g17610	Unknown Protein	-4.67		1.32 ; 24; CMV-Y	Ishihara et al. (2004)
At1g78410	Vq Motif-Containing Protein	-4.02		1.72 ; 3, 5 and 18; CMV-Y	Marathe et al. (2004)

At5g57530	Xyloglucan:Xyloglucosyl Transferase, Putative	-1.56		1.7 ; 14; TMV	Golem and Culver (2003)
At4g28270	Zinc Finger (C3Hc4-Type Ring Finger) Family Protein	-1.91		1.54 ; 3, 5 and 18; CMV-Y	Marathe et al. (2004)
At2g14560	Expressed protein	-5.9		2.24; 10; ORMV / 1.76; 10; TuMV	Yang et al. (2007)
At2g42360	Zinc finger; C3HC4-type RING finger family protein	-2.7		2.4; 10; ORMV / 2.05; 10; TuMV	Yang et al. (2007)
At4g23150	Protein kinase family Protein	-2.9		3.05; 10; ORMV / 2.48; 10; TuMV	Yang et al. (2007)
At3g48360	Speckle-Type Poz Protein-Related		-4.2	1.49 ;3, 5 and 18; CMV-Y	Marathe et al. (2004)
At5g58700	Phospholipase C		-5.3	0.74; ;3, 5 and 18; CMV-Y	Marathe et al. (2004)
**Group D. Genes repressed by infections of PPV and other positive sense RNA viruses**					
At1g67050	Expressed Protein	-1.84		-2.48 ; 24; CMV-Y	Ishihara et al. (2004)
At5g02160	Expressed Protein	-3.67		–1.9 14; TMV	Golem and Culver (2003)
At5g38980	Expressed Protein	-1.91		–2.1 14; TMV	Golem and Culver (2003)
At5g17650	Glycine/Proline-Rich Protein	-1.64		-2.65 ; 12; CMV-Y	Ishihara et al. (2004)
At4g25490	DRE-binding protein; DREB1B	-3.9		-3.9 ; 10; ORMV / -1.09; 10; TuMV	Yang et al. (2007)
At5g65730	Xyloglucan endotransglycosylase	-2.6		-1.8; 10; ORMV / -1.15 ; 10; TuMV	Yang et al. (2007)
At3g52590	Ubiquitin extension protein 1; UBQ1	-3.0		-1.71; 10; ORMV / -1.04 ; 10; TuMV	Yang et al. (2007)
**Group E. Genes induced during PPV infection but either induced or suppressed by infections of other positive sense RNA viruses**					
At4g39950	Cytochrome P450 79B2, Putative (Cyp79B2)	2.69		1 ; 48; CMV-Y	Ishihara et al. (2004)
				1.9-3.9, 4 ; 5.1–8.1 ; 5 ; TVCV, ORMV, PVX, CMV, TuMV	Whitham et al. (2003)
				1.77;10; ORMV /1.39; 10; TuMV	Yang et al. (2007)
At5g19120	Expressed Protein	2.65		2.1 ; 14; TMV	Golem and Culver (2003)
				-1.61 ; 6; CMV-Y	Ishihara et al. (2004)
At1g19180	Expressed Protein	1.89		3.51 ; 24; CMV-Y	Ishihara et al. (2004)
				0.74 ; 3, 5 and 18; CMV-Y	Marathe et al. (2004)
At1g13090	Cytochrome P450 71B28, Putative (Cyp71B28)	2.59		1.4 ; 12; CMV-Y	Ishihara et al. (2004)
				7.8–38.9 ; 5 ; TVCV, ORMV, PVX, CMV, TuMV	Whitham et al. (2003)
				-1.19; 10; ORMV / -1.13; 10; ORMV	Yang et al. (2007)
At4g34390	Extra-Large Guanine Nucleotide Binding Protein, Putative / G-Protein, Putative	2.79		2.76 ; 48; CMV-Y	Ishihara et al. (2004)
				3.0 – 15.3 ; 5; TVCV, ORMV, PVX, CMV, TuMV	Whitham et al. (2003)
				1.41;10; ORMV /1.17; 10; TuMV	Yang et al. (2007)
At5g02490	Heat Shock Cognate 70 Kda Protein 2 (Hsc70-2) (Hsp70-2)	1.81		1.56 ; 12; CMV-Y	Ishihara et al. (2004)
				8.9-88.4, 4 ; 5.3–98.3 ; 5; TVCV, ORMV, PVX, CMV, TuMV	Whitham et al. (2003)
At3g12580	Heat Shock Protein 70, Putative / Hsp70, Putative	5.24		2.7-11.7, 2 ; 1.9–5.6 ; 5 ; TVCV, ORMV, PVX, CMV, TuMV	Whitham et al. (2003)
				0.55 ; 3, 5 and 18; CMV-Y	Marathe et al. (2004)
				4.42;10; ORMV /3.19; 10; TuMV	Yang et al. (2007)
At4g16660	Heat Shock Protein 70, Putative / Hsp70, Putative	3.08		4.27 ; 48; CMV-Y	Ishihara et al. (2004)
				4.7–24.3 ; 5 ; TVCV, ORMV, PVX, CMV, TuMV	Whitham et al. (2003)
				2.2;10; ORMV /1.34; 10; TuMV	Yang et al. (2007)
At5g52640	Heat Shock Protein 81-1 (Hsp81-1) / Heat Shock Protein 83 (Hsp83)	6.36		2.4–6.2 ; 5 ; TVCV, ORMV, PVX, CMV, TuMV	Whitham et al. (2003)
				0.66 ; 3, 5 and 18; CMV-Y	Marathe et al. (2004)
At1g80440	Kelch Repeat-Containing F-Box Family Protein	2.16		1.7 ; 4 ; TMV	Golem and Culver (2003)
				1.38 ; 3, 5 and 18; CMV-Y	Marathe et al. (2004)
At1g62510	Lipid Transfer Protein (Ltp) Family Protein	2.76		-2.02; 10; ORMV	Yang et al (2007)
				1.41; 10; TuMV	
At1g62570	Flavin-Containing Monooxygenase Family Protein / Fmo Family Protein	3.57		-1.20; 10; ORMV	Yang et al (2007)
				1.12; 10; TuMV	
At5g02500	Heat Shock Cognate 70 Kda Protein 1 (Hsc70-1) (Hsp70-1)	4.56		-1.08; 10; ORMV	Yang et al (2007)
				1.01; 10; TuMV	
At5g50920	Atp-Dependent Clp Protease Atp-Binding Subunit / Clpc	4.21		-1.03; 10; ORMV	Yang et al (2007)
				1.01; 10; TuMV	
At1g78900	Vacuolar Atp Synthase Catalytic Subunit A / V-Atpase A Subunit	4.07		1.01; 10; ORMV	Yang et al (2007)

			-1.17; 10; TuMV	
AT5G51070	Atp-Dependent Clp Protease Atp-Binding Subunit (Clpd), (Erd1)	2.91	4.54 ; 48 ; CMV-Y	Ishihara et al. (2004)
			1.05; 10; ORMV	Yang et al (2007)
			1.05; 10; TuMV	
At5g53950	No Apical Meristem (Nam) Family Protein	7.92	1.09; 10; ORMV	Yang et al (2007)
			-1.15; 10; TuMV	
At1g64780	Ammonium Transporter 1, Member 2 (Amt1.2)	2.85	8.95; 10; ORMV	Yang et al (2007)
			-1.60; 10; TuMV	
At4g19390	Expressed Protein	3.15	2.9–5.24; CMV-Y	Ishihara et al. (2004)
			-1.03; 10; ORMV /-1.28; 10; ORMV	Yang et al. (2007)
At3g46780	Expressed Protein	5.80	1.61 ; 12; CMV-Y	Ishihara et al. (2004)
			-1.34; 10; ORMV / -1.29; 10; ORMV	Yang et al. (2007)
At1g24280	Glucose-6-Phosphate 1-Dehydrogenase, Putative / G6Pd, Putative	2.52	5.7–11.5; 5 ; TVCV, ORMV, PVX, CMV, TuMV	Whitham et al. (2003)
			-1.49; 10; ORMV / -1.97; 10; ORMV	Yang et al. (2007)
At4g38770	Proline-Rich Family Protein (Prp4)	3.60	1.37 ; 6; CMV-Y	Ishihara et al. (2004)
			-1.83; 10; ORMV /-1.22; 10; ORMV	Yang et al. (2007)
At4g24190	Shepherd Protein (Shd) / Clavata Formation Protein, Putative	5.51	3.49 ; 48; CMV-Y	Ishihara et al. (2004)
			1.14; 10; ORMV / 1.12; 10; TuMV	Yang et al. (2007)
At3g09440	Heat Shock Cognate 70 Kda Protein 3 (Hsc70-3) (Hsp70-3)	3.18	0.49 ; 3, 5 and 18; CMV-Y	Marathe et al. (2004)
			1.52;10; ORMV /1.49; 10; TuMV	Yang et al. (2007)
At1g17020	Oxidoreductase, 2Og-Fe(Ii) Oxygenase Family Protein	12.32	3.1–16.6 ; 5 ;TVCV, ORMV, PVX, CMV, TuMV	Whitham et al. (2003)
			2.49;10; ORMV /2.27; 10; TuMV	Yang et al. (2007)

Group F. Genes repressed during PPV infection but induced during infections of more than one positive sense RNA virus

At2g32680	Disease Resistance Family Protein	-2.56	2.95 ; 48; CMV-Y	Ishihara et al. (2004)
			23-177, 2 ; 39.8-172.4, 4 ; 13.5–80.3 ; 5; TVCV, ORMV, PVX, CMV, TuMV	Whitham et al. (2003)
			1.9; 10; ORMV / 1.74; 10; TuMV	Yang et al. (2007)
At5g61600	Ethylene-Responsive Element-Binding Family Protein	-2.20	1.5 ; 4; TMV	Golem and Culver (2003)
			1.59 ; 3, 5 and 18; CMV-Y	Marathe et al. (2004)
At3g57260	Glycosyl Hydrolase Family 17 Protein	-3.65	10.16 ; 48; CMV-Y	Ishihara et al. (2004)
			3.3- 49.5,2 ; 68.6-510.3, 4 ; 13.5–83.2 ; 5 ; TVCV, ORMV, PVX, CMV, TuMV	Whitham et al. (2003)
			1.72; 10; ORMV / 1.52; 10; TuMV	Yang et al. (2007)
At5g20230	Plastocyanin-Like Domain-Containing Protein	-2.62	1.25 ; 48; CMV-Y	Ishihara et al. (2004)
			2.6–5.9 ; 5; TVCV, ORMV, PVX, CMV, TuMV	Whitham et al. (2003)
			1.3; 10; ORMV / 1.28; 10; TuMV	Yang et al. (2007)
At4g23140	Receptor-Like Protein Kinase 5 (Rlk5)	-4.87	0.65 ; 3, 5 and 18; CMV-Y	Marathe et al. (2004)
			6.16 ; 48; CMV-Y	Ishihara et al. (2004)
AT2G15390	Xyloglucan Fucosyltransferase, Putative (Fut4)	-2.75	-1.51; 10; ORMV / -1.02 ; 10; TuMV	Yang et al. (2007)
			5.7–23.1 ; 5 ; TVCV, ORMV, PVX, CMV, TuMV	Whitham et al. (2003)
AT1G23710	Expressed Protein	-4.40	-1.3; 10; ORMV / -1.02 ; 10; TuMV	Yang et al. (2007)
			2.1–5.1 ; 5 ; TVCV, ORMV, PVX, CMV, TuMV	Whitham et al. (2003)
AT2G30590	Wrky Family Transcription Factor	-2.76	-1.17; 10; ORMV / -1.15 ; 10; TuMV	Yang et al. (2007)
			2.7–5.6 ; 5; TVCV, ORMV, PVX, CMV, TuMV	Whitham et al. (2003)
AT2G41110	Calmodulin-2/3/5 (Cam2) (Cal1)	-4.60	-1.08; 10; ORMV / -1.10 ; 10; TuMV	Yang et al. (2007)
			2.4–3.8; 5 ; TVCV, ORMV, PVX, CMV, TuMV	Whitham et al. (2003)
At4g10040	Cytochrome C, Putative	-3.27	2.5–4.0 ; 4; TVCV, ORMV, PVX, CMV, TuMV	Whitham et al. (2003)
			1.2; 10; ORMV / 1.1; 10; TuMV	Yang et al. (2007)
At5g59820	Zinc Finger (C2H2 Type) Family Protein (Zat12)	-3.27	4.0–11.5 ; 5; TVCV, ORMV, PVX, CMV, TuMV	Whitham et al. (2003)
			1.21; 10; ORMV / 1.17; 10; TuMV	Yang et al. (2007)
At1g45145	Thioredoxin H-Type 5 (Trx-H-5) (Toul)	-11.53	2.7–3.4 ; 5 ; TVCV, ORMV, PVX, CMV, TuMV	Whitham et al. (2003)
			1.32; 10; ORMV / / 1.18; 10; TuMV	Yang et al. (2007)
At3g54420	Class IV Chitinase	-4.50	3.6-8.4; 4 ; 2.2–5.2 ; 5 ; TVCV, ORMV, PVX, CMV, TuMV	Whitham et al. (2003)

				1.58; 10; ORMV / / 1.33; 10; TuMV	Yang et al. (2007)
At1g75040	Pathogenesis-Related Protein 5 (Pr-5)	-7.74		5.4-76.5, 2 ; 12.8-156.4, 4 ; 15.4–117.5, 5 ; TVCV, ORMV, PVX, CMV, TuMV	Whitham et al. (2003)
				2.15; 10; ORMV / 1.94; 10; TuMV	Yang et al. (2007)
At1g65500	Expressed Protein	-4.64		2.1–7.0 ; 5; TVCV, ORMV, PVX, CMV, TuMV	Whitham et al. (2003)
				2.74; 10; ORMV / 1.69; 10; TuMV	Yang et al. (2007)
At4g39670	Expressed Protein	-5.55		3.7–29.6 ; 2; TVCV, ORMV, PVX, CMV, TuMV	Whitham et al. (2003)
				3.01; 10; ORMV / / 2.40; 10; TuMV	Yang et al. (2007)
At2g29460	Glutathione S-Transferase, Putative	-3.56		7.0–23.3 ; 5; TVCV, ORMV, PVX, CMV, TuMV	Whitham et al. (2003)
				3.19; 10; ORMV / 2.01; 10; TuMV	Yang et al. (2007)
At2g26150	Heat Shock Transcription Factor Family Protein	-5.00	3.16	0.54 ; 3, 5 and 18; CMV-Y	Marathe et al. (2004)
				3.43; 10; ORMV / 2.63; 10; TuMV	Yang et al. (2007)
At2g33240	Myosin, putative	-13.1		-1.16; 10; ORMV	Yang et al. (2007)
				1.03; 10; TuMV	
At4g27280	Calcium-binding EF hand family protein	-4.8		-1.16; 10; ORMV	Yang et al. (2007)
				1.04; 10; TuMV	
At2g23090	Expressed protein	-4.3		-1.13; 10; ORMV	Yang et al. (2007)
				1.02; 10; TuMV	
At2g22860	Phytosulfokines 2 (Psk2)	-4.90		7.1–28.3 ; 4; TVCV, ORMV, PVX, CMV, TuMV	Whitham et al. (2003)
				1.24; 10; ORMV	Yang et al. (2007)
				-1.09; 10; TuMV	

FC: Fold change.

[a]Fold change in PPV-infecetd *Arabidopsis* leaves.

[b]Fold change in *Arabidopsis* protoplasts transfected with a PPV infectious clone.
